# Dose-sparing effect of two adjuvant formulations with a pandemic influenza A/H7N9 vaccine: A randomized, double-blind, placebo-controlled, phase 1 clinical trial

**DOI:** 10.1371/journal.pone.0274943

**Published:** 2022-10-18

**Authors:** Tazio Vanni, Beatriz C. Thomé, Erin Sparrow, Martin Friede, Christopher B. Fox, Anna Marie Beckmann, Chuong Huynh, Gabriella Mondini, Daniela H. Silveira, Juliana Y. K. Viscondi, Patrícia Emilia Braga, Anderson da Silva, Maria da Graça Salomão, Roberta O. Piorelli, Joane P. Santos, Vera Lúcia Gattás, Maria Beatriz B. Lucchesi, Mayra M. M. de Oliveira, Marcelo E. Koike, Esper G. Kallas, Lucia M. A. Campos, Eduardo B. Coelho, Marilda A. M. Siqueira, Cristiana C. Garcia, Milene Dias Miranda, Terezinha M. Paiva, Maria do Carmo S. T. Timenetsky, Eduardo A. Adami, Milena A. Akamatsu, Paulo Lee Ho, Alexander R. Precioso

**Affiliations:** 1 Instituto Butantan, São Paulo, Brazil; 2 World Health Organization, Geneva, Switzerland; 3 Infectious Disease Research Institute, Seattle, WA, United States of America; 4 Biomedical Advanced Research and Development Authority, Washington, DC, United States of America; 5 Clinics Hospital of the School of Medicine of University of São Paulo, São Paulo, Brazil; 6 Child Institute of the Clinics Hospital of the School of Medicine of University of São Paulo, São Paulo, Brazil; 7 Clinics Hospital of the Medical School of Ribeirão Preto of the University of São Paulo, Ribeirão Preto, Brazil; 8 Oswaldo Cruz Foundation, Rio de Janeiro, Brazil; 9 Adolfo Lutz Institute, São Paulo, Brazil; Public Health England, UNITED KINGDOM

## Abstract

The emergence of potentially pandemic viruses has resulted in preparedness efforts to develop candidate vaccines and adjuvant formulations. We evaluated the dose-sparing effect and safety of two distinct squalene-based oil-in-water adjuvant emulsion formulations (IB160 and SE) with influenza A/H7N9 antigen. This phase I, randomized, double-blind, placebo-controlled, dose-finding trial (NCT03330899), enrolled 432 healthy volunteers aged 18 to 59. Participants were randomly allocated to 8 groups: 1A) IB160 + 15μg H7N9, 1B) IB160 + 7.5μg H7N9, 1C) IB160 + 3.75μg H7N9, 2A) SE + 15μg H7N9, 2B) SE + 7.5μg H7N9, 2C) SE + 3.75μg H7N9, 3) unadjuvanted vaccine 15μg H7N9 and 4) placebo. Immunogenicity was evaluated through haemagglutination inhibition (HI) and microneutralization (MN) tests. Safety was evaluated by monitoring local and systemic, solicited and unsolicited adverse events (AE) and reactions (AR) 7 and 28 days after each study injection, respectively, whereas serious adverse events (SAE) were monitored up to 194 days post-second dose. A greater increase in antibody geometric mean titers (GMT) was observed in groups receiving adjuvanted vaccines. Vaccinees receiving IB160-adjuvanted formulations showed the greatest response in group 1B, which induced an HI GMT increase of 4.7 times, HI titers ≥40 in 45.2% of participants (MN titers ≥40 in 80.8%). Vaccinees receiving SE-adjuvanted vaccines showed the greatest response in group 2A, with an HI GMT increase of 2.5 times, HI titers ≥40 in 22.9% of participants (MN titers ≥40 in 65.7%). Frequencies of AE and AR were similar among groups. Pain at the administration site and headache were the most frequent local and systemic solicited ARs. The vaccine candidates were safe and the adjuvanted formulations have a potential dose-sparing effect on immunogenicity against influenza A/H7N9. The magnitude of this effect could be further explored.

## Introduction

The first human infection with an avian influenza A/H7N9 virus was reported in China in March 2013, and since then 1,567 cases have been documented with high mortality rate (~40%) [1]. Most infections are believed to result from poultry exposure, and no evidence of sustained person-to-person spread of H7N9 has yet been found [2], although person-to-person spread appears to have occurred [3]. The potential for viral adaptation that would facilitate person-to-person transmission is a major concern.

Following the emergence of avian influenza, A/H7N9 virus in humans, WHO has been working with manufacturers for development of candidate vaccines and adjuvant formulations. Although the availability of vaccines is an essential part of pandemic preparedness, the dose-sparing effect of adjuvant formulations is paramount to maximize immunization during a potential pandemic. Adjuvant formulations are particularly important in the case of avian influenza to increase the immunogenicity of vaccines due to poor immunogenicity in humans [4].

Squalene-based adjuvant formulations have been shown to enhance the immune response and to improve the efficacy of inactivated influenza vaccines [5–7]. These adjuvant formulations have been tested with H7N9 vaccines and have shown significantly improved immunogenicity at lower antigen doses (dose-sparing) as compared to un-adjuvanted vaccines [5–8].

Instituto Butantan (IB) produced an inactivated, split-virus influenza A/H7N9 vaccine [9]. This vaccine was tested in an immunogenicity and efficacy ferret challenge study with or without an oil-in-water adjuvant formulation [10]. Instituto Butantan and the Infectious Disease Research Institute have developed their own squalene-based adjuvant formulations, IB160 and SE, respectively. Similar to MF59, IB160 adjuvant is composed of Squalene, Span 85, Tween 80 in citrate buffer. However, they differ in concentration of the components and Ph ranges [11]. SE is a stable oil-in water emulsion, where the oil concentration is 2% (v/v), composed of the excipients squalene (oil), glycerol, egg phosphatidylcholine, surfactant (poloxamer) and ammonium phosphate buffer [12]. The H7N9 antigen combined with squalene-based oil-in-water emulsion formulations demonstrated to be immunogenic and to have acceptable safety profiles in mice and ferrets [9–11].

The purpose of this phase 1 clinical trial was to evaluate the dose-sparing effects and safety of two different adjuvant formulations with influenza A/H7N9 antigen in healthy adults with a homologous prime and boost regimen given 28 days apart.

## Methods

### Study design and procedures

This is a phase 1, multicenter, double-blind, placebo-controlled, multi-arm parallel study. Healthy male and female (nonpregnant) subjects aged 18 to 59 were eligible to participate. A screening visit (T) was performed 1 to 30 days before the day of injection visit (V1) to confirm eligibility prior to further procedures. Full inclusion and exclusion criteria are available in the protocol document (S1 File). A two-dose regimen of each adjuvanted and non-adjuvanted vaccine candidates or placebo were delivered intramuscularly (0.5 mL injection) 28 days apart. Subjects were observed for 30 minutes after administration at the clinical site for recording local and systemic reactions at both vaccination visits. Participants were given and instructed how to use a Participant Diary, a digital thermometer to assess their axillary temperature, and a local reaction measurement device to record reactions that might appear within 7 days post-study injection and record any concomitant medications after both injection visits. A member of the investigator’s team called three days after V1 and V2 (C1 and C2 respectively) to check on the participant’s completion of the Participant Diary and participant’s well-being. On the seventh day after each study injection participants returned to the clinical site (S1 and S2) to have a targeted physical examination performed, to check any adverse events (AEs) that may have occurred, and to have blood collected for testing biochemical and hematological parameters. Twenty-eight days after V1 participants came to the study clinic for collection of blood immunogenicity samples and to have the second study injection (V2). Four weeks after V2 the final study visit (I) occurred for collection of immunogenicity samples and review of unsolicited AE. A last study call (C3) was made 194 days after V2 to enquire about any medical event that would constitute an SAE since the previous visit. All individual study data were recorded in the Case Report Form (CRF).

The study was conducted at the Central Institute and the Children´s Institute, both from the Clinics Hospital of the School of Medicine, University of São Paulo, São Paulo, and Clinics Hospital of the School of Medicine, University of São Paulo in Ribeirão Preto, São Paulo, Brazil. All patients provided written informed consent.

The study was approved by the Brazilian Research National Ethics Council (CONEP– 2306302/2017; CAAE 67517317.0.0000.0068), the Brazilian Health Regulatory Agency (CE 0283986/18-5) and WHO Ethics Research Committee (FLP-01-IB). The trial “Safety and Immunogenicity of H7N9 Influenza Antigen With 2 Adjuvant Formulations in Healthy Adults in Brazil” was registered at ClinicalTrials.gov (NCT03330899) and adhered to the ethical principles of the Declaration of Helsinki, International Conference of Harmonization, and to Good Clinical Practice.

### Randomization and masking

Participants were randomly assigned using a computer-generated randomization schedule. The sequence was generated blinded using random permuted blocks of 8 and 16 patients. The randomization schedules were accessed through an Interactive Web Response System by the clinical site pharmacist. The pharmacist prepared the product under investigation according to the study arm to which the participant was allocated. Participants and study staff, including clinicians and nurses, remained blinded to study arm assignments. Participants were randomly allocated (proportion 1:1:1:1:1:1:1:1) to the following intervention arms: 1A) adjuvant formulation IB160 + 15 μg H7N9, 1B) adjuvant formulation IB160 + 7.5 μg H7N9, 1C) adjuvant formulation IB160 + 3.75 μg H7N9, 2A) adjuvant formulation SE + 15 μg H7N9, 2B) adjuvant formulation SE + 7.5 μg H7N9, 2C) adjuvant formulation SE + 3.75 μg H7N9, 3) non-adjuvanted vaccine 15 μg H7N9 and 4) placebo.

### Adjuvants, vaccines and placebo

Two squalene oil-in-water emulsion adjuvant formulations were evaluated concerning their H7N9 influenza antigen dose-sparing effects. IB160 was manufactured by IB and SE was manufactured by the Infectious Disease Research Institute (IDRI) and donated to IB for this trial [11, 12]. Both formulations were stored in a 10 mL vial containing 1.5 mL. The H7N9 influenza antigen component produced by IB is an inactivated split-virus [9], developed from the Candidate Vaccine Virus A/Shanghai/2/2013(H7N9)-PR8-(IDCDC-RG32A) by the Centers for Disease Control and Prevention (CDC), GA, USA. The H7N9 antigen was supplied in multidose vials (5 doses per vial) with thimerosal added as a preservative. The vaccine antigen was mixed with the adjuvant formulations at the clinical sites prior to study injections. The Placebo produced by IB is a PBS solution with thimerosal added as a preservative, kept in a 10 mL vial containing 1.5 mL. All the study products were stored at 2–8°C.

### Immunogenicity assessment

Antibody testing using haemagglutination-inhibition (HI) and microneutralization (MN) assays were performed immediately before study injections, 28 days after first dose (prior to second dose), 35 days after first dose (only by HI), and 56 days after first dose. Serum samples were tested against the homologous influenza A/Shanghai/2/13 (H7N9) reassortant virus obtained from the US Centers for Disease Control and Prevention.

HI detection of influenza A/H7N9 virus antibodies was performed at Instituto Adolfo Lutz, São Paulo—Brazil, according to the WHO protocol [13] Briefly, the haemagglutinin (HA) protein on the surface of influenza virus agglutinates erythrocytes. The presence of specific anti-HA antibodies will inhibit the agglutination between the virus and the erythrocytes, which is the basis for the HI assay. Additional details of the protocol for serological detection of avian influenza A(H7N9) virus infections by modified horse red blood cells haemagglutination-inhibition assay used have been previously described [13, 14].

MN detection of influenza A/H7N9 virus antibodies was performed at Fundação Oswaldo Cruz, Rio de Janeiro–Brazil, according to the WHO protocol [15]. MN assay is a highly sensitive and specific assay for detecting virus-specific neutralizing antibodies to influenza viruses in human sera. Briefly, the assays is based on the assumption that serum-neutralizing antibodies to influenza HA will inhibit the infection of MDCK cells with the virus. Serially diluted sera are pre-incubated with a standardized amount of virus before the addition of MDCK cells. After overnight incubation, the cells are fixed and the presence of influenza A virus nucleoprotein (NP) protein in infected cells is detected by ELISA [15].

Both institutions received training on the two WHO protocols and they went through a validation process [16–18] conducted by Vismederi, Siena—Italy, prior to start of the clinical trial immunogenicity assessments.

### Endpoints

The primary immunologic endpoints included the proportion of participants who had a haemagglutination inhibition antibody (HI) titer of 40 or higher (seroprotection), the proportion of participants who had 4-fold or greater increase in HI titer from a baseline titer of ≥10 or a titer after vaccination of ≥40 if the baseline titer was <10 (seroconversion), and the geometric mean titers (GMTs) up to 28 days after the second vaccination. As secondary immunogenicity endpoints, the proportion of participants who met the definition of seroprotection in MN tests, the proportion of participants who met the definition of seroconversion in MN tests and the GMTs up to 28 days after the second vaccination were also calculated.

The primary safety endpoints included the number, severity and percentage of participants with solicited and/or unsolicited local and/or systemic AEs during a 7-day period post each study injection. As secondary safety endpoints, the number and percentage of participants with unsolicited AEs for 28 days post each study injection, and all serious adverse events (SAEs) occurring over the study period were calculated. As an exploratory safety endpoint, the number and percentage of participants with adverse events of special interest (AESIs), as listed in the S1 File occurring over the study period, were calculated.

Following international standards, adverse reactions were defined as an AE that had reasonable causal relationship to study injection, as defined by the adapted classification of “Uppsala Monitoring Centre” of the World Health [19], which weremonitored by a Data and Safety Monitoring Board (DSMB). The intensity of ARs was classified as grade one to four according to the Toxicity Grading Scale for Healthy Adult and Adolescent Volunteers Enrolled in Preventive Vaccine Clinical Trials of the US Food and Drug Administration (FDA) [20].

### Statistical analysis

Sample size was approximately 432 subjects, randomized equally into 8 treatment groups of approximately 54 participants per study group. As previous phase 1 studies of H7N9 vaccines [6, 7], the study focused on evaluating the safety of the products under investigation. Therefore, sample size was defined in order to detect frequent adverse events. In each of the active arms (N = 54), there is a 94% power to detect an adverse event with frequency ≥ 5%. For all combined dose levels of vaccine adjuvanted with IB160 (N = 162) or SE (N = 162) there is a 80% power to detect an event with frequency ≥ 1%. The demographic characteristics were compared using chi-square test for categorical variables and non-parametric Kruskal-Wallis test for age. Number (n) and proportion (%) of participants, experiencing each AR were calculated along with two-sided exact (Clopper-Pearson) 95% confidence interval (CI) for each safety endpoint and each dose administration by study group. For each solicited AR occurring up to 28 days post each dose administration, frequency (n,%) of medication use, median (P_50_) and quartiles (P_25_, P_75_) of duration (in days), and median (P_50_) and quartiles (P_25_, P_75_) of start time (in days) were reported, by study group. Values of GMT and geometric mean fold rise (GMFR), for each time point, were summarized by treatment group along with the corresponding two-sided 95% CI. Comparisons of groups, for each time point, were performed by Kruskal-Wallis with Bonferroni correction for multiple comparisons. Seroconversion and seroprotection rates (SCR and SPR, respectively) were calculated for each study group along with its associated 95% CI. Comparisons of groups, for each time point, were performed by chi-square test with Bonferroni correction for multiple comparisons. Statistical analysis was performed using Stata version 13 (Stata Corp LP, College Station, Texas, USA). The significance level was set at *p* < 0.05.

## Results

### Study enrollment and demographic characteristics

**Fig 1:**
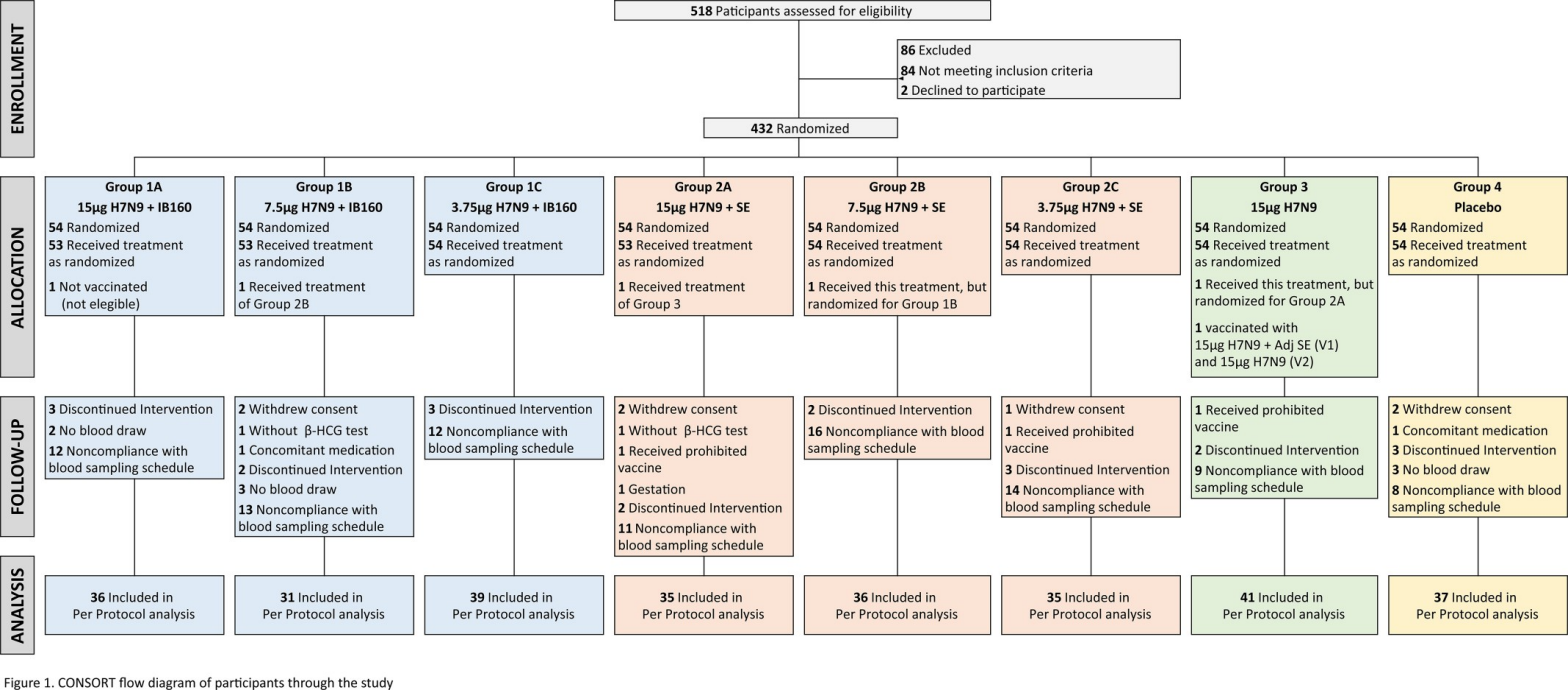
CONSORT flow diagram of participants through the study.

Between September 24, 2018, and April 25, 2019, 518 individuals were assessed for eligibility, and 432 participants were randomized and included in the study. The total number of participants randomized for each study group was 54 (Fig 1). The demographic and baseline characteristics of participants were similar across intervention groups, as described in Table 1. The participants were predominantly white females in their thirties.

**Table 1:** Demographic characteristics, prior seasonal influenza vaccine, and study site, by study group.

	Group 1A		Group 1B		Group 1C		Group 2A		Group 2B		Group 2C		Group 3		Group 4			
VARIABLES	15μg H7N9+ IB160		7.5μg H7N9 + IB160		3.75μg H7N9 + IB160		15μg H7N9 + SE		7.5μg H7N9 + SE		3.75μg H7N9 + SE		15μg H7N9, without adjuvant		Placebo		TOTAL	
	(n = 53)		(n = 53)		(n = 54)		(n = 53)		(n = 55)		(n = 54)		(n = 54)		(n = 54)		(n = 430)	
Age (years)	P_50_	(P_25_-P_75_)	P_50_	(P_25_-P_75_)	P_50_	(P_25_-P_75_)	P_50_	(P_25_-P_75_)	P_50_	(P_25_-P_75_)	P_50_	(P_25_-P_75_)	P_50_	(P_25_-P_75_)	P_50_	(P_25_-P_75_)	P_50_	(P_25_-P_75_)
	38	(30.3–46.6)	35	(27.9–41.5)	35	(29.1–43.2)	39	(28.5–47.2)	37	(29.0–46.6)	36	(25.3–43.9)	34	(24.6–42.6)	35	(26.2–42.1)	35.4	(27.5–44.0)
**Sex**	**n**	**(%)**	**n**	**(%)**	**n**	**(%)**	**n**	**(%)**	**n**	**(%)**	**n**	**(%)**	**n**	**(%)**	**n**	**(%)**	**n**	**(%)**
Female	37	(69.8)	44	(83.0)	37	(68.5)	37	(69.8)	35	(63.6)	35	(64.8)	35	(64.8)	40	(74.1)	300	(69.8)
Male	16	(30.2)	9	(17.0)	17	(31.5)	16	(30.2)	20	(36.4)	19	(35.2)	19	(35.2)	14	(25.9)	130	(30.2)
**Ethnicity**	**n**	**(%)**	**n**	**(%)**	**n**	**(%)**	**n**	**(%)**	**n**	**(%)**	**n**	**(%)**	**n**	**(%)**	**n**	**(%)**	**n**	**(%)**
Asian	0	(0.0)	3	(5.7)	1	(1.9)	3	(5.7)	1	(1.8)	0	(0.0)	1	(1.9)	2	(3.7)	11	(2.6)
Black	6	(11.3)	9	(17.0)	8	(14.8)	4	(7.6)	8	(14.6)	7	(13.0)	13	(24.1)	9	(16.7)	64	(14.9)
Brazilian Indigenous	0	(0.0)	0	(0.0)	0	(0.0)	0	(0.0)	0	(0.0)	0	(0.0)	0	(0.0)	1	(1.9)	1	(0.2)
Multiracial	12	(22.6)	11	(20.8)	15	(27.8)	11	(20.8)	10	(18.2)	10	(18.5)	11	(20.4)	10	(18.5)	90	(20.9)
White	35	(66.0)	30	(56.6)	30	(55.6)	35	(66.0)	36	(65.5)	37	(68.5)	29	(53.7)	32	(59.3)	264	(61.4)
**Prior seasonal influenza vaccine**	**n**	**(%)**	**n**	**(%)**	**n**	**(%)**	**n**	**(%)**	**n**	**(%)**	**n**	**(%)**	**n**	**(%)**	**n**	**(%)**	**n**	**(%)**
None	12	(22.6)	11	(20.8)	15	(27.8)	13	(24.5)	11	(20.0)	16	(29.6)	20	(37.0)	19	(35.2)	117	(27.2)
2017 only	3	(5.7)	4	(7.6)	2	(3.7)	3	(5.7)	5	(9.1)	2	(3.7)	3	(5.6)	1	(1.9)	23	(5.4)
2018 only	3	(5.7)	2	(3.8)	4	(7.4)	4	(7.6)	5	(9.1)	6	(11.1)	2	(3.7)	4	(7.4)	30	(7.0)
2017 and 2018	34	(64.2)	34	(64.2)	33	(61.1)	30	(56.6)	32	(58.2)	29	(53.7)	25	(46.3)	28	(51.9)	245	(57.0)
Unkown*	1	(1.9)	2	(3.8)	0	(0.0)	3	(5.7)	2	(3.6)	1	(1.9)	4	(7.4)	2	(3.7)	15	(3.5)
**Study site**	**n**	**(%)**	**n**	**(%)**	**n**	**(%)**	**n**	**(%)**	**n**	**(%)**	**n**	**(%)**	**n**	**(%)**	**n**	**(%)**	**n**	**(%)**
HCFMUSP	15	(28.3)	15	(28.3)	15	(27.8)	15	(28.3)	15	(27.3)	15	(27.8)	15	(27.8)	15	(27.8)	120	(27.9)
ICr-HCFMUSP	15	(28.3)	15	(28.3)	15	(27.8)	14	(26.4)	15	(27.3)	15	(27.8)	15	(27.8)	15	(27.8)	119	(27.7)
HCFMRP- USP	23	(43.4)	23	(43.4)	24	(44.4)	24	(45.3)	25	(45.5)	24	(44.4)	24	(44.4)	24	(44.4)	191	(44.4)

adj: adjuvant; P_50_: median; P_25_: first quartile; P_75_: third quartile

* any Unknown answer

### Immunogenicity evaluation

#### Haemagglutination inhibition results

Tables 2 and 3 describe the HI data by study group, according to intention-to-treat and per-protocol analysis, respectively. Observed responses after the first study injection (d28) were low in all study groups. After two doses of the candidate vaccines (d56), there was a greater increase in GMT among those who received candidate vaccines with adjuvant formulations. A substantial increase in titers could be detected as soon as one week post second dose. Considering the dose-sparing effect, the GMT results suggest that even the lower antigen dose with the IB160 adjuvant elicited a greater immune response as compared to the higher antigen dose without adjuvant formulations. Among the IB160-adjuvanted groups, the highest HI GMT ratio was observed in group 1B. Among the SE-adjuvanted groups, the highest HI GMT ratio was observed in group 2A. Nonetheless, the difference in titers between group 1B and group 2A was not statistically significant. SCR of group 1B and 2A were 45.2% and 22.9% in the per-protocol analysis. It is worth mentioning that SCR and SPR were essentially identical because nearly all participants were seronegative at baseline.

**Table 2:** Haemagglutination inhibition (HI) data by study group, intention-to-treat analysis.

	Group 1A			Group 1B			Group 1C			Group 2A			Group 2B			Group 2C			Group 3			Group 4		
	15μ H7N9 + IB160			7,5μ H7N9 + IB160			3,75μ H7N9 + IB160			15μ H7N9 + SE			7,5μ H7N9 + SE			3,75μ H7N9 + SE			15μ H7N9, without adj			Placebo		
	n	Value	(95%CI)	n	Value	(95%CI)	n	Value	(95%CI)	n	Value	(95%CI)	n	Value	(95%CI)	n	Value	(95%CI)	n	Value	(95%CI)	n	Value	(95%CI)
**GMT**																								
Pre	51	5.0	(NE)	47	5.0	(NE)	54	5.0	(NE)	49	5.0	(NE)	54	5.0	(NE)	51	5.0	(NE)	52	5.0	(NE)	49	5.0	(NE)
d28	51	6.0	(5.4–6.8)	47	5.8	(5.2–6.4)	54	5.8	(5.2–6.5)	49	5.8	(5.2–6.6)	53	5.5	(5.0–6.1)	51	5.3	(4.9–5.6)	52	5.3	(4.8–5.7)	49	5.1	(4.9–5.2)
d35	50	12.1	(9.8–15.0)	45	14.0	(10.6–18.6)	52	11.3	(8.7–14.7)	48	11.1	(8.4–14.6)	53	7.0	(5.8–8.5)	51	7.8	(6.3–9.7)	52	5.9	(5.1–6.9)	46	5.1	(4.9–5.2)
d56	50	13.8	(10.7–17.8)	44	19.1	(13.8–26.3)	53	14.4	(10.9–19.1)	48	13.3	(9.7–18.4)	54	8.7	(7.0–10.8)	51	8.5	(6.8–10.6)	52	6.1	(5.3–7.1)	48	5.0	(NE)
**GMFR from Pre**																								
d28	51	1.2	(1.1–1.4)	47	1.2	(1.0–1.3)	54	1.2	(1.0–1.3)	49	1.2	(1.0–1.3)	53	1.1	(1.0–1.2)	51	1.1	(1.0–1.1)	52	1.1	(1.0–1.1)	49	1.0	(1.0–1.0)
d35	50	2.4	(2.0–3.0)	45	2.8	(2.1–3.7)	52	2.3	(1.7–2.9)	48	2.2	(1.7–2.9)	53	1.4	(1.2–1.7)	51	1.6	(1.3–1.9)	52	1.2	(1.0–1.4)	46	1.0	(1.0–1.0)
d56	50	2.8	(2.1–3.6)	44	3.8	(2.8–5.3)	53	2.9	(2.2–3.8)	48	2.7	(1.9–3.7)	54	1.7	(1.4–2.2)	51	1.7	(1.4–2.1)	52	1.2	(1.1–1.4)	48	1.0	(1.0–1.0)
**SPR, %**																								
Pre	51	0.0	(0.0–7.0)	47	0.0	(0.0–7.5)	54	0.0	(0.0–6.6)	49	0.0	(0.0–7.3)	54	0.0	(0.0–6.6)	51	0.0	(0.0–7.0)	52	0.0	(0.0–6.8)	49	0.0	(0.0–7.3)
d28	51	0.0	(0.0–7.0)	47	0.0	(0.0–7.5)	54	0.0	(0.0–6.6)	49	2.0	(0.0–10.9)	53	1.9	(0.0–10.1)	51	0.0	(0.0–7.0)	52	1.9	(0.0–10.3)	49	0.0	(0.0–7.3)
d35	50	14.0	(5.8–26.7)	45	22.2	(11.2–37.1)	52	23.1	(12.5–36.8)	48	14.6	(6.1–27.8)	53	5.7	(1.2–15.7)	51	7.8	(2.2–18.9)	52	3.8	(0.5–13.2)	46	0.0	(0.0–7.7)
d56	50	20.0	(10.0–33.7)	44	36.4	(22.4–52.2)	53	32.1	(19.9–46.3)	48	20.8	(10.5–35.0)	54	9.3	(3.1–20.3)	51	9.8	(3.3–21.4)	52	3.8	(0.5–13.2)	48	0.0	(0.0–7.4)
**SCR from Pre, %**																								
d28	51	0.0	(0.0–7.0)	47	0.0	(0.0–7.5)	54	0.0	(0.0–6.6)	49	2.0	(0.0–10.9)	53	1.9	(0.0–10.1)	51	0.0	(0.0–7.0)	52	1.9	(0.0–10.3)	49	0.0	(0.0–7.3)
d35	50	14.0	(5.8–26.7)	45	22.2	(11.2–37.1)	52	23.1	(12.5–36.8)	48	14.6	(6.1–27.8)	53	5.7	(1.2–15.7)	51	7.8	(2.2–18.9)	52	3.8	(0.5–13.2)	46	0.0	(0.0–7.7)
d56	50	20.0	(10.0–33.7)	44	36.4	(22.4–52.2)	53	32.1	(19.9–46.3)	48	20.8	(10.5–35.0)	54	9.3	(3.1–20.3)	51	9.8	(3.3–21.4)	52	3.8	(0.5–13.2)	48	0.0	(0.0–7.4)

adj: adjuvant; 95%CI: 95% confidence interval; GMT: geometric mean titers; GMFR: geometric mean fold rises, i.e., ratio between GMT of baseline and of post-dose; SPR: seroprotection rate (prior and postvaccination HI antibody titers ≥1:40); SCR: seroconversion rate (baseline HI antibody titers <1:10 and postvaccination HI antibody titers ≥1:40, or baseline HI antibody titers ≥1:10 and a postvaccination increase by a factor of four or more); Sample collected at: Pre (prior 1st dose), d28 (prior 2nd dose, 28 days post 1st dose), d35 (7 days post 2nd dose or 35 days post 1st dose), d56 (28 days post 2nd dose or 56 days post 1st dose); NE: not estimable.

**Table 3:** Haemagglutination inhibition (HI) data by study group, per protocol analysis.

	Group 1A			Group 1B			Group 1C			Group 2A			Group 2B			Group 2C			Group 3			Group 4		
	15μ H7N9 + IB160			7,5μ H7N9 + IB160			3,75μ H7N9 + IB160			15μ H7N9 + SE			7,5μ H7N9 + SE			3,75μ H7N9 + SE			15μ H7N9, without adjuvant			Placebo		
	n	Value	(95%CI)	n	Value	(95%CI)	n	Value	(95%CI)	N	Value	(95%CI)	n	Value	(95%CI)	n	Value	(95%CI)	n	Value	(95%CI)	n	Value	(95%CI)
**GMT**																								
Pre	36	5.0	(NE)	31	5.0	(NE)	39	5.0	(NE)	35	5.0	(NE)	36	5.0	(NE)	35	5.0	(NE)	41	5.0	(NE)	37	5.0	(NE)
d28	36	6.3	(5.4–7.4)	31	5.7	(5.1–6.5)	39	6.0	(5.2–6.9)	35	5.9	(5.1–6.7)	36	5.4	(4.9–5.9)	35	5.2	(4.9–5.5)	41	5.3	(4.8–6.0)	37	5.1	(4.9–5.3)
d35	36	11.7	(9.3–14.6)	31	16.0	(11.2–22.8)	39	10.9	(8.2–14.6)	35	10.0	(7.6–13.2)	36	6.3	(5.4–7.4)	35	7.0	(5.7–8.6)	41	6.2	(5.1–7.5)	37	5.1	(4.9–5.3)
d56	36	15.6	(11.8–20.6)	31	23.4	(15.8–34.6)	39	15.0	(10.9–20.8)	35	12.4	(8.8–17.5)	36	7.8	(6.3–9.7)	35	7.7	(6.3–9.5)	41	6.4	(5.3–7.8)	37	5.0	(NE)
**GMFR from Pre**																								
d28	36	1.3	(1.1–1.5)	31	1.1	(1.0–1.3)	39	1.2	(1.0–1.4)	35	1.2	(1.0–1.3)	36	1.1	(1.0–1.2)	35	1.0	(1.0–1.1)	41	1.1	(1.0–1.2)	37	1.0	(1.0–1.0)
d35	36	2.3	(1.9–2.9)	31	3.2	(2.2–4.6)	39	2.2	(1.6–2.9)	35	2.0	(1.5–2.6)	36	1.3	(1.1–1.5)	35	1.4	(1.1–1.7)	41	1.2	(1.0–1.5)	37	1.0	(1.0–1.1)
d56	36	3.1	(2.4–4.1)	31	4.7	(3.2–6.9)	39	3.0	(2.2–4.2)	35	2.5	(1.8–3.5)	36	1.6	(1.3–1.9)	35	1.5	(1.3–1.9)	41	1.3	(1.1–1.6)	37	1.0	(1.0–1.0)
**SPR, %**																								
Pre	36	0.0	(0.0–9.7)	31	0.0	(0.0–11.2)	39	0.0	(0.0–9.0)	35	0.0	(0.0–10.0)	36	0.0	(0.0–9.7)	35	0.0	(0.0–10.0)	41	0.0	(0.0–8.6)	37	0.0	(0.0–9.5)
d28	36	0.0	(0.0–9.7)	31	0.0	(0.0–11.2)	39	0.0	(0.0–9.0)	35	2.0	(0.0–10.0)	36	1.9	(0.0–9.7)	35	0.0	(0.0–10.0)	41	2.4	(0.0–12.9)	37	0.0	(0.0–9.5)
d35	36	11.1	(3.1–26.1)	31	29.0	(14.2–48.0)	39	20.5	(9.3–36.5)	35	11.4	(3.2–26.7)	36	0.0	(0.0–9.7)	35	2.9	(0.1–14.9)	41	4.9	(0.6–16.5)	37	0.0	(0.0–9.5)
d56	36	22.2	(10.1–39.2)	31	45.2	(27.3–64.0)	39	30.8	(17.0–47.6)	35	22.9	(10.4–40.1)	36	5.6	(0.7–18.7)	35	2.9	(0.1–14.9)	41	4.9	(0.6–16.5)	37	0.0	(0.0–9.5)
**SCR from Pre, %**																								
d28	36	0.0	(0.0–9.7)	31	0.0	(0.0–11.2)	39	0.0	(0.0–9.0)	35	2.0	(0.0–10.0)	36	1.9	(0.0–9.7)	35	0.0	(0.0–10.0)	41	2.4	(0.0–12.9)	37	0.0	(0.0–9.5)
d35	36	11.1	(3.1–26.1)	31	29.0	(14.2–48.0)	39	20.5	(9.3–36.5)	35	11.4	(3.2–26.7)	36	0.0	(0.0–9.7)	35	2.9	(0.1–14.9)	41	4.9	(0.6–16.5)	37	0.0	(0.0–9.5)
d56	36	22.2	(10.1–39.2)	31	45.2	(27.3–64.0)	39	30.8	(17.0–47.6)	35	22.9	(10.4–40.1)	36	5.6	(0.7–18.7)	35	2.9	(0.1–14.9)	41	4.9	(0.6–16.5)	37	0.0	(0.0–9.5)

adj: adjuvant; 95%CI: 95% confidence interval; GMT: geometric mean titers; GMFR: geometric mean fold rises, i.e., ratio between GMT of baseline and of post-dose; SPR: seroprotection rate (prior and postvaccination HI antibody titers ≥1:40); SCR: seroconversion rate (baseline HI antibody titers <1:10 and postvaccination HI antibody titers ≥1:40, or baseline HI antibody titers ≥1:10 and a postvaccination increase by a factor of four or more); Sample collected at: Pre (prior 1st dose), d28 (prior 2nd dose, 28 days post 1st dose), d35 (7 days post 2nd dose or 35 days post 1st dose), d56 (28 days post 2nd dose or 56 days post 1st dose); NE: not estimable.

**Fig 2:**
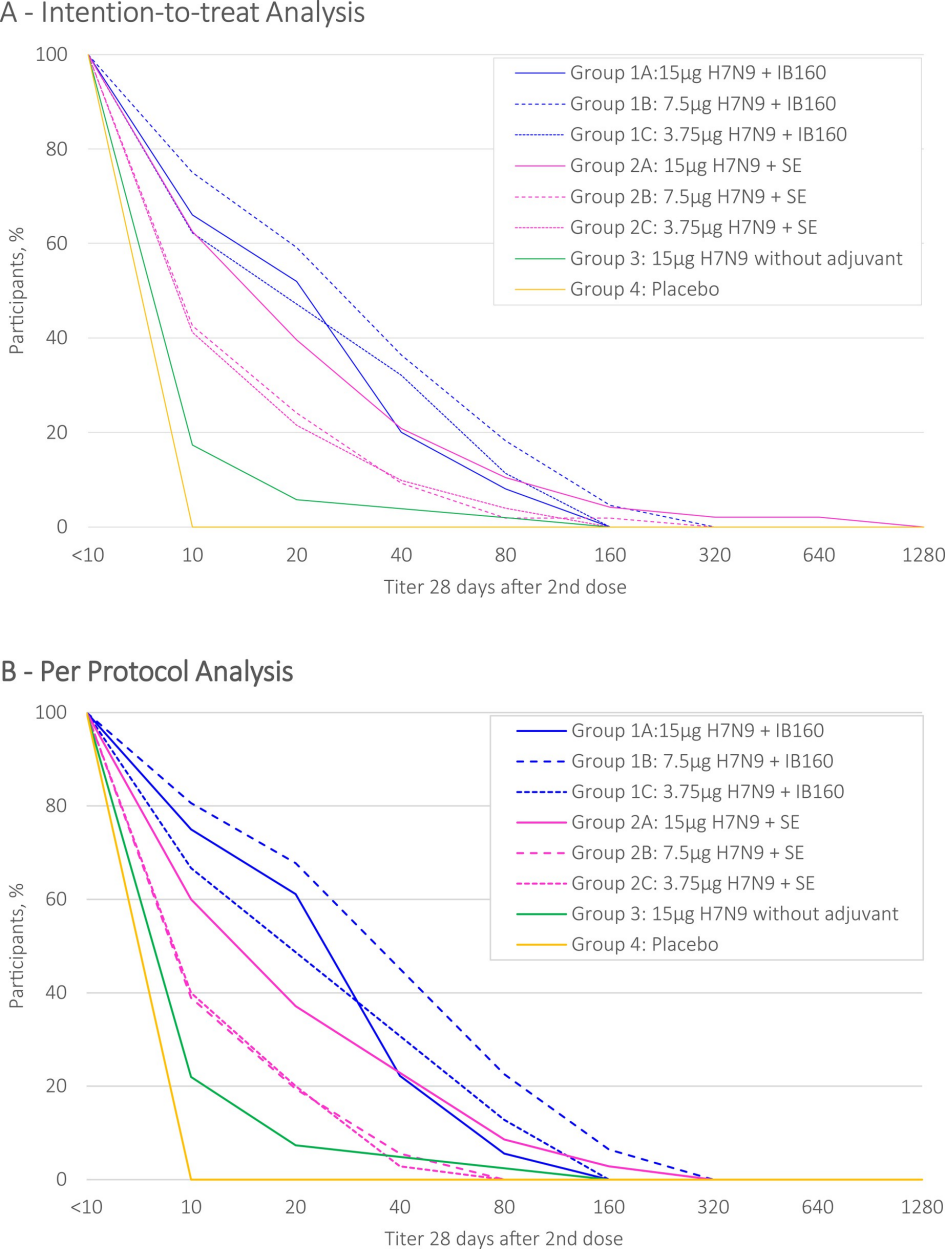
HI antibody against H7N9 reverse cumulative distribution curve after 28(+7) days post 2nd dose administration, intention-to-treat.

**Fig 3:**
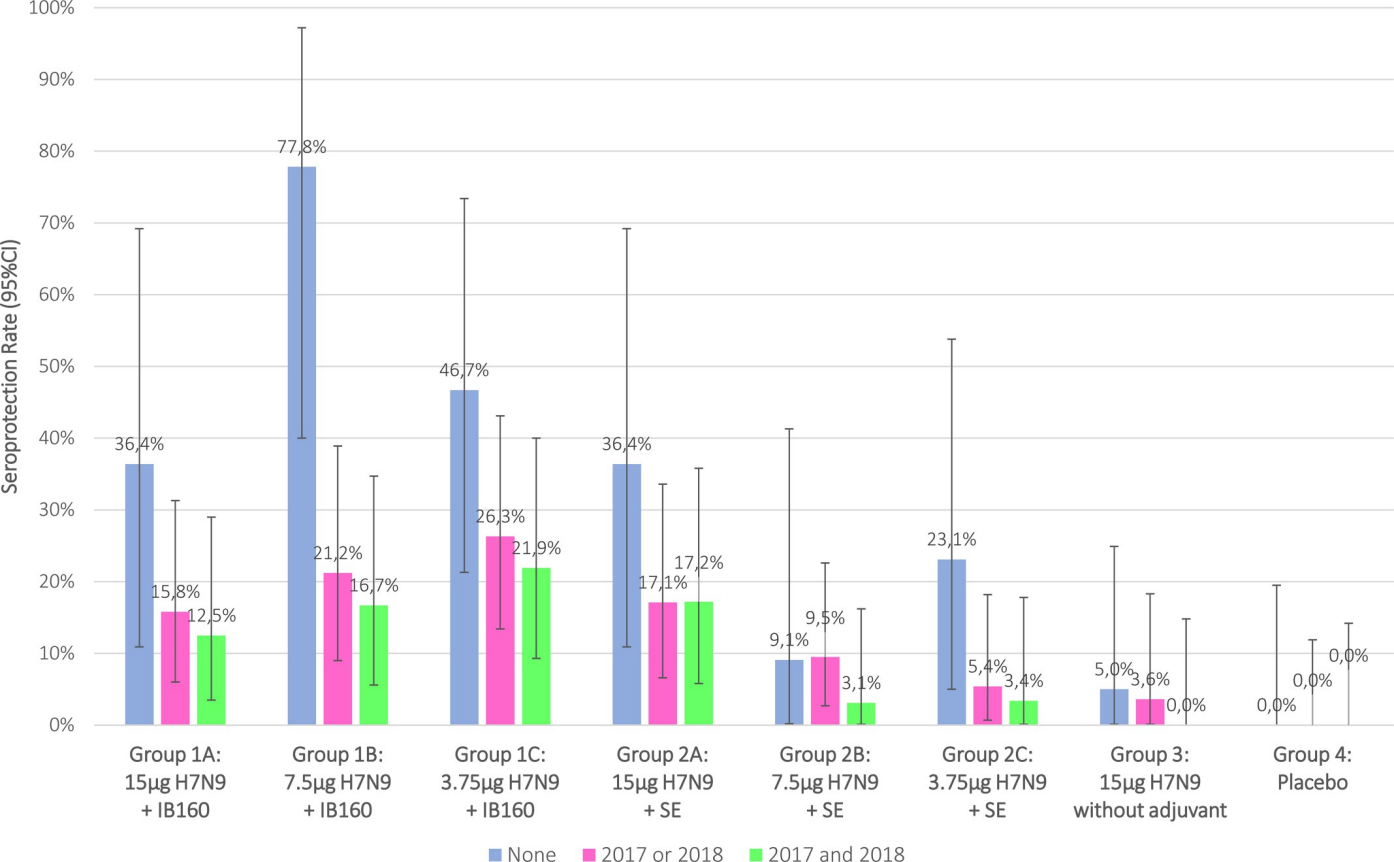
HI antibody against H7N9 reverse cumulative distribution curve after 28(+7) days post 2nd dose administration, per protocol analysis.

**Fig 4:**
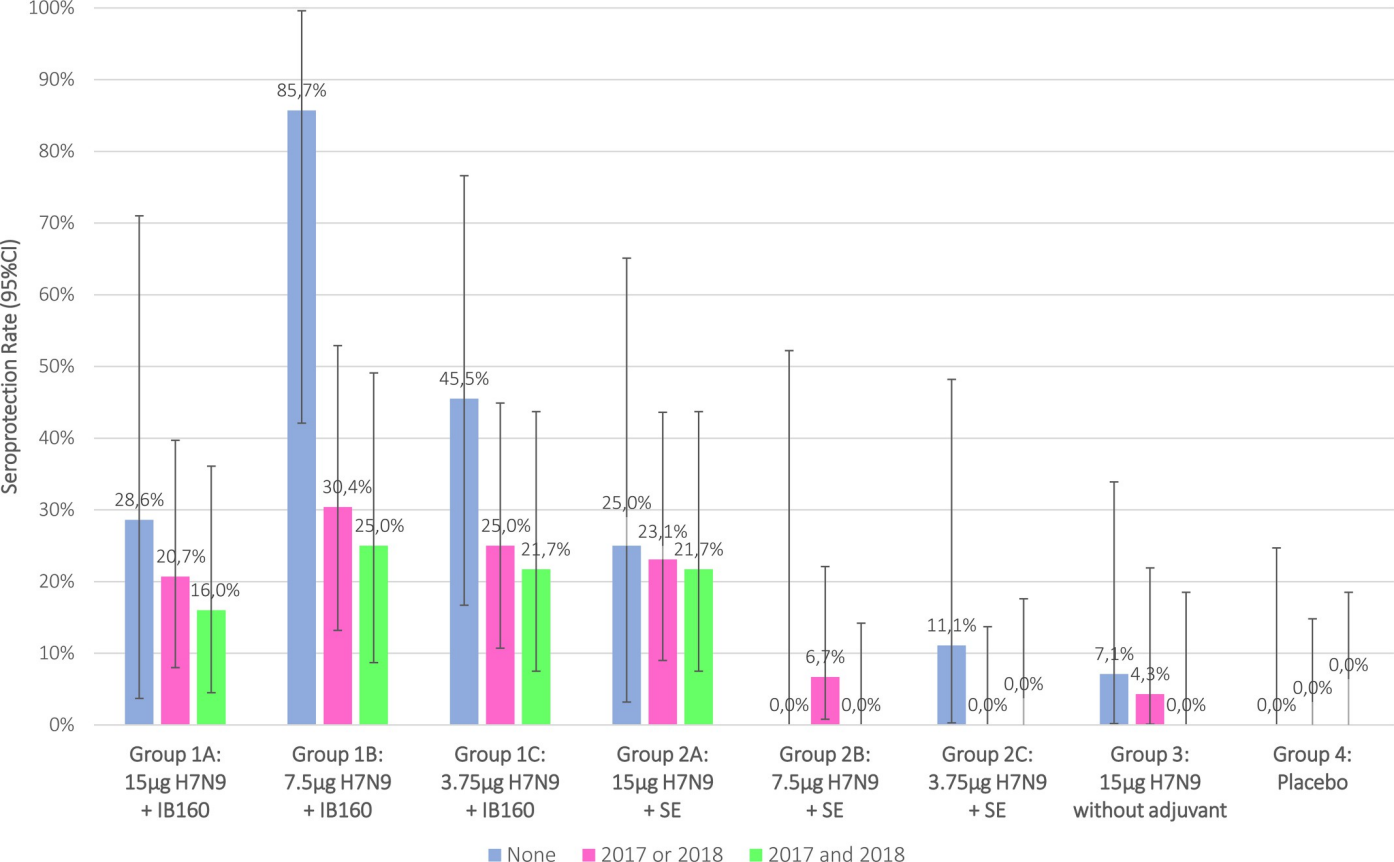
Association of HI seroprotection rate after 28(+7) days post 2nd dose administration with prior receipt of seasonal influenza vaccine, intention-to-treat analysis.

**Fig 5:**
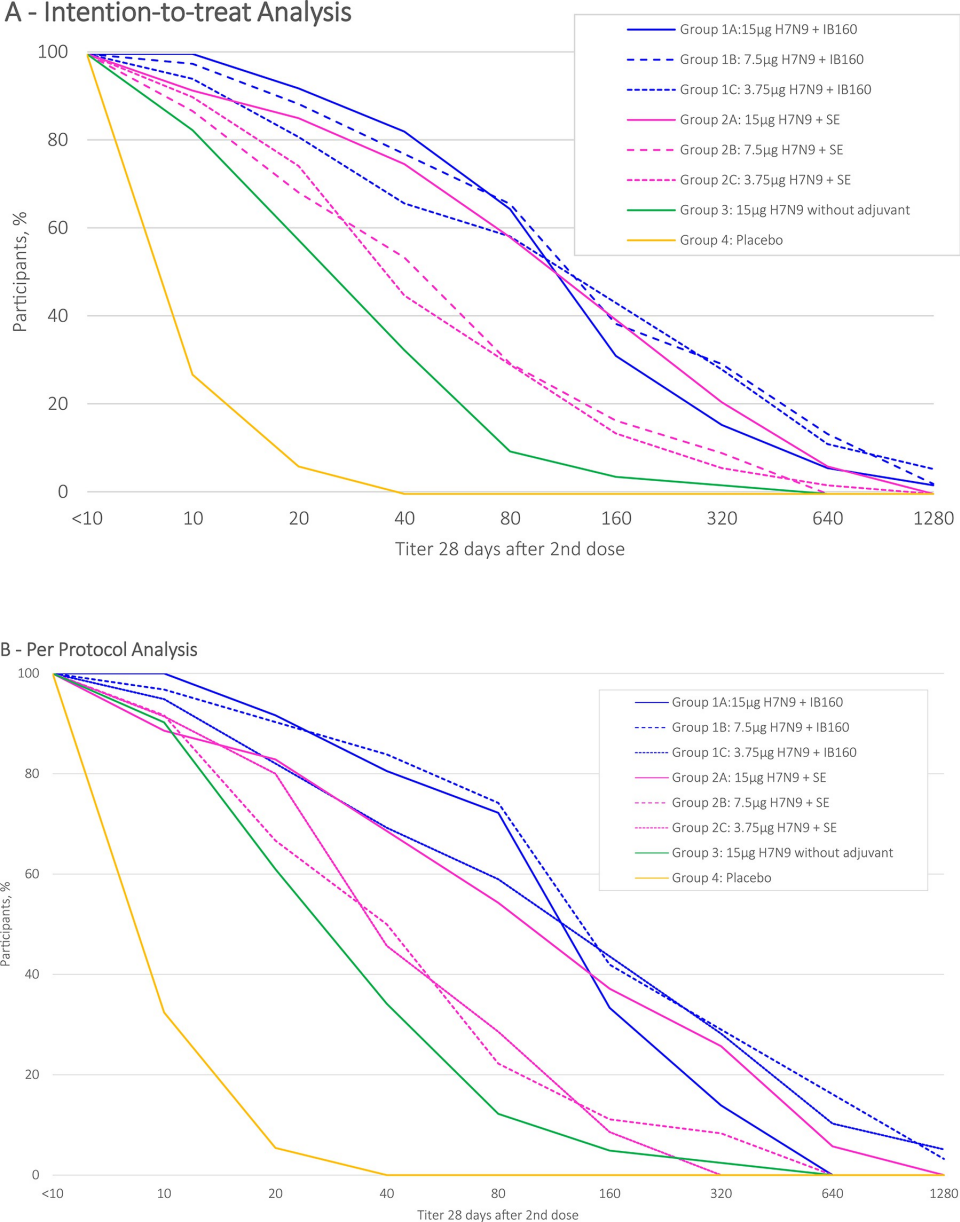
Association of HI seroprotection rate after 28(+7) days post 2nd dose administration with prior receipt of seasonal influenza vaccine, per protocol analysis.

The distribution of HI antibody titers (d56), from each intervention group, are described by reverse cumulative distribution curves for intention-to-treat and per protocol analysis (Figs 2 and 3). Intervention groups 1A, 1B, 1C and 2A presented a stronger effect in a greater proportion of participants. SPR stratified by prior receipt of seasonal influenza vaccine in the intention-to-treat analysis are shown in Fig 4 and in the per protocol analysis in Fig 5. Prior receipt of seasonal influenza vaccine in one of the last two seasons (2017 or 2018), and both last seasons (2017 and 2018) seems to be associated with lower HI SCR when compared to those not receiving seasonal influenza vaccine in the last two seasons. The statistical differences (p<0.05) obtained in HI (GMT), SPR as well as SCR on pairwise comparisons across the groups both for intention-to-treat and per protocol analysis with respective Bonferroni corrections are presented in the S1 File.

#### Microneutralization (MN) results

**Fig 6:**
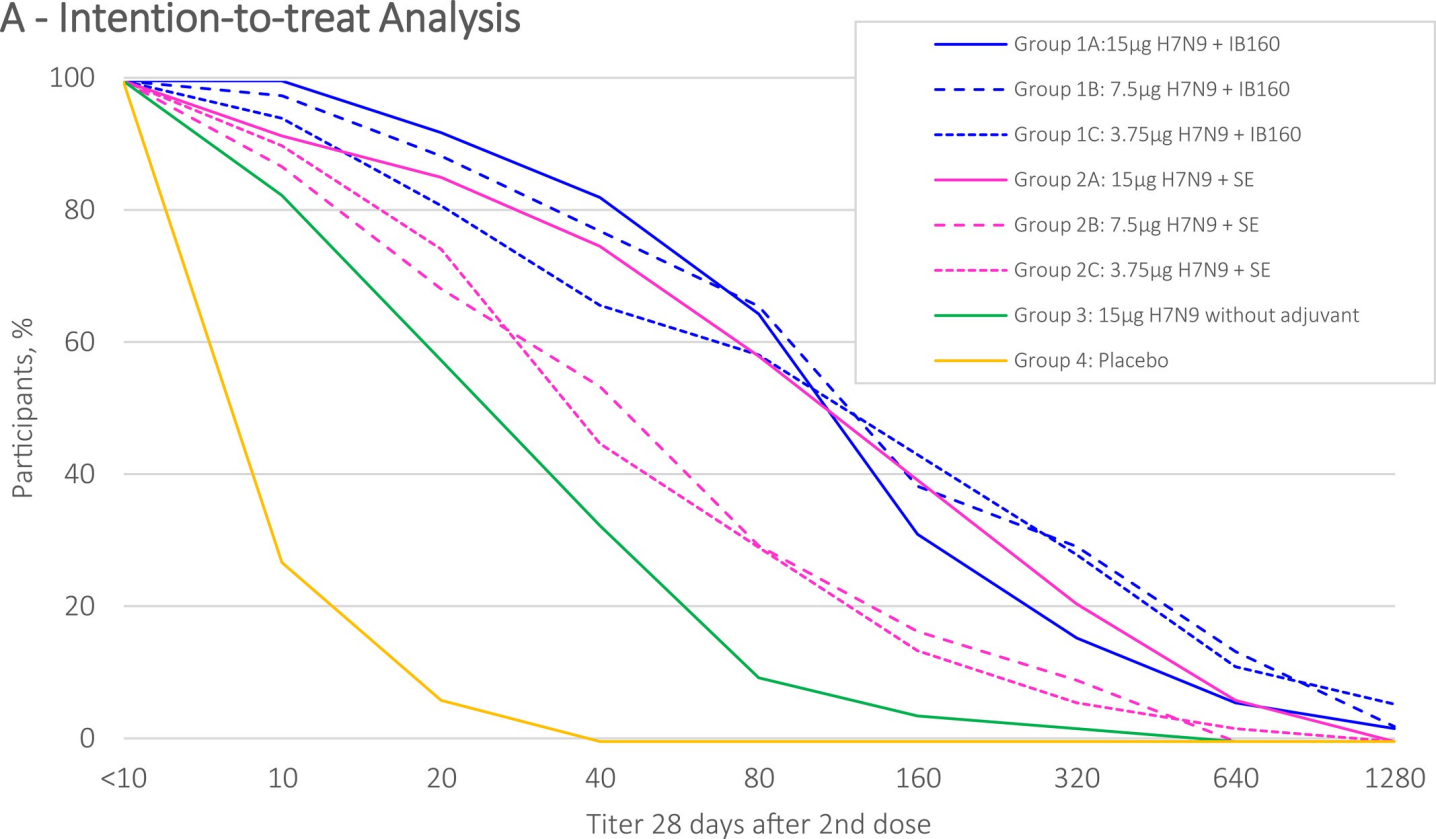
MNT antibody against H7N9 reverse cumulative distribution curve after 28(+7) days post 2nd dose administration, intention-to-treat.

**Fig 7:**
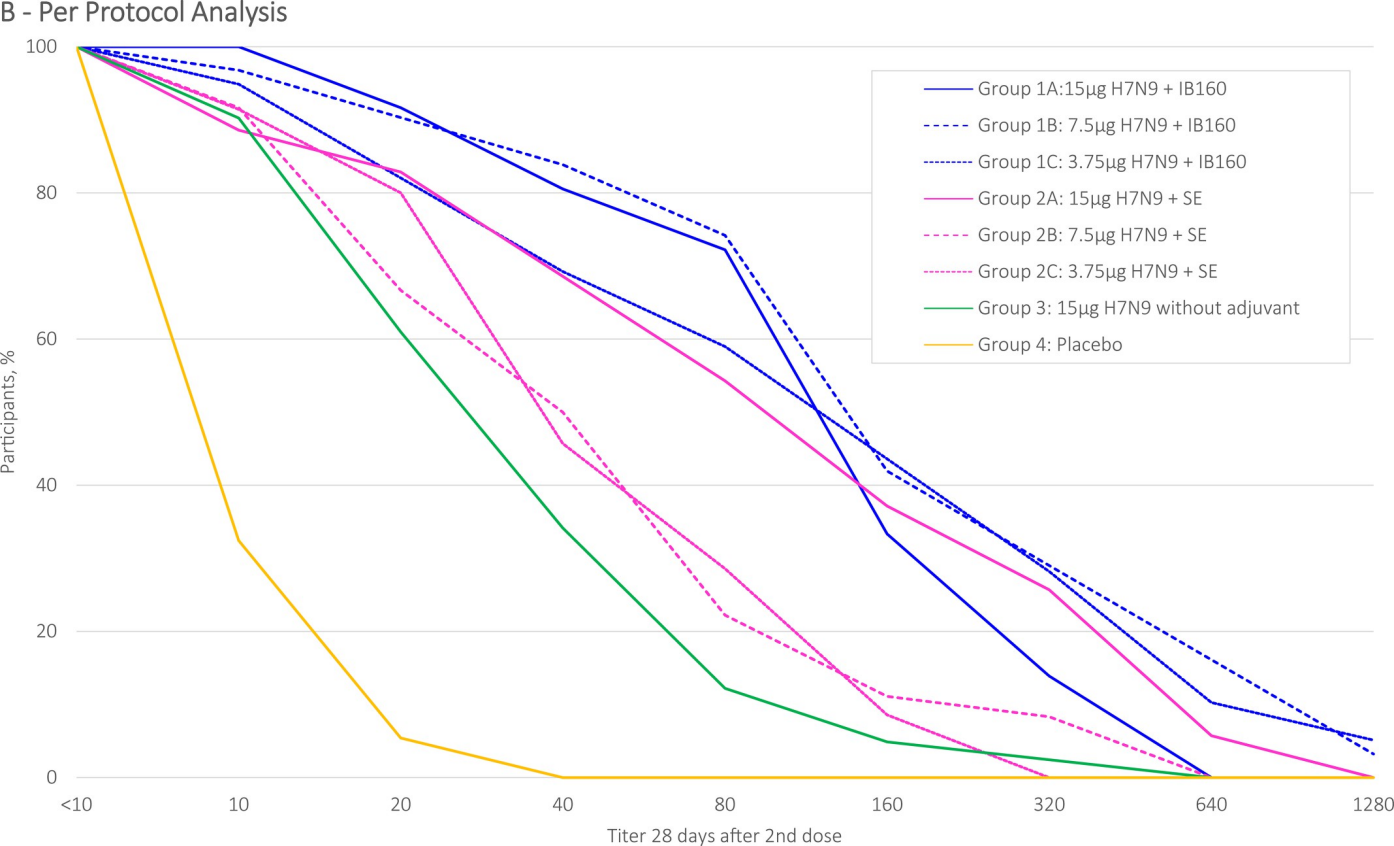
MNT antibody against H7N9 reverse cumulative distribution curve after 28(+7) days post 2nd dose administration, per protocol analysis.

Tables 4 and 5 describe the MN data by study group intention-to-treat and per-protocol analysis, respectively. When comparing the HI and MN results, a greater increase in titers was observed in the latter, particularly after the second dose. As found in HI tests, there was a greater increase in MN GMT among those who received vaccines with adjuvant formulations. The highest GMT ratio was observed in group 1B, for those who received IB160-adjuvanted vaccines, and in group 2A, for those who received SE-adjuvanted vaccines. MN tests showed much higher SPR and SCR rates than HI tests, reaching 80% for groups 1A and 1B. The distribution of MN antibody titers (d56) from each intervention group are described by reverse cumulative distribution curves for intention-to-treat and per protocol analysis (Figs 6 and 7). As showed in HI results, intervention groups 1A, 1B, 1C and 2A presented a stronger effect in a greater proportion of participants. The statistically significant differences (p<0.05) obtained in MN (GMT and GMT ratio), SPR and SCR on pairwise comparisons across the groups in the intention-to-treat and per protocol analysis are presented in the S1 File.

**Table 4:** Microneutralization (MN) data by study group, intention-to-treat analysis.

	**Group 1A**			**Group 1B**			**Group 1C**			**Group 2A**			**Group 2B**			**Group 2C**			**Group 3**			**Group 4**		
	**15μ H7N9 + adj IB160**			**7,5μ H7N9 + adj IB160**			**3,75μ H7N9 + adj IB160**			**15μ H7N9 + adj SE**			**7,5μ H7N9 + adj SE**			**3,75μ H7N9 + adj SE**			**15μ H7N9, without adj**			**Placebo**		
	**n**	**Value**	**(95%CI)**	**n**	**Value**	**(95%CI)**	**n**	**Value**	**(95%CI)**	**n**	**Value**	**(95%CI)**	**n**	**Value**	**(95%CI)**	**n**	**Value**	**(95%CI)**	**n**	**Value**	**(95%CI)**	**n**	**Value**	**(95%CI)**
**GMT**																								
Pre	51	8.6	(7.6–9.7)	47	7.4	(6.4–8.5)	54	9.0	(7.8–10.4)	49	8.1	(7.0–9.4)	54	7.5	(6.6–8.6)	51	8.5	(7.2–10.0)	52	8.3	(7.2–9.6)	49	7.5	(6.6–8.6)
d28	50	15.0	(12.0–18.7)	47	13.2	(10.3–17.0)	53	12.9	(10.0–16.7)	49	11.0	(8.9–13.5)	53	9.7	(8.2–11.4)	51	8.6	(7.1–10.3)	52	8.7	(7.5–10.0)	49	6.1	(5.6–6.7)
d56	50	67.3	(50.4–89.8)	44	78.6	(52.0–118.8)	53	67.8	(44.6–103.2)	48	61.7	(41.8–91.1)	54	28.5	(20.3–39.9)	51	28.1	(20.3–38.9)	52	17.0	(13.2–21.9)	48	5.9	(5.4–6.6)
**GMFR from Pre**																								
d28	50	1.7	(1.4–2.2)	47	1.8	(1.4–2.2)	53	1.4	(1.1–1.8)	49	1.4	(1.1–1.7)	53	1.3	(1.1–1.5)	51	1.0	(0.8–1.2)	52	1.0	(0.9–1.2)	49	0.8	(0.7–0.9)
d56	50	7.8	(5.9–10.4)	44	10.4	(6.8–15.9)	53	7.5	(4.9–11.4)	48	7.8	(5.3–11.5)	54	3.8	(2.7–5.3)	51	3.3	(2.2–4.9)	52	2.1	(1.6–2.6)	48	0.8	(0.7–0.9)
**SPR, %**																								
Pre	51	0.0	(0.0–7.0)	47	0.0	(0.0–7.5)	54	0.0	(0.0–6.6)	49	2.0*	(0.0–10.9)	54	0.0	(0.0–6.6)	51	0.0	(0.0–7.0)	52	0.0	(0.0–6.8)	49	0.0	(0.0–7.3)
d28	50	22.0	(11.5–36.0)	47	17.0	(7.6–30.8)	53	15.1	(6.7–27.6)	49	8.2	(2.3–19.6)	53	3.8	(0.5–13.0)	51	5.9	(1.2–16.2)	52	1.9	(0.0–10.3)	49	0.0	(0.0–7.3)
d56	50	80.0	(66.3–90.0)	44	75.0	(59.7–86.8)	53	66.0	(51.7–78.5)	48	70.8	(55.9–83.0)	54	44.4	(30.9–58.6)	51	41.2	(27.6–55.8)	52	26.9	(15.6–41.0)	48	0.0	(0.0–7.3)
**SCR from Pre, %**																								
d28^‡^	50	18.0	(8.6–31.4)	47	12.8	(4.8–25.7)	53	13.2	(5.5–25.3)	49	8.2	(2.3–19.6)	53	1.9	(0.0–10.1)	51	2.0	(0.0–10.4)	52	0.0	(0.0–6.8)	49	0.0	(0.0–7.3)
d56^‡‡^	50	78.0	(64.0–88.5)	44	75.0	(59.7–86.8)	53	60.4	(46.0–73.5)	48	68.8	(53.7–81.30	54	38.9	(25.9–53.1)	51	33.3	(20.8–47.9)	52	25.0	(14.0–38.9)	48	0.0	(0.0–7.3)

adj: adjuvant; 95%CI: 95% confidence interval; GMT: geometric mean titters; GMFR: geometric mean fold rises, i.e., ratio between GMT of baseline and of post-dose; SPR: seroprotection rate (prior and postvaccination HI antibody titers ≥1:40); SCR: seroconversion rate (baseline HI antibody titers <1:10 and postvaccination HI antibody titers ≥1:40, or baseline HI antibody titers ≥1:10 and a postvaccination increase by a factor of four or more); Sample collected at: Pre (prior 1st dose), d28 (prior 2nd dose, 28 days post 1st dose), d35 (7 days post 2nd dose or 35 days post 1st dose), d56 (28 days post 2nd dose or 56 days post 1st dose); NE: not estimable.

*This percentage represents one single individual which presented the following titers: pre = 40, d28 = 20, d56 = 320.

**Table 5:** Microneutralization (MN) data by study group, per protocol analysis.

	Group 1A			Group 1B			Group 1C			Group 2A			Group 2B			Group 2C			Group 3			Group 4		
	15μ H7N9 + adj IB160			7,5μ H7N9 + adj IB160			3,75μ H7N9 + adj IB160			15μ H7N9 + adj SE			7,5μ H7N9 + adj SE			3,75μ H7N9 + adj SE			15μ H7N9, without adj			Placebo		
	n	Value	(95%CI)	n	Value	(95%CI)	n	Value	(95%CI)	n	Value	(95%CI)	n	Value	(95%CI)	n	Value	(95%CI)	n	Value	(95%CI)	n	Value	(95%CI)
**GMT**																								
Pre	36	8.8	(7.7–10.2)	31	7.5	(6.2–9.0)	39	9.2	(7.7–11.1)	35	7.6	(6.3–9.1)	36	7.9	(6.7–9.4)	35	8.0	(6.5–9.7)	41	8.6	(7.2–10.2)	37	7.4	(6.3–8.7)
d28*	36	17.2	(13.2–22.2)	31	14.0	(10.2–19.1)	39	13.1	(9.6–17.8)	35	10.8	(8.2–14.3)	36	9.7	(7.9–12.0)	35	8.2	(6.9–9.8)	41	9.0	(7.6–10.7)	37	6.1	(5.5–6.9)
d56**	36	70.6	(50.48–99.0)	31	95.7	(59.9–152.7)	39	72.4	(45.3–115.8)	35	57.1	(34.9–93.4)	36	25.7	(17.5–37.7)	35	27.2	(19.6–37.8)	41	18.9	(14.3–24.9)	37	6.1	(5.4–6.8)
**GMFR from Pre**																								
d28^†^	36	1.9	(1.5–2.5)	31	1.9	(1.4–2.5)	39	1.4	(1.1–1.9)	35	1.4	(1.1–1.8)	36	1.2	(1.0–1.5)	35	1.0	(0.8–1.3)	41	1.1	(0.9–1.2)	37	0.8	(0.8–0.9)
d56^††^	36	8.0	(5.7–11.2)	31	12.8	(7.9–20.8)	39	7.8	(4.9–12.6)	35	7.5	(4.7–12.1)	36	3.2	(2.2–4.7)	35	3.4	(2.2–5.3)	41	2.2	(1.6–2.9)	37	0.8	(0.7–0.9)
**SPR, %**																								
Pre	36	0.0	(0.0–9.7)	31	0.0	(0.0–11.2)	39	0.0	(0.0–9.0)	35	2.9*	(0.0–14.9)	36	0.0	(0.0–9.7)	35	0.0	(0.0–10.0)	41	0.0	(0.0–8.6)	37	0.0	(0.0–9.5)
d28^#^	36	25.0	(12.1–42.2)	31	16.1	(5.5–33.7)	39	15.4	(5.9–30.5)	35	11.4	(3.2–26.7)	36	2.8	(0.0–14.5)	35	0.0	(0.0–10.0)	41	2.4	(0.0–12.9)	37	0.0	(0.0–9.5)
d56^##^	36	77.8	(60.8–89.9)	31	80.6	62.5–92.5)	39	69.2	(52.4–83.0)	35	68.6	(50.7–83.1)	36	41.7	(25.5–59.2)	35	42.9	(26.3–60.6)	41	26.8	(14.2–42.9)	37	0.0	(0.0–9.5)
**SCR from Pre, %**																								
d28^‡^	36	19.4	(8.2–36.0)	31	12.9	(3.6–29.8)	39	12.8	(4.3–27.4)	35	11.4	(3.2–26.7)	36	0.0	(0.0–9.7)	35	0.0	(0.0–10.0)	41	0.0	(0.0–8.6)	37	0.0	(0.0–9.5)
d56^‡‡^	36	77.8	(60.8–89.9)	31	80.8	(62.5–92.5)	39	64.1	(47.2–78.8)	35	65.7	(47.8–80.9)	36	33.3	(18.6–51.0)	35	37.1	(21.5–55.1)	41	24.4	(12.4–40.3)	37	0.0	(0.0–9.5)

adj: adjuvant; 95%CI: 95% confidence interval; GMT: geometric mean titers; GMFR: geometric mean fold rises, i.e., ratio between GMT of baseline and of post-dose; SPR: seroprotection rate (prior and postvaccination HI antibody titers ≥1:40); SCR: seroconversion rate (baseline HI antibody titers <1:10 and postvaccination HI antibody titers ≥1:40, or baseline HI antibody titers ≥1:10 and a postvaccination increase by a factor of four or more); Sample collected at: Pre (prior 1st dose), d28 (prior 2nd dose, 28 days post 1st dose), d35 (7 days post 2nd dose or 35 days post 1st dose), d56 (28 days post 2nd dose or 56 days post 1st dose); NE: not estimable.

*This percentage represents one single individual which presented the following titters: pre = 40, d28 = 20, d56 = 320.

### Safety evaluation

As of January 2020, three SAEs occurred (rhabdomyolysis, aspartate aminotransferase increase and migraine). Rhabdomyolysis and aspartate aminotransferase increase occurred in the same participant. According to the DSMB evaluation, it was determined that the relationship with the intervention (group 1A) was possible and probable, respectively. The participant recovered and did not receive the second dose of the vaccine. The migraine was determined to have unlikely relationship with the intervention (group 2B). One adverse event of special interest was reported (thyroiditis). The thyroiditis occurred in the intervention group 4 (placebo) and presented resolution. One pregnancy was reported during the study (group 2A). No abnormalities were detected during the pregnancy follow up.

The median number of AEs and ARs per participant varied from 1 to 3 (Table 6). The frequencies of AEs and ARs were similar among the different intervention groups and little variation occurred between V1 and V2. Pain at the injection site was the most common local solicited AR and headache was the most common systemic solicited AR occurring within 7 days after V1 and V2, as described in the supplement tables. Most solicited AEs started on the day of vaccination and lasted less than a day. Headache and myalgia were the solicited AR that most often required medication. The unsolicited ARs were reported by very few participants (range 0 to 2) and were evenly observed among the eight intervention groups within 28 days post each study injection (supplement tables).

**Table 6:** Demographic characteristics, prior seasonal influenza vaccine, and study site, by study group.

	Post 1st dose administration				Post 2nd dose administration			
**STUDY GROUP**	**AEs**	**Participants**	**AE per participant**		**AEs**	**Participants**	**AE per participant**	
	**n** ^**o**^	**with AE (n** ^**o**^ **)**	**Median**	**(P**_**25**_ **-P**_**75**_ **)**	**n** ^**o**^	**with AE (n** ^**o**^ **)**	**Median**	**(P**_**25**_ **-P**_**75**_ **)**
Group 1A: 15μg H7N9 + IB160	135	47	2	(1–4)	83	39	2	(1–3)
Group 1B: 7.5μg H7N9 + IB160	134	43	2	(1–4)	105	39	2	(2–4)
Group 1C: 3.75μg H7N9 + IB160	111	46	2	(1–3)	113	37	3	(1–4)
Group 2A: 15μg H7N9 + SE	94	37	2	(1–4)	58	28	2	(1–3)
Group 2B: 7.5μg H7N9 + SE	108	46	2	(1–3)	91	33	2	(1–3)
Group 2C: 3.75μg H7N9 + SE	95	37	2	(1–3)	91	36	2	(1–3)
Group 3: 15μg H7N9 without adjuvant	86	33	2	(1–4)	59	26	2	(1–3)
Group 4: Placebo	88	37	2	(1–3)	39	21	2	(1–2)
**TOTAL**	851	326	2	(1–3)	639	259	2	(1–3)
**STUDY GROUP**	**ARs**	**Participants**	**AR per participant**		**ARs**	**Participants**	**AR per participant**	
	**n** ^**o**^	**with AR (n** ^**o**^ **)**	**Median**	**(P**_**25**_ **-P**_**75**_ **)**	**n** ^**o**^	**with AR (n** ^**o**^ **)**	**Median**	**(P**_**25**_ **-P**_**75**_ **)**
Group 1A: 15μg H7N9 + IB160	120	47	2	(1–3)	71	38	1	(1–2)
Group 1B: 7.5μg H7N9 + IB160	122	41	2	(1–4)	86	37	2	(1–3)
Group 1C: 3.75μg H7N9 + IB160	95	45	2	(1–3)	95	36	2	(1–3.5)
Group 2A: 15μg H7N9 + SE	82	35	2	(1–3)	49	25	2	(1–3)
Group 2B: 7.5μg H7N9 + SE	94	43	1	(1–2)	76	28	2	(1–3)
Group 2C: 3.75μg H7N9 + SE	81	33	2	(1–3)	70	32	1.5	(1–2.5)
Group 3: 15μg H7N9 without adjuvant	72	31	2	(1–3)	43	23	2	(1–2)
Group 4: Placebo	68	33	2	(1–3)	30	15	2	(1–3)
**TOTAL**	734	308	2	(1–3)	520	234	2	(1–3)

adj: adjuvant; P_25_: first quartile; P_75_: third quartile

## Discussion

In this study, we observed that the immune responses after the first dose were low in all study groups. After two doses of the candidate vaccines, there was a greater increase in antibody titers among those who received vaccines with adjuvant formulations. Among those who received IB160-adjuvanted vaccines, the greatest response was seen after 2 doses of 7.5 μg antigen, which induced an HI titer of 40 or higher in at least 45.2% of participants (80.8% for neutralizing antibodies). Among those who received SE-adjuvanted vaccines, the greatest response was seen after 2 doses of 15 μg antigen, which induced an HI titer of 40 or higher in at least 22.9% of participants (65.7% for neutralizing antibodies). However, none of the studied interventions met the criteria suggested by the US Food and Drug Administration for accelerated approval of pandemic vaccines, which are based on SCR and SPR according to HI titers [21].

In comparison with three studies of H7N9 adjuvanted-vaccines [5–7], we observed lower SCR and SPR in respect to HI titers, but similar SCR and SPR estimates in respect to MN titers. It is important to point out that these studies used different vaccination schedule (2 doses 21 days apart, instead of 28 days apart) and a different immunogenicity evaluation schedule (42 days post first dose, instead of 56 days post first dose). Additionally, the age of participants and the percentage of those with no previous seasonal influenza vaccination in the last 2 years is quite different among studies.

As in previous studies, recent receipt of seasonal influenza vaccine seems to be associated with diminished antibody responses, suggesting interference from pre-existing immunity [5, 6]. The mechanisms and clinical relevance of seasonal vaccination interference with H7N9 vaccine response are unclear and deserve further investigation. Original antigenic sin was described in the 1950s as an exposure to a new influenza strain with resulting preferential induction of antibodies to a previously encountered related strain and diminished response to the new strain [5, 22, 23]. Cross-reactive antibody binding to the conserved HA2 (stem), cellular responses, or both might play a role. The detection of low levels of neutralizing antibody at baseline, and their more rapid increase relative to HI antibodies after vaccination, are likely due to pre-existing long-lived plasma cells and memory B cells, respectively, that recognize HA2 [5].

This study has limitations. The study did not assess antibody longevity beyond day 56. Vaccine immunological responses up to 385 days post first study injection, showed a peak around 42 days and a subsequent decline in titers [7]. It would also be important to evaluate the potency of the adjuvant formulations, since another study observed that the potency of the adjuvant formulations (11.86 or 5.93 mg tocopherol, 10.69 or 5.34 mg squalene, 4.86 or 2.43 mg polysorbate 80 in AS03A or AS03B, respectively) seems to have more influence than antigen content on immunogenicity [7, 24]. The assessment of immunogenicity for this study is based on HI and MN assays; no cell-mediated immunity was performed which could bring additional information but for which no regulatory acceptance criteria have been established [25–27].

Further analysis may consider other assays, such as single radial hemolysis (SRH) [28, 29] and enzyme-linked lectin assay (ELLA) [30, 31], in order to obtain further information about the immune response elicited by the vaccine candidates and to determine whether recipients of the study vaccine develop antibodies that cross react with other H7N9 viruses. Additional HI and MNT analysis could also be conducted using other lineages of H7N9 to assess needs for heterologous boosting [32, 33].

Tolerability seemed to be acceptable, as most participants returned for the second vaccine dose. Most AEs were low grade and resolved spontaneously. None of the three SAEs reported could be related to vaccination with certainty, and none of them resulted in death or permanent impairment. Most of the solicited AR reported by participants lasted less than a day. Pain at the vaccine administration site was the most frequent local solicited AR, and headache was the most frequent solicited systemic AR. Unsolicited Ars were reported by very few participants (range 0 to 2) and were evenly observed in the 8 intervention groups.

Vaccine adjuvants enhance the magnitude and duration of the immune response to antigens. Alum was the first adjuvant included in many vacccines as a safe and effective adjuvant administered in billions doses of vaccines to diverse populations throughout the world over the past 80–90 years. However, in the recent years the licensure of several human vaccines containing novel adjuvants, such as the squalene emulsion-based adjuvants MF59 and AS03 gave new perspectives in vaccine developments. In this regard, the squalene-based oil-in-water emulsion vaccine adjuvant MF59 and AS03 have been administered to more than 200 million people in more than 30 countries, in both seasonal and pandemic influenza vaccines with a safe and efficacious profile since 1997 [34]. IB160 is also a squalene emulsion-based adjuvant with minor differences to MF59 [11]. IB160 was developed at Butantan Institute through a Technology Transfer agreement with Infectious Diseases Research Institute (Seattle, USA) with the support of BARDA and WHO for preparedness of a pandemic influenza response. SE is a stable oil-in water emulsion, where the oil concentration is 2% (v/v), composed of the excipients squalene (oil), glycerol, egg phosphatidylcholine, surfactant (poloxamer) and ammonium phosphate buffer [12]. In this sense, IB160 or SE was combined to a pandemic influenza A/H7N9 antigen [9], which is a poor immunogen, in order to evaluate the safety and efficacy of these vaccines in a phase 1 clinical trial. In summary, our results showed that IB160 provided the greatest response after 2 doses, with a much greater response when compared with the non-adjuvanted H7N9 vaccine, showing the necessity of an adjuvant like IB160 for a better immune response. Though SE also provided a better immune response than non-adjuvanted vaccine, it was inferior to IB160 and the differences may rely on differences in the composition of these adjuvants [11, 12]. In addition, pandemic preparedness strategies include development of vaccines that are antigen sparing, which could help provide effective vaccine coverage in short time. The results of this study showed the adjuvanted-vaccine candidates have a potential dose-sparing effect on immune response of healthy adults against influenza A/H7N9. The magnitude of this effect could be further explored using different immunological assays in order to inform future studies of the evaluated products.

## Supporting information

S1 Appendix (DOCX)

S1 File (DOCX)

## Abbreviations

WHO: World Health Organization
IB: Instituto Butantan
IDRI: Infectious Disease Research Institute
CRF: Case Report Form
CONEP: Brazilian Research National Ethics Council
CDC: Centers for Disease Control and Prevention
AE: Adverse Event; SAE, Serious Adverse Events
AESI: Adverse Event of Special Interest
AR: Adverse Reaction
USFDA: US Food and Drug Administration
UMC: Uppsala Monitoring Centre
