# Supplementary material for: Dose-sparing effect of two adjuvant formulations with a pandemic influenza A/H7N9 vaccine: A randomized, double-blind, placebo-controlled, phase 1 clinical trial
Source: PLoS One. 2022 Oct 18;17(10):e0274943. doi: 10.1371/journal.pone.0274943 (PMC9578608; doi:10.1371/journal.pone.0274943)
Supplement: S1 Appendix — (DOCX) [file pone.0274943.s001.docx]

**SUPPORTING INFORMATION**

**Dose sparing effect of two adjuvant formulations with a pandemic influenza A/H7N9 vaccine: a randomized, double-blind, placebo-controlled, phase 1 clinical trial**

**List title:** Dose sparing effect of two adjuvant formulations with a pandemic influenza A/H7N9 vaccine

**Authors:** Tazio Vanni^1^, Beatriz C Thomé^2^, Erin Sparrow^3^, Martin Friede^3^, Christopher B. Fox^4^, Anna Marie Beckmann^4^, Chuong Huynh^5^, Gabriella Mondini^6^, Daniela H Silveira^7^, Juliana Y K Viscondi^7^, Patrícia Emilia Braga^7^, Anderson da Silva^8^, Maria da Graça Salomão^7^, Roberta O Piorelli^8^, Joane P Santos^8^, Vera Lúcia Gattás^7^, Maria Beatriz Bastos Lucchesi^7^, Mayra Martho Moura de Oliveira^7^, Marcelo Eiji Koike^7^, Esper G Kallas^9^, Lucia M A Campos^10^, Eduardo B Coelho^11^, Marilda A M Siqueira^12^, Cristiana C Garcia^12^, Milene Dias Miranda^12^, Terezinha M Paiva^13^, Maria do Carmo S T Timenetsky^13^, Eduardo Alfredo Adami^8^, Milena Apetito Akamatsu^7^, Paulo Lee Ho^7^, Alexander R Precioso^7,10^.

**Authors’ affiliation**

1.Instituto Butantan – Former employee. Núcleo de Medicina Tropical, Faculdade de Medicina da Universidade de Brasília e Hospital de Base do Distrito Federal, Brazil.

2. Instituto Butantan - Former employee. Federal University of São Paulo, São Paulo, Brazil

3. World Health Organization, Geneva, Switzerland.

4. Infectious Disease Research Institute, Seattle, USA.

5. Biomedical Advanced Research and Development Authority, Washington DC, USA.

6. Instituto Butantan – Former employee. Oxford University, Oxford, UK.

7. Instituto Butantan, São Paulo, Brazil.

8. Instituto Butantan – Former employee.

9. Clinics Hospital of the School of Medicine of University of São Paulo, Brazil.

10. Child Institute of the Clinics Hospital of the School of Medicine of University of São Paulo, São Paulo, Brazil

11. Clinics Hospital of the Medical School of Ribeirão Preto of the University of São Paulo, Ribeirão Preto, Brazil.

12. Oswaldo Cruz Foundation, Rio de Janeiro, Brazil.

13. Adolfo Lutz Institute, São Paulo, Brazil.

.

**Corresponding author:**

1. Alexander Roberto Precioso, M.D., PhD – Pharmacovigilance, Clinical Safety and Risk Management Center - Instituto Butantan. Av. da Universidade 210. São Paulo, SP, ZIP code: 05508-040, Brazil. Phone: (+55-11) 3723-2150. E-mail: [alexander.precioso@butantan.gov.br](mailto:alexander.precioso@butantan.gov.br)

**TABLE OF CONTENTS**

[**Appendix A – Eligibility criteria 17**](#_Toc60588149)

[1.1.1 Inclusion criteria 17](#_Toc60588150)

[1.1.2 Exclusion criteria 17](#_Toc60588151)

[**Appendix B 20**](#_Toc60588152)

[I - Severity classification 20](#_Toc60588153)

[II - Causal relationship classification 24](#_Toc60588154)

[III – List of adverse events of special interest 27](#_Toc60588155)

[**Appendix C – Tables and Charts 30**](#_Toc60588156)

[S. Table 1. Proportion of participants with solicited AR occurring within 30 minutes after the first vaccination, by study group. 31](#_Toc60588157)

[S. Table 2. Proportion of participants with solicited AR occurring within 30 minutes after the second vaccination, by study group. 33](#_Toc60588158)

[S. Table 3. Proportion of participants with solicited AR occurring 7 days after the first vaccination, by study group. 35](#_Toc60588159)

[S. Table 4. Proportion of participants with solicited AR occurring 7 days after the second vaccination, by study group. 37](#_Toc60588160)

[S. Table 5. Proportion of participants with solicited AR occurring 28 days after the first vaccination, by study group. 39](#_Toc60588161)

[S. Table 6. Proportion of participants with solicited AR occurring 28 days after the second vaccination, by study group. 41](#_Toc60588162)

[S. Table 7. Distribution of solicited AR, and medication use, occurring up to 28 days after the first vaccination, by study group. 43](#_Toc60588163)

[S. Table 8. Distribution of solicited AR, and medication use, occurring up to 28 days after the second vaccination, by study group. 45](#_Toc60588164)

[S. Table 9. Distribution and duration of solicited AR occurring up to 28 days after the first vaccination, by study group. 47](#_Toc60588165)

[S. Table 10. Distribution and duration of solicited AR occurring up to 28 days after the second vaccination, by study group. 49](#_Toc60588166)

[S. Table 11. Distribution and start day of solicited AR occurring up to 28 after the first vaccination, by study group. 51](#_Toc60588167)

[S. Table 12. Distribution and start day of solicited AR occurring up to 28 days post second dose administration by study group. 53](#_Toc60588168)

[S. Table 13. Proportion of participants with unsolicited adverse reactions occurring within 30 minutes after the first vaccination, by study group. 55](#_Toc60588169)

[S. Table 14. Proportion of participants with unsolicited adverse reactions occurring within 30 minutes after the second vaccination, by study group. 57](#_Toc60588170)

[S. Table 15. Proportion of participants with unsolicited adverse reactions occurring 7 days after the first vaccination, by study group. 59](#_Toc60588171)

[S. Table 15. Proportion of participants with unsolicited adverse reactions occurring 7 days after the first vaccination, by study group (continuation). 61](#_Toc60588172)

[S. Table 15. Proportion of participants with unsolicited adverse reactions occurring 7 days after the first vaccination, by study group (continuation). 63](#_Toc60588173)

[S. Table 16. Proportion of participants with unsolicited adverse reactions occurring 7 days after the second vaccination, by study group. 65](#_Toc60588174)

[S. Table 16. Proportion of participants with unsolicited adverse reactions occurring 7 days after the second vaccination, by study group (continuation). 67](#_Toc60588175)

[S. Table 17. Proportion of participants with unsolicited adverse reactions occurring 28 days after the first vaccination, by study group. 69](#_Toc60588176)

[S. Table 18. Proportion of participants with unsolicited adverse reactions occurring 28 days after the second vaccination, by study group. 75](#_Toc60588177)

[S. Table 19. Hemagglutination inhibition (HI) data by study group, intention-to-treat analysis. 80](#_Toc60588178)

[S. Table 20. Immunogenicity of serum antibody to Influenza H7N9 virus detected by microneutralization assay by study group, intention-to-treat analysis. 81](#_Toc60588179)

[S. Chart 1. Hemagglutination inhibition (GMT and SPR) pairwise comparisons for intention-to-treat analysis. 84](#_Toc60588180)

[S. Chart 2. Hemagglutination inhibition (GMT and SPR) pairwise comparisons for per-protocol analysis. 86](#_Toc60588181)

[S. Chart 3. Microneutralization (MN) results (GMT, GMFR, SPR and SCR) pairwise comparisons for intention-to-treat analysis. 88](#_Toc60588182)

[S. Chart 4. Microneutralization (MN) results (GMT, GMFR, SPR and SCR) pairwise comparisons for per-protocol analysis. 90](#_Toc60588183)

[References **91**](#_Toc60588184)

**Appendix A – Eligibility criteria**

To be included in the study participants had to meet all the inclusion criteria and could not fulfill any of the exclusion criteria.

**1.1.1 Inclusion criteria**

The following criteria had to be met before a participant could be enrolled for participation:

1. Healthy male or female (non-pregnant) adults 18 through 59 years of age at the enrolment visit;
2. Available to participate in the study throughout its duration (approximately seven months);
3. Healthy, as established by the medical history, physical examination, and screening laboratory evaluations;
4. Capable and willing to complete Participant Diaries;
5. Willing to participate in the study, as documented by signature in the study´s informed consent form;
6. For females of child-bearing potential, willing to utilize reliable birth control measures from V1 through at least 60 days following the last study vaccination.

**1.1.2 Exclusion criteria**

Participants meeting any of the following criteria will be excluded from participation:

1. Participation in another clinical trial involving any experimental therapy within the previous three months or planned enrolment in such a trial during the period of this study.
2. Evidence of active neurological, cardiac, pulmonary, hepatic or renal disease as clinical history and/or physical examination (except hypertension under control)
3. Compromised infection/immune system diseases including: HIV, Hepattis B and C, diabetes mellitus, cancer (except basal cell carcinoma) and autoimmune diseases.
4. Behavioral, cognitive or psychiatric disease that in the opinion of the principal investigator or his representative physician, affects the participant ability to understand and cooperate with all study protocol requirements.
5. Abusive usage of alcohol or drugs in the past 12 months that has caused medical, professional or family problems, indicated by clinical history.
6. Known systemic hypersensitivity to eggs or to any component of the vaccine.
7. History of severe adverse reaction after previous administration of an Influenza vaccine within 6 weeks following vaccination.
8. History of Guillain-Barre Syndrome or other demyelinating disease.
9. History of severe reactions following previous immunization with licensed or unlicensed influenza virus vaccines.
10. Diagnosis of asthma with a history of hospitalization related to this condition in the last six months due to illness.
11. Suspected or confirmed fever in the 3 days prior to vaccination or axillary temperature greater than 37.8 ° C on the day of vaccination.
12. Use of corticosteroids (except topical or nasal) or other immunosuppressive drugs within 42 days before study initiation/baseline. It will be considered immunosuppressive dose of corticosteroids the equivalent to a dose ≥10 mg of prednisone per day for over 14 days.
13. Impaired coagulation due to chronic disease or due to use anticoagulant medication (warfarin or heparin) in the 7 days preceding vaccination.
14. Have received live virus vaccine within 28 days or killed virus vaccine in the last 14 days prior to vaccination or have a scheduled immunization from the first study vaccination until 28 days after the second vaccination.
15. Have received any influenza A/H7 vaccine.
16. History of asplenia.
17. Have received blood products in the past 6 months, including transfusions or immunoglobulin, or scheduled administration of blood products or immunoglobulin for the first 28 days after vaccination
18. Any other condition that might put at risk the safety/rights of a potential participant or his/her compliance with this protocol in investigator’s opinion or his representative physician
19. Laboratory values at screening equal to or greater than Grade 2 will be considered to be exclusionary. Vital signs may be performed up to three times to allow for transient conditions to resolve. Screening laboratory values that are out of range but are considered to be due to an acute illness or process may be repeated once. Grade 1 laboratory values will be reviewed by a licensed study clinician and the clinician will determine whether the laboratory abnormality is clinically significant and should be considered exclusionary. If determined to be clinically insignificant, the study team is not required to follow the laboratory until resolution, or the value is determined to be clinically stable.

**Appendix B**

**I - Severity classification**

Based on the guide “Toxicity Grading Scale for Healthy Adult and Adolescent Volunteers Enrolled in Preventive Vaccine Clinical Trials” from the US Food and Drug Administration (USFDA).^1,2,3^

**Classification of the severity of the solicited clinical adverse events.**

| **Solicited Local Adverse Events** | | | | |
| --- | --- | --- | --- | --- |
| **Adverse Event** | **Grade 1** | **Grade 2** | **Grade 3** | **Grade 4** |
| Pain/tenderness at the injection site of the investigational product | Does not interfere  with daily activities | Repeated use of non-narcotic analgesic >24 hours  OR  Mild interference with daily activities | Any use of a narcotic analgesic  OR  Prevent daily activities | Emergency department visit*  OR  Hospitalization |
| Erythema at the injection site of the investigational product ^†^ | 25 – 50 mm | 51 – 100 mm | >100 mm | Necrosis  OR  Exfoliative dermatitis |
| Swelling/Induration at the injection site of the investigational product ^†^ | 25 – 50 mm | 51 – 100 mm  OR  Mild interference with daily activities | >100 mm | Necrosis |
| Ecchymosis at the injection site of the investigational product ^†^ | 25 – 50 mm | 51 – 100 mm | >100 mm | Necrosis |
| Pruritus at the injection site of the investigational product | Does not interfere  with daily activities | Mild interference with daily activities | Prevent daily activities | Emergency department visit*  OR  Hospitalization |
| **Solicited Systemic Adverse Events** | | | | |
| Nausea/vomiting | Does not interfere  with daily activities  OR  1 to 2 episodes in 24 hours | Mild interference with daily activities  OR  More than 2 episodes in 24 hours | Prevent daily activities, it requires IV hydration | Emergency department visit*  OR  Hospitalization  OR  Hypovolemic shock |
| Fever | 37.8 – 38.4°C | 38.5 – 38.9°C | 39.0 – 40.0°C | >40°C |
| Chills | Slight cold sensation; chills, teeth chatter | Moderate chills in the entire body, it requires the use of narcotics | Serious or prolonged, no response to narcotics | ----- |
| Headache | Does not interfere  with daily activities | Repeated use of non-narcotic analgesic >24 hours  OR  Mild interference with daily activities | Any use of a narcotic analgesic  OR  Prevent daily activities | Emergency department visit*  OR  Hospitalization |
| Fatigue/malaise | Does not interfere  with daily activities | Mild interference with daily activities | Prevent daily activities | Emergency department visit*  OR  Hospitalization |
| Myalgia | Does not interfere  with daily activities | Mild interference with daily activities | Prevent daily activities | Emergency department visit*  OR  Hospitalization |
| Arthralgia | Does not interfere  with daily activities | Mild interference with daily activities | Prevent daily activities | Emergency department visit*  OR  Hospitalization |

* It requires 12 hours or more of admission to a ward or emergency department for the management of the adverse event.

^†^ The value recorded should be measured at the largest diameter and as a continuous variable.

## **II - Causal relationship classification**

All adverse events had their causal relationship to the investigational product classified according to the adapted classification of the "Uppsala Monitoring Centre" of the World^4^.

**Classification of the causal relationship of the adverse events**.

| **A reasonable causal relationship** | | | **A causal relationship NOT reasonable** | |
| --- | --- | --- | --- | --- |
| The Adverse Event is considered an Adverse Reaction | | | The Adverse Event cannot be considered an Adverse Reaction | |
| **Certain** | **Probable** | **Possible** | **Unlikely** | **Not related** |
| A clinical event, including a laboratory test abnormality (abnormal value) with a plausible temporal relationship to the administration of the intervention; | A clinical event, including a laboratory test abnormality (abnormal value) with a reasonable temporal relationship to the administration of the intervention; | A clinical event, including a laboratory test abnormality (abnormal value) with a reasonable temporal relationship to the administration of the intervention; | A clinical event, including a laboratory test abnormality (abnormal value) that due to the timing of the administration of the intervention has an unlikely relationship, but not impossible; | A clinical event, including a laboratory test abnormality (abnormal value) that due to the timing of the administration of the intervention has no relationship; |
| It cannot be explained by a concurrent disease or other intervention or medication;  The event must be definitive pharmacologically or phenomenologically (i.e., is an objective and specific disorder or a pharmacologically recognized phenomenon); | It is unlikely to be attributed to a concomitant disease or other medication or intervention; | It can also be explained by a concurrent disease or other medication or interventions; | Another disease or another medication provides a plausible explanation. | Another disease or another medication provides a plausible explanation. |
| The response to an interruption or withdrawal is plausible (pharmacologically, pathologically); | The response to the interruption or withdrawal is clinically reasonable; | There is a lack of information or lack of clarity about the withdrawal or interruption of treatment. |  |  |
| A satisfactory rechallenge procedure if necessary. | A rechallenge procedure is not necessary. |  |  |  |

**III – List of adverse events of special interest**

**Gastrointestinal disorders**

- Celiac disease
- Crohn’s disease
- Ulcerative colitis
- Ulcerative proctitis

**Liver disorders**

- Autoimmune cholangitis
- Autoimmune hepatitis
- Primary biliary cirrhosis
- Primary sclerosing cholangitis

**Metabolic disease**

- Addison’s disease
- Autoimmune thyroiditis (including Hashimoto thyroiditis)
- Diabetes mellitus type I
- Grave’s or Basedow’s disease

**Musculoskeletal disorders**

- Antisynthetase syndrome
- Dermatomyositis
- Juvenile chronic arthritis (including Still’s disease)
- Mixed connective tissue disorder
- Polymyalgia rheumatic
- Polymyositis
- Psoriatic arthropathy
- Relapsing polychondritis
- Rheumatoid arthritis
- Scleroderma, including diffuse systemic form and CREST syndrome
- Spondyloarthritis, including ankylosing spondylitis, reactive arthritis (Reiter’s Syndrome) and undifferentiated spondyloarthritis
- Systemic lupus erythematosus
- Systemic sclerosis

**Neuroinflammatory disorders**

- Acute disseminated encephalomyelitis, including site specific variants (e.g. non-infectious encephalitis, encephalomyelitis, myelitis, myeloradiculomyelitis)
- Cranial nerve disorders, including paralyses/paresis (e.g.Bell’s palsy)
- Guillain-Barré syndrome, including Miller Fisher syndrome and other variants
- Immune-mediated peripheral neuropathies and plexopathies, including chronic inflammatory demyelinating polyneuropathy, multifocal motor neuropathy and polyneuropathies associated with monoclonal gammopathy
- Multiple sclerosis
- Narcolepsy
- Optic neuritis
- Transverse Myelitis

**Skin disorders**

- Alopecia areata
- Autoimmune bullous skin diseases, including pemphigus, pemphigoid and dermatitis herpetiformis
- Cutaneous lupus erythematosus
- Erythema nodosum
- Morphoea
- Lichen planus
- Psoriasis
- Sweet’s syndrome
- Vitiligo

**Vasculitis**

- Large vessels vasculitis including giant cell arteritis such as Takayasu’s arteritis and temporal arteritis.
- Medium sized and/or small vessels vasculitis including: polyarteritis nodosa, Kawasaki’s disease, microscopic polyangiitis, Wegener’s granulomatosis, Churg-Strauss syndrome (allergic granulomatous angiitis), Buerger’s disease thromboangiitis obliterans, necrotizing vasculitis and anti-neurophil cytoplasmic antibody (ANCA) positive vasculitis (type unspecified), Henoch–Schönlein purpura, Behçet's syndrome, leukocytoclastic vasculitis.

**Others**

- Antiphospholipid syndrome
- Autoimmune hemolytic anemia
- Autoimmune glomerulonephritis (including IgA nephropathy, glomerulonephritis rapidly progressive, membranous glomerulonephritis, membranoproliferative glomerulonephritis, and mesangial proliferative glomerulonephritis)
- Autoimmune myocarditis/cardiomyopathy
- Autoimmune thrombocytopenia
- Goodpasture syndrome
- Idiopathic pulmonary fibrosis
- Pernicious anemia
- Raynaud’s phenomenon
- Sarcoidosis
- Sjögren's syndrome
- Stevens-Johnson syndrome
- Uveitis

1. **Appendix C – Tables and Charts**

**S. Table 1.** Proportion of participants with solicited AR occurring within 30 minutes after the first vaccination, by study group.

| **Solicited Adverse Reactions** | **Group 1A** | | | **Group 1B** | | | **Group 1C** | | | **Group 2A** | | | **Group 2B** | | | **Group 2C** | | | **Group 3** | | | **Group 4** | | |
| --- | --- | --- | --- | --- | --- | --- | --- | --- | --- | --- | --- | --- | --- | --- | --- | --- | --- | --- | --- | --- | --- | --- | --- | --- |
|  | **15μg H7N9 + IB160** | | | **7.5μg H7N9 + IB160** | | | **3.75μg H7N9 + IB160** | | | **15μg H7N9 + SE** | | | **7.5μg H7N9 + SE** | | | **3.75μg H7N9 + SE** | | | **15μg H7N9, without adjuvant** | | | **Placebo** | | |
|  | **(n=53)** | | | **(n=53)** | | | **(n=54)** | | | **(n=53)** | | | **(n=55)** | | | **(n=54)** | | | **(n=54)** | | | **(n=54)** | | |
|  | **n** | **%** | **(95%CI)** | **n** | **%** | **(95%CI)** | **n** | **%** | **(95%CI)** | **n** | **%** | **(95%CI)** | **n** | **%** | **(95%CI)** | **n** | **%** | **(95%CI)** | **n** | **%** | **(95%CI)** | **n** | **%** | **(95%CI)** |
| **Local** |  |  |  |  |  |  |  |  |  |  |  |  |  |  |  |  |  |  |  |  |  |  |  |  |
| Bruise | 1 | 1.9 | (0.0 - 10.1) | 0 | 0.0 | (0.0 - 6.7) | 0 | 0.0 | (0.0 - 6.6) | 0 | 0.0 | (0.0 - 6.7) | 0 | 0.0 | (0.0 - 6.5) | 0 | 0.0 | (0.0 - 6.6) | 0 | 0.0 | (0.0 - 6.6) | 0 | 0.0 | (0.0 - 6.6) |
| Erythema | 0 | 0.0 | (0.0 - 6.7) | 0 | 0.0 | (0.0 - 6.7) | 0 | 0.0 | (0.0 - 6.6) | 0 | 0.0 | (0.0 - 6.7) | 1 | 1.8 | (0.0 - 9.7) | 1 | 1.9 | (0.0 - 9.9) | 0 | 0.0 | (0.0 - 6.6) | 1 | 1.9 | (0.0 - 9.9) |
| Induration | 0 | 0.0 | (0.0 - 6.7) | 0 | 0.0 | (0.0 - 6.7) | 0 | 0.0 | (0.0 - 6.6) | 0 | 0.0 | (0.0 - 6.7) | 0 | 0.0 | (0.0 - 6.5) | 0 | 0.0 | (0.0 - 6.6) | 0 | 0.0 | (0.0 - 6.6) | 0 | 0.0 | (0.0 - 6.6) |
| Pain | 6 | 11.3 | (4.3 - 23) | 3 | 5.7 | (1.2 - 15.7) | 6 | 11.1 | (4.2 - 22.6) | 3 | 5.7 | (1.2 - 15.7) | 5 | 9.1 | (3.0 - 20.0) | 5 | 9.3 | (3.1 - 20.3) | 4 | 7.4 | (2.1 - 17.9) | 4 | 7.4 | (2.1 - 17.9) |
| Pruritus | 0 | 0.0 | (0.0 - 6.7) | 0 | 0.0 | (0.0 - 6.7) | 0 | 0.0 | (0.0 - 6.6) | 0 | 0.0 | (0.0 - 6.7) | 0 | 0.0 | (0.0 - 6.5) | 0 | 0.0 | (0.0 - 6.6) | 0 | 0.0 | (0.0 - 6.6) | 1 | 1.9 | (0.0 - 9.9) |
| Swelling | 0 | 0.0 | (0.0 - 6.7) | 0 | 0.0 | (0.0 - 6.7) | 0 | 0.0 | (0.0 - 6.6) | 0 | 0.0 | (0.0 - 6.7) | 0 | 0.0 | (0.0 - 6.5) | 0 | 0.0 | (0.0 - 6.6) | 0 | 0.0 | (0.0 - 6.6) | 0 | 0.0 | (0.0 - 6.6) |
| Tenderness | 0 | 0.0 | (0.0 - 6.7) | 0 | 0.0 | (0.0 - 6.7) | 0 | 0.0 | (0.0 - 6.6) | 1 | 1.9 | (0.0 - 10.1) | 2 | 3.6 | (0.4 - 12.5) | 0 | 0.0 | (0.0 - 6.6) | 0 | 0.0 | (0.0 - 6.6) | 0 | 0.0 | (0.0 - 6.6) |
| **Systemic** |  |  |  |  |  |  |  |  |  |  |  |  |  |  |  |  |  |  |  |  |  |  |  |  |
| Arthralgia | 0 | 0.0 | (0.0 - 6.7) | 0 | 0.0 | (0.0 - 6.7) | 0 | 0.0 | (0.0 - 6.6) | 0 | 0.0 | (0.0 - 6.7) | 1 | 1.8 | (0.0 - 9.7) | 0 | 0.0 | (0.0 - 6.6) | 0 | 0.0 | (0.0 - 6.6) | 0 | 0.0 | (0.0 - 6.6) |
| Chills | 0 | 0.0 | (0.0 - 6.7) | 0 | 0.0 | (0.0 - 6.7) | 0 | 0.0 | (0.0 - 6.6) | 0 | 0.0 | (0.0 - 6.7) | 1 | 1.8 | (0.0 - 9.7) | 0 | 0.0 | (0.0 - 6.6) | 0 | 0.0 | (0.0 - 6.6) | 0 | 0.0 | (0.0 - 6.6) |
| Discomfort | 0 | 0.0 | (0.0 - 6.7) | 0 | 0.0 | (0.0 - 6.7) | 0 | 0.0 | (0.0 - 6.6) | 0 | 0.0 | (0.0 - 6.7) | 0 | 0.0 | (0.0 - 6.5) | 0 | 0.0 | (0.0 - 6.6) | 0 | 0.0 | (0.0 - 6.6) | 0 | 0.0 | (0.0 - 6.6) |
| Fatigue | 0 | 0.0 | (0.0 - 6.7) | 0 | 0.0 | (0.0 - 6.7) | 0 | 0.0 | (0.0 - 6.6) | 0 | 0.0 | (0.0 - 6.7) | 0 | 0.0 | (0.0 - 6.5) | 0 | 0.0 | (0.0 - 6.6) | 0 | 0.0 | (0.0 - 6.6) | 0 | 0.0 | (0.0 - 6.6) |
| Headache | 0 | 0.0 | (0.0 - 6.7) | 0 | 0.0 | (0.0 - 6.7) | 0 | 0.0 | (0.0 - 6.6) | 0 | 0.0 | (0.0 - 6.7) | 1 | 1.8 | (0.0 - 9.7) | 0 | 0.0 | (0.0 - 6.6) | 0 | 0.0 | (0.0 - 6.6) | 0 | 0.0 | (0.0 - 6.6) |
| Myalgia | 0 | 0.0 | (0.0 - 6.7) | 0 | 0.0 | (0.0 - 6.7) | 0 | 0.0 | (0.0 - 6.6) | 0 | 0.0 | (0.0 - 6.7) | 0 | 0.0 | (0.0 - 6.5) | 0 | 0.0 | (0.0 - 6.6) | 0 | 0.0 | (0.0 - 6.6) | 0 | 0.0 | (0.0 - 6.6) |
| Nausea | 0 | 0.0 | (0.0 - 6.7) | 0 | 0.0 | (0.0 - 6.7) | 0 | 0.0 | (0.0 - 6.6) | 0 | 0.0 | (0.0 - 6.7) | 0 | 0.0 | (0.0 - 6.5) | 1 | 1.9 | (0.0 - 9.9) | 0 | 0.0 | (0.0 - 6.6) | 0 | 0.0 | (0.0 - 6.6) |
| Pyrexia | 0 | 0.0 | (0.0 - 6.7) | 0 | 0.0 | (0.0 - 6.7) | 0 | 0.0 | (0.0 - 6.6) | 0 | 0.0 | (0.0 - 6.7) | 0 | 0.0 | (0.0 - 6.5) | 0 | 0.0 | (0.0 - 6.6) | 0 | 0.0 | (0.0 - 6.6) | 0 | 0.0 | (0.0 - 6.6) |
| Vomiting | 0 | 0.0 | (0.0 - 6.7) | 0 | 0.0 | (0.0 - 6.7) | 0 | 0.0 | (0.0 - 6.6) | 0 | 0.0 | (0.0 - 6.7) | 0 | 0.0 | (0.0 - 6.5) | 0 | 0.0 | (0.0 - 6.6) | 0 | 0.0 | (0.0 - 6.6) | 0 | 0.0 | (0.0 - 6.6) |
| **TOTAL** | 7 | 13.2 | (5.5 - 25.3) | 3 | 5.7 | (1.2 - 15.7) | 6 | 11.1 | (4.2 - 22.6) | 4 | 7.5 | (2.1 - 18.2) | 7 | 12.7 | (5.3 - 24.5) | 7 | 13.0 | (5.4 - 24.9) | 4 | 7.4 | (2.1 - 17.9) | 5 | 9.3 | (3.1 - 20.3) |
| adj: adjuvant; 95%CI: 95% confidence interval | | | | | | | | | | | | | | | | | | | | | | | | |

**S. Table 2.** Proportion of participants with solicited AR occurring within 30 minutes after the second vaccination, by study group.

| **Solicited Adverse Reactions** | **Group 1A** | | | **Group 1B** | | | **Group 1C** | | | **Group 2A** | | | **Group 2B** | | | **Group 2C** | | | **Group 3** | | | **Group 4** | | |
| --- | --- | --- | --- | --- | --- | --- | --- | --- | --- | --- | --- | --- | --- | --- | --- | --- | --- | --- | --- | --- | --- | --- | --- | --- |
|  | **15μg H7N9 + IB160** | | | **7.5μg H7N9 + IB160** | | | **3.75μg H7N9 + IB160** | | | **15μg H7N9 + SE** | | | **7.5μg H7N9 + SE** | | | **3.75μg H7N9 + SE** | | | **15μg H7N9, without adjuvant** | | | **Placebo** | | |
|  | **(n=53)** | | | **(n=53)** | | | **(n=54)** | | | **(n=53)** | | | **(n=55)** | | | **(n=54)** | | | **(n=54)** | | | **(n=54)** | | |
|  | **n** | **%** | **(95%CI)** | **n** | **%** | **(95%CI)** | **n** | **%** | **(95%CI)** | **n** | **%** | **(95%CI)** | **n** | **%** | **(95%CI)** | **n** | **%** | **(95%CI)** | **n** | **%** | **(95%CI)** | **n** | **%** | **(95%CI)** |
| **Local** |  |  |  |  |  |  |  |  |  |  |  |  |  |  |  |  |  |  |  |  |  |  |  |  |
| Bruise | 0 | 0.0 | (0.0 - 7.1) | 0 | 0.0 | (0.0 - 7.4) | 0 | 0.0 | (0.0 - 7.0) | 0 | 0.0 | (0.0 - 7.4) | 0 | 0.0 | (0.0 - 6.7) | 0 | 0.0 | (0.0 - 7.0) | 0 | 0.0 | (0.0 - 7.0) | 0 | 0.0 | (0.0 - 7.4) |
| Erythema | 1 | 2.0 | (0.1 - 10.6) | 0 | 0.0 | (0.0 - 7.4) | 0 | 0.0 | (0.0 - 7.0) | 0 | 0.0 | (0.0 - 7.4) | 0 | 0.0 | (0.0 - 6.7) | 0 | 0.0 | (0.0 - 7.0) | 0 | 0.0 | (0.0 - 7.0) | 0 | 0.0 | (0.0 - 7.4) |
| Induration | 0 | 0.0 | (0.0 - 7.1) | 0 | 0.0 | (0.0 - 7.4) | 0 | 0.0 | (0.0 - 7.0) | 0 | 0.0 | (0.0 - 7.4) | 0 | 0.0 | (0.0 - 6.7) | 0 | 0.0 | (0.0 - 7.0) | 0 | 0.0 | (0.0 - 7.0) | 0 | 0.0 | (0.0 - 7.4) |
| Pain | 7 | 14.0 | (5.8 - 26.7) | 0 | 0.0 | (0.0 - 7.4) | 5 | 9.8 | (3.3 - 21.4) | 2 | 4.2 | (0.5 - 14.3) | 4 | 7.5 | (2.1 - 18.2) | 3 | 5.9 | (1.2 - 16.2) | 1 | 2.0 | (0.0 - 10.4) | 2 | 4.2 | (0.5 - 14.3) |
| Pruritus | 0 | 0.0 | (0.0 - 7.1) | 1 | 2.1 | (0.1 - 11.1) | 0 | 0.0 | (0.0 - 7.0) | 0 | 0.0 | (0.0 - 7.4) | 0 | 0.0 | (0.0 - 6.7) | 0 | 0.0 | (0.0 - 7.0) | 0 | 0.0 | (0.0 - 7.0) | 0 | 0.0 | (0.0 - 7.4) |
| Swelling | 0 | 0.0 | (0.0 - 7.1) | 0 | 0.0 | (0.0 - 7.4) | 0 | 0.0 | (0.0 - 7.0) | 0 | 0.0 | (0.0 - 7.4) | 0 | 0.0 | (0.0 - 6.7) | 0 | 0.0 | (0.0 - 7.0) | 1 | 2.0 | (0.0 - 10.4) | 0 | 0.0 | (0.0 - 7.4) |
| Tenderness | 1 | 2.0 | (0.1 - 10.6) | 0 | 0.0 | (0.0 - 7.4) | 1 | 2.0 | (0.0 - 10.4) | 0 | 0.0 | (0.0 - 7.4) | 0 | 0.0 | (0.0 - 6.7) | 0 | 0.0 | (0.0 - 7.0) | 0 | 0.0 | (0.0 - 7.0) | 0 | 0.0 | (0.0 - 7.4) |
| **Systemic** |  |  |  |  |  |  |  |  |  |  |  |  |  |  |  |  |  |  |  |  |  |  |  |  |
| Arthralgia | 0 | 0.0 | (0.0 - 7.1) | 0 | 0.0 | (0.0 - 7.4) | 0 | 0.0 | (0.0 - 7.0) | 0 | 0.0 | (0.0 - 7.4) | 0 | 0.0 | (0.0 - 6.7) | 0 | 0.0 | (0.0 - 7.0) | 0 | 0.0 | (0.0 - 7.0) | 0 | 0.0 | (0.0 - 7.4) |
| Chills | 0 | 0.0 | (0.0 - 7.1) | 0 | 0.0 | (0.0 - 7.4) | 0 | 0.0 | (0.0 - 7.0) | 0 | 0.0 | (0.0 - 7.4) | 0 | 0.0 | (0.0 - 6.7) | 1 | 2.0 | (0.0 - 10.4) | 0 | 0.0 | (0.0 - 7.0) | 0 | 0.0 | (0.0 - 7.4) |
| Discomfort | 0 | 0.0 | (0.0 - 7.1) | 0 | 0.0 | (0.0 - 7.4) | 0 | 0.0 | (0.0 - 7.0) | 0 | 0.0 | (0.0 - 7.4) | 0 | 0.0 | (0.0 - 6.7) | 0 | 0.0 | (0.0 - 7.0) | 0 | 0.0 | (0.0 - 7.0) | 0 | 0.0 | (0.0 - 7.4) |
| Fatigue | 0 | 0.0 | (0.0 - 7.1) | 0 | 0.0 | (0.0 - 7.4) | 0 | 0.0 | (0.0 - 7.0) | 0 | 0.0 | (0.0 - 7.4) | 0 | 0.0 | (0.0 - 6.7) | 0 | 0.0 | (0.0 - 7.0) | 0 | 0.0 | (0.0 - 7.0) | 0 | 0.0 | (0.0 - 7.4) |
| Headache | 0 | 0.0 | (0.0 - 7.1) | 1 | 2.1 | (0.1 - 11.1) | 0 | 0.0 | (0.0 - 7.0) | 0 | 0.0 | (0.0 - 7.4) | 1 | 1.9 | (0.0 - 10.1) | 0 | 0.0 | (0.0 - 7.0) | 0 | 0.0 | (0.0 - 7.0) | 0 | 0.0 | (0.0 - 7.4) |
| Myalgia | 0 | 0.0 | (0.0 - 7.1) | 0 | 0.0 | (0.0 - 7.4) | 0 | 0.0 | (0.0 - 7.0) | 0 | 0.0 | (0.0 - 7.4) | 0 | 0.0 | (0.0 - 6.7) | 0 | 0.0 | (0.0 - 7.0) | 0 | 0.0 | (0.0 - 7.0) | 0 | 0.0 | (0.0 - 7.4) |
| Nausea | 0 | 0.0 | (0.0 - 7.1) | 0 | 0.0 | (0.0 - 7.4) | 0 | 0.0 | (0.0 - 7.0) | 0 | 0.0 | (0.0 - 7.4) | 0 | 0.0 | (0.0 - 6.7) | 0 | 0.0 | (0.0 - 7.0) | 0 | 0.0 | (0.0 - 7.0) | 0 | 0.0 | (0.0 - 7.4) |
| Pyrexia | 0 | 0.0 | (0.0 - 7.1) | 0 | 0.0 | (0.0 - 7.4) | 0 | 0.0 | (0.0 - 7.0) | 0 | 0.0 | (0.0 - 7.4) | 0 | 0.0 | (0.0 - 6.7) | 0 | 0.0 | (0.0 - 7.0) | 0 | 0.0 | (0.0 - 7.0) | 0 | 0.0 | (0.0 - 7.4) |
| Vomiting | 0 | 0.0 | (0.0 - 7.1) | 0 | 0.0 | (0.0 - 7.4) | 0 | 0.0 | (0.0 - 7.0) | 0 | 0.0 | (0.0 - 7.4) | 0 | 0.0 | (0.0 - 6.7) | 0 | 0.0 | (0.0 - 7.0) | 0 | 0.0 | (0.0 - 7.0) | 0 | 0.0 | (0.0 - 7.4) |
| **TOTAL** | 7 | 14.0 | (5.8 - 26.7) | 2 | 4.2 | (0.5 - 14.3) | 5 | 9.8 | (3.3 - 21.4) | 2 | 4.2 | (0.5 - 14.3) | 4 | 7.5 | (2.1 - 18.2) | 4 | 7.8 | (2.2 - 18.9) | 1 | 2.0 | (0.0 - 10.4) | 2 | 4.2 | (0.5 - 14.3) |
| adj: adjuvant; 95%CI: 95% confidence interval | | | | | | | | | | | | | | | | | | | | | | | | |

**S. Table 3.** Proportion of participants with solicited AR occurring 7 days after the first vaccination, by study group.

| **Solicited Adverse Reactions** | **Group 1A** | | | **Group 1B** | | | **Group 1C** | | | **Group 2A** | | | **Group 2B** | | | **Group 2C** | | | **Group 3** | | | **Group 4** | | |
| --- | --- | --- | --- | --- | --- | --- | --- | --- | --- | --- | --- | --- | --- | --- | --- | --- | --- | --- | --- | --- | --- | --- | --- | --- |
|  | **15μg H7N9 + IB160** | | | **7.5μg H7N9 + IB160** | | | **3.75μg H7N9 + IB160** | | | **15μg H7N9 + SE** | | | **7.5μg H7N9 + SE** | | | **3.75μg H7N9 + SE** | | | **15μg H7N9, without adjuvant** | | | **Placebo** | | |
|  | **(n=53)** | | | **(n=53)** | | | **(n=54)** | | | **(n=53)** | | | **(n=55)** | | | **(n=54)** | | | **(n=54)** | | | **(n=54)** | | |
|  | **n** | **%** | **(95%CI)** | **n** | **%** | **(95%CI)** | **n** | **%** | **(95%CI)** | **n** | **%** | **(95%CI)** | **n** | **%** | **(95%CI)** | **n** | **%** | **(95%CI)** | **n** | **%** | **(95%CI)** | **n** | **%** | **(95%CI)** |
| **Local** |  |  |  |  |  |  |  |  |  |  |  |  |  |  |  |  |  |  |  |  |  |  |  |  |
| Bruise | 3 | 5.7 | (1.2 - 15.7) | 0 | 0.0 | (0.0 - 6.7) | 0 | 0.0 | (0.0 - 6.6) | 1 | 1.9 | (0.0 - 10.1) | 1 | 1.8 | (0.0 - 9.7) | 1 | 1.9 | (0.0 - 9.9) | 0 | 0.0 | (0.0 - 6.6) | 0 | 0.0 | (0.0 - 6.6) |
| Erythema | 2 | 3.8 | (0.5 - 13.0) | 2 | 3.8 | (0.5 - 13.0) | 1 | 1.9 | (0.0 - 9.9) | 0 | 0.0 | (0.0 - 6.7) | 1 | 1.8 | (0.0 - 9.7) | 2 | 3.7 | (0.5 - 12.7) | 0 | 0.0 | (0.0 - 6.6) | 1 | 1.9 | (0.0 - 9.9) |
| Induration | 1 | 1.9 | (0.0 - 10.1) | 3 | 5.7 | (1.2 - 15.7) | 0 | 0.0 | (0.0 - 6.6) | 0 | 0.0 | (0.0 - 6.7) | 2 | 3.6 | (0.4 - 12.5) | 2 | 3.7 | (0.5 - 12.7) | 0 | 0.0 | (0.0 - 6.6) | 0 | 0.0 | (0.0 - 6.6) |
| Pain | 39 | 73.6 | (59.7 - 84.7) | 32 | 60.4 | (46.0 - 73.5) | 40 | 74.1 | (60.3 - 85.0) | 28 | 52.8 | (38.6 - 66.7) | 32 | 58.2 | (44.1 - 71.3) | 23 | 42.6 | (29.2 - 56.8) | 23 | 42.6 | (29.2 - 56.8) | 12 | 22.2 | (12.0 - 35.6) |
| Pruritus | 0 | 0.0 | (0.0 - 6.7) | 5 | 9.4 | (3.1 - 20.7) | 1 | 1.9 | (0.0 - 9.9) | 1 | 1.9 | (0.0 - 10.1) | 1 | 1.8 | (0.0 - 9.7) | 2 | 3.7 | (0.5 - 12.7) | 0 | 0.0 | (0.0 - 6.6) | 2 | 3.7 | (0.5 - 12.7) |
| Swelling | 5 | 9.4 | (3.1 - 20.7) | 6 | 11.3 | (4.3 - 23.0) | 3 | 5.6 | (1.2 - 15.4) | 3 | 5.7 | (1.2 - 15.7) | 2 | 3.6 | (0.4 - 12.5) | 2 | 3.7 | (0.5 - 12.7) | 0 | 0.0 | (0.0 - 6.6) | 1 | 1.9 | (0.0 - 9.9) |
| Tenderness | 14 | 26.4 | (15.3 - 40.3) | 18 | 34.0 | (21.5 - 48.3) | 8 | 14.8 | (6.6 - 27.1) | 12 | 22.6 | (12.3 - 36.2) | 9 | 16.4 | (7.8 - 28.8) | 6 | 11.1 | (4.2 - 22.6) | 3 | 5.6 | (1.2 - 15.4) | 5 | 9.3 | (3.1 - 20.3) |
| **Systemic** |  |  |  |  |  |  |  |  |  |  |  |  |  |  |  |  |  |  |  |  |  |  |  |  |
| Arthralgia | 5 | 9.4 | (3.1 - 20.7) | 3 | 5.7 | (1.2 - 15.7) | 7 | 13.0 | (5.4 - 24.9) | 5 | 9.4 | (3.1 - 20.7) | 4 | 7.3 | (2.0 - 17.6) | 2 | 3.7 | (0.5 - 12.7) | 5 | 9.3 | (3.1 - 20.3) | 3 | 5.6 | (1.2 - 15.4) |
| Chills | 2 | 3.8 | (0.5 - 13.0) | 1 | 1.9 | (0.0 - 10.1) | 1 | 1.9 | (0.0 - 9.9) | 1 | 1.9 | (0.0 - 10.1) | 2 | 3.6 | (0.4 - 12.5) | 2 | 3.7 | (0.5 - 12.7) | 2 | 3.7 | (0.5 - 12.7) | 1 | 1.9 | (0.0 - 9.9) |
| Discomfort | 4 | 7.5 | (2.1 - 18.2) | 3 | 5.7 | (1.2 - 15.7) | 1 | 1.9 | (0.0 - 9.9) | 2 | 3.8 | (0.5 - 13.0) | 2 | 3.6 | (0.4 - 12.5) | 1 | 1.9 | (0.0 - 9.9) | 2 | 3.7 | (0.5 - 12.7) | 3 | 5.6 | (1.2 - 15.4) |
| Fatigue | 3 | 5.7 | (1.2 - 15.7) | 5 | 9.4 | (3.1 - 20.7) | 1 | 1.9 | (0.0 - 9.9) | 1 | 1.9 | (0.0 - 10.1) | 1 | 1.8 | (0.0 - 9.7) | 0 | 0.0 | (0.0 - 6.6) | 2 | 3.7 | (0.5 - 12.7) | 3 | 5.6 | (1.2 - 15.4) |
| Headache | 10 | 18.9 | (9.4 - 32.0) | 6 | 11.3 | (4.3 - 23.0) | 9 | 16.7 | (7.9 - 29.3) | 9 | 17.0 | (8.1 - 29.8) | 10 | 18.2 | (9.1 - 30.9) | 5 | 9.3 | (3.1 - 20.3) | 8 | 14.8 | (6.6 - 27.1) | 8 | 14.8 | (6.6 - 27.1) |
| Myalgia | 9 | 17.0 | (8.1 - 29.8) | 7 | 13.2 | (5.5 - 25.3) | 9 | 16.7 | (7.9 - 29.3) | 5 | 9.4 | (3.1 - 20.7) | 4 | 7.3 | (2.0 - 17.6) | 6 | 11.1 | (4.2 - 22.6) | 7 | 13.0 | (5.4 - 24.9) | 6 | 11.1 | (4.2 - 22.6) |
| Nausea | 2 | 3.8 | (0.5 - 13.0) | 8 | 15.1 | (6.7 - 27.6) | 2 | 3.7 | (0.5 - 12.7) | 2 | 3.8 | (0.5 - 13.0) | 2 | 3.6 | (0.4 - 12.5) | 4 | 7.4 | (2.1 - 17.9) | 4 | 7.4 | (2.1 - 17.9) | 3 | 5.6 | (1.2 - 15.4) |
| Pyrexia | 1 | 1.9 | (0.0 - 10.1) | 1 | 1.9 | (0.0 - 10.1) | 0 | 0.0 | (0.0 - 6.6) | 0 | 0.0 | (0.0 - 6.7) | 1 | 1.8 | (0.0 - 9.7) | 0 | 0.0 | (0.0 - 6.6) | 1 | 1.9 | (0.0 - 9.9) | 0 | 0.0 | (0.0 - 6.6) |
| Vomiting | 0 | 0.0 | (0.0 - 6.7) | 2 | 3.8 | (0.5 - 13.0) | 0 | 0.0 | (0.0 - 6.6) | 0 | 0.0 | (0.0 - 6.7) | 1 | 1.8 | (0.0 - 9.7) | 1 | 1.9 | (0.0 - 9.9) | 0 | 0.0 | (0.0 - 6.6) | 0 | 0.0 | (0.0 - 6.6) |
| **TOTAL** | 46 | 86.8 | (74.7 - 94.5) | 40 | 75.5 | (61.7 - 86.2) | 45 | 83.3 | (70.7 - 92.1) | 33 | 62.3 | (47.9 - 75.2) | 38 | 69.1 | (55.2 - 80.9) | 31 | 57.4 | (43.2 - 70.8) | 28 | 51.9 | (37.8 - 65.7) | 25 | 46.3 | (32.6 - 60.4) |
| adj: adjuvant; 95%CI: 95% confidence interval | | | | | | | | | | | | | | | | | | | | | | | | |

**S. Table 4.** Proportion of participants with solicited AR occurring 7 days after the second vaccination, by study group.

| **Solicited Adverse Reactions** | **Group 1A** | | | **Group 1B** | | | **Group 1C** | | | **Group 2A** | | | **Group 2B** | | | **Group 2C** | | | **Group 3** | | | **Group 4** | | |
| --- | --- | --- | --- | --- | --- | --- | --- | --- | --- | --- | --- | --- | --- | --- | --- | --- | --- | --- | --- | --- | --- | --- | --- | --- |
|  | **15μg H7N9 + IB160** | | | **7.5μg H7N9 + IB160** | | | **3.75μg H7N9 + IB160** | | | **15μg H7N9 + SE** | | | **7.5μg H7N9 + SE** | | | **3.75μg H7N9 + SE** | | | **15μg H7N9, without adjuvant** | | | **Placebo** | | |
|  | **(n=53)** | | | **(n=53)** | | | **(n=54)** | | | **(n=53)** | | | **(n=55)** | | | **(n=54)** | | | **(n=54)** | | | **(n=54)** | | |
|  | **n** | **%** | **(95%CI)** | **n** | **%** | **(95%CI)** | **n** | **%** | **(95%CI)** | **n** | **%** | **(95%CI)** | **n** | **%** | **(95%CI)** | **n** | **%** | **(95%CI)** | **n** | **%** | **(95%CI)** | **n** | **%** | **(95%CI)** |
| **Local** |  |  |  |  |  |  |  |  |  |  |  |  |  |  |  |  |  |  |  |  |  |  |  |  |
| Bruise | 3 | 5.7 | (1.2 - 15.7) | 0 | 0.0 | (0.0 - 6.7) | 0 | 0.0 | (0.0 - 6.6) | 1 | 1.9 | (0.0 - 10.1) | 1 | 1.8 | (0.0 - 9.7) | 1 | 1.9 | (0.0 - 9.9) | 0 | 0.0 | (0.0 - 6.6) | 0 | 0.0 | (0.0 - 6.6) |
| Erythema | 2 | 3.8 | (0.5 - 13.0) | 2 | 3.8 | (0.5 - 13.0) | 1 | 1.9 | (0.0 - 9.9) | 0 | 0.0 | (0.0 - 6.7) | 1 | 1.8 | (0.0 - 9.7) | 2 | 3.7 | (0.5 - 12.7) | 0 | 0.0 | (0.0 - 6.6) | 1 | 1.9 | (0.0 - 9.9) |
| Induration | 1 | 1.9 | (0.0 - 10.1) | 3 | 5.7 | (1.2 - 15.7) | 0 | 0.0 | (0.0 - 6.6) | 0 | 0.0 | (0.0 - 6.7) | 2 | 3.6 | (0.4 - 12.5) | 2 | 3.7 | (0.5 - 12.7) | 0 | 0.0 | (0.0 - 6.6) | 0 | 0.0 | (0.0 - 6.6) |
| Pain | 39 | 73.6 | (59.7 - 84.7) | 32 | 60.4 | (46.0 - 73.5) | 40 | 74.1 | (60.3 - 85.0) | 28 | 52.8 | (38.6 - 66.7) | 32 | 58.2 | (44.1 - 71.3) | 23 | 42.6 | (29.2 - 56.8) | 23 | 42.6 | (29.2 - 56.8) | 12 | 22.2 | (12.0 - 35.6) |
| Pruritus | 0 | 0.0 | (0.0 - 6.7) | 5 | 9.4 | (3.1 - 20.7) | 1 | 1.9 | (0.0 - 9.9) | 1 | 1.9 | (0.0 - 10.1) | 1 | 1.8 | (0.0 - 9.7) | 2 | 3.7 | (0.5 - 12.7) | 0 | 0.0 | (0.0 - 6.6) | 2 | 3.7 | (0.5 - 12.7) |
| Swelling | 5 | 9.4 | (3.1 - 20.7) | 6 | 11.3 | (4.3 - 23.0) | 3 | 5.6 | (1.2 - 15.4) | 3 | 5.7 | (1.2 - 15.7) | 2 | 3.6 | (0.4 - 12.5) | 2 | 3.7 | (0.5 - 12.7) | 0 | 0.0 | (0.0 - 6.6) | 1 | 1.9 | (0.0 - 9.9) |
| Tenderness | 14 | 26.4 | (15.3 - 40.3) | 18 | 34.0 | (21.5 - 48.3) | 8 | 14.8 | (6.6 - 27.1) | 12 | 22.6 | (12.3 - 36.2) | 9 | 16.4 | (7.8 - 28.8) | 6 | 11.1 | (4.2 - 22.6) | 3 | 5.6 | (1.2 - 15.4) | 5 | 9.3 | (3.1 - 20.3) |
| **Systemic** |  |  |  |  |  |  |  |  |  |  |  |  |  |  |  |  |  |  |  |  |  |  |  |  |
| Arthralgia | 5 | 9.4 | (3.1 - 20.7) | 3 | 5.7 | (1.2 - 15.7) | 7 | 13.0 | (5.4 - 24.9) | 5 | 9.4 | (3.1 - 20.7) | 4 | 7.3 | (2.0 - 17.6) | 2 | 3.7 | (0.5 - 12.7) | 5 | 9.3 | (3.1 - 20.3) | 3 | 5.6 | (1.2 - 15.4) |
| Chills | 2 | 3.8 | (0.5 - 13.0) | 1 | 1.9 | (0.0 - 10.1) | 1 | 1.9 | (0.0 - 9.9) | 1 | 1.9 | (0.0 - 10.1) | 2 | 3.6 | (0.4 - 12.5) | 2 | 3.7 | (0.5 - 12.7) | 2 | 3.7 | (0.5 - 12.7) | 1 | 1.9 | (0.0 - 9.9) |
| Discomfort | 4 | 7.5 | (2.1 - 18.2) | 3 | 5.7 | (1.2 - 15.7) | 1 | 1.9 | (0.0 - 9.9) | 2 | 3.8 | (0.5 - 13.0) | 2 | 3.6 | (0.4 - 12.5) | 1 | 1.9 | (0.0 - 9.9) | 2 | 3.7 | (0.5 - 12.7) | 3 | 5.6 | (1.2 - 15.4) |
| Fatigue | 3 | 5.7 | (1.2 - 15.7) | 5 | 9.4 | (3.1 - 20.7) | 1 | 1.9 | (0.0 - 9.9) | 1 | 1.9 | (0.0 - 10.1) | 1 | 1.8 | (0.0 - 9.7) | 0 | 0.0 | (0.0 - 6.6) | 2 | 3.7 | (0.5 - 12.7) | 3 | 5.6 | (1.2 - 15.4) |
| Headache | 10 | 18.9 | (9.4 - 32.0) | 6 | 11.3 | (4.3 - 23.0) | 9 | 16.7 | (7.9 - 29.3) | 9 | 17.0 | (8.1 - 29.8) | 10 | 18.2 | (9.1 - 30.9) | 5 | 9.3 | (3.1 - 20.3) | 8 | 14.8 | (6.6 - 27.1) | 8 | 14.8 | (6.6 - 27.1) |
| Myalgia | 9 | 17.0 | (8.1 - 29.8) | 7 | 13.2 | (5.5 - 25.3) | 9 | 16.7 | (7.9 - 29.3) | 5 | 9.4 | (3.1 - 20.7) | 4 | 7.3 | (2.0 - 17.6) | 6 | 11.1 | (4.2 - 22.6) | 7 | 13.0 | (5.4 - 24.9) | 6 | 11.1 | (4.2 - 22.6) |
| Nausea | 2 | 3.8 | (0.5 - 13.0) | 8 | 15.1 | (6.7 - 27.6) | 2 | 3.7 | (0.5 - 12.7) | 2 | 3.8 | (0.5 - 13.0) | 2 | 3.6 | (0.4 - 12.5) | 4 | 7.4 | (2.1 - 17.9) | 4 | 7.4 | (2.1 - 17.9) | 3 | 5.6 | (1.2 - 15.4) |
| Pyrexia | 1 | 1.9 | (0.0 - 10.1) | 1 | 1.9 | (0.0 - 10.1) | 0 | 0.0 | (0.0 - 6.6) | 0 | 0.0 | (0.0 - 6.7) | 1 | 1.8 | (0.0 - 9.7) | 0 | 0.0 | (0.0 - 6.6) | 1 | 1.9 | (0.0 - 9.9) | 0 | 0.0 | (0.0 - 6.6) |
| Vomiting | 0 | 0.0 | (0.0 - 6.7) | 2 | 3.8 | (0.5 - 13.0) | 0 | 0.0 | (0.0 - 6.6) | 0 | 0.0 | (0.0 - 6.7) | 1 | 1.8 | (0.0 - 9.7) | 1 | 1.9 | (0.0 - 9.9) | 0 | 0.0 | (0.0 - 6.6) | 0 | 0.0 | (0.0 - 6.6) |
| **TOTAL** | 46 | 86.8 | (74.7 - 94.5) | 40 | 75.5 | (61.7 - 86.2) | 45 | 83.3 | (70.7 - 92.1) | 33 | 62.3 | (47.9 - 75.2) | 38 | 69.1 | (55.2 - 80.9) | 31 | 57.4 | (43.2 - 70.8) | 28 | 51.9 | (37.8 - 65.7) | 25 | 46.3 | (32.6 - 60.4) |
| adj: adjuvant; 95%CI: 95% confidence interval | | | | | | | | | | | | | | | | | | | | | | | | |

**S. Table 5.** Proportion of participants with solicited AR occurring 28 days after the first vaccination, by study group.

| **Solicited Adverse Reactions** | **Group 1A** | | | **Group 1B** | | | **Group 1C** | | | **Group 2A** | | | **Group 2B** | | | **Group 2C** | | | **Group 3** | | | **Group 4** | | |
| --- | --- | --- | --- | --- | --- | --- | --- | --- | --- | --- | --- | --- | --- | --- | --- | --- | --- | --- | --- | --- | --- | --- | --- | --- |
|  | **15μg H7N9 + IB160** | | | **7.5μg H7N9 + IB160** | | | **3.75μg H7N9 + IB160** | | | **15μg H7N9 + SE** | | | **7.5μg H7N9 + SE** | | | **3.75μg H7N9 + SE** | | | **15μg H7N9, without adjuvant** | | | **Placebo** | | |
|  | **(n=53)** | | | **(n=53)** | | | **(n=54)** | | | **(n=53)** | | | **(n=55)** | | | **(n=54)** | | | **(n=54)** | | | **(n=54)** | | |
|  | **n** | **%** | **(95%CI)** | **n** | **%** | **(95%CI)** | **n** | **%** | **(95%CI)** | **n** | **%** | **(95%CI)** | **n** | **%** | **(95%CI)** | **n** | **%** | **(95%CI)** | **n** | **%** | **(95%CI)** | **n** | **%** | **(95%CI)** |
| **Local** |  |  |  |  |  |  |  |  |  |  |  |  |  |  |  |  |  |  |  |  |  |  |  |  |
| Bruise | 3 | 5.7 | (1.2 - 15.7) | 0 | 0.0 | (0.0 - 6.7) | 0 | 0.0 | (0.0 - 6.6) | 1 | 1.9 | (0.0 - 10.1) | 1 | 1.8 | (0.0 - 9.7) | 1 | 1.9 | (0.0 - 9.9) | 0 | 0.0 | (0.0 - 6.6) | 0 | 0.0 | (0.0 - 6.6) |
| Erythema | 2 | 3.8 | (0.5 - 13.0) | 2 | 3.8 | (0.5 - 13.0) | 1 | 1.9 | (0.0 - 9.9) | 0 | 0.0 | (0.0 - 6.7) | 1 | 1.8 | (0.0 - 9.7) | 2 | 3.7 | (0.5 - 12.7) | 0 | 0.0 | (0.0 - 6.6) | 1 | 1.9 | (0.0 - 9.9) |
| Induration | 1 | 1.9 | (0.0 - 10.1) | 3 | 5.7 | (1.2 - 15.7) | 0 | 0.0 | (0.0 - 6.6) | 0 | 0.0 | (0.0 - 6.7) | 2 | 3.6 | (0.4 - 12.5) | 2 | 3.7 | (0.5 - 12.7) | 0 | 0.0 | (0.0 - 6.6) | 0 | 0.0 | (0.0 - 6.6) |
| Pain | 39 | 73.6 | (59.7 - 84.7) | 32 | 60.4 | (46.0 - 73.5) | 40 | 74.1 | (60.3 - 85.0) | 28 | 52.8 | (38.6 - 66.7) | 32 | 58.2 | (44.1 - 71.3) | 23 | 42.6 | (29.2 - 56.8) | 23 | 42.6 | (29.2 - 56.8) | 12 | 22.2 | (12.0 - 35.6) |
| Pruritus | 0 | 0.0 | (0.0 - 6.7) | 5 | 9.4 | (3.1 - 20.7) | 1 | 1.9 | (0.0 - 9.9) | 1 | 1.9 | (0.0 - 10.1) | 1 | 1.8 | (0.0 - 9.7) | 2 | 3.7 | (0.5 - 12.7) | 0 | 0.0 | (0.0 - 6.6) | 2 | 3.7 | (0.5 - 12.7) |
| Swelling | 5 | 9.4 | (3.1 - 20.7) | 6 | 11.3 | (4.3 - 23.0) | 3 | 5.6 | (1.2 - 15.4) | 3 | 5.7 | (1.2 - 15.7) | 2 | 3.6 | (0.4 - 12.5) | 2 | 3.7 | (0.5 - 12.7) | 0 | 0.0 | (0.0 - 6.6) | 1 | 1.9 | (0.0 - 9.9) |
| Tenderness | 14 | 26.4 | (15.3 - 40.3) | 18 | 34.0 | (21.5 - 48.3) | 8 | 14.8 | (6.6 - 27.1) | 12 | 22.6 | (12.3 - 36.2) | 9 | 16.4 | (7.8 - 28.8) | 6 | 11.1 | (4.2 - 22.6) | 3 | 5.6 | (1.2 - 15.4) | 5 | 9.3 | (3.1 - 20.3) |
| **Systemic** |  |  |  |  |  |  |  |  |  |  |  |  |  |  |  |  |  |  |  |  |  |  |  |  |
| Arthralgia | 5 | 9.4 | (3.1 - 20.7) | 3 | 5.7 | (1.2 - 15.7) | 7 | 13.0 | (5.4 - 24.9) | 5 | 9.4 | (3.1 - 20.7) | 4 | 7.3 | (2.0 - 17.6) | 2 | 3.7 | (0.5 - 12.7) | 5 | 9.3 | (3.1 - 20.3) | 3 | 5.6 | (1.2 - 15.4) |
| Chills | 2 | 3.8 | (0.5 - 13.0) | 1 | 1.9 | (0.0 - 10.1) | 1 | 1.9 | (0.0 - 9.9) | 1 | 1.9 | (0.0 - 10.1) | 2 | 3.6 | (0.4 - 12.5) | 2 | 3.7 | (0.5 - 12.7) | 2 | 3.7 | (0.5 - 12.7) | 1 | 1.9 | (0.0 - 9.9) |
| Discomfort | 4 | 7.5 | (2.1 - 18.2) | 3 | 5.7 | (1.2 - 15.7) | 1 | 1.9 | (0.0 - 9.9) | 2 | 3.8 | (0.5 - 13.0) | 2 | 3.6 | (0.4 - 12.5) | 1 | 1.9 | (0.0 - 9.9) | 2 | 3.7 | (0.5 - 12.7) | 3 | 5.6 | (1.2 - 15.4) |
| Fatigue | 3 | 5.7 | (1.2 - 15.7) | 5 | 9.4 | (3.1 - 20.7) | 1 | 1.9 | (0.0 - 9.9) | 1 | 1.9 | (0.0 - 10.1) | 1 | 1.8 | (0.0 - 9.7) | 0 | 0.0 | (0.0 - 6.6) | 2 | 3.7 | (0.5 - 12.7) | 3 | 5.6 | (1.2 - 15.4) |
| Headache | 10 | 18.9 | (9.4 - 32.0) | 6 | 11.3 | (4.3 - 23.0) | 9 | 16.7 | (7.9 - 29.3) | 9 | 17.0 | (8.1 - 29.8) | 10 | 18.2 | (9.1 - 30.9) | 5 | 9.3 | (3.1 - 20.3) | 8 | 14.8 | (6.6 - 27.1) | 8 | 14.8 | (6.6 - 27.1) |
| Myalgia | 9 | 17.0 | (8.1 - 29.8) | 7 | 13.2 | (5.5 - 25.3) | 9 | 16.7 | (7.9 - 29.3) | 5 | 9.4 | (3.1 - 20.7) | 4 | 7.3 | (2.0 - 17.6) | 6 | 11.1 | (4.2 - 22.6) | 7 | 13.0 | (5.4 - 24.9) | 6 | 11.1 | (4.2 - 22.6) |
| Nausea | 2 | 3.8 | (0.5 - 13.0) | 8 | 15.1 | (6.7 - 27.6) | 2 | 3.7 | (0.5 - 12.7) | 2 | 3.8 | (0.5 - 13.0) | 2 | 3.6 | (0.4 - 12.5) | 4 | 7.4 | (2.1 - 17.9) | 4 | 7.4 | (2.1 - 17.9) | 3 | 5.6 | (1.2 - 15.4) |
| Pyrexia | 1 | 1.9 | (0.0 - 10.1) | 1 | 1.9 | (0.0 - 10.1) | 0 | 0.0 | (0.0 - 6.6) | 0 | 0.0 | (0.0 - 6.7) | 1 | 1.8 | (0.0 - 9.7) | 0 | 0.0 | (0.0 - 6.6) | 1 | 1.9 | (0.0 - 9.9) | 0 | 0.0 | (0.0 - 6.6) |
| Vomiting | 0 | 0.0 | (0.0 - 6.7) | 2 | 3.8 | (0.5 - 13.0) | 0 | 0.0 | (0.0 - 6.6) | 0 | 0.0 | (0.0 - 6.7) | 1 | 1.8 | (0.0 - 9.7) | 1 | 1.9 | (0.0 - 9.9) | 0 | 0.0 | (0.0 - 6.6) | 0 | 0.0 | (0.0 - 6.6) |
| **TOTAL** | 46 | 86.8 | (74.7 - 94.5) | 40 | 75.5 | (61.7 - 86.2) | 45 | 83.3 | (70.7 - 92.1) | 33 | 62.3 | (47.9 - 75.2) | 38 | 69.1 | (55.2 - 80.9) | 31 | 57.4 | (43.2 - 70.8) | 28 | 51.9 | (37.8 - 65.7) | 25 | 46.3 | (32.6 - 60.4) |
| adj: adjuvant; 95%CI: 95% confidence interval | | | | | | | | | | | | | | | | | | | | | | | | |

**S. Table 6.** Proportion of participants with solicited AR occurring 28 days after the second vaccination, by study group.

| **Solicited Adverse Reactions** | **Group 1A** | | | **Group 1B** | | | **Group 1C** | | | **Group 2A** | | | **Group 2B** | | | **Group 2C** | | | **Group 3** | | | **Group 4** | | |
| --- | --- | --- | --- | --- | --- | --- | --- | --- | --- | --- | --- | --- | --- | --- | --- | --- | --- | --- | --- | --- | --- | --- | --- | --- |
|  | **15μg H7N9 + IB160** | | | **7.5μg H7N9 + IB160** | | | **3.75μg H7N9 + IB160** | | | **15μg H7N9 + SE** | | | **7.5μg H7N9 + SE** | | | **3.75μg H7N9 + SE** | | | **15μg H7N9, without adjuvant** | | | **Placebo** | | |
|  | **(n=53)** | | | **(n=53)** | | | **(n=54)** | | | **(n=53)** | | | **(n=55)** | | | **(n=54)** | | | **(n=54)** | | | **(n=54)** | | |
|  | **n** | **%** | **(95%CI)** | **n** | **%** | **(95%CI)** | **n** | **%** | **(95%CI)** | **n** | **%** | **(95%CI)** | **n** | **%** | **(95%CI)** | **n** | **%** | **(95%CI)** | **n** | **%** | **(95%CI)** | **n** | **%** | **(95%CI)** |
| **Local** |  |  |  |  |  |  |  |  |  |  |  |  |  |  |  |  |  |  |  |  |  |  |  |  |
| Bruise | 0 | 0.0 | (0.0 - 7.1) | 0 | 0.0 | (0.0 - 7.4) | 0 | 0.0 | (0.0 - 7.0) | 1 | 2.1 | (0.1 - 11.1) | 1 | 1.9 | (0.0 - 10.1) | 1 | 2.0 | (0.0 - 10.4) | 1 | 2.0 | (0.0 - 10.4) | 0 | 0.0 | (0.0 - 7.4) |
| Erythema | 2 | 4.0 | (0.5 - 13.7) | 1 | 2.1 | (0.1 - 11.1) | 1 | 2.0 | (0.0 - 10.4) | 0 | 0.0 | (0.0 - 7.4) | 1 | 1.9 | (0.0 - 10.1) | 1 | 2.0 | (0.0 - 10.4) | 1 | 2.0 | (0.0 - 10.4) | 0 | 0.0 | (0.0 - 7.4) |
| Induration | 1 | 2.0 | (0.1 - 10.6) | 0 | 0.0 | (0.0 - 7.4) | 1 | 2.0 | (0.0 - 10.4) | 1 | 2.1 | (0.1 - 11.1) | 2 | 3.8 | (0.5 - 13.0) | 2 | 3.9 | (0.5 - 13.5) | 0 | 0.0 | (0.0 - 7.0) | 1 | 2.1 | (0.1 - 11.1) |
| Pain | 29 | 58.0 | (43.2 - 71.8) | 32 | 66.7 | (51.6 - 79.6) | 30 | 58.8 | (44.2 - 72.4) | 19 | 39.6 | (25.8 - 54.7) | 19 | 35.8 | (23.1 - 50.2) | 22 | 43.1 | (29.3 - 57.8) | 11 | 21.6 | (11.3 - 35.3) | 8 | 16.7 | (7.5 - 30.2) |
| Pruritus | 0 | 0.0 | (0.0 - 7.1) | 1 | 2.1 | (0.1 - 11.1) | 1 | 2.0 | (0.0 - 10.4) | 0 | 0.0 | (0.0 - 7.4) | 1 | 1.9 | (0.0 - 10.1) | 1 | 2.0 | (0.0 - 10.4) | 3 | 5.9 | (1.2 - 16.2) | 0 | 0.0 | (0.0 - 7.4) |
| Swelling | 1 | 2.0 | (0.1 - 10.6) | 2 | 4.2 | (0.5 - 14.3) | 1 | 2.0 | (0.0 - 10.4) | 0 | 0.0 | (0.0 - 7.4) | 3 | 5.7 | (1.2 - 15.7) | 3 | 5.9 | (1.2 - 16.2) | 1 | 2.0 | (0.0 - 10.4) | 0 | 0.0 | (0.0 - 7.4) |
| Tenderness | 10 | 20.0 | (10.0 - 33.7) | 13 | 27.1 | (15.3 - 41.8) | 13 | 25.5 | (14.3 - 39.6) | 8 | 16.7 | (7.5 - 30.2) | 12 | 22.6 | (12.3 - 36.2) | 10 | 19.6 | (9.8 - 33.1) | 4 | 7.8 | (2.2 - 18.9) | 3 | 6.3 | (1.3 - 17.2) |
| **Systemic** |  |  |  |  |  |  |  |  |  |  |  |  |  |  |  |  |  |  |  |  |  |  |  |  |
| Arthralgia | 3 | 6.0 | (1.3 - 16.5) | 1 | 2.1 | (0.1 - 11.1) | 3 | 5.9 | (1.2 - 16.2) | 2 | 4.2 | (0.5 - 14.3) | 3 | 5.7 | (1.2 - 15.7) | 2 | 3.9 | (0.5 - 13.5) | 0 | 0.0 | (0.0 - 7.0) | 0 | 0.0 | (0.0 - 7.4) |
| Chills | 3 | 6.0 | (1.3 - 16.5) | 1 | 2.1 | (0.1 - 11.1) | 4 | 7.8 | (2.2 - 18.9) | 0 | 0.0 | (0.0 - 7.4) | 1 | 1.9 | (0.0 - 10.1) | 2 | 3.9 | (0.5 - 13.5) | 0 | 0.0 | (0.0 - 7.0) | 2 | 4.2 | (0.5 - 14.3) |
| Discomfort | 1 | 2.0 | (0.1 - 10.6) | 3 | 6.3 | (1.3 - 17.2) | 6 | 11.8 | (4.4 - 23.9) | 1 | 2.1 | (0.1 - 11.1) | 2 | 3.8 | (0.5 - 13.0) | 3 | 5.9 | (1.2 - 16.2) | 2 | 3.9 | (0.5 - 13.5) | 1 | 2.1 | (0.1 - 11.1) |
| Fatigue | 1 | 2.0 | (0.1 - 10.6) | 4 | 8.3 | (2.3 - 20.0) | 5 | 9.8 | (3.3 - 21.4) | 0 | 0.0 | (0.0 - 7.4) | 3 | 5.7 | (1.2 - 15.7) | 3 | 5.9 | (1.2 - 16.2) | 2 | 3.9 | (0.5 - 13.5) | 0 | 0.0 | (0.0 - 7.4) |
| Headache | 3 | 6.0 | (1.3 - 16.5) | 8 | 16.7 | (7.5 - 30.2) | 5 | 9.8 | (3.3 - 21.4) | 4 | 8.3 | (2.3 - 20.0) | 11 | 20.8 | (10.8 - 34.1) | 6 | 11.8 | (4.4 - 23.9) | 4 | 7.8 | (2.2 - 18.9) | 4 | 8.3 | (2.3 - 20.0) |
| Myalgia | 5 | 10.0 | (3.3 - 21.8) | 5 | 10.4 | (3.5 - 22.7) | 10 | 19.6 | (9.8 - 33.1) | 1 | 2.1 | (0.1 - 11.1) | 3 | 5.7 | (1.2 - 15.7) | 5 | 9.8 | (3.3 - 21.4) | 0 | 0.0 | (0.0 - 7.0) | 0 | 0.0 | (0.0 - 7.4) |
| Nausea | 1 | 2.0 | (0.1 - 10.6) | 0 | 0.0 | (0.0 - 7.4) | 3 | 5.9 | (1.2 - 16.2) | 2 | 4.2 | (0.5 - 14.3) | 2 | 3.8 | (0.5 - 13.0) | 3 | 5.9 | (1.2 - 16.2) | 3 | 5.9 | (1.2 - 16.2) | 2 | 4.2 | (0.5 - 14.3) |
| Pyrexia | 0 | 0.0 | (0.0 - 7.1) | 2 | 4.2 | (0.5 - 14.3) | 2 | 3.9 | (0.5 - 13.5) | 0 | 0.0 | (0.0 - 7.4) | 0 | 0.0 | (0.0 - 6.7) | 0 | 0.0 | (0.0 - 7.0) | 0 | 0.0 | (0.0 - 7.0) | 0 | 0.0 | (0.0 - 7.4) |
| Vomiting | 0 | 0.0 | (0.0 - 7.1) | 0 | 0.0 | (0.0 - 7.4) | 1 | 2.0 | (0.0 - 10.4) | 0 | 0.0 | (0.0 - 7.4) | 0 | 0.0 | (0.0 - 6.7) | 0 | 0.0 | (0.0 - 7.0) | 1 | 2.0 | (0.0 - 10.4) | 0 | 0.0 | (0.0 - 7.4) |
| **TOTAL** | 37 | 74.0 | (59.7 - 85.4) | 36 | 75.0 | (60.4 - 86.4) | 33 | 64.7 | (50.1 - 77.6) | 22 | 45.8 | (31.4 - 60.8) | 26 | 49.1 | (35.1 - 63.2) | 28 | 54.9 | (40.3 - 68.9) | 17 | 33.3 | (20.8 - 47.9) | 12 | 25.0 | (13.6 - 39.6) |
| adj: adjuvant; 95%CI: 95% confidence interval | | | | | | | | | | | | | | | | | | | | | | | | |

**S. Table 7.** Distribution of solicited AR, and medication use, occurring up to 28 days after the first vaccination, by study group.

| **Solicited Adverse Reactions** | **Group 1A** | | | **Group 1B** | | | **Group 1C** | | | **Group 2A** | | | **Group 2B** | | | **Group 2C** | | | **Group 3** | | | **Group 4** | | |
| --- | --- | --- | --- | --- | --- | --- | --- | --- | --- | --- | --- | --- | --- | --- | --- | --- | --- | --- | --- | --- | --- | --- | --- | --- |
|  | **15μg H7N9 + IB160** | | | **7.5μg H7N9 + IB160** | | | **3.75μg H7N9 + IB160** | | | **15μg H7N9 + SE** | | | **7.5μg H7N9 + SE** | | | **3.75μg H7N9 + SE** | | | **15μg H7N9, without adjuvant** | | | **Placebo** | | |
|  | **(n=53)** | | | **(n=53)** | | | **(n=54)** | | | **(n=53)** | | | **(n=55)** | | | **(n=54)** | | | **(n=54)** | | | **(n=54)** | | |
|  | **No.**  **AR**  **Total** | **AR with**  **medication** | | **No.**  **AR**  **Total** | **AR with**  **medication** | | **No.**  **AR**  **Total** | **AR with**  **medication** | | **No.**  **AR**  **Total** | **AR with**  **medication** | | **No.**  **AR**  **Total** | **AR with**  **medication** | | **No.**  **AR**  **Total** | **AR with**  **medication** | | **No.**  **AR**  **Total** | **AR with**  **medication** | | **No.**  **AR**  **Total** | **AR with**  **medication** | |
|  |  | **n** | **(%)** |  | **n** | **(%)** |  | **n** | **(%)** |  | **n** | **(%)** |  | **n** | **(%)** |  | **n** | **(%)** |  | **n** | **(%)** |  | **n** | **(%)** |
| **Local** |  |  |  |  |  |  |  |  |  |  |  |  |  |  |  |  |  |  |  |  |  |  |  |  |
| Bruise | 3 | 0 | (0.0) | 0 | - | - | 0 | - | - | 1 | 0 | (0.0) | 1 | 0 | (0.0) | 1 | 0 | (0.0) | 0 | - | - | 0 | - | - |
| Erythema | 2 | 0 | (0.0) | 3 | 0 | (0.0) | 1 | 0 | (0.0) | 0 | - | - | 1 | 0 | (0.0) | 2 | 0 | (0.0) | 0 | - | - | 1 | 0 | (0.0) |
| Induration | 1 | 0 | (0.0) | 3 | 0 | (0.0) | 0 | - | - | 0 | - | - | 2 | 0 | (0.0) | 2 | 0 | (0.0) | 0 | - | - | 0 | - | - |
| Pain | 41 | 4 | (9.8) | 33 | 3 | (9.1) | 41 | 0 | (0.0) | 28 | 0 | (0.0) | 32 | 2 | (6.3) | 24 | 0 | (0.0) | 24 | 2 | (8.3) | 12 | 0 | (0.0) |
| Pruritus | 0 | - | - | 6 | 1 | (16.7) | 1 | 0 | (0.0) | 1 | 0 | (0.0) | 1 | 0 | (0.0) | 2 | 0 | (0.0) | 0 | - | - | 2 | 0 | (0.0) |
| Swelling | 5 | 0 | (0.0) | 7 | 0 | (0.0) | 4 | 0 | (0.0) | 4 | 0 | (0.0) | 2 | 0 | (0.0) | 2 | 0 | (0.0) | 0 | - | - | 1 | 0 | (0.0) |
| Tenderness | 15 | 2 | (13.3) | 18 | 1 | (5.6) | 8 | 0 | (0.0) | 12 | 0 | (0.0) | 9 | 0 | (0.0) | 6 | 0 | (0.0) | 3 | 0 | (0.0) | 5 | 0 | (0.0) |
| **Systemic** |  |  |  |  |  |  |  |  |  |  |  |  |  |  |  |  |  |  |  |  |  |  |  |  |
| Arthralgia | 6 | 2 | (33.3) | 3 | 1 | (33.3) | 7 | 3 | (42.9) | 5 | 2 | (40.0) | 4 | 2 | (50.0) | 2 | 1 | (50.0) | 5 | 2 | (40.0) | 3 | 1 | (33.3) |
| Chills | 2 | 0 | (0.0) | 2 | 0 | (0.0) | 1 | 0 | (0.0) | 1 | 1 | (100) | 2 | 0 | (0.0) | 2 | 0 | (0.0) | 2 | 1 | (50.0) | 1 | 0 | (0.0) |
| Discomfort | 4 | 0 | (0.0) | 3 | 0 | (0.0) | 1 | 0 | (0.0) | 2 | 1 | (50.0) | 2 | 0 | (0.0) | 1 | 0 | (0.0) | 2 | 0 | (0.0) | 3 | 0 | (0.0) |
| Fatigue | 4 | 0 | (0.0) | 5 | 0 | (0.0) | 1 | 0 | (0.0) | 1 | 0 | (0.0) | 1 | 0 | (0.0) | 0 | - | - | 2 | 0 | (0.0) | 3 | 0 | (0.0) |
| Headache | 10 | 6 | (60.0) | 6 | 4 | (66.7) | 10 | 7 | (70.0) | 10 | 7 | (70.0) | 10 | 6 | (60.0) | 7 | 5 | (71.4) | 9 | 2 | (22.2) | 10 | 6 | (60.0) |
| Myalgia | 11 | 5 | (45.5) | 8 | 3 | (37.5) | 9 | 5 | (55.6) | 5 | 2 | (40.0) | 4 | 3 | (75.0) | 6 | 0 | (0.0) | 7 | 3 | (42.9) | 7 | 4 | (57.1) |
| Nausea | 2 | 0 | (0.0) | 8 | 0 | (0.0) | 2 | 0 | (0.0) | 2 | 1 | (50.0) | 3 | 1 | (33.3) | 4 | 0 | (0.0) | 4 | 1 | (25.0) | 3 | 0 | (0.0) |
| Pyrexia | 1 | 1 | (100) | 2 | 2 | (100) | 0 | - | - | 0 | - | - | 1 | 0 | (0.0) | 0 | - | - | 1 | 1 | (100) | 0 | - | - |
| Vomiting | 0 | - | - | 2 | 0 | (0.0) | 0 | - | - | 0 | - | - | 1 | 0 | (0.0) | 1 | 0 | (0.0) | 0 | 0 | (0.0) | 0 | - | - |
| **TOTAL** | 107 | 20 | (18.7) | 109 | 15 | (13.8) | 86 | 15 | (17.4) | 72 | 14 | (19.4) | 76 | 14 | (18.4) | 62 | 6 | (9.7) | 59 | 12 | (20.3) | 51 | 11 | (21.6) |
| adj: adjuvante; AR=adverse reaction | | | | | | | | | | | | | | | | | | | | | | | | |

**S. Table 8.** Distribution of solicited AR, and medication use, occurring up to 28 days after the second vaccination, by study group.

| **Solicited Adverse Reactions** | **Group 1A** | | | **Group 1B** | | | **Group 1C** | | | **Group 2A** | | | **Group 2B** | | | **Group 2C** | | | **Group 3** | | | **Group 4** | | |
| --- | --- | --- | --- | --- | --- | --- | --- | --- | --- | --- | --- | --- | --- | --- | --- | --- | --- | --- | --- | --- | --- | --- | --- | --- |
|  | **15μg H7N9 + IB160** | | | **7.5μg H7N9 + IB160** | | | **3.75μg H7N9 + IB160** | | | **15μg H7N9 + SE** | | | **7.5μg H7N9 + SE** | | | **3.75μg H7N9 + SE** | | | **15μg H7N9, without adjuvant** | | | **Placebo** | | |
|  | **(n=53)** | | | **(n=53)** | | | **(n=54)** | | | **(n=53)** | | | **(n=55)** | | | **(n=54)** | | | **(n=54)** | | | **(n=54)** | | |
|  | **No.**  **AR**  **Total** | **AR with**  **medication** | | **No.**  **AR**  **Total** | **AR with**  **medication** | | **No.**  **AR**  **Total** | **AR with**  **medication** | | **No.**  **AR**  **Total** | **AR with**  **medication** | | **No.**  **AR**  **Total** | **AR with**  **medication** | | **No.**  **AR**  **Total** | **AR with**  **medication** | | **No.**  **AR**  **Total** | **AR with**  **medication** | | **No.**  **AR**  **Total** | **AR with**  **medication** | |
|  |  | **n** | **(%)** |  | **n** | **(%)** |  | **n** | **(%)** |  | **n** | **(%)** |  | **n** | **(%)** |  | **n** | **(%)** |  | **n** | **(%)** |  | **n** | **(%)** |
| **Local** |  |  |  |  |  |  |  |  |  |  |  |  |  |  |  |  |  |  |  |  |  |  |  |  |
| Bruise | 0 | - | - | 0 | - | - | 0 | - | - | 1 | 0 | (0.0) | 1 | 0 | (0.0) | 1 | 0 | (0.0) | 1 | 0 | (0.0) | 0 | - | - |
| Erythema | 2 | 0 | (0.0) | 1 | 0 | (0.0) | 1 | 0 | (0.0) | 0 | - | - | 1 | 0 | (0.0) | 1 | 0 | (0.0) | 1 | 0 | (0.0) | 0 | - | - |
| Induration | 1 | 0 | (0.0) | 0 | - | - | 1 | 0 | (0.0) | 1 | 0 | (0.0) | 2 | 0 | (0.0) | 2 | 0 | (0.0) | 0 | - | - | 1 | 0 | (0.0) |
| Pain | 29 | 1 | (3.4) | 32 | 1 | (3.1) | 30 | 0 | (0.0) | 19 | 1 | (5.3) | 20 | 1 | (5.0) | 22 | 1 | (0.0) | 11 | 0 | (0.0) | 8 | 0 | (0.0) |
| Pruritus | 0 | - | - | 1 | 0 | (0.0) | 1 | 0 | (0.0) | 0 | - | - | 1 | 0 | (0.0) | 1 | 0 | (0.0) | 3 | 0 | (0.0) | 0 | - | - |
| Swelling | 1 | 0 | (0.0) | 3 | 0 | (0.0) | 1 | 0 | (0.0) | 0 | - | - | 3 | 0 | (0.0) | 3 | 0 | (0.0) | 1 | 0 | (0.0) | 0 | - | - |
| Tenderness | 10 | 2 | (20.0) | 13 | 0 | (0.0) | 13 | 0 | (0.0) | 8 | 0 | (0.0) | 12 | 0 | (0.0) | 10 | 0 | (0.0) | 4 | 0 | (0.0) | 3 | 0 | (0.0) |
| **Systemic** |  |  |  |  |  |  |  |  |  |  |  |  |  |  |  |  |  |  |  |  |  |  |  |  |
| Arthralgia | 3 | 1 | (33.3) | 1 | 0 | (0.0) | 3 | 1 | (33.3) | 2 | 1 | (50.0) | 3 | 0 | (0.0) | 2 | 0 | (0.0) | 0 | - | - | 0 | - | - |
| Chills | 3 | 0 | (0.0) | 1 | 0 | (0.0) | 4 | 0 | (0.0) | 0 | - | - | 1 | 0 | (0.0) | 2 | 0 | (0.0) | 0 | - | - | 2 | 0 | (0.0) |
| Discomfort | 1 | 0 | (0.0) | 3 | 0 | (0.0) | 6 | 0 | (0.0) | 1 | 0 | (0.0) | 2 | 0 | (0.0) | 3 | 0 | (0.0) | 2 | 0 | (0.0) | 1 | 0 | (0.0) |
| Fatigue | 1 | 0 | (0.0) | 4 | 1 | (25.0) | 5 | 0 | (0.0) | 0 | - | - | 3 | 0 | (0.0) | 3 | 0 | (0.0) | 2 | 1 | (50.0) | 0 | - | - |
| Headache | 3 | 2 | (66.7) | 9 | 5 | (55.6) | 5 | 3 | (60.0) | 4 | 1 | (25.0) | 12 | 7 | (58.3) | 6 | 4 | (66.7) | 4 | 0 | (0.0) | 4 | 2 | (50.0) |
| Myalgia | 5 | 1 | (20.0) | 5 | 1 | (20.0) | 10 | 4 | (40.0) | 1 | 1 | (100) | 3 | 0 | (0.0) | 5 | 2 | (40.0) | 0 | - | - | 0 | - | - |
| Nausea | 1 | 0 | (0.0) | 0 | - | - | 3 | 1 | (33.3) | 2 | 0 | (0.0) | 2 | 0 | (0.0) | 3 | 1 | (33.3) | 3 | 1 | (33.3) | 2 | 0 | (0.0) |
| Pyrexia | 0 | - | - | 2 | 1 | (50.0) | 2 | 2 | (100) | 0 | - | - | 0 | - | - | 0 | - | - | 0 | - | - | 0 | - | - |
| Vomiting | 0 | - | - | 0 | - | - | 1 | 1 | (100) | 0 | - | - | 0 | - | - | 0 | - | - | 1 | 1 | (100) | 0 | - | - |
| **TOTAL** | 60 | 7 | (11.7) | 75 | 9 | (12.0) | 86 | 12 | (14.0) | 39 | 4 | (10.3) | 66 | 8 | (12.1) | 64 | 8 | (12.5) | 33 | 3 | (9.1) | 21 | 2 | (9.5) |
| adj: adjuvante; AR=adverse reaction | | | | | | | | | | | | | | | | | | | | | | | | |

**S. Table 9.** Distribution and duration of solicited AR occurring up to 28 days after the first vaccination, by study group.

| **Solicited Adverse Reactions** | **Group 1A** | | | **Group 1B** | | | **Group 1C** | | | **Group 2A** | | | **Group 2B** | | | **Group 2C** | | | **Group 3** | | | **Group 4** | | |
| --- | --- | --- | --- | --- | --- | --- | --- | --- | --- | --- | --- | --- | --- | --- | --- | --- | --- | --- | --- | --- | --- | --- | --- | --- |
|  | **15μg H7N9 + IB160** | | | **7.5μg H7N9 + IB160** | | | **3.75μg H7N9 + IB160** | | | **15μg H7N9 + SE** | | | **7.5μg H7N9 + SE** | | | **3.75μg H7N9 + SE** | | | **15μg H7N9, without adjuvant** | | | **Placebo** | | |
|  | **(n=53)** | | | **(n=53)** | | | **(n=54)** | | | **(n=53)** | | | **(n=55)** | | | **(n=54)** | | | **(n=54)** | | | **(n=54)** | | |
|  | **No.**  **AR**  **Total** | **AR Duration***  **median**  **(P25 - P75)** | | **No.**  **AR**  **Total** | **AR Duration***  **median**  **(P25 - P75)** | | **No.**  **AR**  **Total** | **AR Duration***  **median**  **(P25 - P75)** | | **No.**  **AR**  **Total** | **AR Duration***  **median**  **(P25 - P75)** | | **No.**  **AR**  **Total** | **AR Duration***  **median**  **(P25 - P75)** | | **No.**  **AR**  **Total** | **AR Duration***  **median**  **(P25 - P75)** | | **No.**  **AR**  **Total** | **AR Duration***  **median**  **(P25 - P75)** | | **No.**  **AR**  **Total** | **AR Duration***  **median**  **(P25 - P75)** | |
| **Local** |  |  |  |  |  |  |  |  |  |  |  |  |  |  |  |  |  |  |  |  |  |  |  |  |
| Bruise | 3 | 1 | (0 - 10) | 0 | - | - | 0 | - | - | 1 | 0 | (0 - 0) | 1 | 10 | (10 - 10) | 1 | 0 | (0 - 0) | 0 | - | - | 0 | - | - |
| Erythema | 2 | 1 | (0 - 2) | 3 | 0 | (0 - 0) | 1 | 1 | (1 - 1) | 0 | - | - | 1 | 0 | (0 - 0) | 2 | 2 | (1 - 3) | 0 | - | - | 1 | 0 | (0 - 0) |
| Induration | 1 | 0 | (0 - 0) | 3 | 1 | (1 - 2) | 0 | - | - | 0 | - | - | 2 | 0.5 | (0 - 1) | 2 | 2 | (2 - 2) | 0 | - | - | 0 | - | - |
| Pain | 41 | 1 | (0 - 2) | 33 | 1 | (0 - 2) | 41 | 1 | (0 - 1) | 28 | 1 | (0 - 1) | 32 | 1 | (0 - 2) | 24 | 1 | (0 - 2) | 24 | 1 | (0 - 1) | 12 | 0.5 | (0 - 1) |
| Pruritus | 0 | - | - | 6 | 0 | (0 - 0) | 1 | 2 | (2 - 2) | 1 | 0 | (0 - 0) | 1 | 1 | (1 - 1) | 2 | 1.5 | (1 - 2) | 0 | - | - | 2 | 0 | (0 - 0) |
| Swelling | 5 | 0 | (0 - 1) | 7 | 0 | (0 - 2) | 4 | 1 | (0.5 - 1) | 4 | 1 | (0.5 - 2) | 2 | 0.5 | (0 - 1) | 2 | 3 | (2 - 4) | 0 | - | - | 1 | 1 | (1 - 1) |
| Tenderness | 15 | 1 | (0 - 1) | 18 | 0 | (0 - 1) | 8 | 1 | (0.5 - 1.5) | 12 | 0.5 | (0 - 1) | 9 | 1 | (0 - 1) | 6 | 1 | (1 - 1) | 3 | 1 | (0 - 5) | 5 | 0 | (0 - 1) |
| **Systemic** |  |  |  |  |  |  |  |  |  |  |  |  |  |  |  |  |  |  |  |  |  |  |  |  |
| Arthralgia | 6 | 0 | (0 - 0) | 3 | 0 | (0 - 2) | 7 | 1 | (0 - 1) | 5 | 0 | (0 - 1) | 4 | 5.5 | (1 - 12.5) | 2 | 1 | (1 - 1) | 5 | 1 | (0 - 1) | 3 | 2 | (0 - 3) |
| Chills | 2 | 0 | (0 - 0) | 2 | 0 | (0 - 0) | 1 | 0 | (0 - 0) | 1 | 0 | (0 - 0) | 2 | 0 | (0 - 0) | 2 | 2 | (1 -3) | 2 | 1.5 | (1 - 2) | 1 | 1 | (1 - 1) |
| Discomfort | 4 | 0.5 | (0 - 1) | 3 | 0 | (0 - 2) | 1 | 1 | (1 - 1) | 2 | 0 | (0 - 0) | 2 | 5.5 | (2 - 9) | 1 | 1 | (1 - 1) | 2 | 0 | (0 - 0) | 3 | 0 | (0 - 1) |
| Fatigue | 4 | 0 | (0 - 0.5) | 5 | 0 | (0 - 1) | 1 | 1 | (1 - 1) | 1 | 2 | (2 - 2) | 1 | 10 | (10 - 10) | 0 | - | - | 2 | 0.5 | (0 - 1) | 3 | 1 | (0 - 2) |
| Headache | 10 | 0 | (0 - 1) | 6 | 1 | (0 - 2) | 10 | 1 | (0 - 5) | 10 | 0 | (0 - 1) | 10 | 0 | (0 - 1) | 7 | 0 | (0 - 0) | 9 | 0 | (0 - 1) | 10 | 0 | (0 - 1) |
| Myalgia | 11 | 1 | (0 - 5) | 8 | 0 | (0 - 1) | 9 | 1 | (1 - 1) | 5 | 0 | (0 - 0) | 4 | 2.5 | (1.5 - 3.5) | 6 | 1.5 | (1 - 2) | 7 | 1 | (0 - 2) | 7 | 0 | (0 - 3) |
| Nausea | 2 | 9 | (0 - 18) | 8 | 0 | (0 - 1) | 2 | 0 | (0 - 0) | 2 | 0.5 | (0 - 1) | 3 | 0 | (0 - 10) | 4 | 0.5 | (0 - 3) | 4 | 0 | (0 - 1.5) | 3 | 1 | (0 1) |
| Pyrexia | 1 | 1 | (1 - 1) | 2 | 0 | (0 - 0) | 0 | - | - | 0 | - | - | 1 | 4 | (4 - 4) | 0 | - | - | 1 | 0 | (0 - 0) | 0 | - | - |
| Vomiting | 0 | - | - | 2 | 0.5 | (0 - 1) | 0 | - | - | 0 | - | - | 1 | 4 | (4 - 4) | 1 | 0 | (0 - 0) | 0 | - | - | 0 | - | - |
| **TOTAL** | 107 | 1 | (0 - 1) | 109 | 0 | (0 - 1) | 86 | 1 | (0 - 1) | 72 | 1 | (0 - 1) | 76 | 1 | (0 - 2) | 62 | 1 | (0 - 2) | 59 | 1 | (0 - 1) | 51 | 0 | (0 - 1) |
| * in days; adj: adjuvant; AR=adverse reaction; P25: first quartile; P75: third quartile | | | | | | | | | | | | | | | | | | | | | | | | |

**S. Table 10.** Distribution and duration of solicited AR occurring up to 28 days after the second vaccination, by study group.

| **Solicited Adverse Reactions** | **Group 1A** | | | **Group 1B** | | | **Group 1C** | | | **Group 2A** | | | **Group 2B** | | | **Group 2C** | | | **Group 3** | | | **Group 4** | | |
| --- | --- | --- | --- | --- | --- | --- | --- | --- | --- | --- | --- | --- | --- | --- | --- | --- | --- | --- | --- | --- | --- | --- | --- | --- |
|  | **15μg H7N9 + IB160** | | | **7.5μg H7N9 + IB160** | | | **3.75μg H7N9 + IB160** | | | **15μg H7N9 + SE** | | | **7.5μg H7N9 + SE** | | | **3.75μg H7N9 + SE** | | | **15μg H7N9, without adjuvant** | | | **Placebo** | | |
|  | **(n=53)** | | | **(n=53)** | | | **(n=54)** | | | **(n=53)** | | | **(n=55)** | | | **(n=54)** | | | **(n=54)** | | | **(n=54)** | | |
|  | **No.**  **AR**  **Total** | **AR Duration***  **median**  **(P25 - P75)** | | **No.**  **AR**  **Total** | **AR Duration***  **median**  **(P25 - P75)** | | **No.**  **AR**  **Total** | **AR Duration***  **median**  **(P25 - P75)** | | **No.**  **AR**  **Total** | **AR Duration***  **median**  **(P25 - P75)** | | **No.**  **AR**  **Total** | **AR Duration***  **median**  **(P25 - P75)** | | **No.**  **AR**  **Total** | **AR Duration***  **median**  **(P25 - P75)** | | **No.**  **AR**  **Total** | **AR Duration***  **median**  **(P25 - P75)** | | **No.**  **AR**  **Total** | **AR Duration***  **median**  **(P25 - P75)** | |
| **Local** |  |  |  |  |  |  |  |  |  |  |  |  |  |  |  |  |  |  |  |  |  |  |  |  |
| Bruise | 0 | - | - | 0 | - | - | 0 | - | - | 1 | 0 | (0 - 0) | 1 | 1 | (1 - 1) | 1 | 1 | (1 - 1) | 1 | 2 | (2 - 2) | 0 | - | - |
| Erythema | 2 | 0.5 | (0 - 1) | 1 | 0 | (0 - 0) | 1 | 0 | (0 - 0) | 0 | - | - | 1 | 0 | (0 - 0) | 1 | 1 | (1 - 1) | 1 | 0 | (0 - 0) | 0 | - | - |
| Induration | 1 | 1 | (1 - 1) | 0 | - | - | 1 | 1 | (1 - 1) | 1 | 1 | (1 - 1) | 2 | 2.5 | (2 - 3) | 2 | 0.5 | (0 - 1) | 0 | - | - | 1 | 0 | (0 - 0) |
| Pain | 29 | 1 | (0 - 1) | 32 | 1 | (0.5 - 2) | 30 | 1 | (0 - 1) | 19 | 1 | (0 - 1) | 20 | 1 | (0 - 1) | 22 | 0 | (0 - 2) | 11 | 1 | (0 - 1) | 8 | 0.5 | (0 - 1) |
| Pruritus | 0 | - | - | 1 | 0 | (0 - 0) | 1 | 0 | (0 - 0) | 0 | - | - | 1 | 0 | (0 - 0) | 1 | 1 | (1 - 1) | 3 | 1 | (0 - 1) | 0 | - | - |
| Swelling | 1 | 0 | (0 - 0) | 3 | 1 | (0 - 2) | 1 | 0 | (0 - 0) | 0 | - | - | 3 | 2 | (1 - 3) | 3 | 1 | (0 - 1) | 1 | 0 | (0 - 0) | 0 | - | - |
| Tenderness | 10 | 1 | (1 - 1) | 13 | 1 | (0 - 1) | 13 | 1 | (1 - 1) | 8 | 0.5 | (0 - 1.5) | 12 | 1 | (0 - 1) | 10 | 0.5 | (0 - 1) | 4 | 0.5 | (0 - 1) | 3 | 1 | (1 - 1) |
| **Systemic** |  |  |  |  |  |  |  |  |  |  |  |  |  |  |  |  |  |  |  |  |  |  |  |  |
| Arthralgia | 3 | 0 | (0 - 1) | 1 | 0 | (0 - 0) | 3 | 2 | (0 - 5) | 2 | 0.5 | (0 - 1) | 3 | 1 | (0 - 7) | 2 | 0 | (0 - 0) | 0 | - | - | 0 | - | - |
| Chills | 3 | 0 | (0 - 1) | 1 | 3 | (3 - 3) | 4 | 1.5 | (0.5 - 3.5) | 0 | - | - | 1 | 0 | (0 - 0) | 2 | 0.5 | (0 - 1) | 0 | - | - | 2 | 0.5 | (0 - 1) |
| Discomfort | 1 | 1 | (1 - 1) | 3 | 1 | (0 - 3) | 6 | 0 | (0 - 1) | 1 | 0 | (0 - 0) | 2 | 1 | (1 - 1) | 3 | 0 | (0 - 0) | 2 | 0.5 | (0 - 1) | 1 | 1 | (1 - 1) |
| Fatigue | 1 | 0 | (0 - 0) | 4 | 1 | (0.5 - 3) | 5 | 1 | (0 - 2) | 0 | - | - | 3 | 2 | (1 - 3) | 3 | 0 | (0 - 0) | 2 | 0.5 | (0 - 1) | 0 | - | - |
| Headache | 3 | 0 | (0 - 5) | 9 | 1 | (1 - 4) | 5 | 1 | (0 - 2) | 4 | 0 | (0 - 0.5) | 12 | 1 | (0 - 2) | 6 | 0 | (0 - 0) | 4 | 0.5 | (0 - 1) | 4 | 3.5 | (2 - 4) |
| Myalgia | 5 | 1 | (1 - 1) | 5 | 1 | (0 - 4) | 10 | 2 | (0 - 4) | 1 | 0 | (0 - 0) | 3 | 1 | (0 - 2) | 5 | 0 | (0 - 0) | 0 | - | - | 0 | - | - |
| Nausea | 1 | 0 | (0 - 0) | 0 | - | - | 3 | 0 | (0 - 1) | 2 | 0 | (0 - 0) | 2 | 0.5 | (0 - 1) | 3 | 0 | (0 - 1) | 3 | 0 | (0 - 13) | 2 | 1 | (0 - 2) |
| Pyrexia | 0 | - | - | 2 | 1.5 | (0 - 3) | 2 | 0.5 | (0 - 1) | 0 | - | - | 0 | - | - | 0 | - | - | 0 | - | - | 0 | - | - |
| Vomiting | 0 | - | - | 0 | - | - | 1 | 1 | (1 - 1) | 0 | - | - | 0 | - | - | 0 | - | - | 1 | 11 | (11 - 11) | 0 | - | - |
| **TOTAL** | 60 | 1 | (0 - 1) | 75 | 1 | (0 - 2) | 86 | 1 | (0 - 2) | 39 | 0 | (0 - 1) | 66 | 1 | (0 - 2) | 64 | 0 | (0 - 1) | 33 | 1 | (0 - 11) | 21 | 1 | (0 - 1) |
| * in days; adj: adjuvant; AR=adverse reaction; P25: first quartile; P75: third quartile | | | | | | | | | | | | | | | | | | | | | | | | |

**S. Table 11.** Distribution and start day of solicited AR occurring up to 28 after the first vaccination, by study group.

| **Solicited Adverse Reactions** | **Group 1A** | | | **Group 1B** | | | **Group 1C** | | | **Group 2A** | | | **Group 2B** | | | **Group 2C** | | | **Group 3** | | | **Group 4** | | |
| --- | --- | --- | --- | --- | --- | --- | --- | --- | --- | --- | --- | --- | --- | --- | --- | --- | --- | --- | --- | --- | --- | --- | --- | --- |
|  | **15μg H7N9 + IB160** | | | **7.5μg H7N9 + IB160** | | | **3.75μg H7N9 + IB160** | | | **15μg H7N9 + SE** | | | **7.5μg H7N9 + SE** | | | **3.75μg H7N9 + SE** | | | **15μg H7N9, without adjuvant** | | | **Placebo** | | |
|  | **(n=53)** | | | **(n=53)** | | | **(n=54)** | | | **(n=53)** | | | **(n=55)** | | | **(n=54)** | | | **(n=54)** | | | **(n=54)** | | |
|  | **No.**  **AR**  **Total** | **AR start day***  **median**  **(P_25_ - P_75_)** | | **No.**  **AR**  **Total** | **AR start day***  **median**  **(P_25_ - P_75_)** | | **No.**  **AR**  **Total** | **AR start day***  **median**  **(P_25_ - P_75_)** | | **No.**  **AR**  **Total** | **AR start day***  **median**  **(P_25_ - P_75_)** | | **No.**  **AR**  **Total** | **AR start day***  **median**  **(P_25_ - P_75_)** | | **No.**  **AR**  **Total** | **AR start day***  **median**  **(P_25_ - P_75_)** | | **No.**  **AR**  **Total** | **AR start day***  **median**  **(P_25_ - P_75_)** | | **No.**  **AR**  **Total** | **AR start day***  **median**  **(P_25_ - P_75_)** | |
| **Local** |  |  |  |  |  |  |  |  |  |  |  |  |  |  |  |  |  |  |  |  |  |  |  |  |
| Bruise | 3 | 1 | (0 - 2) | 0 | - | - | 0 | - | - | 1 | 1 | (1 - 1) | 1 | 1 | (1 - 1) | 1 | 1 | (1 - 1) | 0 | - | - | 0 | - | - |
| Erythema | 2 | 0 | (0 - 0) | 3 | 3 | (2 - 4) | 1 | 0 | (0 - 0) | 0 | - | - | 1 | 0 | (0 - 0) | 2 | 0 | (0 - 0) | 0 | - | - | 1 | 0 | (0 - 0) |
| Induration | 1 | 1 | (1 - 1) | 3 | 0 | (0 - 1) | 0 | - | - | 0 | - | - | 2 | 1 | (1 - 1) | 2 | 1 | (1 - 1) | 0 | - | - | 0 | - | - |
| Pain | 41 | 0 | (0 - 0) | 33 | 0 | (0 - 1) | 41 | 0 | (0 - 0) | 28 | 0 | (0 - 1) | 32 | 0 | (0 - 1) | 24 | 0 | (0 - 0.5) | 24 | 0 | (0 - 0) | 12 | 0 | (0 - 0) |
| Pruritus | 0 | - | - | 6 | 1 | (0 - 3) | 1 | 0 | (0 - 0) | 1 | 2 | (2 - 2) | 1 | 0 | (0 - 0) | 2 | 2 | (1 - 3) | 0 | - | - | 2 | 0.5 | (0 - 1) |
| Swelling | 5 | 0 | (0 - 0) | 7 | 0 | (0 - 1) | 4 | 1 | (0.5 - 1) | 4 | 0.5 | (0 - 1) | 2 | 1 | (1 - 1) | 2 | 0 | (0 - 0) | 0 | - | - | 1 | 0 | (0 - 0) |
| Tenderness | 15 | 0 | (0 - 1) | 18 | 0 | (0 - 1) | 8 | 0.5 | (0 - 1) | 12 | 0 | (0 - 1) | 9 | 0 | (0 - 1) | 6 | 0 | (0 - 0) | 3 | 0 | (0 - 0) | 5 | 0 | (0 - 0) |
| **Systemic** |  |  |  |  |  |  |  |  |  |  |  |  |  |  |  |  |  |  |  |  |  |  |  |  |
| Arthralgia | 6 | 1 | (1 - 1) | 3 | 0 | (0 - 3) | 7 | 2 | (1 - 5) | 5 | 2 | (1 - 2) | 4 | 0.5 | (0 - 2) | 2 | 1.5 | (0 - 3) | 5 | 1 | (1 - 2) | 3 | 1 | (1 - 1) |
| Chills | 2 | 0.5 | (0 - 1) | 2 | 2 | (1 - 3) | 1 | 1 | (1 - 1) | 1 | 4 | (4 - 4) | 2 | 0 | (0 - 0) | 2 | 0.5 | (0 - 1) | 2 | 2.5 | (2 - 3) | 1 | 0 | (0 - 0) |
| Discomfort | 4 | 0.5 | (0 - 1) | 3 | 2 | (0 - 3) | 1 | 1 | (1 - 1) | 2 | 2.5 | (1 - 4) | 2 | 0.5 | (0 - 1) | 1 | 4 | (4 - 4) | 2 | 1 | (1 - 1) | 3 | 0 | (0 - 0) |
| Fatigue | 4 | 2.5 | (0 - 5) | 5 | 1 | (0 - 2) | 1 | 1 | (1 - 1) | 1 | 0 | (0 - 0) | 1 | 0 | (0 - 0) | 0 | - | - | 2 | 0.5 | (0 - 1) | 3 | 2 | (0 - 3) |
| Headache | 10 | 1 | (0 - 3) | 6 | 1 | (0 - 2) | 10 | 3 | (1 - 4) | 10 | 1 | (0 - 5) | 10 | 2 | (0 - 3) | 7 | 4 | (1 - 5) | 9 | 3 | (1 - 4) | 10 | 1.5 | (1 - 4) |
| Myalgia | 11 | 1 | (0 - 6) | 8 | 1 | (0 - 1.5) | 9 | 1 | (1 - 2) | 5 | 2 | (1 - 3) | 4 | 4.5 | (1.5 - 6) | 6 | 1.5 | (1 - 2) | 7 | 1 | (0 - 1) | 7 | 1 | (0 - 4) |
| Nausea | 2 | 3 | (0 - 6) | 8 | 1.5 | (0.5 - 4.5) | 2 | 0.5 | (0 - 1) | 2 | 2.5 | (0 - 5) | 3 | 1 | (0.5 - 2) | 4 | 1 | (0.5 - 2) | 4 | 2.5 | (1 - 3) | 3 | 0 | (0 - 4) |
| Pyrexia | 1 | 1 | (1 - 1) | 2 | 2 | (1 - 3) | 0 | - | - | 0 | - | - | 1 | 6 | (6 - 6) | 0 | - | - | 1 | 3 | (3 - 3) | 0 | - | - |
| Vomiting | 0 | - | - | 2 | 2.5 | (0 - 5) | 0 | - | - | 0 | - | - | 1 | 6 | (6 - 6) | 1 | 1 | (1 - 1) | 0 | - | - | 0 | - | - |
| **TOTAL** | 107 | 0 | (0 - 1) | 109 | 0 | (0 - 1) | 86 | 0 | (0 - 1) | 72 | 0.5 | (0 - 2) | 76 | 0 | (0 - 1) | 62 | 0 | (0 - 1) | 59 | 0 | (0 - 2) | 51 | 0 | (0 - 2) |
| * day the AR started since vaccination day; adj: adjuvant; AR=adverse reaction; P_25_: first quartile; P_75_: third quartile | | | | | | | | | | | | | | | | | | | | | | | | |

**S. Table 12.** Distribution and start day of solicited AR occurring up to 28 days post second dose administration by study group.

| **Solicited Adverse Reactions** | **Group 1A** | | | **Group 1B** | | | **Group 1C** | | | **Group 2A** | | | **Group 2B** | | | **Group 2C** | | | **Group 3** | | | **Group 4** | | |
| --- | --- | --- | --- | --- | --- | --- | --- | --- | --- | --- | --- | --- | --- | --- | --- | --- | --- | --- | --- | --- | --- | --- | --- | --- |
|  | **15μg H7N9 + IB160** | | | **7.5μg H7N9 + IB160** | | | **3.75μg H7N9 + IB160** | | | **15μg H7N9 + SE** | | | **7.5μg H7N9 + SE** | | | **3.75μg H7N9 + SE** | | | **15μg H7N9, without adjuvant** | | | **Placebo** | | |
|  | **(n=53)** | | | **(n=53)** | | | **(n=54)** | | | **(n=53)** | | | **(n=55)** | | | **(n=54)** | | | **(n=54)** | | | **(n=54)** | | |
|  | **No.**  **AR**  **Total** | **AR start day***  **median**  **(P_25_ - P_75_)** | | **No.**  **AR**  **Total** | **AR start day***  **median**  **(P_25_ - P_75_)** | | **No.**  **AR**  **Total** | **AR start day***  **median**  **(P_25_ - P_75_)** | | **No.**  **AR**  **Total** | **AR start day***  **median**  **(P_25_ - P_75_)** | | **No.**  **AR**  **Total** | **AR start day***  **median**  **(P_25_ - P_75_)** | | **No.**  **AR**  **Total** | **AR start day***  **median**  **(P_25_ - P_75_)** | | **No.**  **AR**  **Total** | **AR start day***  **median**  **(P_25_ - P_75_)** | | **No.**  **AR**  **Total** | **AR start day***  **median**  **(P_25_ - P_75_)** | |
| **Local** |  |  |  |  |  |  |  |  |  |  |  |  |  |  |  |  |  |  |  |  |  |  |  |  |
| Bruise | 0 | - | - | 0 | - | - | 0 | - | - | 1 | 0 | (0 - 0) | 1 | 1 | (1 - 1) | 1 | 0 | (0 - 0) | 1 | 1 | (1 - 1) | 0 | - | - |
| Erythema | 2 | 0 | (0 - 0) | 1 | 0 | (0 - 0) | 1 | 0 | (0 - 0) | 0 | - | - | 1 | 1 | (1 - 1) | 1 | 0 | (0 - 0) | 1 | 1 | (1 - 1) | 0 | - | - |
| Induration | 1 | 0 | (0 - 0) | 0 | - | - | 1 | 0 | (0 - 0) | 1 | 0 | (0 - 0) | 2 | 0.5 | (0 - 1) | 2 | 0 | (0 - 0) | 0 | - | - | 1 | 0 | (0 - 0) |
| Pain | 29 | 0 | (0 - 0) | 32 | 0 | (0 - 0) | 30 | 0 | (0 - 0) | 19 | 0 | (0 - 0) | 20 | 0 | (0 - 0) | 22 | 0 | (0 - 1) | 11 | 0 | (0 - 0) | 8 | 0 | (0 - 0) |
| Pruritus | 0 | - | - | 1 | 0 | (0 - 0) | 1 | 1 | (1 - 1) | 0 | - | - | 1 | 0 | (0 - 0) | 1 | 0 | (0 - 0) | 3 | 0 | (0 - 1) | 0 | - | - |
| Swelling | 1 | 0 | (0 - 0) | 3 | 0 | (0 - 0) | 1 | 1 | (1 - 1) | 0 | - | - | 3 | 0 | (0 - 0) | 3 | 0 | (0 - 1) | 1 | 0 | (0 - 0) | 0 | - | - |
| Tenderness | 10 | 0 | (0 - 0) | 13 | 0 | (0 - 0) | 13 | 0 | (0 - 0) | 8 | 0 | (0 - 0) | 12 | 0 | (0 - 0.5) | 10 | 0 | (0 - 1) | 4 | 0.5 | (0 - 1) | 3 | 0 | (0 - 2) |
| **Systemic** |  |  |  |  |  |  |  |  |  |  |  |  |  |  |  |  |  |  |  |  |  |  |  |  |
| Arthralgia | 3 | 1 | (0 - 6) | 1 | 0 | (0 - 0) | 3 | 1 | (1 - 1) | 2 | 3 | (0 - 3) | 3 | 1 | (0 - 3) | 2 | 5.5 | (4 - 7) | 0 | - | - | 0 | - | - |
| Chills | 3 | 0 | (0 - 1) | 1 | 1 | (1 - 1) | 4 | 0.5 | (0 - 2) | 0 | - | - | 1 | 0 | (0 - 0) | 2 | 0.5 | (0 - 1) | 0 | - | - | 2 | 2 | (0 - 4) |
| Discomfort | 1 | 0 | (0 - 0) | 3 | 0 | (0 - 2) | 6 | 1 | (1 - 1) | 1 | 1 | (1 - 1) | 2 | 2.5 | (2 - 3) | 3 | 1 | (0 - 2) | 2 | 4.5 | (3 - 6) | 1 | 5 | (5 - 5) |
| Fatigue | 1 | 0 | (0 - 0) | 4 | 0 | (0 - 1) | 5 | 1 | (1 - 1) | 0 | - | - | 3 | 3 | (1 - 3) | 3 | 1 | (1 - 2) | 2 | 2.5 | (1 - 4) | 0 | - | - |
| Headache | 3 | 1 | (0 - 3) | 9 | 0 | (0 - 1) | 5 | 1 | (0 - 1) | 4 | 0.5 | (0 - 2.5) | 12 | 1.5 | (1 - 3) | 6 | 1 | (0 - 2) | 4 | 3 | (1.5 - 3.5) | 4 | 0 | (0 - 2.5) |
| Myalgia | 5 | 0 | (0 - 0) | 5 | 0 | (0 - 1) | 10 | 1 | (1 - 3) | 1 | 0 | (0 - 0) | 3 | 1 | (1 - 3) | 5 | 2 | (1 - 4) | 0 | - | - | 0 | - | - |
| Nausea | 1 | 0 | (0 - 0 | 0 | - | - | 3 | 1 | (0 - 3) | 2 | 3.5 | (1 - 6) | 2 | 1.5 | (1 - 2) | 3 | 3 | (0 - 4) | 3 | 2 | (0 - 6) | 2 | 2 | (0 - 4) |
| Pyrexia | 0 | - | - | 2 | 2 | (2 - 2) | 2 | 1 | (0 - 2) | 0 | - | - | 0 | - | - | 0 | - | - | 0 | - | - | 0 | - | - |
| Vomiting | 0 | - | - | 0 | - | - | 1 | 0 | (0 - 0) | 0 | - | - | 0 | - | - | 0 | - | - | 1 | 4 | (4 - 4) | 0 | - | - |
| **TOTAL** | 60 | 0 | (0 - 0) | 75 | 0 | (0 - 0) | 86 | 0 | (0 - 1) | 39 | 0 | (0 - 1) | 66 | 0 | (0 - 1) | 64 | 0 | (0 - 1) | 33 | 0 | (0 - 2) | 21 | 0 | (0 - 0) |
| * in days; adj: adjuvant; AR=adverse reaction; P_25_: first quartile; P_75_: third quartile | | | | | | | | | | | | | | | | | | | | | | | | |

**S. Table 13.** Proportion of participants with unsolicited adverse reactions occurring within 30 minutes after the first vaccination, by study group.

| **Unsolicited Adverse Reactions** | **Group 1A** | | | **Group 1B** | | | **Group 1C** | | | **Group 2A** | | | **Group 2B** | | | **Group 2C** | | | **Group 3** | | | **Group 4** | | |
| --- | --- | --- | --- | --- | --- | --- | --- | --- | --- | --- | --- | --- | --- | --- | --- | --- | --- | --- | --- | --- | --- | --- | --- | --- |
|  | **15μg H7N9 + IB160** | | | **7.5μg H7N9 + IB160** | | | **3.75μg H7N9 + IB160** | | | **15μg H7N9 + SE** | | | **7.5μg H7N9 + SE** | | | **3.75μg H7N9 + SE** | | | **15μg H7N9, without adj** | | | **Placebo** | | |
|  | **(n=53)** | | | **(n=53)** | | | **(n=54)** | | | **(n=53)** | | | **(n=55)** | | | **(n=54)** | | | **(n=54)** | | | **(n=54)** | | |
|  | **n** | **%** | **(95%CI)** | **n** | **%** | **(95%CI)** | **n** | **%** | **(95%CI)** | **n** | **%** | **(95%CI)** | **n** | **%** | **(95%CI)** | **n** | **%** | **(95%CI)** | **n** | **%** | **(95%CI)** | **n** | **%** | **(95%CI)** |
| Administration site paraesthesia | 0 | 0.0 | (0.0 - 6.7) | 0 | 0.0 | (0.0 - 6.7) | 0 | 0.0 | (0.0 - 6.6) | 0 | 0.0 | (0.0 - 6.7) | 0 | 0.0 | (0.0 - 6.5) | 0 | 0.0 | (0.0 - 6.6) | 1 | 1.9 | (0.0 - 9.9) | 0 | 0.0 | (0.0 - 6.6) |
| Dizziness | 0 | 0.0 | (0.0 - 6.7) | 0 | 0.0 | (0.0 - 6.7) | 0 | 0.0 | (0.0 - 6.6) | 0 | 0.0 | (0.0 - 6.7) | 0 | 0.0 | (0.0 - 6.5) | 1 | 1.9 | (0.0 - 9.9) | 0 | 0.0 | (0.0 - 6.6) | 0 | 0.0 | (0.0 - 6.6) |
| Dry throat | 0 | 0.0 | (0.0 - 6.7) | 0 | 0.0 | (0.0 - 6.7) | 0 | 0.0 | (0.0 - 6.6) | 0 | 0.0 | (0.0 - 6.7) | 1 | 1.8 | (0.0 - 9.7) | 0 | 0.0 | (0.0 - 6.6) | 0 | 0.0 | (0.0 - 6.6) | 0 | 0.0 | (0.0 - 6.6) |
| Muscle spasms | 0 | 0.0 | (0.0 - 6.7) | 0 | 0.0 | (0.0 - 6.7) | 0 | 0.0 | (0.0 - 6.6) | 0 | 0.0 | (0.0 - 6.7) | 1 | 1.8 | (0.0 - 9.7) | 0 | 0.0 | (0.0 - 6.6) | 0 | 0.0 | (0.0 - 6.6) | 0 | 0.0 | (0.0 - 6.6) |
| Paraesthesia | 0 | 0.0 | (0.0 - 6.7) | 0 | 0.0 | (0.0 - 6.7) | 0 | 0.0 | (0.0 - 6.6) | 0 | 0.0 | (0.0 - 6.7) | 0 | 0.0 | (0.0 - 6.5) | 0 | 0.0 | (0.0 - 6.6) | 1 | 1.9 | (0.0 - 9.9) | 0 | 0.0 | (0.0 - 6.6) |
| Pruritus | 0 | 0.0 | (0.0 - 6.7) | 0 | 0.0 | (0.0 - 6.7) | 0 | 0.0 | (0.0 - 6.6) | 0 | 0.0 | (0.0 - 6.7) | 0 | 0.0 | (0.0 - 6.5) | 0 | 0.0 | (0.0 - 6.6) | 1 | 1.9 | (0.0 - 9.9) | 0 | 0.0 | (0.0 - 6.6) |
| Somnolence | 0 | 0.0 | (0.0 - 6.7) | 0 | 0.0 | (0.0 - 6.7) | 0 | 0.0 | (0.0 - 6.6) | 0 | 0.0 | (0.0 - 6.7) | 0 | 0.0 | (0.0 - 6.5) | 0 | 0.0 | (0.0 - 6.6) | 0 | 0.0 | (0.0 - 6.6) | 2 | 3.7 | (0.5 - 12.7) |
| Others | 1 | 1.9 | (0.0 - 10.1) | 0 | 0.0 | (0.0 - 6.7) | 0 | 0.0 | (0.0 - 6.6) | 0 | 0.0 | (0.0 - 6.7) | 0 | 0.0 | (0.0 - 6.5) | 0 | 0.0 | (0.0 - 6.6) | 0 | 0.0 | (0.0 - 6.6) | 1 | 1.9 | (0.0 - 9.9) |
| **TOTAL** | 1 | 1.9 | (0.0 - 10.1) | 0 | 0.0 | (0.0 - 6.7) | 0 | 0.0 | (0.0 - 6.6) | 0 | 0.0 | (0.0 - 6.7) | 2 | 3.6 | (0.4 - 12.5) | 1 | 1.9 | (0.0 - 9.9) | 2 | 3.7 | (0.5 - 12.7) | 2 | 3.7 | (0.5 - 12.7) |
| adj: adjuvant; 95%CI: 95% confidence interval | | | | | | | | | | | | | | | | | | | | | | | | |

**S. Table 14.** Proportion of participants with unsolicited adverse reactions occurring within 30 minutes after the second vaccination, by study group.

| **Unsolicited Adverse Reactions** | **Group 1A** | | | **Group 1B** | | | **Group 1C** | | | **Group 2A** | | | **Group 2B** | | | **Group 2C** | | | **Group 3** | | | **Group 4** | | |
| --- | --- | --- | --- | --- | --- | --- | --- | --- | --- | --- | --- | --- | --- | --- | --- | --- | --- | --- | --- | --- | --- | --- | --- | --- |
|  | **15μg H7N9 + IB160** | | | **7.5μg H7N9 + IB160** | | | **3.75μg H7N9 + IB160** | | | **15μg H7N9 + SE** | | | **7.5μg H7N9 + SE** | | | **3.75μg H7N9 + SE** | | | **15μg H7N9, without adj** | | | **Placebo** | | |
|  | **(n=53)** | | | **(n=53)** | | | **(n=54)** | | | **(n=53)** | | | **(n=55)** | | | **(n=54)** | | | **(n=54)** | | | **(n=54)** | | |
|  | **n** | **%** | **(95%CI)** | **n** | **%** | **(95%CI)** | **n** | **%** | **(95%CI)** | **n** | **%** | **(95%CI)** | **n** | **%** | **(95%CI)** | **n** | **%** | **(95%CI)** | **n** | **%** | **(95%CI)** | **n** | **%** | **(95%CI)** |
| Administration site haemorrhage | 1 | 2.0 | (0.1 - 10.6) | 0 | 0.0 | (0.0 - 7.4) | 0 | 0.0 | (0.0 - 7.0) | 0 | 0.0 | (0.0 - 7.4) | 0 | 0.0 | (0.0 - 6.7) | 0 | 0.0 | (0.0 - 7.0) | 0 | 0.0 | (0.0 - 7.0) | 1 | 2.1 | (0.1 - 11.1) |
| Administration site paraesthesia | 1 | 2.0 | (0.1 - 10.6) | 0 | 0.0 | (0.0 - 7.4) | 0 | 0.0 | (0.0 - 7.0) | 0 | 0.0 | (0.0 - 7.4) | 0 | 0.0 | (0.0 - 6.7) | 0 | 0.0 | (0.0 - 7.0) | 0 | 0.0 | (0.0 - 7.0) | 0 | 0.0 | (0.0 - 7.4) |
| Dizziness | 0 | 0.0 | (0.0 - 7.1) | 0 | 0.0 | (0.0 - 7.4) | 0 | 0.0 | (0.0 - 7.0) | 1 | 2.1 | (0.1 - 11.1) | 1 | 1.9 | (0.0 - 10.1) | 0 | 0.0 | (0.0 - 7.0) | 0 | 0.0 | (0.0 - 7.0) | 0 | 0.0 | (0.0 - 7.4) |
| Dry throat | 0 | 0.0 | (0.0 - 7.1) | 0 | 0.0 | (0.0 - 7.4) | 0 | 0.0 | (0.0 - 7.0) | 0 | 0.0 | (0.0 - 7.4) | 1 | 1.9 | (0.0 - 10.1) | 0 | 0.0 | (0.0 - 7.0) | 0 | 0.0 | (0.0 - 7.0) | 0 | 0.0 | (0.0 - 7.4) |
| Paraesthesia | 1 | 2.0 | (0.1 - 10.6) | 0 | 0.0 | (0.0 - 7.4) | 0 | 0.0 | (0.0 - 7.0) | 0 | 0.0 | (0.0 - 7.4) | 0 | 0.0 | (0.0 - 6.7) | 0 | 0.0 | (0.0 - 7.0) | 0 | 0.0 | (0.0 - 7.0) | 0 | 0.0 | (0.0 - 7.4) |
| Transaminases increased | 0 | 0.0 | (0.0 - 7.1) | 0 | 0.0 | (0.0 - 7.4) | 0 | 0.0 | (0.0 - 7.0) | 0 | 0.0 | (0.0 - 7.4) | 1 | 1.9 | (0.0 - 10.1) | 0 | 0.0 | (0.0 - 7.0) | 0 | 0.0 | (0.0 - 7.0) | 1 | 2.1 | (0.1 - 11.1) |
| Others | 0 | 0.0 | (0.0 - 7.1) | 1 | 2.1 | (0.1 - 11.1) | 0 | 0.0 | (0.0 - 7.0) | 0 | 0.0 | (0.0 - 7.4) | 1 | 1.9 | (0.0 - 10.1) | 0 | 0.0 | (0.0 - 7.0) | 1 | 2.0 | (0.0 - 10.4) | 0 | 0.0 | (0.0 - 7.4) |
| **TOTAL** | 1 | 2.0 | (0.1 - 10.6) | 1 | 2.1 | (0.1 - 11.1) | 0 | 0.0 | (0.0 - 7.0) | 1 | 2.1 | (0.1 - 11.1) | 2 | 3.8 | (0.5 - 13) | 0 | 0.0 | (0.0 - 7.0) | 1 | 2.0 | (0.0 - 10.4) | 2 | 4.2 | (0.5 - 14.3) |
| adj: adjuvant; 95%CI: 95% confidence interval | | | | | | | | | | | | | | | | | | | | | | | | |

**S. Table 15.** Proportion of participants with unsolicited adverse reactions occurring 7 days after the first vaccination, by study group.

| **Unsolicited Adverse Reactions** | **Group 1A** | | | **Group 1B** | | | **Group 1C** | | | **Group 2A** | | | **Group 2B** | | | **Group 2C** | | | **Group 3** | | | **Group 4** | | |
| --- | --- | --- | --- | --- | --- | --- | --- | --- | --- | --- | --- | --- | --- | --- | --- | --- | --- | --- | --- | --- | --- | --- | --- | --- |
|  | **15μg H7N9 + IB160** | | | **7.5μg H7N9 + IB160** | | | **3.75μg H7N9 + IB160** | | | **15μg H7N9 + SE** | | | **7.5μg H7N9 + SE** | | | **3.75μg H7N9 + SE** | | | **15μg H7N9, without adj** | | | **Placebo** | | |
|  | **(n=53)** | | | **(n=53)** | | | **(n=54)** | | | **(n=53)** | | | **(n=55)** | | | **(n=54)** | | | **(n=54)** | | | **(n=54)** | | |
|  | **n** | **%** | **(95%CI)** | **n** | **%** | **(95%CI)** | **n** | **%** | **(95%CI)** | **n** | **%** | **(95%CI)** | **n** | **%** | **(95%CI)** | **n** | **%** | **(95%CI)** | **n** | **%** | **(95%CI)** | **n** | **%** | **(95%CI)** |
| Abdominal pain | 0 | 0.0 | (0.0 - 6.7) | 0 | 0.0 | (0.0 - 6.7) | 0 | 0.0 | (0.0 - 6.6) | 0 | 0.0 | (0.0 - 6.7) | 0 | 0.0 | (0.0 - 6.5) | 1 | 1.9 | (0.0 - 9.9) | 0 | 0.0 | (0.0 - 6.6) | 0 | 0.0 | (0.0 - 6.6) |
| Abdominal pain upper | 0 | 0.0 | (0.0 - 6.7) | 0 | 0.0 | (0.0 - 6.7) | 0 | 0.0 | (0.0 - 6.6) | 0 | 0.0 | (0.0 - 6.7) | 1 | 1.8 | (0.0 - 9.7) | 1 | 1.9 | (0.0 - 9.9) | 0 | 0.0 | (0.0 - 6.6) | 0 | 0.0 | (0.0 - 6.6) |
| Administration site haematoma | 1 | 1.9 | (0.0 - 10.1) | 1 | 1.9 | (0.0 - 10.1) | 0 | 0.0 | (0.0 - 6.6) | 0 | 0.0 | (0.0 - 6.7) | 0 | 0.0 | (0.0 - 6.5) | 0 | 0.0 | (0.0 - 6.6) | 0 | 0.0 | (0.0 - 6.6) | 0 | 0.0 | (0.0 - 6.6) |
| Administration site paraesthesia | 0 | 0.0 | (0.0 - 6.7) | 0 | 0.0 | (0.0 - 6.7) | 0 | 0.0 | (0.0 - 6.6) | 0 | 0.0 | (0.0 - 6.7) | 0 | 0.0 | (0.0 - 6.5) | 1 | 1.9 | (0.0 - 9.9) | 1 | 1.9 | (0.0 - 9.9) | 0 | 0.0 | (0.0 - 6.6) |
| Alanine aminotransferase increased | 0 | 0.0 | (0.0 - 6.7) | 0 | 0.0 | (0.0 - 6.7) | 0 | 0.0 | (0.0 - 6.6) | 0 | 0.0 | (0.0 - 6.7) | 1 | 1.8 | (0.0 - 9.7) | 0 | 0.0 | (0.0 - 6.6) | 1 | 1.9 | (0.0 - 9.9) | 0 | 0.0 | (0.0 - 6.6) |
| Anaemia | 0 | 0.0 | (0.0 - 6.7) | 0 | 0.0 | (0.0 - 6.7) | 0 | 0.0 | (0.0 - 6.6) | 1 | 1.9 | (0.0 - 10.1) | 0 | 0.0 | (0.0 - 6.5) | 1 | 1.9 | (0.0 - 9.9) | 0 | 0.0 | (0.0 - 6.6) | 2 | 3.7 | (0.5 - 12.7) |
| Aspartate aminotransferase increased | 0 | 0.0 | (0.0 - 6.7) | 0 | 0.0 | (0.0 - 6.7) | 0 | 0.0 | (0.0 - 6.6) | 0 | 0.0 | (0.0 - 6.7) | 0 | 0.0 | (0.0 - 6.5) | 0 | 0.0 | (0.0 - 6.6) | 1 | 1.9 | (0.0 - 9.9) | 0 | 0.0 | (0.0 - 6.6) |
| Back pain | 0 | 0.0 | (0.0 - 6.7) | 0 | 0.0 | (0.0 - 6.7) | 0 | 0.0 | (0.0 - 6.6) | 0 | 0.0 | (0.0 - 6.7) | 0 | 0.0 | (0.0 - 6.5) | 1 | 1.9 | (0.0 - 9.9) | 0 | 0.0 | (0.0 - 6.6) | 0 | 0.0 | (0.0 - 6.6) |
| Blood bilirubin increased | 0 | 0.0 | (0.0 - 6.7) | 0 | 0.0 | (0.0 - 6.7) | 0 | 0.0 | (0.0 - 6.6) | 0 | 0.0 | (0.0 - 6.7) | 0 | 0.0 | (0.0 - 6.5) | 1 | 1.9 | (0.0 - 9.9) | 0 | 0.0 | (0.0 - 6.6) | 1 | 1.9 | (0.0 - 9.9) |
| Breast pain | 0 | 0.0 | (0.0 - 6.7) | 0 | 0.0 | (0.0 - 6.7) | 0 | 0.0 | (0.0 - 6.6) | 0 | 0.0 | (0.0 - 6.7) | 0 | 0.0 | (0.0 - 6.5) | 0 | 0.0 | (0.0 - 6.6) | 0 | 0.0 | (0.0 - 6.6) | 1 | 1.9 | (0.0 - 9.9) |
| Diarrhoea | 0 | 0.0 | (0.0 - 6.7) | 0 | 0.0 | (0.0 - 6.7) | 0 | 0.0 | (0.0 - 6.6) | 0 | 0.0 | (0.0 - 6.7) | 0 | 0.0 | (0.0 - 6.5) | 1 | 1.9 | (0.0 - 9.9) | 1 | 1.9 | (0.0 - 9.9) | 1 | 1.9 | (0.0 - 9.9) |
| Dizziness | 0 | 0.0 | (0.0 - 6.7) | 1 | 1.9 | (0.0 - 10.1) | 0 | 0.0 | (0.0 - 6.6) | 0 | 0.0 | (0.0 - 6.7) | 1 | 1.8 | (0.0 - 9.7) | 1 | 1.9 | (0.0 - 9.9) | 0 | 0.0 | (0.0 - 6.6) | 0 | 0.0 | (0.0 - 6.6) |
| Dry throat | 0 | 0.0 | (0.0 - 6.7) | 0 | 0.0 | (0.0 - 6.7) | 0 | 0.0 | (0.0 - 6.6) | 0 | 0.0 | (0.0 - 6.7) | 1 | 1.8 | (0.0 - 9.7) | 0 | 0.0 | (0.0 - 6.6) | 0 | 0.0 | (0.0 - 6.6) | 0 | 0.0 | (0.0 - 6.6) |
| Dysphonia | 0 | 0.0 | (0.0 - 6.7) | 0 | 0.0 | (0.0 - 6.7) | 0 | 0.0 | (0.0 - 6.6) | 0 | 0.0 | (0.0 - 6.7) | 0 | 0.0 | (0.0 - 6.5) | 1 | 1.9 | (0.0 - 9.9) | 0 | 0.0 | (0.0 - 6.6) | 0 | 0.0 | (0.0 - 6.6) |
| Ear pain | 0 | 0.0 | (0.0 - 6.7) | 0 | 0.0 | (0.0 - 6.7) | 0 | 0.0 | (0.0 - 6.6) | 0 | 0.0 | (0.0 - 6.7) | 0 | 0.0 | (0.0 - 6.5) | 0 | 0.0 | (0.0 - 6.6) | 0 | 0.0 | (0.0 - 6.6) | 1 | 1.9 | (0.0 - 9.9) |
| Erythema | 0 | 0.0 | (0.0 - 6.7) | 1 | 1.9 | (0.0 - 10.1) | 0 | 0.0 | (0.0 - 6.6) | 0 | 0.0 | (0.0 - 6.7) | 0 | 0.0 | (0.0 - 6.5) | 0 | 0.0 | (0.0 - 6.6) | 0 | 0.0 | (0.0 - 6.6) | 0 | 0.0 | (0.0 - 6.6) |

(to be continued)

**S. Table 15.** Proportion of participants with unsolicited adverse reactions occurring 7 days after the first vaccination, by study group (continuation).

| **Unsolicited Adverse Reactions** | **Group 1A** | | | **Group 1B** | | | **Group 1C** | | | **Group 2A** | | | **Group 2B** | | | **Group 2C** | | | **Group 3** | | | **Group 4** | | |
| --- | --- | --- | --- | --- | --- | --- | --- | --- | --- | --- | --- | --- | --- | --- | --- | --- | --- | --- | --- | --- | --- | --- | --- | --- |
|  | **15μg H7N9 + IB160** | | | **7.5μg H7N9 + IB160** | | | **3.75μg H7N9 + IB160** | | | **15μg H7N9 + SE** | | | **7.5μg H7N9 + SE** | | | **3.75μg H7N9 + SE** | | | **15μg H7N9, without adj** | | | **Placebo** | | |
|  | **(n=53)** | | | **(n=53)** | | | **(n=54)** | | | **(n=53)** | | | **(n=55)** | | | **(n=54)** | | | **(n=54)** | | | **(n=54)** | | |
|  | **n** | **%** | **(95%CI)** | **n** | **%** | **(95%CI)** | **n** | **%** | **(95%CI)** | **n** | **%** | **(95%CI)** | **n** | **%** | **(95%CI)** | **n** | **%** | **(95%CI)** | **n** | **%** | **(95%CI)** | **n** | **%** | **(95%CI)** |
| Generalized urticaria | 0 | 0.0 | (0.0 - 6.7) | 1 | 1.9 | (0.0 - 10.1) | 0 | 0.0 | (0.0 - 6.6) | 0 | 0.0 | (0.0 - 6.7) | 0 | 0.0 | (0.0 - 6.5) | 0 | 0.0 | (0.0 - 6.6) | 0 | 0.0 | (0.0 - 6.6) | 0 | 0.0 | (0.0 - 6.6) |
| Hyperhidrosis | 1 | 1.9 | (0.0 - 10.1) | 1 | 1.9 | (0.0 - 10.1) | 0 | 0.0 | (0.0 - 6.6) | 0 | 0.0 | (0.0 - 6.7) | 0 | 0.0 | (0.0 - 6.5) | 0 | 0.0 | (0.0 - 6.6) | 0 | 0.0 | (0.0 - 6.6) | 0 | 0.0 | (0.0 - 6.6) |
| Influenza | 1 | 1.9 | (0.0 - 10.1) | 0 | 0.0 | (0.0 - 6.7) | 0 | 0.0 | (0.0 - 6.6) | 0 | 0.0 | (0.0 - 6.7) | 0 | 0.0 | (0.0 - 6.5) | 0 | 0.0 | (0.0 - 6.6) | 0 | 0.0 | (0.0 - 6.6) | 0 | 0.0 | (0.0 - 6.6) |
| Leukocytosis | 0 | 0.0 | (0.0 - 6.7) | 0 | 0.0 | (0.0 - 6.7) | 2 | 3.7 | (0.5 - 12.7) | 0 | 0.0 | (0.0 - 6.7) | 0 | 0.0 | (0.0 - 6.5) | 0 | 0.0 | (0.0 - 6.6) | 0 | 0.0 | (0.0 - 6.6) | 0 | 0.0 | (0.0 - 6.6) |
| Leukopenia | 2 | 3.8 | (0.5 - 13.0) | 0 | 0.0 | (0.0 - 6.7) | 0 | 0.0 | (0.0 - 6.6) | 0 | 0.0 | (0.0 - 6.7) | 0 | 0.0 | (0.0 - 6.5) | 0 | 0.0 | (0.0 - 6.6) | 0 | 0.0 | (0.0 - 6.6) | 0 | 0.0 | (0.0 - 6.6) |
| Lymphopenia | 0 | 0.0 | (0.0 - 6.7) | 0 | 0.0 | (0.0 - 6.7) | 0 | 0.0 | (0.0 - 6.6) | 2 | 3.8 | (0.5 - 13.0) | 0 | 0.0 | (0.0 - 6.5) | 0 | 0.0 | (0.0 - 6.6) | 0 | 0.0 | (0.0 - 6.6) | 0 | 0.0 | (0.0 - 6.6) |
| Migraine | 0 | 0.0 | (0.0 - 6.7) | 0 | 0.0 | (0.0 - 6.7) | 0 | 0.0 | (0.0 - 6.6) | 0 | 0.0 | (0.0 - 6.7) | 1 | 1.8 | (0.0 - 9.7) | 0 | 0.0 | (0.0 - 6.6) | 1 | 1.9 | (0.0 - 9.9) | 0 | 0.0 | (0.0 - 6.6) |
| Mouth ulceration | 0 | 0.0 | (0.0 - 6.7) | 0 | 0.0 | (0.0 - 6.7) | 0 | 0.0 | (0.0 - 6.6) | 1 | 1.9 | (0.0 - 10.1) | 0 | 0.0 | (0.0 - 6.5) | 0 | 0.0 | (0.0 - 6.6) | 0 | 0.0 | (0.0 - 6.6) | 0 | 0.0 | (0.0 - 6.6) |
| Muscle spasms | 0 | 0.0 | (0.0 - 6.7) | 0 | 0.0 | (0.0 - 6.7) | 1 | 1.9 | (0.0 - 9.9) | 0 | 0.0 | (0.0 - 6.7) | 1 | 1.8 | (0.0 - 9.7) | 0 | 0.0 | (0.0 - 6.6) | 0 | 0.0 | (0.0 - 6.6) | 0 | 0.0 | (0.0 - 6.6) |
| Nasal congestion | 1 | 1.9 | (0.0 - 10.1) | 0 | 0.0 | (0.0 - 6.7) | 0 | 0.0 | (0.0 - 6.6) | 0 | 0.0 | (0.0 - 6.7) | 0 | 0.0 | (0.0 - 6.5) | 0 | 0.0 | (0.0 - 6.6) | 0 | 0.0 | (0.0 - 6.6) | 0 | 0.0 | (0.0 - 6.6) |
| Neutropenia | 1 | 1.9 | (0.0 - 10.1) | 0 | 0.0 | (0.0 - 6.7) | 0 | 0.0 | (0.0 - 6.6) | 0 | 0.0 | (0.0 - 6.7) | 2 | 3.6 | (0.4 - 12.5) | 0 | 0.0 | (0.0 - 6.6) | 0 | 0.0 | (0.0 - 6.6) | 0 | 0.0 | (0.0 - 6.6) |
| Oedema | 0 | 0.0 | (0.0 - 6.7) | 1 | 1.9 | (0.0 - 10.1) | 0 | 0.0 | (0.0 - 6.6) | 0 | 0.0 | (0.0 - 6.7) | 0 | 0.0 | (0.0 - 6.5) | 0 | 0.0 | (0.0 - 6.6) | 0 | 0.0 | (0.0 - 6.6) | 0 | 0.0 | (0.0 - 6.6) |
| Oropharyngeal pain | 0 | 0.0 | (0.0 - 6.7) | 0 | 0.0 | (0.0 - 6.7) | 0 | 0.0 | (0.0 - 6.6) | 0 | 0.0 | (0.0 - 6.7) | 1 | 1.8 | (0.0 - 9.7) | 1 | 1.9 | (0.0 - 9.9) | 1 | 1.9 | (0.0 - 9.9) | 2 | 3.7 | (0.5 - 12.7) |
| Pain in extremity | 0 | 0.0 | (0.0 - 6.7) | 1 | 1.9 | (0.0 - 10.1) | 0 | 0.0 | (0.0 - 6.6) | 0 | 0.0 | (0.0 - 6.7) | 0 | 0.0 | (0.0 - 6.5) | 0 | 0.0 | (0.0 - 6.6) | 0 | 0.0 | (0.0 - 6.6) | 0 | 0.0 | (0.0 - 6.6) |
| Paraesthesia | 0 | 0.0 | (0.0 - 6.7) | 0 | 0.0 | (0.0 - 6.7) | 0 | 0.0 | (0.0 - 6.6) | 0 | 0.0 | (0.0 - 6.7) | 0 | 0.0 | (0.0 - 6.5) | 0 | 0.0 | (0.0 - 6.6) | 1 | 1.9 | (0.0 - 9.9) | 0 | 0.0 | (0.0 - 6.6) |
| Pharyngitis | 0 | 0.0 | (0.0 - 6.7) | 0 | 0.0 | (0.0 - 6.7) | 0 | 0.0 | (0.0 - 6.6) | 0 | 0.0 | (0.0 - 6.7) | 1 | 1.8 | (0.0 - 9.7) | 0 | 0.0 | (0.0 - 6.6) | 0 | 0.0 | (0.0 - 6.6) | 0 | 0.0 | (0.0 - 6.6) |
| Pruritus | 0 | 0.0 | (0.0 - 6.7) | 1 | 1.9 | (0.0 - 10.1) | 0 | 0.0 | (0.0 - 6.6) | 0 | 0.0 | (0.0 - 6.7) | 0 | 0.0 | (0.0 - 6.5) | 2 | 3.7 | (0.5 - 12.7) | 1 | 1.9 | (0.0 - 9.9) | 0 | 0.0 | (0.0 - 6.6) |
| Rash | 0 | 0.0 | (0.0 - 6.7) | 0 | 0.0 | (0.0 - 6.7) | 0 | 0.0 | (0.0 - 6.6) | 0 | 0.0 | (0.0 - 6.7) | 0 | 0.0 | (0.0 - 6.5) | 1 | 1.9 | (0.0 - 9.9) | 0 | 0.0 | (0.0 - 6.6) | 0 | 0.0 | (0.0 - 6.6) |
| Rhinitis | 0 | 0.0 | (0.0 - 6.7) | 0 | 0.0 | (0.0 - 6.7) | 0 | 0.0 | (0.0 - 6.6) | 0 | 0.0 | (0.0 - 6.7) | 1 | 1.8 | (0.0 - 9.7) | 1 | 1.9 | (0.0 - 9.9) | 0 | 0.0 | (0.0 - 6.6) | 0 | 0.0 | (0.0 - 6.6) |

(to be continued)

**S. Table 15.** Proportion of participants with unsolicited adverse reactions occurring 7 days after the first vaccination, by study group (continuation).

| **Unsolicited Adverse Reactions** | **Group 1A** | | | **Group 1B** | | | **Group 1C** | | | **Group 2A** | | | **Group 2B** | | | **Group 2C** | | | **Group 3** | | | **Group 4** | | |
| --- | --- | --- | --- | --- | --- | --- | --- | --- | --- | --- | --- | --- | --- | --- | --- | --- | --- | --- | --- | --- | --- | --- | --- | --- |
|  | **15μg H7N9 + IB160** | | | **7.5μg H7N9 + IB160** | | | **3.75μg H7N9 + IB160** | | | **15μg H7N9 + SE** | | | **7.5μg H7N9 + SE** | | | **3.75μg H7N9 + SE** | | | **15μg H7N9, without adj** | | | **Placebo** | | |
|  | **(n=53)** | | | **(n=53)** | | | **(n=54)** | | | **(n=53)** | | | **(n=55)** | | | **(n=54)** | | | **(n=54)** | | | **(n=54)** | | |
|  | **n** | **%** | **(95%CI)** | **n** | **%** | **(95%CI)** | **n** | **%** | **(95%CI)** | **n** | **%** | **(95%CI)** | **n** | **%** | **(95%CI)** | **n** | **%** | **(95%CI)** | **n** | **%** | **(95%CI)** | **n** | **%** | **(95%CI)** |
| Rhinorrhoea | 0 | 0.0 | (0.0 - 6.7) | 0 | 0.0 | (0.0 - 6.7) | 0 | 0.0 | (0.0 - 6.6) | 1 | 1.9 | (0.0 - 10.1) | 2 | 3.6 | (0.4 - 12.5) | 0 | 0.0 | (0.0 - 6.6) | 1 | 1.9 | (0.0 - 9.9) | 1 | 1.9 | (0.0 - 9.9) |
| Sneezing | 0 | 0.0 | (0.0 - 6.7) | 0 | 0.0 | (0.0 - 6.7) | 1 | 1.9 | (0.0 - 9.9) | 0 | 0.0 | (0.0 - 6.7) | 0 | 0.0 | (0.0 - 6.5) | 0 | 0.0 | (0.0 - 6.6) | 0 | 0.0 | (0.0 - 6.6) | 0 | 0.0 | (0.0 - 6.6) |
| Somnolence | 0 | 0.0 | (0.0 - 6.7) | 0 | 0.0 | (0.0 - 6.7) | 0 | 0.0 | (0.0 - 6.6) | 0 | 0.0 | (0.0 - 6.7) | 1 | 1.8 | (0.0 - 9.7) | 2 | 3.7 | (0.5 - 12.7) | 0 | 0.0 | (0.0 - 6.6) | 2 | 3.7 | (0.5 - 12.7) |
| Transaminases increased | 0 | 0.0 | (0.0 - 6.7) | 1 | 1.9 | (0.0 - 10.1) | 1 | 1.9 | (0.0 - 9.9) | 0 | 0.0 | (0.0 - 6.7) | 0 | 0.0 | (0.0 - 6.5) | 0 | 0.0 | (0.0 - 6.6) | 0 | 0.0 | (0.0 - 6.6) | 1 | 1.9 | (0.0 - 9.9) |
| Upper respiratory tract infection | 0 | 0.0 | (0.0 - 6.7) | 0 | 0.0 | (0.0 - 6.7) | 0 | 0.0 | (0.0 - 6.6) | 0 | 0.0 | (0.0 - 6.7) | 0 | 0.0 | (0.0 - 6.5) | 1 | 1.9 | (0.0 - 9.9) | 0 | 0.0 | (0.0 - 6.6) | 0 | 0.0 | (0.0 - 6.6) |
| Others | 1 | 1.9 | (0.0 - 10.1) | 2 | 3.8 | (0.5 - 13.0) | 2 | 3.7 | (0.5 - 12.7) | 1 | 1.9 | (0.0 - 10.1) | 1 | 1.8 | (0.0 - 9.7) | 0 | 0.0 | (0.0 - 6.6) | 0 | 0.0 | (0.0 - 6.6) | 3 | 5.6 | (1.2 - 15.4) |
| Abdominal pain | 0 | 0.0 | (0.0 - 6.7) | 0 | 0.0 | (0.0 - 6.7) | 0 | 0.0 | (0.0 - 6.6) | 0 | 0.0 | (0.0 - 6.7) | 0 | 0.0 | (0.0 - 6.5) | 1 | 1.9 | (0.0 - 9.9) | 0 | 0.0 | (0.0 - 6.6) | 0 | 0.0 | (0.0 - 6.6) |
| **TOTAL** | 7 | 13.2 | (5.5 - 25.3) | 7 | 13.2 | (5.5 - 25.3) | 7 | 13.0 | (5.4 - 24.9) | 6 | 11.3 | (4.3 - 23.0) | 13 | 23.6 | (13.2 - 37.0) | 13 | 24.1 | (13.5 - 37.6) | 6 | 11.1 | (4.2 - 22.6) | 13 | 24.1 | (13.5 - 37.6) |
| adj: adjuvant; 95%CI: 95% confidence interval | | | | | | | | | | | | | | | | | | | | | | | | |

(end)

**S. Table 16.** Proportion of participants with unsolicited adverse reactions occurring 7 days after the second vaccination, by study group.

| **Unsolicited Adverse Reactions** | **Group 1A** | | | **Group 1B** | | | **Group 1C** | | | **Group 2A** | | | **Group 2B** | | | **Group 2C** | | | **Group 3** | | | **Group 4** | | |
| --- | --- | --- | --- | --- | --- | --- | --- | --- | --- | --- | --- | --- | --- | --- | --- | --- | --- | --- | --- | --- | --- | --- | --- | --- |
|  | **15μg H7N9 + IB160** | | | **7.5μg H7N9 + IB160** | | | **3.75μg H7N9 + IB160** | | | **15μg H7N9 + SE** | | | **7.5μg H7N9 + SE** | | | **3.75μg H7N9 + SE** | | | **15μg H7N9, without adj** | | | **Placebo** | | |
|  | **(n=53)** | | | **(n=53)** | | | **(n=54)** | | | **(n=53)** | | | **(n=55)** | | | **(n=54)** | | | **(n=54)** | | | **(n=54)** | | |
|  | **n** | **%** | **(95%CI)** | **n** | **%** | **(95%CI)** | **n** | **%** | **(95%CI)** | **n** | **%** | **(95%CI)** | **n** | **%** | **(95%CI)** | **n** | **%** | **(95%CI)** | **n** | **%** | **(95%CI)** | **n** | **%** | **(95%CI)** |
| Administration site haematoma | 1 | 2.0 | (0.1 - 10.6) | 1 | 2.1 | (0.1 - 11.1) | 0 | 0.0 | (0.0 - 7.0) | 0 | 0.0 | (0.0 - 7.4) | 0 | 0.0 | (0.0 - 6.7) | 0 | 0.0 | (0.0 - 7.0) | 1 | 2.0 | (0.0 - 10.4) | 0 | 0.0 | (0.0 - 7.4) |
| Administration site haemorrhage | 1 | 2.0 | (0.1 - 10.6) | 0 | 0.0 | (0.0 - 7.4) | 0 | 0.0 | (0.0 - 7.0) | 0 | 0.0 | (0.0 - 7.4) | 0 | 0.0 | (0.0 - 6.7) | 0 | 0.0 | (0.0 - 7.0) | 0 | 0.0 | (0.0 - 7.0) | 1 | 2.1 | (0.1 - 11.1) |
| Administration site paraesthesia | 1 | 2.0 | (0.1 - 10.6) | 0 | 0.0 | (0.0 - 7.4) | 0 | 0.0 | (0.0 - 7.0) | 0 | 0.0 | (0.0 - 7.4) | 0 | 0.0 | (0.0 - 6.7) | 0 | 0.0 | (0.0 - 7.0) | 0 | 0.0 | (0.0 - 7.0) | 0 | 0.0 | (0.0 - 7.4) |
| Alanine aminotransferase increased | 1 | 2.0 | (0.1 - 10.6) | 1 | 2.1 | (0.1 - 11.1) | 0 | 0.0 | (0.0 - 7.0) | 1 | 2.1 | (0.1 - 11.1) | 1 | 1.9 | (0.0 - 10.1) | 1 | 2.0 | (0.0 - 10.4) | 0 | 0.0 | (0.0 - 7.0) | 0 | 0.0 | (0.0 - 7.4) |
| Anaemia | 0 | 0.0 | (0.0 - 7.1) | 0 | 0.0 | (0.0 - 7.4) | 1 | 2.0 | (0.0 - 10.4) | 1 | 2.1 | (0.1 - 11.1) | 0 | 0.0 | (0.0 - 6.7) | 0 | 0.0 | (0.0 - 7.0) | 0 | 0.0 | (0.0 - 7.0) | 0 | 0.0 | (0.0 - 7.4) |
| Back pain | 0 | 0.0 | (0.0 - 7.1) | 0 | 0.0 | (0.0 - 7.4) | 0 | 0.0 | (0.0 - 7.0) | 0 | 0.0 | (0.0 - 7.4) | 0 | 0.0 | (0.0 - 6.7) | 0 | 0.0 | (0.0 - 7.0) | 0 | 0.0 | (0.0 - 7.0) | 1 | 2.1 | (0.1 - 11.1) |
| Blood bilirubin increased | 1 | 2.0 | (0.1 - 10.6) | 0 | 0.0 | (0.0 - 7.4) | 0 | 0.0 | (0.0 - 7.0) | 0 | 0.0 | (0.0 - 7.4) | 0 | 0.0 | (0.0 - 6.7) | 1 | 2.0 | (0.0 - 10.4) | 1 | 2.0 | (0.0 - 10.4) | 0 | 0.0 | (0.0 - 7.4) |
| Dizziness | 0 | 0.0 | (0.0 - 7.1) | 1 | 2.1 | (0.1 - 11.1) | 0 | 0.0 | (0.0 - 7.0) | 1 | 2.1 | (0.1 - 11.1) | 2 | 3.8 | (0.5 - 13.0) | 0 | 0.0 | (0.0 - 7.0) | 1 | 2.0 | (0.0 - 10.4) | 0 | 0.0 | (0.0 - 7.4) |
| Dry throat | 0 | 0.0 | (0.0 - 7.1) | 0 | 0.0 | (0.0 - 7.4) | 0 | 0.0 | (0.0 - 7.0) | 0 | 0.0 | (0.0 - 7.4) | 1 | 1.9 | (0.0 - 10.1) | 0 | 0.0 | (0.0 - 7.0) | 0 | 0.0 | (0.0 - 7.0) | 0 | 0.0 | (0.0 - 7.4) |
| Dysphonia | 0 | 0.0 | (0.0 - 7.1) | 0 | 0.0 | (0.0 - 7.4) | 1 | 2.0 | (0.0 - 10.4) | 0 | 0.0 | (0.0 - 7.4) | 0 | 0.0 | (0.0 - 6.7) | 0 | 0.0 | (0.0 - 7.0) | 0 | 0.0 | (0.0 - 7.0) | 0 | 0.0 | (0.0 - 7.4) |
| Eye pain | 0 | 0.0 | (0.0 - 7.1) | 1 | 2.1 | (0.1 - 11.1) | 0 | 0.0 | (0.0 - 7.0) | 0 | 0.0 | (0.0 - 7.4) | 0 | 0.0 | (0.0 - 6.7) | 0 | 0.0 | (0.0 - 7.0) | 0 | 0.0 | (0.0 - 7.0) | 0 | 0.0 | (0.0 - 7.4) |
| Influenza | 0 | 0.0 | (0.0 - 7.1) | 0 | 0.0 | (0.0 - 7.4) | 0 | 0.0 | (0.0 - 7.0) | 0 | 0.0 | (0.0 - 7.4) | 1 | 1.9 | (0.0 - 10.1) | 0 | 0.0 | (0.0 - 7.0) | 1 | 2.0 | (0.0 - 10.4) | 0 | 0.0 | (0.0 - 7.4) |
| Leukopenia | 0 | 0.0 | (0.0 - 7.1) | 0 | 0.0 | (0.0 - 7.4) | 0 | 0.0 | (0.0 - 7.0) | 1 | 2.1 | (0.1 - 11.1) | 0 | 0.0 | (0.0 - 6.7) | 0 | 0.0 | (0.0 - 7.0) | 0 | 0.0 | (0.0 - 7.0) | 1 | 2.1 | (0.1 - 11.1) |
| Musculoskeletal pain | 0 | 0.0 | (0.0 - 7.1) | 0 | 0.0 | (0.0 - 7.4) | 0 | 0.0 | (0.0 - 7.0) | 0 | 0.0 | (0.0 - 7.4) | 0 | 0.0 | (0.0 - 6.7) | 0 | 0.0 | (0.0 - 7.0) | 1 | 2.0 | (0.0 - 10.4) | 0 | 0.0 | (0.0 - 7.4) |
| Neutropenia | 0 | 0.0 | (0.0 - 7.1) | 0 | 0.0 | (0.0 - 7.4) | 0 | 0.0 | (0.0 - 7.0) | 0 | 0.0 | (0.0 - 7.4) | 0 | 0.0 | (0.0 - 6.7) | 0 | 0.0 | (0.0 - 7.0) | 0 | 0.0 | (0.0 - 7.0) | 2 | 4.2 | (0.5 - 14.3) |
| Odynophagia | 0 | 0.0 | (0.0 - 7.1) | 1 | 2.1 | (0.1 - 11.1) | 1 | 2.0 | (0.0 - 10.4) | 0 | 0.0 | (0.0 - 7.4) | 0 | 0.0 | (0.0 - 6.7) | 0 | 0.0 | (0.0 - 7.0) | 0 | 0.0 | (0.0 - 7.0) | 0 | 0.0 | (0.0 - 7.4) |
| Oropharyngeal pain | 1 | 2.0 | (0.1 - 10.6) | 1 | 2.1 | (0.1 - 11.1) | 0 | 0.0 | (0.0 - 7.0) | 0 | 0.0 | (0.0 - 7.4) | 0 | 0.0 | (0.0 - 6.7) | 0 | 0.0 | (0.0 - 7.0) | 0 | 0.0 | (0.0 - 7.0) | 0 | 0.0 | (0.0 - 7.4) |

(to be continued)

**S. Table 16.** Proportion of participants with unsolicited adverse reactions occurring 7 days after the second vaccination, by study group (continuation).

| **Unsolicited Adverse Reactions** | **Group 1A** | | | **Group 1B** | | | **Group 1C** | | | **Group 2A** | | | **Group 2B** | | | **Group 2C** | | | **Group 3** | | | **Group 4** | | |
| --- | --- | --- | --- | --- | --- | --- | --- | --- | --- | --- | --- | --- | --- | --- | --- | --- | --- | --- | --- | --- | --- | --- | --- | --- |
|  | **15μg H7N9 + IB160** | | | **7.5μg H7N9 + IB160** | | | **3.75μg H7N9 + IB160** | | | **15μg H7N9 + SE** | | | **7.5μg H7N9 + SE** | | | **3.75μg H7N9 + SE** | | | **15μg H7N9, without adj** | | | **Placebo** | | |
|  | **(n=53)** | | | **(n=53)** | | | **(n=54)** | | | **(n=53)** | | | **(n=55)** | | | **(n=54)** | | | **(n=54)** | | | **(n=54)** | | |
|  | **n** | **%** | **(95%CI)** | **n** | **%** | **(95%CI)** | **n** | **%** | **(95%CI)** | **n** | **%** | **(95%CI)** | **n** | **%** | **(95%CI)** | **n** | **%** | **(95%CI)** | **n** | **%** | **(95%CI)** | **n** | **%** | **(95%CI)** |
| Pain in extremity | 0 | 0.0 | (0.0 - 7.1) | 0 | 0.0 | (0.0 - 7.4) | 0 | 0.0 | (0.0 - 7.0) | 0 | 0.0 | (0.0 - 7.4) | 0 | 0.0 | (0.0 - 6.7) | 0 | 0.0 | (0.0 - 7.0) | 0 | 0.0 | (0.0 - 7.0) | 1 | 2.1 | (0.1 - 11.1) |
| Paraesthesia | 1 | 2.0 | (0.1 - 10.6) | 0 | 0.0 | (0.0 - 7.4) | 0 | 0.0 | (0.0 - 7.0) | 0 | 0.0 | (0.0 - 7.4) | 0 | 0.0 | (0.0 - 6.7) | 0 | 0.0 | (0.0 - 7.0) | 0 | 0.0 | (0.0 - 7.0) | 0 | 0.0 | (0.0 - 7.4) |
| Rhinorrhoea | 0 | 0.0 | (0.0 - 7.1) | 0 | 0.0 | (0.0 - 7.4) | 1 | 2.0 | (0.0 - 10.4) | 0 | 0.0 | (0.0 - 7.4) | 0 | 0.0 | (0.0 - 6.7) | 0 | 0.0 | (0.0 - 7.0) | 0 | 0.0 | (0.0 - 7.0) | 0 | 0.0 | (0.0 - 7.4) |
| Sneezing | 0 | 0.0 | (0.0 - 7.1) | 0 | 0.0 | (0.0 - 7.4) | 1 | 2.0 | (0.0 - 10.4) | 0 | 0.0 | (0.0 - 7.4) | 0 | 0.0 | (0.0 - 6.7) | 0 | 0.0 | (0.0 - 7.0) | 0 | 0.0 | (0.0 - 7.0) | 0 | 0.0 | (0.0 - 7.4) |
| Somnolence | 0 | 0.0 | (0.0 - 7.1) | 0 | 0.0 | (0.0 - 7.4) | 0 | 0.0 | (0.0 - 7.0) | 0 | 0.0 | (0.0 - 7.4) | 0 | 0.0 | (0.0 - 6.7) | 1 | 2.0 | (0.0 - 10.4) | 0 | 0.0 | (0.0 - 7.0) | 0 | 0.0 | (0.0 - 7.4) |
| Throat irritation | 0 | 0.0 | (0.0 - 7.1) | 0 | 0.0 | (0.0 - 7.4) | 0 | 0.0 | (0.0 - 7.0) | 1 | 2.1 | (0.1 - 11.1) | 0 | 0.0 | (0.0 - 6.7) | 0 | 0.0 | (0.0 - 7.0) | 0 | 0.0 | (0.0 - 7.0) | 0 | 0.0 | (0.0 - 7.4) |
| Transaminases increased | 0 | 0.0 | (0.0 - 7.1) | 0 | 0.0 | (0.0 - 7.4) | 0 | 0.0 | (0.0 - 7.0) | 1 | 2.1 | (0.1 - 11.1) | 1 | 1.9 | (0.0 - 10.1) | 1 | 2.0 | (0.0 - 10.4) | 0 | 0.0 | (0.0 - 7.0) | 1 | 2.1 | (0.1 - 11.1) |
| Others | 1 | 2.0 | (0.1 - 10.6) | 3 | 6.3 | (1.3 - 17.2) | 2 | 3.9 | (0.5 - 13.5) | 0 | 0.0 | (0.0 - 7.4) | 3 | 5.7 | (1.2 - 15.7) | 0 | 0.0 | (0.0 - 7.0) | 3 | 5.9 | (1.2 - 16.2) | 1 | 2.1 | (0.1 - 11.1) |
| **TOTAL** | 5 | 10.0 | (3.3 - 21.8) | 8 | 16.7 | (7.5 - 30.2) | 5 | 9.8 | (3.3 - 21.4) | 5 | 10.4 | (3.5 - 22.7) | 7 | 13.2 | (5.5 - 25.3) | 4 | 7.8 | (2.2 - 18.9) | 8 | 15.7 | (7.0 - 28.6) | 6 | 12.5 | (4.7 - 25.2) |
| adj: adjuvant; 95%CI: 95% confidence interval | | | | | | | | | | | | | | | | | | | | | | | | |

(end)

**S. Table 17.** Proportion of participants with unsolicited adverse reactions occurring 28 days after the first vaccination, by study group.

| **Unsolicited Adverse Reactions** | **Group 1A** | | | **Group 1B** | | | **Group 1C** | | | **Group 2A** | | | **Group 2B** | | | **Group 2C** | | | **Group 3** | | | **Group 4** | | |
| --- | --- | --- | --- | --- | --- | --- | --- | --- | --- | --- | --- | --- | --- | --- | --- | --- | --- | --- | --- | --- | --- | --- | --- | --- |
|  | **15μg H7N9 + IB160** | | | **7.5μg H7N9 + IB160** | | | **3.75μg H7N9 + IB160** | | | **15μg H7N9 + SE** | | | **7.5μg H7N9 + SE** | | | **3.75μg H7N9 + SE** | | | **15μg H7N9, without adj** | | | **Placebo** | | |
|  | **(n=53)** | | | **(n=53)** | | | **(n=54)** | | | **(n=53)** | | | **(n=55)** | | | **(n=54)** | | | **(n=54)** | | | **(n=54)** | | |
|  | **n** | **%** | **(95%CI)** | **n** | **%** | **(95%CI)** | **n** | **%** | **(95%CI)** | **n** | **%** | **(95%CI)** | **n** | **%** | **(95%CI)** | **n** | **%** | **(95%CI)** | **n** | **%** | **(95%CI)** | **n** | **%** | **(95%CI)** |
| Abdominal pain | 0 | 0.0 | (0.0 - 6.7) | 0 | 0.0 | (0.0 - 6.7) | 0 | 0.0 | (0.0 - 6.6) | 0 | 0.0 | (0.0 - 6.7) | 0 | 0.0 | (0.0 - 6.5) | 1 | 1.9 | (0.0 - 9.9) | 0 | 0.0 | (0.0 - 6.6) | 0 | 0.0 | (0.0 - 6.6) |
| Abdominal pain upper | 0 | 0.0 | (0.0 - 6.7) | 0 | 0.0 | (0.0 - 6.7) | 0 | 0.0 | (0.0 - 6.6) | 0 | 0.0 | (0.0 - 6.7) | 1 | 1.8 | (0.0 - 9.7) | 1 | 1.9 | (0.0 - 9.9) | 0 | 0.0 | (0.0 - 6.6) | 0 | 0.0 | (0.0 - 6.6) |
| Administration site haematoma | 1 | 1.9 | (0.0 - 10.1) | 1 | 1.9 | (0.0 - 10.1) | 0 | 0.0 | (0.0 - 6.6) | 0 | 0.0 | (0.0 - 6.7) | 0 | 0.0 | (0.0 - 6.5) | 0 | 0.0 | (0.0 - 6.6) | 0 | 0.0 | (0.0 - 6.6) | 0 | 0.0 | (0.0 - 6.6) |
| Administration site paraesthesia | 0 | 0.0 | (0.0 - 6.7) | 0 | 0.0 | (0.0 - 6.7) | 0 | 0.0 | (0.0 - 6.6) | 0 | 0.0 | (0.0 - 6.7) | 0 | 0.0 | (0.0 - 6.5) | 1 | 1.9 | (0.0 - 9.9) | 1 | 1.9 | (0.0 - 9.9) | 0 | 0.0 | (0.0 - 6.6) |
| Alanine aminotransferase increased | 0 | 0.0 | (0.0 - 6.7) | 0 | 0.0 | (0.0 - 6.7) | 0 | 0.0 | (0.0 - 6.6) | 0 | 0.0 | (0.0 - 6.7) | 1 | 1.8 | (0.0 - 9.7) | 0 | 0.0 | (0.0 - 6.6) | 2 | 3.7 | (0.5 - 12.7) | 0 | 0.0 | (0.0 - 6.6) |
| Anaemia | 0 | 0.0 | (0.0 - 6.7) | 0 | 0.0 | (0.0 - 6.7) | 0 | 0.0 | (0.0 - 6.6) | 1 | 1.9 | (0.0 - 10.1) | 0 | 0.0 | (0.0 - 6.5) | 1 | 1.9 | (0.0 - 9.9) | 0 | 0.0 | (0.0 - 6.6) | 2 | 3.7 | (0.5 - 12.7) |
| Aspartate aminotransferase increased | 1 | 1.9 | (0.0 - 10.1) | 0 | 0.0 | (0.0 - 6.7) | 0 | 0.0 | (0.0 - 6.6) | 0 | 0.0 | (0.0 - 6.7) | 0 | 0.0 | (0.0 - 6.5) | 0 | 0.0 | (0.0 - 6.6) | 1 | 1.9 | (0.0 - 9.9) | 0 | 0.0 | (0.0 - 6.6) |
| Back pain | 0 | 0.0 | (0.0 - 6.7) | 0 | 0.0 | (0.0 - 6.7) | 0 | 0.0 | (0.0 - 6.6) | 0 | 0.0 | (0.0 - 6.7) | 0 | 0.0 | (0.0 - 6.5) | 1 | 1.9 | (0.0 - 9.9) | 0 | 0.0 | (0.0 - 6.6) | 0 | 0.0 | (0.0 - 6.6) |
| Blood bilirubin increased | 0 | 0.0 | (0.0 - 6.7) | 0 | 0.0 | (0.0 - 6.7) | 0 | 0.0 | (0.0 - 6.6) | 0 | 0.0 | (0.0 - 6.7) | 0 | 0.0 | (0.0 - 6.5) | 1 | 1.9 | (0.0 - 9.9) | 0 | 0.0 | (0.0 - 6.6) | 1 | 1.9 | (0.0 - 9.9) |
| Breast pain | 0 | 0.0 | (0.0 - 6.7) | 0 | 0.0 | (0.0 - 6.7) | 0 | 0.0 | (0.0 - 6.6) | 0 | 0.0 | (0.0 - 6.7) | 0 | 0.0 | (0.0 - 6.5) | 0 | 0.0 | (0.0 - 6.6) | 0 | 0.0 | (0.0 - 6.6) | 1 | 1.9 | (0.0 - 9.9) |
| Diarrhoea | 1 | 1.9 | (0.0 - 10.1) | 0 | 0.0 | (0.0 - 6.7) | 0 | 0.0 | (0.0 - 6.6) | 0 | 0.0 | (0.0 - 6.7) | 0 | 0.0 | (0.0 - 6.5) | 1 | 1.9 | (0.0 - 9.9) | 1 | 1.9 | (0.0 - 9.9) | 1 | 1.9 | (0.0 - 9.9) |
| Dizziness | 0 | 0.0 | (0.0 - 6.7) | 1 | 1.9 | (0.0 - 10.1) | 0 | 0.0 | (0.0 - 6.6) | 0 | 0.0 | (0.0 - 6.7) | 1 | 1.8 | (0.0 - 9.7) | 1 | 1.9 | (0.0 - 9.9) | 0 | 0.0 | (0.0 - 6.6) | 0 | 0.0 | (0.0 - 6.6) |
| Dry throat | 0 | 0.0 | (0.0 - 6.7) | 0 | 0.0 | (0.0 - 6.7) | 0 | 0.0 | (0.0 - 6.6) | 0 | 0.0 | (0.0 - 6.7) | 1 | 1.8 | (0.0 - 9.7) | 0 | 0.0 | (0.0 - 6.6) | 0 | 0.0 | (0.0 - 6.6) | 0 | 0.0 | (0.0 - 6.6) |
| Dysphonia | 0 | 0.0 | (0.0 - 6.7) | 0 | 0.0 | (0.0 - 6.7) | 0 | 0.0 | (0.0 - 6.6) | 0 | 0.0 | (0.0 - 6.7) | 0 | 0.0 | (0.0 - 6.5) | 1 | 1.9 | (0.0 - 9.9) | 0 | 0.0 | (0.0 - 6.6) | 0 | 0.0 | (0.0 - 6.6) |
| Ear pain | 0 | 0.0 | (0.0 - 6.7) | 0 | 0.0 | (0.0 - 6.7) | 0 | 0.0 | (0.0 - 6.6) | 0 | 0.0 | (0.0 - 6.7) | 0 | 0.0 | (0.0 - 6.5) | 0 | 0.0 | (0.0 - 6.6) | 0 | 0.0 | (0.0 - 6.6) | 1 | 1.9 | (0.0 - 9.9) |

(to be continued)

**S. Table 17.** Proportion of participants with unsolicited adverse reactions occurring 28 days after the first vaccination, by study group (continuation).

| **Unsolicited Adverse Reactions** | **Group 1A** | | | **Group 1B** | | | **Group 1C** | | | **Group 2A** | | | **Group 2B** | | | **Group 2C** | | | **Group 3** | | | **Group 4** | | |
| --- | --- | --- | --- | --- | --- | --- | --- | --- | --- | --- | --- | --- | --- | --- | --- | --- | --- | --- | --- | --- | --- | --- | --- | --- |
|  | **15μg H7N9 + IB160** | | | **7.5μg H7N9 + IB160** | | | **3.75μg H7N9 + IB160** | | | **15μg H7N9 + SE** | | | **7.5μg H7N9 + SE** | | | **3.75μg H7N9 + SE** | | | **15μg H7N9, without adj** | | | **Placebo** | | |
|  | **(n=53)** | | | **(n=53)** | | | **(n=54)** | | | **(n=53)** | | | **(n=55)** | | | **(n=54)** | | | **(n=54)** | | | **(n=54)** | | |
|  | **n** | **%** | **(95%CI)** | **n** | **%** | **(95%CI)** | **n** | **%** | **(95%CI)** | **n** | **%** | **(95%CI)** | **n** | **%** | **(95%CI)** | **n** | **%** | **(95%CI)** | **n** | **%** | **(95%CI)** | **n** | **%** | **(95%CI)** |
| Erythema | 0 | 0.0 | (0.0 - 6.7) | 1 | 1.9 | (0.0 - 10.1) | 0 | 0.0 | (0.0 - 6.6) | 0 | 0.0 | (0.0 - 6.7) | 0 | 0.0 | (0.0 - 6.5) | 0 | 0.0 | (0.0 - 6.6) | 0 | 0.0 | (0.0 - 6.6) | 0 | 0.0 | (0.0 - 6.6) |
| Generalized urticaria | 0 | 0.0 | (0.0 - 6.7) | 1 | 1.9 | (0.0 - 10.1) | 0 | 0.0 | (0.0 - 6.6) | 0 | 0.0 | (0.0 - 6.7) | 0 | 0.0 | (0.0 - 6.5) | 0 | 0.0 | (0.0 - 6.6) | 0 | 0.0 | (0.0 - 6.6) | 0 | 0.0 | (0.0 - 6.6) |
| Headache | 0 | 0.0 | (0.0 - 6.7) | 0 | 0.0 | (0.0 - 6.7) | 1 | 1.9 | (0.0 - 9.9) | 1 | 1.9 | (0.0 - 10.1) | 2 | 3.6 | (0.4 - 12.5) | 1 | 1.9 | (0.0 - 9.9) | 1 | 1.9 | (0.0 - 9.9) | 0 | 0.0 | (0.0 - 6.6) |
| Hyperhidrosis | 1 | 1.9 | (0.0 - 10.1) | 1 | 1.9 | (0.0 - 10.1) | 0 | 0.0 | (0.0 - 6.6) | 0 | 0.0 | (0.0 - 6.7) | 0 | 0.0 | (0.0 - 6.5) | 0 | 0.0 | (0.0 - 6.6) | 0 | 0.0 | (0.0 - 6.6) | 0 | 0.0 | (0.0 - 6.6) |
| Influenza | 1 | 1.9 | (0.0 - 10.1) | 0 | 0.0 | (0.0 - 6.7) | 0 | 0.0 | (0.0 - 6.6) | 0 | 0.0 | (0.0 - 6.7) | 0 | 0.0 | (0.0 - 6.5) | 0 | 0.0 | (0.0 - 6.6) | 0 | 0.0 | (0.0 - 6.6) | 0 | 0.0 | (0.0 - 6.6) |
| Leukocytosis | 0 | 0.0 | (0.0 - 6.7) | 0 | 0.0 | (0.0 - 6.7) | 2 | 3.7 | (0.5 - 12.7) | 0 | 0.0 | (0.0 - 6.7) | 0 | 0.0 | (0.0 - 6.5) | 0 | 0.0 | (0.0 - 6.6) | 0 | 0.0 | (0.0 - 6.6) | 0 | 0.0 | (0.0 - 6.6) |
| Leukopenia | 2 | 3.8 | (0.5 - 13.0) | 0 | 0.0 | (0.0 - 6.7) | 0 | 0.0 | (0.0 - 6.6) | 0 | 0.0 | (0.0 - 6.7) | 0 | 0.0 | (0.0 - 6.5) | 0 | 0.0 | (0.0 - 6.6) | 0 | 0.0 | (0.0 - 6.6) | 1 | 1.9 | (0.0 - 9.9) |
| Lymphopenia | 0 | 0.0 | (0.0 - 6.7) | 0 | 0.0 | (0.0 - 6.7) | 0 | 0.0 | (0.0 - 6.6) | 2 | 3.8 | (0.5 - 13.0) | 0 | 0.0 | (0.0 - 6.5) | 0 | 0.0 | (0.0 - 6.6) | 0 | 0.0 | (0.0 - 6.6) | 0 | 0.0 | (0.0 - 6.6) |
| Migraine | 0 | 0.0 | (0.0 - 6.7) | 0 | 0.0 | (0.0 - 6.7) | 0 | 0.0 | (0.0 - 6.6) | 0 | 0.0 | (0.0 - 6.7) | 1 | 1.8 | (0.0 - 9.7) | 0 | 0.0 | (0.0 - 6.6) | 1 | 1.9 | (0.0 - 9.9) | 0 | 0.0 | (0.0 - 6.6) |
| Mouth ulceration | 0 | 0.0 | (0.0 - 6.7) | 0 | 0.0 | (0.0 - 6.7) | 0 | 0.0 | (0.0 - 6.6) | 1 | 1.9 | (0.0 - 10.1) | 0 | 0.0 | (0.0 - 6.5) | 0 | 0.0 | (0.0 - 6.6) | 0 | 0.0 | (0.0 - 6.6) | 0 | 0.0 | (0.0 - 6.6) |
| Muscle spasms | 0 | 0.0 | (0.0 - 6.7) | 0 | 0.0 | (0.0 - 6.7) | 1 | 1.9 | (0.0 - 9.9) | 0 | 0.0 | (0.0 - 6.7) | 1 | 1.8 | (0.0 - 9.7) | 0 | 0.0 | (0.0 - 6.6) | 0 | 0.0 | (0.0 - 6.6) | 0 | 0.0 | (0.0 - 6.6) |
| Nasal congestion | 1 | 1.9 | (0.0 - 10.1) | 0 | 0.0 | (0.0 - 6.7) | 0 | 0.0 | (0.0 - 6.6) | 0 | 0.0 | (0.0 - 6.7) | 0 | 0.0 | (0.0 - 6.5) | 0 | 0.0 | (0.0 - 6.6) | 0 | 0.0 | (0.0 - 6.6) | 0 | 0.0 | (0.0 - 6.6) |
| Nausea | 0 | 0.0 | (0.0 - 6.7) | 0 | 0.0 | (0.0 - 6.7) | 1 | 1.9 | (0.0 - 9.9) | 0 | 0.0 | (0.0 - 6.7) | 0 | 0.0 | (0.0 - 6.5) | 0 | 0.0 | (0.0 - 6.6) | 1 | 1.9 | (0.0 - 9.9) | 0 | 0.0 | (0.0 - 6.6) |
| Neutropenia | 1 | 1.9 | (0.0 - 10.1) | 0 | 0.0 | (0.0 - 6.7) | 0 | 0.0 | (0.0 - 6.6) | 0 | 0.0 | (0.0 - 6.7) | 2 | 3.6 | (0.4 - 12.5) | 0 | 0.0 | (0.0 - 6.6) | 0 | 0.0 | (0.0 - 6.6) | 0 | 0.0 | (0.0 - 6.6) |
| Odynophagia | 0 | 0.0 | (0.0 - 6.7) | 0 | 0.0 | (0.0 - 6.7) | 0 | 0.0 | (0.0 - 6.6) | 1 | 1.9 | (0.0 - 10.1) | 0 | 0.0 | (0.0 - 6.5) | 0 | 0.0 | (0.0 - 6.6) | 0 | 0.0 | (0.0 - 6.6) | 0 | 0.0 | (0.0 - 6.6) |
| Oedema | 0 | 0.0 | (0.0 - 6.7) | 1 | 1.9 | (0.0 - 10.1) | 0 | 0.0 | (0.0 - 6.6) | 0 | 0.0 | (0.0 - 6.7) | 0 | 0.0 | (0.0 - 6.5) | 0 | 0.0 | (0.0 - 6.6) | 0 | 0.0 | (0.0 - 6.6) | 0 | 0.0 | (0.0 - 6.6) |
| Oral herpes | 0 | 0.0 | (0.0 - 6.7) | 0 | 0.0 | (0.0 - 6.7) | 0 | 0.0 | (0.0 - 6.6) | 0 | 0.0 | (0.0 - 6.7) | 1 | 1.8 | (0.0 - 9.7) | 0 | 0.0 | (0.0 - 6.6) | 0 | 0.0 | (0.0 - 6.6) | 0 | 0.0 | (0.0 - 6.6) |
| Oropharyngeal pain | 0 | 0.0 | (0.0 - 6.7) | 0 | 0.0 | (0.0 - 6.7) | 0 | 0.0 | (0.0 - 6.6) | 0 | 0.0 | (0.0 - 6.7) | 1 | 1.8 | (0.0 - 9.7) | 1 | 1.9 | (0.0 - 9.9) | 1 | 1.9 | (0.0 - 9.9) | 2 | 3.7 | (0.5 - 12.7) |

(to be continued)

**S. Table 17.** Proportion of participants with unsolicited adverse reactions occurring 28 days after the first vaccination, by study group (continuation).

| **Unsolicited Adverse Reactions** | **Group 1A** | | | **Group 1B** | | | **Group 1C** | | | **Group 2A** | | | | | **Group 2B** | | | | | **Group 2C** | | | | | **Group 3** | | | | | | **Group 4** | | | | |
| --- | --- | --- | --- | --- | --- | --- | --- | --- | --- | --- | --- | --- | --- | --- | --- | --- | --- | --- | --- | --- | --- | --- | --- | --- | --- | --- | --- | --- | --- | --- | --- | --- | --- | --- | --- |
|  | **15μg H7N9 + IB160** | | | **7.5μg H7N9 + IB160** | | | **3.75μg H7N9 + IB160** | | | **15μg H7N9 + SE** | | | | | **7.5μg H7N9 + SE** | | | | | **3.75μg H7N9 + SE** | | | | | **15μg H7N9, without adj** | | | | | | **Placebo** | | | | |
|  | **(n=53)** | | | **(n=53)** | | | **(n=54)** | | | **(n=53)** | | | | | **(n=55)** | | | | | **(n=54)** | | | | | **(n=54)** | | | | | | **(n=54)** | | | | |
|  | **n** | **%** | **(95%CI)** | **n** | **%** | **(95%CI)** | **n** | **%** | **(95%CI)** | **n** | | **%** | | **(95%CI)** | **n** | | **%** | | **(95%CI)** | **n** | | **%** | | **(95%CI)** | **n** | | **%** | | **(95%CI)** | | **n** | | **%** | | **(95%CI)** |
| Pain in extremity | 0 | 0.0 | (0.0 - 6.7) | 1 | 1.9 | (0.0 - 10.1) | 0 | 0.0 | (0.0 - 6.6) | 0 | 0.0 | | (0.0 - 6.7) | | 0 | 0.0 | | (0.0 - 6.5) | | 0 | 0.0 | | (0.0 - 6.6) | | 0 | 0.0 | | (0.0 - 6.6) | | 0 | | 0.0 | | (0.0 - 6.6) | |
| Paraesthesia | 0 | 0.0 | (0.0 - 6.7) | 0 | 0.0 | (0.0 - 6.7) | 0 | 0.0 | (0.0 - 6.6) | 0 | 0.0 | | (0.0 - 6.7) | | 0 | 0.0 | | (0.0 - 6.5) | | 0 | 0.0 | | (0.0 - 6.6) | | 1 | 1.9 | | (0.0 - 9.9) | | 0 | | 0.0 | | (0.0 - 6.6) | |
| Pharyngitis | 0 | 0.0 | (0.0 - 6.7) | 0 | 0.0 | (0.0 - 6.7) | 0 | 0.0 | (0.0 - 6.6) | 0 | 0.0 | | (0.0 - 6.7) | | 1 | 1.8 | | (0.0 - 9.7) | | 0 | 0.0 | | (0.0 - 6.6) | | 0 | 0.0 | | (0.0 - 6.6) | | 0 | | 0.0 | | (0.0 - 6.6) | |
| Pruritus | 0 | 0.0 | (0.0 - 6.7) | 1 | 1.9 | (0.0 - 10.1) | 0 | 0.0 | (0.0 - 6.6) | 0 | 0.0 | | (0.0 - 6.7) | | 0 | 0.0 | | (0.0 - 6.5) | | 2 | 0.0 | | (0.0 - 6.6) | | 1 | 1.9 | | (0.0 - 9.9) | | 0 | | 0.0 | | (0.0 - 6.6) | |
| Rash | 0 | 0.0 | (0.0 - 6.7) | 0 | 0.0 | (0.0 - 6.7) | 0 | 0.0 | (0.0 - 6.6) | 0 | 0.0 | | (0.0 - 6.7) | | 0 | 0.0 | | (0.0 - 6.5) | | 1 | 1.9 | | (0.0 - 9.9) | | 0 | 0.0 | | (0.0 - 6.6) | | 0 | | 0.0 | | (0.0 - 6.6) | |
| Rhinitis | 0 | 0.0 | (0.0 - 6.7) | 0 | 0.0 | (0.0 - 6.7) | 0 | 0.0 | (0.0 - 6.6) | 0 | 0.0 | | (0.0 - 6.7) | | 1 | 1.8 | | (0.0 - 9.7) | | 1 | 1.9 | | (0.0 - 9.9) | | 0 | 0.0 | | (0.0 - 6.6) | | 0 | | 0.0 | | (0.0 - 6.6) | |
| Rhinorrhoea | 0 | 0.0 | (0.0 - 6.7) | 0 | 0.0 | (0.0 - 6.7) | 0 | 0.0 | (0.0 - 6.6) | 2 | 3.8 | | (0.5 - 13.0) | | 2 | 3.6 | | (0.4 - 12.5) | | 0 | 0.0 | | (0.0 - 6.6) | | 1 | 1.9 | | (0.0 - 9.9) | | 1 | | 1.9 | | (0.0 - 9.9) | |
| Sneezing | 0 | 0.0 | (0.0 - 6.7) | 0 | 0.0 | (0.0 - 6.7) | 1 | 1.9 | (0.0 - 9.9) | 0 | 0.0 | | (0.0 - 6.7) | | 0 | 0.0 | | (0.0 - 6.5) | | 0 | 0.0 | | (0.0 - 6.6) | | 0 | 0.0 | | (0.0 - 6.6) | | 0 | | 0.0 | | (0.0 - 6.6) | |
| Somnolence | 0 | 0.0 | (0.0 - 6.7) | 0 | 0.0 | (0.0 - 6.7) | 0 | 0.0 | (0.0 - 6.6) | 0 | 0.0 | | (0.0 - 6.7) | | 1 | 1.8 | | (0.0 - 9.7) | | 2 | 0.0 | | (0.0 - 6.6) | | 0 | 0.0 | | (0.0 - 6.6) | | 2 | | 3.7 | | (0.5 - 12.7) | |
| Transaminases increased | 0 | 0.0 | (0.0 - 6.7) | 1 | 1.9 | (0.0 - 10.1) | 1 | 1.9 | (0.0 - 9.9) | 0 | 0.0 | | (0.0 - 6.7) | | 0 | 0.0 | | (0.0 - 6.5) | | 0 | 0.0 | | (0.0 - 6.6) | | 0 | 0.0 | | (0.0 - 6.6) | | 1 | | 1.9 | | (0.0 - 9.9) | |
| Upper respiratory tract infection | 0 | 0.0 | (0.0 - 6.7) | 0 | 0.0 | (0.0 - 6.7) | 0 | 0.0 | (0.0 - 6.6) | 0 | 0.0 | | (0.0 - 6.7) | | 0 | 0.0 | | (0.0 - 6.5) | | 1 | 1.9 | | (0.0 - 9.9) | | 0 | 0.0 | | (0.0 - 6.6) | | 0 | | 0.0 | | (0.0 - 6.6) | |
| Vomiting | 1 | 1.9 | (0.0 - 10.1) | 0 | 0.0 | (0.0 - 6.7) | 0 | 0.0 | (0.0 - 6.6) | 1 | 1.9 | | (0.0 - 10.1) | | 0 | 0.0 | | (0.0 - 6.5) | | 0 | 0.0 | | (0.0 - 6.6) | | 0 | 0.0 | | (0.0 - 6.6) | | 0 | | 0.0 | | (0.0 - 6.6) | |
| Others | 2 | 3.8 | (0.5 - 13.0) | 4 | 7.5 | (2.1 - 18.2) | 2 | 3.7 | (0.5 - 12.7) | 1 | 1.9 | | (0.0 - 10.1) | | 1 | 1.8 | | (0.0 - 9.7) | | 1 | 1.9 | | (0.0 - 9.9) | | 1 | 1.9 | | (0.0 - 9.9) | | 4 | | 7.4 | | (2.1 - 17.9) | |
| **TOTAL** | 9 | 17.0 | (8.1 - 29.8) | 9 | 17.0 | (8.1 - 29.8) | 8 | 14.8 | (6.6 - 29.8) | 9 | 17.0 | | (8.1 - 29.8) | | 15 | 27.3 | | (16.1 - 41.0) | | 14 | 25.9 | | (15.0 - 39.7) | | 9 | 16.7 | | (7.9 - 29.3) | | 15 | | 27.8 | | (16.5 - 41.6) | |
| adj: adjuvant; 95%CI: 95% confidence interval | | | | | | | | | | | | | | | | | | | | | | | | | | | | | | | | | | | |

(end)

1. **S. Table 18.** Proportion of participants with unsolicited adverse reactions occurring 28 days after the second vaccination, by study group.

| **Unsolicited Adverse Reactions** | **Group 1A** | | | **Group 1B** | | | **Group 1C** | | | **Group 2A** | | | **Group 2B** | | | **Group 2C** | | | **Group 3** | | | **Group 4** | | |
| --- | --- | --- | --- | --- | --- | --- | --- | --- | --- | --- | --- | --- | --- | --- | --- | --- | --- | --- | --- | --- | --- | --- | --- | --- |
|  | **15μg H7N9 + IB160** | | | **7.5μg H7N9 + IB160** | | | **3.75μg H7N9 + IB160** | | | **15μg H7N9 + SE** | | | **7.5μg H7N9 + SE** | | | **3.75μg H7N9 + SE** | | | **15μg H7N9, without adj** | | | **Placebo** | | |
|  | **(n=53)** | | | **(n=53)** | | | **(n=54)** | | | **(n=53)** | | | **(n=55)** | | | **(n=54)** | | | **(n=54)** | | | **(n=54)** | | |
|  | **n** | **%** | **(95%CI)** | **n** | **%** | **(95%CI)** | **n** | **%** | **(95%CI)** | **n** | **%** | **(95%CI)** | **n** | **%** | **(95%CI)** | **n** | **%** | **(95%CI)** | **n** | **%** | **(95%CI)** | **n** | **%** | **(95%CI)** |
| Administration site haematoma | 1 | 2.0 | (0.1 - 10.6) | 1 | 2.1 | (0.1 - 11.1) | 0 | 0.0 | (0.0 - 7.0) | 0 | 0.0 | (0.0 - 7.4) | 0 | 0.0 | (0.0 - 6.7) | 0 | 0.0 | (0.0 - 7.0) | 1 | 2.0 | (0.0 - 10.4) | 0 | 0.0 | (0.0 - 7.4) |
| Administration site haemorrhage | 1 | 2.0 | (0.1 - 10.6) | 0 | 0.0 | (0.0 - 7.4) | 0 | 0.0 | (0.0 - 7.0) | 0 | 0.0 | (0.0 - 7.4) | 0 | 0.0 | (0.0 - 6.7) | 0 | 0.0 | (0.0 - 7.0) | 0 | 0.0 | (0.0 - 7.0) | 1 | 2.1 | (0.1 - 11.1) |
| Administration site paraesthesia | 1 | 2.0 | (0.1 - 10.6) | 0 | 0.0 | (0.0 - 7.4) | 0 | 0.0 | (0.0 - 7.0) | 0 | 0.0 | (0.0 - 7.4) | 0 | 0.0 | (0.0 - 6.7) | 0 | 0.0 | (0.0 - 7.0) | 0 | 0.0 | (0.0 - 7.0) | 0 | 0.0 | (0.0 - 7.4) |
| Alanine aminotransferase increased | 2 | 4.0 | (0.5 - 13.7) | 1 | 2.1 | (0.1 - 11.1) | 0 | 0.0 | (0.0 - 7.0) | 2 | 4.2 | (0.5 - 14.3) | 1 | 1.9 | (0.0 - 10.1) | 2 | 3.9 | (0.5 - 13.5) | 0 | 0.0 | (0.0 - 7.0) | 0 | 0.0 | (0.0 - 7.4) |
| Anaemia | 1 | 2.0 | (0.1 - 10.6) | 0 | 0.0 | (0.0 - 7.4) | 1 | 2.0 | (0.0 - 10.4) | 1 | 2.1 | (0.1 - 11.1) | 0 | 0.0 | (0.0 - 6.7) | 0 | 0.0 | (0.0 - 7.0) | 0 | 0.0 | (0.0 - 7.0) | 0 | 0.0 | (0.0 - 7.4) |
| Back pain | 0 | 0.0 | (0.0 - 7.1) | 0 | 0.0 | (0.0 - 7.4) | 0 | 0.0 | (0.0 - 7.0) | 0 | 0.0 | (0.0 - 7.4) | 0 | 0.0 | (0.0 - 6.7) | 0 | 0.0 | (0.0 - 7.0) | 0 | 0.0 | (0.0 - 7.0) | 1 | 2.1 | (0.1 - 11.1) |
| Blood bilirubin increased | 1 | 2.0 | (0.1 - 10.6) | 0 | 0.0 | (0.0 - 7.4) | 1 | 2.0 | (0.0 - 10.4) | 0 | 0.0 | (0.0 - 7.4) | 0 | 0.0 | (0.0 - 6.7) | 1 | 2.0 | (0.0 - 10.4) | 1 | 2.0 | (0.0 - 10.4) | 0 | 0.0 | (0.0 - 7.4) |
| Dizziness | 0 | 0.0 | (0.0 - 7.1) | 1 | 2.1 | (0.1 - 11.1) | 0 | 0.0 | (0.0 - 7.0) | 1 | 2.1 | (0.1 - 11.1) | 2 | 3.8 | (0.5 - 13.0) | 0 | 0.0 | (0.0 - 7.0) | 1 | 2.0 | (0.0 - 10.4) | 0 | 0.0 | (0.0 - 7.4) |
| Dry throat | 0 | 0.0 | (0.0 - 7.1) | 0 | 0.0 | (0.0 - 7.4) | 0 | 0.0 | (0.0 - 7.0) | 0 | 0.0 | (0.0 - 7.4) | 1 | 1.9 | (0.0 - 10.1) | 0 | 0.0 | (0.0 - 7.0) | 0 | 0.0 | (0.0 - 7.0) | 0 | 0.0 | (0.0 - 7.4) |
| Dysphonia | 0 | 0.0 | (0.0 - 7.1) | 0 | 0.0 | (0.0 - 7.4) | 1 | 2.0 | (0.0 - 10.4) | 0 | 0.0 | (0.0 - 7.4) | 0 | 0.0 | (0.0 - 6.7) | 0 | 0.0 | (0.0 - 7.0) | 0 | 0.0 | (0.0 - 7.0) | 0 | 0.0 | (0.0 - 7.4) |
| Eye pain | 0 | 0.0 | (0.0 - 7.1) | 1 | 2.1 | (0.1 - 11.1) | 0 | 0.0 | (0.0 - 7.0) | 0 | 0.0 | (0.0 - 7.4) | 0 | 0.0 | (0.0 - 6.7) | 0 | 0.0 | (0.0 - 7.0) | 0 | 0.0 | (0.0 - 7.0) | 0 | 0.0 | (0.0 - 7.4) |
| Headache | 0 | 0.0 | (0.0 - 7.1) | 0 | 0.0 | (0.0 - 7.4) | 1 | 2.0 | (0.0 - 10.4) | 0 | 0.0 | (0.0 - 7.4) | 0 | 0.0 | (0.0 - 6.7) | 0 | 0.0 | (0.0 - 7.0) | 0 | 0.0 | (0.0 - 7.0) | 0 | 0.0 | (0.0 - 7.4) |
| Influenza | 0 | 0.0 | (0.0 - 7.1) | 0 | 0.0 | (0.0 - 7.4) | 0 | 0.0 | (0.0 - 7.0) | 0 | 0.0 | (0.0 - 7.4) | 1 | 1.9 | (0.0 - 10.1) | 0 | 0.0 | (0.0 - 7.0) | 1 | 2.0 | (0.0 - 10.4) | 0 | 0.0 | (0.0 - 7.4) |
| Leukopenia | 0 | 0.0 | (0.0 - 7.1) | 0 | 0.0 | (0.0 - 7.4) | 0 | 0.0 | (0.0 - 7.0) | 2 | 4.2 | (0.5 - 14.3) | 0 | 0.0 | (0.0 - 6.7) | 0 | 0.0 | (0.0 - 7.0) | 0 | 0.0 | (0.0 - 7.0) | 1 | 2.1 | (0.1 - 11.1) |
| Lymphopenia | 0 | 0.0 | (0.0 - 7.1) | 0 | 0.0 | (0.0 - 7.4) | 0 | 0.0 | (0.0 - 7.0) | 1 | 2.1 | (0.1 - 11.1) | 0 | 0.0 | (0.0 - 6.7) | 0 | 0.0 | (0.0 - 7.0) | 0 | 0.0 | (0.0 - 7.0) | 0 | 0.0 | (0.0 - 7.4) |
| Musculoskeletal pain | 0 | 0.0 | (0.0 - 7.1) | 0 | 0.0 | (0.0 - 7.4) | 0 | 0.0 | (0.0 - 7.0) | 0 | 0.0 | (0.0 - 7.4) | 0 | 0.0 | (0.0 - 6.7) | 0 | 0.0 | (0.0 - 7.0) | 1 | 2.0 | (0.0 - 10.4) | 0 | 0.0 | (0.0 - 7.4) |
| Neutropenia | 0 | 0.0 | (0.0 - 7.1) | 0 | 0.0 | (0.0 - 7.4) | 0 | 0.0 | (0.0 - 7.0) | 1 | 2.1 | (0.1 - 11.1) | 0 | 0.0 | (0.0 - 6.7) | 0 | 0.0 | (0.0 - 7.0) | 2 | 3.9 | (0.5 - 13.5) | 2 | 4.2 | (0.5 - 14.3) |

(to be continued)

**S. Table 18.** Proportion of participants with unsolicited adverse reactions occurring 28 days after the second vaccination, by study group (continuation).

| **Unsolicited Adverse Reactions** | **Group 1A** | | | **Group 1B** | | | **Group 1C** | | | **Group 2A** | | | **Group 2B** | | | **Group 2C** | | | **Group 3** | | | **Group 4** | | |
| --- | --- | --- | --- | --- | --- | --- | --- | --- | --- | --- | --- | --- | --- | --- | --- | --- | --- | --- | --- | --- | --- | --- | --- | --- |
|  | **15μg H7N9 + IB160** | | | **7.5μg H7N9 + IB160** | | | **3.75μg H7N9 + IB160** | | | **15μg H7N9 + SE** | | | **7.5μg H7N9 + SE** | | | **3.75μg H7N9 + SE** | | | **15μg H7N9, without adj** | | | **Placebo** | | |
|  | **(n=53)** | | | **(n=53)** | | | **(n=54)** | | | **(n=53)** | | | **(n=55)** | | | **(n=54)** | | | **(n=54)** | | | **(n=54)** | | |
|  | **n** | **%** | **(95%CI)** | **n** | **%** | **(95%CI)** | **n** | **%** | **(95%CI)** | **n** | **%** | **(95%CI)** | **n** | **%** | **(95%CI)** | **n** | **%** | **(95%CI)** | **n** | **%** | **(95%CI)** | **n** | **%** | **(95%CI)** |
| Odynophagia | 1 | 2.0 | (0.1 - 10.6) | 1 | 2.1 | (0.1 - 11.1) | 1 | 2.0 | (0.0 - 10.4) | 0 | 0.0 | (0.0 - 7.4) | 0 | 0.0 | (0.0 - 6.7) | 0 | 0.0 | (0.0 - 7.0) | 0 | 0.0 | (0.0 - 7.0) | 0 | 0.0 | (0.0 - 7.4) |
| Oropharyngeal pain | 1 | 2.0 | (0.1 - 10.6) | 1 | 2.1 | (0.1 - 11.1) | 0 | 0.0 | (0.0 - 7.0) | 0 | 0.0 | (0.0 - 7.4) | 0 | 0.0 | (0.0 - 6.7) | 0 | 0.0 | (0.0 - 7.0) | 0 | 0.0 | (0.0 - 7.0) | 0 | 0.0 | (0.0 - 7.4) |
| Pain in extremity | 0 | 0.0 | (0.0 - 7.1) | 0 | 0.0 | (0.0 - 7.4) | 0 | 0.0 | (0.0 - 7.0) | 0 | 0.0 | (0.0 - 7.4) | 0 | 0.0 | (0.0 - 6.7) | 0 | 0.0 | (0.0 - 7.0) | 0 | 0.0 | (0.0 - 7.0) | 1 | 2.1 | (0.1 - 11.1) |
| Papule | 0 | 0.0 | (0.0 - 7.1) | 0 | 0.0 | (0.0 - 7.4) | 0 | 0.0 | (0.0 - 7.0) | 0 | 0.0 | (0.0 - 7.4) | 0 | 0.0 | (0.0 - 6.7) | 1 | 2.0 | (0.0 - 10.4) | 0 | 0.0 | (0.0 - 7.0) | 0 | 0.0 | (0.0 - 7.4) |
| Paraesthesia | 1 | 2.0 | (0.1 - 10.6) | 0 | 0.0 | (0.0 - 7.4) | 0 | 0.0 | (0.0 - 7.0) | 0 | 0.0 | (0.0 - 7.4) | 0 | 0.0 | (0.0 - 6.7) | 0 | 0.0 | (0.0 - 7.0) | 0 | 0.0 | (0.0 - 7.0) | 0 | 0.0 | (0.0 - 7.4) |
| Rhinorrhoea | 0 | 0.0 | (0.0 - 7.1) | 0 | 0.0 | (0.0 - 7.4) | 1 | 2.0 | (0.0 - 10.4) | 0 | 0.0 | (0.0 - 7.4) | 0 | 0.0 | (0.0 - 6.7) | 0 | 0.0 | (0.0 - 7.0) | 0 | 0.0 | (0.0 - 7.0) | 0 | 0.0 | (0.0 - 7.4) |
| Sneezing | 0 | 0.0 | (0.0 - 7.1) | 0 | 0.0 | (0.0 - 7.4) | 1 | 2.0 | (0.0 - 10.4) | 0 | 0.0 | (0.0 - 7.4) | 0 | 0.0 | (0.0 - 6.7) | 0 | 0.0 | (0.0 - 7.0) | 0 | 0.0 | (0.0 - 7.0) | 0 | 0.0 | (0.0 - 7.4) |
| Somnolence | 0 | 0.0 | (0.0 - 7.1) | 0 | 0.0 | (0.0 - 7.4) | 0 | 0.0 | (0.0 - 7.0) | 0 | 0.0 | (0.0 - 7.4) | 0 | 0.0 | (0.0 - 6.7) | 1 | 2.0 | (0.0 - 10.4) | 0 | 0.0 | (0.0 - 7.0) | 0 | 0.0 | (0.0 - 7.4) |
| Throat irritation | 0 | 0.0 | (0.0 - 7.1) | 0 | 0.0 | (0.0 - 7.4) | 0 | 0.0 | (0.0 - 7.0) | 1 | 2.1 | (0.1 - 11.1) | 0 | 0.0 | (0.0 - 6.7) | 0 | 0.0 | (0.0 - 7.0) | 0 | 0.0 | (0.0 - 7.0) | 0 | 0.0 | (0.0 - 7.4) |
| Transaminases increased | 0 | 0.0 | (0.0 - 7.1) | 0 | 0.0 | (0.0 - 7.4) | 0 | 0.0 | (0.0 - 7.0) | 1 | 2.1 | (0.1 - 11.1) | 1 | 1.9 | (0.0 - 10.1) | 1 | 2.0 | (0.0 - 10.4) | 0 | 0.0 | (0.0 - 7.0) | 1 | 2.1 | (0.1 - 11.1) |
| Others | 1 | 2.0 | (0.1 - 10.6) | 5 | 10.4 | (3.5 - 22.7) | 2 | 3.9 | (0.5 - 13.5) | 0 | 0.0 | (0.0 - 7.4) | 3 | 5.7 | (1.2 - 15.7) | 0 | 0.0 | (0.0 - 7.0) | 3 | 5.9 | (1.2 - 16.2) | 1 | 2.1 | (0.1 - 11.1) |
| **TOTAL** | 8 | 16.0 | (7.2 - 29.1) | 10 | 20.8 | (10.5 - 35.0) | 7 | 13.7 | (5.7 - 26.3) | 8 | 16.7 | (7.5 - 30.2) | 7 | 13.2 | (5.5 - 25.3) | 6 | 11.8 | (4.4 - 23.9) | 10 | 19.6 | (9.8 - 33.1) | 6 | 12.5 | (4.7 - 25.2) |
| adj: adjuvant; 95%CI: 95% confidence interval | | | | | | | | | | | | | | | | | | | | | | | | |

(end)

|  | **Group 1A** | | | **Group 1B** | | | **Group 1C** | | | **Group 2A** | | | **Group 2B** | | | **Group 2C** | | | **Group 3** | | | **Group 4** | | |
| --- | --- | --- | --- | --- | --- | --- | --- | --- | --- | --- | --- | --- | --- | --- | --- | --- | --- | --- | --- | --- | --- | --- | --- | --- |
|  | **15μ H7N9 + IB160** | | | **7,5μ H7N9 + IB160** | | | **3,75μ H7N9 + IB160** | | | **15μ H7N9 + SE** | | | **7,5μ H7N9 + SE** | | | **3,75μ H7N9 + SE** | | | **15μ H7N9, without adj** | | | **Placebo** | | |
|  | **n** | **Value** | **(95%CI)** | **n** | **Value** | **(95%CI)** | **n** | **Value** | **(95%CI)** | **n** | **Value** | **(95%CI)** | **n** | **Value** | **(95%CI)** | **n** | **Value** | **(95%CI)** | **n** | **Value** | **(95%CI)** | **n** | **Value** | **(95%CI)** |
| **GMT** |  |  |  |  |  |  |  |  |  |  |  |  |  |  |  |  |  |  |  |  |  |  |  |  |
| Pre | 51 | 5.0 | (NE) | 47 | 5.0 | (NE) | 54 | 5.0 | (NE) | 49 | 5.0 | (NE) | 54 | 5.0 | (NE) | 51 | 5.0 | (NE) | 52 | 5.0 | (NE) | 49 | 5.0 | (NE) |
| d28 | 51 | 6.0 | (5.4 - 6.8) | 47 | 5.8 | (5.2 - 6.4) | 54 | 5.8 | (5.2 - 6.5) | 49 | 5.8 | (5.2 - 6.6) | 53 | 5.5 | (5.0 - 6.1) | 51 | 5.3 | (4.9 - 5.6) | 52 | 5.3 | (4.8 - 5.7) | 49 | 5.1 | (4.9 - 5.2) |
| d35 | 50 | 12.1 | (9.8 - 15.0) | 45 | 14.0 | (10.6 - 18.6) | 52 | 11.3 | (8.7 - 14.7) | 48 | 11.1 | (8.4 - 14.6) | 53 | 7.0 | (5.8 - 8.5) | 51 | 7.8 | (6.3 - 9.7) | 52 | 5.9 | (5.1 - 6.9) | 46 | 5.1 | (4.9 - 5.2) |
| d56 | 50 | 13.8 | (10.7 - 17.8) | 44 | 19.1 | (13.8 - 26.3) | 53 | 14.4 | (10.9 - 19.1) | 48 | 13.3 | (9.7 - 18.4) | 54 | 8.7 | (7.0 - 10.8) | 51 | 8.5 | (6.8 - 10.6) | 52 | 6.1 | (5.3 - 7.1) | 48 | 5.0 | (NE) |
| **GMFR (from Pre)** | | | | | | | | | | | | | | | | | | | | | | | | |
| d28 | 51 | 1.2 | (1.1 - 1.4) | 47 | 1.2 | (1.0 - 1.3) | 54 | 1.2 | (1.0 - 1.3) | 49 | 1.2 | (1.0 - 1.3) | 53 | 1.1 | (1.0 - 1.2) | 51 | 1.1 | (1.0 - 1.1) | 52 | 1.1 | (1.0 - 1.1) | 49 | 1.0 | (1.0 - 1.0) |
| d35 | 50 | 2.4 | (2.0 - 3.0) | 45 | 2.8 | (2.1 - 3.7) | 52 | 2.3 | (1.7 - 2.9) | 48 | 2.2 | (1.7 - 2.9) | 53 | 1.4 | (1.2 - 1.7) | 51 | 1.6 | (1.3 - 1.9) | 52 | 1.2 | (1.0 - 1.4) | 46 | 1.0 | (1.0 - 1.0) |
| d56 | 50 | 2.8 | (2.1 - 3.6) | 44 | 3.8 | (2.8 - 5.3) | 53 | 2.9 | (2.2 - 3.8) | 48 | 2.7 | (1.9 - 3.7) | 54 | 1.7 | (1.4 - 2.2) | 51 | 1.7 | (1.4 - 2.1) | 52 | 1.2 | (1.1 - 1.4) | 48 | 1.0 | (1.0 - 1.0) |
| **SPR (%)** | | | | | | | | | | | | | | | | | | | | | | | | |
| Pre | 51 | 0.0 | (0.0 - 7.0) | 47 | 0.0 | (0.0 - 7.5) | 54 | 0.0 | (0.0 - 6.6) | 49 | 0.0 | (0.0 - 7.3) | 54 | 0.0 | (0.0 - 6.6) | 51 | 0.0 | (0.0 - 7.0) | 52 | 0.0 | (0.0 - 6.8) | 49 | 0.0 | (0.0 - 7.3) |
| d28 | 51 | 0.0 | (0.0 - 7.0) | 47 | 0.0 | (0.0 - 7.5) | 54 | 0.0 | (0.0 - 6.6) | 49 | 2.0 | (0.0 - 10.9) | 53 | 1.9 | (0.0 - 10.1) | 51 | 0.0 | (0.0 - 7.0) | 52 | 1.9 | (0.0 - 10.3) | 49 | 0.0 | (0.0 - 7.3) |
| d35 | 50 | 14.0 | (5.8 - 26.7) | 45 | 22.2 | (11.2 - 37.1) | 52 | 23.1 | (12.5 - 36.8) | 48 | 14.6 | (6.1 - 27.8) | 53 | 5.7 | (1.2 - 15.7) | 51 | 7.8 | (2.2 - 18.9) | 52 | 3.8 | (0.5 - 13.2) | 46 | 0.0 | (0.0 - 7.7) |
| d56 | 50 | 20.0 | (10.0 - 33.7) | 44 | 36.4 | (22.4 - 52.2) | 53 | 32.1 | (19.9 - 46.3) | 48 | 20.8 | (10.5 - 35.0) | 54 | 9.3 | (3.1 - 20.3) | 51 | 9.8 | (3.3 - 21.4) | 52 | 3.8 | (0.5 - 13.2) | 48 | 0.0 | (0.0 - 7.4) |
| **SCR (from Pre - %)** | | | | | | | | | | | | | | | | | | | | | | | | |
| d28 | 51 | 0.0 | (0.0 - 7.0) | 47 | 0.0 | (0.0 - 7.5) | 54 | 0.0 | (0.0 - 6.6) | 49 | 2.0 | (0.0 - 10.9) | 53 | 1.9 | (0.0 - 10.1) | 51 | 0.0 | (0.0 - 7.0) | 52 | 1.9 | (0.0 - 10.3) | 49 | 0.0 | (0.0 - 7.3) |
| d35 | 50 | 14.0 | (5.8 - 26.7) | 45 | 22.2 | (11.2 - 37.1) | 52 | 23.1 | (12.5 - 36.8) | 48 | 14.6 | (6.1 - 27.8) | 53 | 5.7 | (1.2 - 15.7) | 51 | 7.8 | (2.2 - 18.9) | 52 | 3.8 | (0.5 - 13.2) | 46 | 0.0 | (0.0 - 7.7) |
| d56 | 50 | 20.0 | (10.0 - 33.7) | 44 | 36.4 | (22.4 - 52.2) | 53 | 32.1 | (19.9 - 46.3) | 48 | 20.8 | (10.5 - 35.0) | 54 | 9.3 | (3.1 - 20.3) | 51 | 9.8 | (3.3 - 21.4) | 52 | 3.8 | (0.5 - 13.2) | 48 | 0.0 | (0.0 - 7.4) |
| adj: adjuvant; 95%CI: 95% confidence interval; GMT: geometric mean titters; GMFR: geometric mean fold rises, i.e., ratio between GMT of baseline and of post-dose; SPR: seroprotection rate (prior and postvaccination HI antibody titers ≥1:40); SCR: seroconversion rate (baseline HI antibody titers <1:10 and postvaccination HI antibody titers ≥1:40, or baseline HI antibody titers ≥1:10 and a postvaccination increase by a factor of four or more); Sample collected at: Pre (prior 1st dose), d28 (prior 2nd dose, 28 days post 1st dose), d35 (7 days post 2nd dose or 35 days post 1st dose), d56 (28 days post 2nd dose or 56 days post 1st dose); NE: not estimable. | | | | | | | | | | | | | | | | | | | | | | | | |

**S. Table 19.** Hemagglutination inhibition (HI) data by study group, intention-to-treat analysis.

**S. Table 20.** Immunogenicity of serum antibody to Influenza H7N9 virus detected by microneutralization assay by study group, intention-to-treat analysis.

|  | **Group 1A** | | | **Group 1B** | | | **Group 1C** | | | **Group 2A** | | | **Group 2B** | | | **Group 2C** | | | **Group 3** | | | **Group 4** | | |
| --- | --- | --- | --- | --- | --- | --- | --- | --- | --- | --- | --- | --- | --- | --- | --- | --- | --- | --- | --- | --- | --- | --- | --- | --- |
|  | **15μ H7N9 + adj IB160** | | | **7,5μ H7N9 + adj IB160** | | | **3,75μ H7N9 + adj IB160** | | | **15μ H7N9 + adj SE** | | | **7,5μ H7N9 + adj SE** | | | **3,75μ H7N9 + adj SE** | | | **15μ H7N9, without adj** | | | **Placebo** | | |
|  | **n** | **Value** | **(95%CI)** | **n** | **Value** | **(95%CI)** | **n** | **Value** | **(95%CI)** | **n** | **Value** | **(95%CI)** | **n** | **Value** | **(95%CI)** | **n** | **Value** | **(95%CI)** | **n** | **Value** | **(95%CI)** | **n** | **Value** | **(95%CI)** |
| **GMT** |  |  |  |  |  |  |  |  |  |  |  |  |  |  |  |  |  |  |  |  |  |  |  |  |
| Pre | 51 | 8.6 | (7.6 - 9.7) | 47 | 7.4 | (6.4 - 8.5) | 54 | 9.0 | (7.8 - 10.4) | 49 | 8.1 | (7.0 - 9.4) | 54 | 7.5 | (6.6 - 8.6) | 51 | 8.5 | (7.2 - 10.0) | 52 | 8.3 | (7.2 - 9.6) | 49 | 7.5 | (6.6 - 8.6) |
| d28 | 50 | 15.0 | (12.0 - 18.7) | 47 | 13.2 | (10.3 - 17.0) | 53 | 12.9 | (10.0 - 16.7) | 49 | 11.0 | (8.9 - 13.5) | 53 | 9.7 | (8.2 - 11.4) | 51 | 8.6 | (7.1 - 10.3) | 52 | 8.7 | (7.5 - 10.0) | 49 | 6.1 | (5.6 - 6.7) |
| d56 | 50 | 67.3 | (50.4 - 89.8) | 44 | 78.6 | (52.0 - 118.8) | 53 | 67.8 | (44.6 - 103.2) | 48 | 61.7 | (41.8 - 91.1) | 54 | 28.5 | (20.3 - 39.9) | 51 | 28.1 | (20.3 - 38.9) | 52 | 17.0 | (13.2 - 21.9) | 48 | 5.9 | (5.4 - 6.6) |
| **GMFR (from Pre)** | | | | | | | | | | | | | | | | | | | | | | | | |
| d28 | 50 | 1.7 | (1.4 - 2.2) | 47 | 1.8 | (1.4 - 2.2) | 53 | 1.4 | (1.1 - 1.8) | 49 | 1.4 | (1.1 - 1.7) | 53 | 1.3 | (1.1 - 1.5) | 51 | 1.0 | (0.8 - 1.2) | 52 | 1.0 | (0.9 - 1.2) | 49 | 0.8 | (0.7 - 0.9) |
| d56 | 50 | 7.8 | (5.9 - 10.4) | 44 | 10.4 | (6.8 - 15.9) | 53 | 7.5 | (4.9 - 11.4) | 48 | 7.8 | (5.3 - 11.5) | 54 | 3.8 | (2.7 - 5.3) | 51 | 3.3 | (2.2 - 4.9) | 52 | 2.1 | (1.6 - 2.6) | 48 | 0.8 | (0.7 - 0.9) |
| **SPR (%)** | | | | | | | | | | | | | | | | | | | | | | | | |
| Pre | 51 | 0.0 | (0.0 - 7.0) | 47 | 0.0 | (0.0 - 7.5) | 54 | 0.0 | (0.0 - 6.6) | 49 | 2.0* | (0.0 - 10.9) | 54 | 0.0 | (0.0 - 6.6) | 51 | 0.0 | (0.0 - 7.0) | 52 | 0.0 | (0.0 - 6.8) | 49 | 0.0 | (0.0 - 7.3) |
| d28 | 50 | 22.0 | (11.5 - 36.0) | 47 | 17.0 | (7.6 - 30.8) | 53 | 15.1 | (6.7 - 27.6) | 49 | 8.2 | (2.3 - 19.6) | 53 | 3.8 | (0.5 - 13.0) | 51 | 5.9 | (1.2 - 16.2) | 52 | 1.9 | (0.0 - 10.3) | 49 | 0.0 | (0.0 - 7.3) |
| d56 | 50 | 80.0 | (66.3 - 90.0) | 44 | 75.0 | (59.7 - 86.8) | 53 | 66.0 | (51.7 - 78.5) | 48 | 70.8 | (55.9 - 83.0) | 54 | 44.4 | (30.9 - 58.6) | 51 | 41.2 | (27.6 - 55.8) | 52 | 26.9 | (15.6 - 41.0) | 48 | 0.0 | (0.0 - 7.3) |
| **SCR (from Pre - %)** | | | | | | | | | | | | | | | | | | | | | | | | |
| d28^‡^ | 50 | 18.0 | (8.6 - 31.4) | 47 | 12.8 | (4.8 - 25.7) | 53 | 13.2 | (5.5 - 25.3) | 49 | 8.2 | (2.3 - 19.6) | 53 | 1.9 | (0.0 - 10.1) | 51 | 2.0 | (0.0 - 10.4) | 52 | 0.0 | (0.0 - 6.8) | 49 | 0.0 | (0.0 - 7.3) |
| d56^‡‡^ | 50 | 78.0 | (64.0 - 88.5) | 44 | 75.0 | (59.7 - 86.8) | 53 | 60.4 | (46.0 - 73.5) | 48 | 68.8 | (53.7 - 81.30 | 54 | 38.9 | (25.9 - 53.1) | 51 | 33.3 | (20.8 - 47.9) | 52 | 25.0 | (14.0 - 38.9) | 48 | 0.0 | (0.0 - 7.3) |
| adj: adjuvant; 95%CI: 95% confidence interval; GMT: geometric mean titters; GMFR: geometric mean fold rises, i.e., ratio between GMT of baseline and of post-dose; SPR: seroprotection rate (prior and postvaccination HI antibody titers ≥1:40); SCR: seroconversion rate (baseline HI antibody titers <1:10 and postvaccination HI antibody titers ≥1:40, or baseline HI antibody titers ≥1:10 and a postvaccination increase by a factor of four or more); Sample collected at: Pre (prior 1st dose), d28 (prior 2nd dose, 28 days post 1st dose), d35 (7 days post 2nd dose or 35 days post 1st dose), d56 (28 days post 2nd dose or 56 days post 1st dose); NE: not estimable.*This percentage represents one single individual which presented the following titers: pre=40, d28=20, d56=320. | | | | | | | | | | | | | | | | | | | | | | | | |

|  | ***p* values - Bonferroni Correction** | | | | | | | | | | | | | |
| --- | --- | --- | --- | --- | --- | --- | --- | --- | --- | --- | --- | --- | --- | --- |
|  | **Intention To Treat** | | | | | | | | | | | | | |
| Intervention groups | **HI** | | | | | | | | | | | | | |
|  | **GMT** | | | | | | | | | | | | | |
|  | **d35** | | | | | | | **d56** | | | | | | |
|  | Intervention groups | | | | | | | | | | | | | |
|  | **1A** | **1B** | **1C** | **2A** | 2B | **2C** | **3** | **1A** | **1B** | **1C** | **2A** | **2B** | **2C** | **3** |
| **Group 1B** | >0.999 |  |  |  |  |  |  | 0.987 |  |  |  |  |  |  |
| **Group 1C** | 0.914 | 0.911 |  |  |  |  |  | >0.999 | 0.973 |  |  |  |  |  |
| **Group 2A** | 0.990 | 0.988 | >0.999 |  |  |  |  | >0.999 | 0.741 | >0.999 |  |  |  |  |
| **Group 2B** | **<0.001** | **<0.001** | **0.033** | **0.016** |  |  |  | 0.065 | **<0.001** | 0.070 | 0.335 |  |  |  |
| **Group 2C** | **0.001** | **0.001** | 0.316 | 0.186 | >0.999 |  |  | **0.046** | **<0.001** | **0.049** | 0.255 | >0.999 |  |  |
| **Group 3** | **<0.001** | **<0.001** | **<0.001** | **<0.001** | 0.982 | 0.514 |  | **<0.001** | **<0.001** | **<0.001** | **<0.001** | 0.185 | 0.298 |  |
| **Group 4** | **<0.001** | **<0.001** | **<0.001** | **<0.001** | 0.249 | **0.030** | >0.999 | **<0.001** | **<0.001** | **<0.001** | **<0.001** | **<0.001** | **0.002** | 0.961 |
|  | **SPR** | | | | | | | | | | | | | |
|  | **1A** | **1B** | **1C** | **2A** | **2B** | **2C** | **3** | **1A** | **1B** | **1C** | **2A** | **2B** | **2C** | **3** |
| **Group 1B** | >0.999 |  |  |  |  |  |  | 0.704 |  |  |  |  |  |  |
| **Group 1C** | >0.999 | >0.999 |  |  |  |  |  | >0.999 | >0.999 |  |  |  |  |  |
| **Group 2A** | >0.999 | >0.999 | >0.999 |  |  |  |  | >0.999 | 0.987 | >0.999 |  |  |  |  |
| **Group 2B** | >0.999 | 0.246 | 0.119 | >0.999 |  |  |  | >0.999 | **0.005** | **0.025** | >0.999 |  |  |  |
| **Group 2C** | >0.999 | 0.672 | 0.368 | >0.999 | >0.999 |  |  | >0.999 | **0.008** | **0.038** | >0.999 | >0.999 |  |  |
| **Group 3** | >0.999 | 0.107 | **0.048** | >0.999 | >0.999 | >0.999 |  | 0.590 | **<0.001** | **0.001** | 0.460 | >0.999 | >0.999 |  |
| **Group 4** | 0.778 | **0.020** | **0.008** | 0.651 | >0.999 | >0.999 | >0.999 | 0.145 | **<0.001** | **<0.001** | 0.111 | >0.999 | >0.999 | >0.999 |

**S. Chart 1.** Hemagglutination inhibition (GMT and SPR) pairwise comparisons for intention-to-treat analysis.

|  | ***p* values - Bonferroni Correction** | | | | | | | | | | | | | |
| --- | --- | --- | --- | --- | --- | --- | --- | --- | --- | --- | --- | --- | --- | --- |
|  | **Per Protocol** | | | | | | | | | | | | | |
| Intervention groups | **HI** | | | | | | | | | | | | | |
|  | **GMT** | | | | | | | | | | | | | |
|  | **d35** | | | | | | | **d56** | | | | | | |
|  | Intervention groups | | | | | | | | | | | | | |
|  | **1A** | **1B** | **1C** | **2A** | **2B** | **2C** | **3** | **1A** | **1B** | **1C** | **2A** | **2B** | **2C** | **3** |
| **Group 1B** | >0.999 |  |  |  |  |  |  | 0.996 |  |  |  |  |  |  |
| **Group 1C** | 0.984 | 0.621 |  |  |  |  |  | >0.999 | 0.787 |  |  |  |  |  |
| **Group 2A** | 0.977 | 0.596 | >0.999 |  |  |  |  | 0.969 | 0.125 | >0.999 |  |  |  |  |
| **Group 2B** | **<0.001** | **<0.001** | **0.021** | **0.038** |  |  |  | **0.002** | **<0.001** | **0.015** | 0.371 |  |  |  |
| **Group 2C** | **0.002** | **<0.001** | 0.198 | 0.288 | >0.999 |  |  | **0.003** | **<0.001** | **0.018** | 0.403 | >0.999 |  |  |
| **Group 3** | **<0.001** | **<0.001** | **0.002** | **0.005** | >0.999 | 0.999 |  | **<0.001** | **<0.001** | **<0.001** | **0.003** | 0.987 | 0.985 |  |
| **Group 4** | **<0.001** | **<0.001** | **<0.001** | **<0.001** | 0.902 | 0.350 | 0.997 | **<0.001** | **<0.001** | **<0.001** | **<0.001** | **0.038** | **0.039** | 0.849 |
|  | **SPR** | | | | | | | | | | | | | |
|  | 1A | 1B | 1C | 2A | 2B | 2C | 3 | 1A | 1B | 1C | 2A | 2B | 2C | 3 |
| **Group 1B** | 0.293 |  |  |  |  |  |  | 0.188 |  |  |  |  |  |  |
| **Group 1C** | >0.999 | >0.999 |  |  |  |  |  | >0.999 | >0.999 |  |  |  |  |  |
| **Group 2A** | >0.999 | 0.349 | >0.999 |  |  |  |  | >0.999 | 0.247 | >0.999 |  |  |  |  |
| **Group 2B** | >0.999 | **0.001** | 0.055 | >0.999 |  |  |  | >0.999 | **<0.001** | **0.045** | 0.962 |  |  |  |
| **Group 2C** | >0.999 | **0.006** | 0.224 | >0.999 | >0.999 |  |  | >0.999 | **<0.001** | **0.015** | 0.428 | >0.999 |  |  |
| **Group 3** | >0.999 | **0.012** | 0.403 | >0.999 | >0.999 | >0.999 |  | 0.771 | **<0.001** | **0.023** | 0.656 | >0.999 | >0.999 |  |
| **Group 4** | >0.999 | **0.001** | **0.051** | >0.999 | >0.999 | >0.999 | >0.999 | 0.168 | **<0.001** | **0.003** | 0.141 | >0.999 | >0.999 | >0.999 |

**S. Chart 2.** Hemagglutination inhibition (GMT and SPR) pairwise comparisons for per-protocol analysis.

|  | ***p* values - Bonferroni Correction** | | | | | | | | | | | | | | |
| --- | --- | --- | --- | --- | --- | --- | --- | --- | --- | --- | --- | --- | --- | --- | --- |
|  | **Intention To Treat** | | | | | | | | | | | | | | |
| Intervention groups | **MN - ITT** | | | | | | | | | | | | | | |
|  | **GMT** | | | | | | | | | | | | | | |
|  | **d28** | | | | | | | | **d56** | | | | | | |
|  | **1A** | **1B** | **1C** | **2A** | **2B** | **2C** | **3** | **1A** | | **1B** | **1C** | **2A** | **2B** | **2C** | **3** |
| **Group 1B** | >0.999 |  |  |  |  |  |  | >0.999 | |  |  |  |  |  |  |
| **Group 1C** | 0.987 | >0.999 |  |  |  |  |  | >0.999 | | >0.999 |  |  |  |  |  |
| **Group 2A** | 0.711 | >0.999 | >0.999 |  |  |  |  | >0.999 | | >0.999 | >0.999 |  |  |  |  |
| **Group 2B** | 0.136 | 0.934 | 0.995 | >0.999 |  |  |  | **0.002** | | **0.001** | **0.010** | **0.023** |  |  |  |
| **Group 2C** | **0.001** | 0.067 | 0.152 | 0.623 | 0.992 |  |  | **0.002** | | **0.001** | **0.011** | **0.024** | >0.999 |  |  |
| **Group 3** | **0.014** | 0.397 | 0.665 | 0.989 | >0.999 | >0.999 |  | **<0.001** | | **<0.001** | **<0.001** | **<0.001** | 0.412 | 0.470 |  |
| **Group 4** | **<0.001** | **<0.001** | **<0.001** | **<0.001** | **0.001** | 0.153 | **0.016** | **<0.001** | | **<0.001** | **<0.001** | **<0.001** | **<0.001** | **<0.001** | **<0.001** |
|  | **GMFR** | | | | | | | | | | | | | | |
|  | **1A** | **1B** | **1C** | **2A** | **2B** | **2C** | **3** | **1A** | | **1B** | **1C** | **2A** | **2B** | **2C** | **3** |
| **Group 1B** | >0.999 |  |  |  |  |  |  | >0.999 | |  |  |  |  |  |  |
| **Group 1C** | 0.972 | 0.902 |  |  |  |  |  | >0.999 | | 0.991 |  |  |  |  |  |
| **Group 2A** | 0.981 | 0.925 | >0.999 |  |  |  |  | >0.999 | | >0.999 | >0.999 |  |  |  |  |
| **Group 2B** | 0.766 | 0.596 | >0.999 | >0.999 |  |  |  | **0.031** | | **0.003** | 0.224 | 0.096 |  |  |  |
| **Group 2C** | **0.002** | **0.001** | 0.267 | 0.288 | 0.599 |  |  | **0.003** | | **<0.001** | **0.034** | **0.013** | >0.999 |  |  |
| **Group 3** | **0.012** | **0.006** | 0.704 | 0.724 | 0.954 | >0.999 |  | **<0.001** | | **<0.001** | **<0.001** | **<0.001** | 0.166 | 0.701 |  |
| **Group 4** | **<0.001** | **<0.001** | **<0.001** | **<0.001** | **0.001** | 0.612 | 0.200 | **<0.001** | | **<0.001** | **<0.001** | **<0.001** | **<0.001** | **<0.001** | **<0.001** |
|  | **SPR** | | | | | | | | | | | | | | |
|  | 1A | 1B | 1C | 2A | 2B | 2C | 3 | 1A | | 1B | 1C | 2A | 2B | 2C | 3 |
| **Group 1B** | >0.999 |  |  |  |  |  |  | >0.999 | |  |  |  |  |  |  |
| **Group 1C** | >0.999 | >0.999 |  |  |  |  |  | >0.999 | | >0.999 |  |  |  |  |  |
| **Group 2A** | 0.419 | >0.999 | >0.999 |  |  |  |  | >0.999 | | >0.999 | >0.999 |  |  |  |  |
| **Group 2B** | **0.031** | 0.543 | >0.999 | >0.999 |  |  |  | **0.001** | | **0.017** | 0.294 | 0.066 |  |  |  |
| **Group 2C** | 0.119 | >0.999 | >0.999 | >0.999 | >0.999 |  |  | **<0.001** | | **0.005** | 0.104 | **0.021** | >0.999 |  |  |
| **Group 3** | **0.010** | 0.225 | 0.477 | >0.999 | >0.999 | >0.999 |  | **<0.001** | | **<0.001** | **<0.001** | **<0.001** | >0.999 | >0.999 |  |
| **Group 4** | **0.003** | 0.091 | 0.200 | >0.999 | >0.999 | >0.999 | >0.999 | **<0.001** | | **<0.001** | **<0.001** | **<0.001** | **<0.001** | **<0.001** | 0.059 |
|  | **SCR** | | | | | | | | | | | | | | |
|  | 1A | 1B | 1C | 2A | 2B | 2C | 3 | 1A | | 1B | 1C | 2A | 2B | 2C | 3 |
| **Group 1B** | >0.999 |  |  |  |  |  |  | >0.999 | |  |  |  |  |  |  |
| **Group 1C** | >0.999 | >0.999 |  |  |  |  |  | >0.999 | | >0.999 |  |  |  |  |  |
| **Group 2A** | >0.999 | >0.999 | >0.999 |  |  |  |  | >0.999 | | >0.999 | >0.999 |  |  |  |  |
| **Group 2B** | **0.030** | 0.813 | 0.537 | >0.999 |  |  |  | **<0.001** | | **0.001** | 0.302 | **0.016** |  |  |  |
| **Group 2C** | **0.035** | 0.887 | 0.594 | >0.999 | >0.999 |  |  | **<0.001** | | **<0.001** | **0.045** | **0.002** | >0.999 |  |  |
| **Group 3** | **0.008** | 0.304 | 0.185 | >0.999 | >0.999 | >0.999 |  | **<0.001** | | **<0.001** | **0.001** | **<0.001** | >0.999 | >0.999 |  |
| **Group 4** | **0.010** | 0.337 | 0.209 | >0.999 | >0.999 | >0.999 | >0.999 | **<0.001** | | **<0.001** | **<0.001** | **<0.001** | **<0.001** | **0.004** | 0.118 |

**S. Chart 3.** Microneutralization (MN) results (GMT, GMFR, SPR and SCR) pairwise comparisons for intention-to-treat analysis.

|  | ***p* values - Bonferroni Correction** | | | | | | | | | | | | | |
| --- | --- | --- | --- | --- | --- | --- | --- | --- | --- | --- | --- | --- | --- | --- |
|  | **Per Protocol** | | | | | | | | | | | | | |
| Intervention groups | **MN - PP** | | | | | | | | | | | | | |
|  | **GMT** | | | | | | | | | | | | | |
|  | **d28** | | | | | | | **d56** | | | | | | |
|  | **1A** | **1B** | **1C** | **2A** | **2B** | **2C** | **3** | **1A** | **1B** | **1C** | **2A** | **2B** | **2C** | **3** |
| **Group 1B** | 0.996 |  |  |  |  |  |  | >0.999 |  |  |  |  |  |  |
| **Group 1C** | 0.686 | >0.999 |  |  |  |  |  | >0.999 | >0.999 |  |  |  |  |  |
| **Group 2A** | 0.100 | 0.987 | >0.999 |  |  |  |  | >0.999 | 0.851 | >0.999 |  |  |  |  |
| **Group 2B** | **0.041** | 0.914 | 0.999 | >0.999 |  |  |  | **0.001** | **<0.001** | **0.004** | 0.085 |  |  |  |
| **Group 2C** | **0.001** | 0.133 | 0.405 | 0.993 | >0.999 |  |  | **0.005** | **0.001** | **0.017** | 0.229 | >0.999 |  |  |
| **Group 3** | **0.007** | 0.602 | 0.950 | >0.999 | >0.999 | >0.999 |  | **<0.001** | **<0.001** | **<0.001** | **0.001** | 0.999 | 0.966 |  |
| **Group 4** | **<0.001** | **<0.001** | **<0.001** | **0.006** | **0.014** | 0.424 | **0.035** | **<0.001** | **<0.001** | **<0.001** | **<0.001** | **<0.001** | **<0.001** | **<0.001** |
|  | **GMFR** | | | | | | | | | | | | | |
|  | **1A** | **1B** | **1C** | **2A** | **2B** | **2C** | **3** | **1A** | **1B** | **1C** | **2A** | **2B** | **2C** | **3** |
| **Group 1B** | >0.999 |  |  |  |  |  |  | >0.999 |  |  |  |  |  |  |
| **Group 1C** | 0.505 | 0.908 |  |  |  |  |  | >0.999 | 0.951 |  |  |  |  |  |
| **Group 2A** | 0.700 | 0.973 | >0.999 |  |  |  |  | >0.999 | 0.958 | >0.999 |  |  |  |  |
| **Group 2B** | 0.286 | 0.719 | >0.999 | >0.999 |  |  |  | **0.018** | **<0.001** | 0.070 | 0.095 |  |  |  |
| **Group 2C** | **0.001** | **0.012** | 0.767 | 0.679 | 0.960 |  |  | **0.039** | **0.001** | 0.138 | 0.177 | >0.999 |  |  |
| **Group 3** | **0.003** | **0.025** | 0.932 | 0.877 | 0.997 | >0.999 |  | **<0.001** | **<0.001** | **<0.001** | **<0.001** | 0.978 | 0.920 |  |
| **Group 4** | **<0.001** | **<0.001** | **0.002** | **0.001** | **0.008** | 0.715 | 0.342 | **<0.001** | **<0.001** | **<0.001** | **<0.001** | **<0.001** | **<0.001** | **0.001** |
|  | **SPR** | | | | | | | | | | | | | |
|  | 1A | 1B | 1C | 2A | 2B | 2C | 3 | 1A | 1B | 1C | 2A | 2B | 2C | 3 |
| **Group 1B** | >0.999 |  |  |  |  |  |  | >0.999 |  |  |  |  |  |  |
| **Group 1C** | >0.999 | >0.999 |  |  |  |  |  | >0.999 | >0.999 |  |  |  |  |  |
| **Group 2A** | >0.999 | >0.999 | >0.999 |  |  |  |  | >0.999 | >0.999 | >0.999 |  |  |  |  |
| **Group 2B** | **0.021** | >0.999 | >0.999 | >0.999 |  |  |  | **0.012** | **0.007** | 0.167 | 0.251 |  |  |  |
| **Group 2C** | **0.005** | 0.520 | 0.487 | >0.999 | >0.999 |  |  | **0.020** | **0.012** | 0.252 | 0.365 | >0.999 |  |  |
| **Group 3** | **0.011** | >0.999 | >0.999 | >0.999 | >0.999 | >0.999 |  | **<0.001** | **<0.001** | **<0.001** | **0.001** | >0.999 | >0.999 |  |
| **Group 4** | **0.004** | 0.479 | 0.444 | >0.999 | >0.999 | >0.999 | >0.999 | **<0.001** | **<0.001** | **<0.001** | **<0.001** | **0.001** | **0.001** | 0.179 |
|  | **SCR** | | | | | | | | | | | | | |
|  | 1A | 1B | 1C | 2A | 2B | 2C | 3 | 1A | 1B | 1C | 2A | 2B | 2C | 3 |
| **Group 1B** | >0.999 |  |  |  |  |  |  | >0.999 |  |  |  |  |  |  |
| **Group 1C** | >0.999 | >0.999 |  |  |  |  |  | >0.999 | >0.999 |  |  |  |  |  |
| **Group 2A** | >0.999 | >0.999 | >0.999 |  |  |  |  | >0.999 | >0.999 | >0.999 |  |  |  |  |
| **Group 2B** | **0.025** | 0.922 | 0.693 | >0.999 |  |  |  | **<0.001** | **<0.001** | 0.058 | **0.045** |  |  |  |
| **Group 2C** | **0.027** | 0.955 | 0.723 | >0.999 | >0.999 |  |  | **0.002** | **0.001** | 0.203 | 0.156 | >0.999 |  |  |
| **Group 3** | **0.017** | 0.788 | 0.570 | >0.999 | >0.999 | >0.999 |  | **<0.001** | **<0.001** | **0.001** | **0.001** | >0.999 | >0.999 |  |
| **Group 4** | **0.023** | 0.892 | 0.665 | >0.999 | >0.999 | >0.999 | >0.999 | **<0.001** | **<0.001** | **<0.001** | **<0.001** | **0.028** | **0.008** | 0.351 |

**S. Chart 4.** Microneutralization (MN) results (GMT, GMFR, SPR and SCR) pairwise comparisons for per-protocol analysis.

**References**

1. CBER/USFDA/USDHHS Guidance for Industry: Toxicity Grading Scale for Healthy Adult and Adolescent Volunteers Enrolled in Preventive Vaccine Clinical Trials [Internet]. Silver Spring: US Food and Drug Administration; 2007 [cited 2011 Set 29].Available at: <http://www.fda.gov/downloads/BiologicsBloodVaccines/GuidanceComplianceRegulatoryInformation/Guidances/Vaccines/ucm091977.pdf>

2. ICH Clinical Safety Data management: Definitions and Standards for Expedited Reporting [Internet]. Genebra: International Conference on Harmonisation of Technical Requirements ror Registration of Pharmaceuticals for Human Use; 1994 [cited 2012 Oct 15]. E2A. Available at: <http://www.ich.org/fileadmin/Public_Web_Site/ICH_Products/Guidelines/Efficacy/E2A/Step4/E2A_Guideline.pdf>

3. NIC/NIH NCI Common Terminology Criteria for Adverse Events (CTCAE) [Internet]. Available at: <http://evs.nci.nih.gov/ftp1/CTCAE/About.html>.

4. UMC/WHO The use of the WHO-UMC system for standardised case causality assessment [Internet]. Uppsala:The Uppsala Monitoring Centre. Available at: <http://www.who-umc.org/Graphics/24734.pdf>.
