# Supplementary material for: Dose-sparing effect of two adjuvant formulations with a pandemic influenza A/H7N9 vaccine: A randomized, double-blind, placebo-controlled, phase 1 clinical trial
Source: PLoS One. 2022 Oct 18;17(10):e0274943. doi: 10.1371/journal.pone.0274943 (PMC9578608; doi:10.1371/journal.pone.0274943)
Supplement: S1 File — (DOCX) [file pone.0274943.s002.docx]

**RESEARCH PROTOCOL OF VACCINE CLINICAL TRIAL**

**A Phase I RANDOMIZED, DOUBLE-BLIND, PLACEBO CONTROLLED, DOSE FINDING CLINICAL TRIAL TO EVALUATE THE SAFETY AND IMMUNOGENICITY OF H7N9 INFLUEnZa ANTIGEN ADJUVANTED WITH 2 DIFFERENT ADJUVANT FORMULATIONS in healthy adult volunteers IN BRAZIL**

**Protocol Number:** FLP-01-IB

**Version Number:** 4.0

**Date:** 21 June 2018

**Summary name: H7N9 INACTIVATED, ADJUVANTED influenza vaccine pHASE I clinical trial in BRAZIL**

| **Sponsor:** | Insituto Butantan, Brazil |
| --- | --- |
| **Manufacturer:** | Instituto Butantan, Brazil  H7N9 antigen and IB160 adjuvant  Infectious Disease Research Institute (IDRI), USA  SE adjuvant |
| **Planned time:** | Approximately 12 months |

Statement of compliance

I am Esper Georges Kallas, principal investigator of the study “A Phase I Randomized, Placebo-controlled, Dose Finding Clinical Trial to Evaluate the Safety and Immunogencity of H7N9 Influenza Antigen Adjuvanted with 2 different Adjuvant Formulations in Healthy Adult Volunteers in Brazil”. By signing below to ensure that the study will be carried out on schedule, content of approved protocol, and in accordance with Good Clinical Practice (GCP) as required by applicable rules of Brazil or in accordance with ICH E6, Good Clinical Practice: Consolidated Guideline.

The study informed consent documents will embody the elements of consent as described in the Declaration of Helsinki, 2013.

All key personnel (those responsible for the design and conduct of this study) will have completed Human Participants Protection Training prior to interaction with any participants or to have access to their confidential study data.

I declare that I have no potential or real conflict of interests.

***São Paulo, Brazil***

Principal Investigator

Table of Contents

[List of Abbreviations vii](#_Toc504159589)

[Protocol Summary ix](#_Toc504159590)

[Key Roles for Individuals and Institutions Involved 1](#_Toc504159591)

[1 Background Information and Scientific Rationale 5](#_Toc504159592)

[1.1 Background Information 5](#_Toc504159593)

[1.2 Overview of H7N9 Influenza disease 6](#_Toc504159594)

[1.3 Treatment and Prevention (vaccine, biological) 6](#_Toc504159595)

[1.4 Completed and planned preclinical trials 6](#_Toc504159596)

[1.5 Dose Rationale 7](#_Toc504159597)

[1.6 Potential Risks and Benefits of investigational products 7](#_Toc504159598)

[1.6.1 Potential Risks 7](#_Toc504159599)

[1.6.2 Potential Benefits 9](#_Toc504159600)

[2 Study Hypothesis and Objectives 10](#_Toc504159601)

[2.1 Study Hypothesis 10](#_Toc504159602)

[2.2 Study Objectives 10](#_Toc504159603)

[2.2.1 Primary Objectives: Safety, immunogencity and dose finding 10](#_Toc504159604)

[3 Study Outcome Measures (Endpoints) 10](#_Toc504159605)

[3.1 Safety Endpoints 10](#_Toc504159606)

[3.2 Immunogenicity Endpoints 11](#_Toc504159607)

[4 Study Design 11](#_Toc504159608)

[5 Study Enrollment and Withdrawal 15](#_Toc504159609)

[5.1 Description of Participants, Source of Participants 15](#_Toc504159610)

[5.2 Participant Inclusion Criteria 15](#_Toc504159611)

[5.3 Participant Exclusion Criteria 15](#_Toc504159612)

[5.4 Treatment Assignment Procedures 17](#_Toc504159613)

[5.4.1 Randomization Procedures 17](#_Toc504159614)

[5.4.2 Blinding and Unblinding Procedures 17](#_Toc504159615)

[5.4.3 Reasons for Withdrawal 18](#_Toc504159616)

[5.4.4 Handling of Withdrawals 18](#_Toc504159617)

[5.4.5 Strategies to Maintain and Recruit Additional Participants 18](#_Toc504159618)

[6 Study Products 19](#_Toc504159619)

[6.1 Study Product Descriptions 19](#_Toc504159620)

[6.1.1 Acquisition 19](#_Toc504159621)

[6.1.2 Formulation, Packaging and Labeling of Vaccine Components 19](#_Toc504159622)

[6.1.3 Shipment, Storage and Stability 20](#_Toc504159623)

[6.2 Dosage, Preparation and Administration of Study Products 21](#_Toc504159624)

[6.2.1 Dosage and Schedule 21](#_Toc504159625)

[6.2.2 Precautions and Warnings 21](#_Toc504159626)

[6.2.3 Administration of study products 21](#_Toc504159627)

[6.3 Accountability Procedures for Study Products 22](#_Toc504159628)

[6.4 Assessment of Compliance with Use of the Study Products 22](#_Toc504159629)

[6.5 Concomitant Medications/Treatment 22](#_Toc504159630)

[6.6 Unauthorized Products 22](#_Toc504159631)

[7 Study Schedule; Description of Visits 23](#_Toc504159632)

[7.1 Screening/Triage (visit T) 23](#_Toc504159633)

[7.2 Study Injections and Follow-up Periods 23](#_Toc504159634)

[7.2.1 Day of Study Injection (V1) 23](#_Toc504159635)

[7.2.2 First Week after Study Injection (contact C1) 25](#_Toc504159636)

[7.2.3 Seventh Day after Study Injection (visit S1) 25](#_Toc504159637)

[7.2.4 Second and Third weeks after Study Injection 26](#_Toc504159638)

[7.2.5 Immunogenicity Visit and Second Study Injection (visit V2) 26](#_Toc504159639)

[7.2.6 First Week after second Study Injection (contact C2) 27](#_Toc504159640)

[7.2.7 Seventh Day after second Study Injection (visit S2) 27](#_Toc504159641)

[7.2.8 Second and Third weeks after second Study Injection 28](#_Toc504159642)

[7.2.9 Final Study Visit (visit I) 28](#_Toc504159643)

[7.3 Final Study Call (contact C3) 28](#_Toc504159644)

[The contact C3 is schedule for day V2+194 and it is the last contact with the participant. 28](#_Toc504159645)

[7.4 Early Termination Visit 29](#_Toc504159646)

[7.5 Unscheduled Visits 29](#_Toc504159647)

[7.6 Termination of the Trial 29](#_Toc504159648)

[7.6.1 End of Trial According to the Protocol 29](#_Toc504159649)

[7.6.2 Suspension and/or Premature Termination of the Trial 30](#_Toc504159650)

[8 Study Evaluations 30](#_Toc504159651)

[8.1 Clinical Evaluations 30](#_Toc504159652)

[8.1.1 Medical History 30](#_Toc504159653)

[8.1.2 Physical Examination 30](#_Toc504159654)

[8.2 Laboratory Evaluations 31](#_Toc504159655)

[8.2.1 Clinical Laboratory Evaluations 31](#_Toc504159656)

[8.2.2 Immunogenicity Assays 32](#_Toc504159657)

[8.2.3 Preparation, Processing and Specimens 33](#_Toc504159658)

[9 Assessment of Safety and Adverse Events 33](#_Toc504159659)

[9.1 Definition and Categorization of AEs 33](#_Toc504159660)

[9.2 Specification of Safety Parameters 34](#_Toc504159661)

[9.3 Methods and Timing for Assessing and Recording Safety Parameters 34](#_Toc504159662)

[9.3.1 Adverse Events 34](#_Toc504159663)

[9.3.2 Severity of Event 35](#_Toc504159664)

[9.3.3 Causal relationship classification 38](#_Toc504159665)

[9.3.4 Solicited Reactogenicity (Expected Reactions) 40](#_Toc504159666)

[9.3.5 Solicited Local and Systemic Reactions 40](#_Toc504159667)

[9.3.6 Unsolicited Adverse Events 40](#_Toc504159668)

[9.3.7 Serious Adverse Events 41](#_Toc504159669)

[9.3.8 Procedures for Out-of-range Laboratory Test Values 41](#_Toc504159670)

[9.4 Reporting Procedures 42](#_Toc504159671)

[9.4.1 Serious Adverse Events (SAEs) 42](#_Toc504159672)

[9.4.2 Adverse Events of Special Interest (AESIs) 42](#_Toc504159673)

[9.4.3 Reporting of AEs 43](#_Toc504159674)

[9.4.4 Other Unexpected Issues/Unanticipated Problems 43](#_Toc504159675)

[9.4.5 Reporting of Pregnancy 43](#_Toc504159676)

[9.5 Duration of Follow up for AE Resolution 43](#_Toc504159677)

[9.6 Halting Rules 44](#_Toc504159678)

[9.7 Safety Oversight 45](#_Toc504159679)

[9.7.1 Study Safety Physician 45](#_Toc504159680)

[9.7.2 Data Safety and Monitoring Board (DSMB) 45](#_Toc504159681)

[10 Clinical Monitoring 46](#_Toc504159682)

[10.1 Monitoring Plan 46](#_Toc504159683)

[11 Statistical Considerations 46](#_Toc504159684)

[11.1 Sample size 46](#_Toc504159685)

[11.2 Data Analysis 47](#_Toc504159686)

[11.3 Definition of Analysis Sets 47](#_Toc504159687)

[11.4 Analysis of Immunogenicity Endpoints 48](#_Toc504159688)

[11.5 Analysis of Safety Endpoints: 48](#_Toc504159689)

[11.6 Interim analysis 49](#_Toc504159690)

[12 Data Handling and Record Keeping 50](#_Toc504159691)

[12.1 Data Capture Methods 50](#_Toc504159692)

[12.2 Database Management and Analysis Software 51](#_Toc504159693)

[12.3 Source Documents and Source Document Access 51](#_Toc504159694)

[13 Quality Control and Quality Assurance 52](#_Toc504159695)

[14 Ethics/Protection of Human Participants 52](#_Toc504159696)

[14.1 Ethical Standard 52](#_Toc504159697)

[14.2 Financing and Insurance 52](#_Toc504159698)

[14.3 Assurance of Emergency Medical Care and Care for other Adverse Events 52](#_Toc504159699)

[14.4 Independent Ethics Committee and Regulatory Approval 53](#_Toc504159700)

[14.5 Informed Consent Process 53](#_Toc504159701)

[14.6 Participant Confidentiality 54](#_Toc504159702)

[14.6.1 Confidentiality of Data 54](#_Toc504159703)

[14.6.2 Confidentiality of Participant Records 55](#_Toc504159704)

[14.7 Sharing of Study Results 55](#_Toc504159705)

[14.7.1 Sharing of Study Results with the Participant 55](#_Toc504159706)

[14.7.2 Incidental Health Findings 55](#_Toc504159707)

[14.8 Biological samples storage and use of biorepository 55](#_Toc504159708)

[14.9 Potential Risks and How They are Addressed 56](#_Toc504159709)

[14.10 Benefits to Study Participants 57](#_Toc504159710)

[15 Clinical Study Report and Publication Policy 57](#_Toc504159711)

[15.1 Clinical Study Report 57](#_Toc504159712)

[15.2 Publication Policy 58](#_Toc504159713)

[16 Annex A: List of Adverse Events of Special Interest 59](#_Toc504159714)

[17 References 61](#_Toc504159715)

## List of Abbreviations

| AE | Adverse Event |
| --- | --- |
| AESI | Adverse Events of Special Interest |
| ANVISA | Brazil's National Health Surveillance Agency |
| ALT=SGPT | Alanine Aminotransferase=Serum glutamic pyruvic transaminase |
| AST=SGOT | Aspartate Aminotransferase= Serum glutamic oxaloacetic transaminase |
| BARDA | Biomedical Advanced Research and Development Authority (of US Department of Health and Human Services) |
| CDC | US Centers for Disease Control and Prevention |
| CI | Confidence Interval |
| cm | Centimeter |
| CONEP | National Committee for Ethics and Research (Brazil) |
| CRF | Case Report Form |
| CSR | Clinical Study Report |
| °C | Degrees Celsius |
| D | Day |
| DSMB | Data and Safety Monitoring Board |
| eCRF | Electronic Case Report Form |
| EDC | Electronic Data Capture |
| ERC | Ethics Review Committee (WHO) |
| FA | Full Analysis (population) |
| FDA | U S Food and Drug Administration |
| GAP | The Global Action Plan for Influenza Vaccines |
| GCP | Good Clinical Practice |
| GLP | Good Laboratory Practices |
| GMFR | Geometric Mean Fold Rises |
| GMT | Geometric Mean Titer |
| H5N1 | Influenza A virus subtype hemagglutinin 5, neuraminidase 1 |
| H7N9 | Influenza A virus subtype hemagglutinin 7, neuraminidase 9 |
| HA | Hemagglutinin |
| HI | Hemagglutination Inhibition |
| HBsAg | Hepatitis B Surface Antigen |
| HBV | Hepatitis B Virus |
| hCG | Human Chorionic Gonadotropin |
| Hct | Hematocrit |
| HCV | Hepatitis C Virus |
| HIV | human immunodeficiency virus |
| Hgb  IB | Hemoglobin  Instituto Butantan |
| IB160 | Oil-in-water emulsion adjuvant formulation produced by Butantan |
| ICF | Informed Consent Form |
| ICH | International Conference on Harmonization |
| ICMJE | International Committee of Medical Journal Editors |
| IDRI  IEC  IITRI | Infectious Disease Research Institute  Independent Ethics Committee  IIT Research Institute, Chicago, USA |
| IM | Intramuscular |
| IP | Investigational Product |
| IRB | Institutional Review Board |
| LEC | Local Ethics Committee |
| LLN | Lower Limit of Normal |
| MedDRA | Medical Dictionary for Regulatory Activities |
| mg | Milligram |
| mL | Milliliter |
| mm | Millimeter |
| MNT | Microneutralization test/assay |
| MOH | Ministry of Health |
| N | number (typically refers to number of participants) |
| NA | Neuraminidase |
| NCS | Non Clinically Significant |
| PI | Principal Investigator |
| PBS | Phosphate buffered saline |
| PP | Per Protocol (population) |
| SAE | Serious Adverse Event |
| SE | Stable Emulsion, oil-in-water adjuvant produced by IDRI |
| SOP | Standard Operating Procedure |
| STATA | Data Analysis and Statistical Software |
| µg | Microgram |
| ULN | Upper Limit of Normal |
| US | United States |
| WBC | White Blood Cell |
| WHO | World Health Organization |
|  |  |

## Protocol Summary

| **Title:** | A Phase I Randomized, Double-blind, Placebo-controlled, Dose Finding Clinical Trial to Evaluate the Safety and Immunogencity of H7N9 Influenza Antigen Adjuvanted with 2 different Adjuvant Formulations in Healthy Adult Volunteers in Brazil. |
| --- | --- |
| **Study Population:** | Approximately 432 healthy male and female (non-pregnant) adults, 18 to 59 years of age. |
| **Candidate vaccine components overview:** | Butantan has produced a clinical grade stock of an H7N9 vaccine antigen component to be tested in conjunction with an adjuvant component in clinical trials as a potential pandemic influenza vaccine;  Butantan has produced a clinical grade adjuvant formulation, IB160, intended to be formulated with the Butantan H7N9 vaccine antigen as a vaccine candidate for pandemic influenza.  IDRI has produced a clinical grade adjuvant formulation, SE, intended to be formulated with the Butantan H7N9 vaccine antigen as a vaccine candidate for pandemic influenza; |
| **Invervention Groups:** | There will be four intervention groups:  **Group 1**: H7N9 + adjuvant IB160   - 15 µg H7N9 - 7.5 µg H7N9 - 3.75 µg H7N9   **Group 2**: H7N9 + adjuvant SE  • 15 µg H7N9  • 7.5 µg H7N9  • 3.75 µg H7N9  **Group 3:** H7N9 + No adjuvant  • 15 µg H7N9  **Group 4:** Placebo (PBS) |
| **Phase** | Phase I |
| **Study Aim** | The overall aim of this study is to evaluate the safety, immunogenicity and dose sparing effects of H7N9 influenza antigen formulated with 2 different adjuvants |
| **Objectives** | **Safety, immunogencity and dose finding**   - To evaluate the safety and reactogenicity of 2 intramuscular doses given 28 days apart in healthy adults of H7N9 adjuvanted and non-adjuvanted vaccine candidates compared to placebo. - To evaluate the immunogenicity by HI of 2 intramuscular doses given 28 days apart in healthy adults of H7N9 adjuvanted and non-adjuvanted vaccine candidates as determined by: serconversion, GMT titres and seroprotection. - Based on safety and immunogenicity findings to select 1 vaccine candidate from group 1 and group 2 to take into a phase II study in the future. |
| **Number of Sites:** | Minimum of 3 clinical sites |
| **Study Duration:** | Approximately 12 months |
| **Participation Duration:** | Approximately 8 months (excluding screening) |
|  |  |

**Table 1** Study procedures.

| **Type of visit/contact** | | | | | | **Triage** | | | **Vaccine 1** | | | **Contact 1** | | **Safety 1** | | **Vaccine 2** | | | **Contact 2** | | **Safety 2** | | **Immunity** | | | **Contact 3** |  |
| --- | --- | --- | --- | --- | --- | --- | --- | --- | --- | --- | --- | --- | --- | --- | --- | --- | --- | --- | --- | --- | --- | --- | --- | --- | --- | --- | --- |
| **Visit** | | | | | | **T** | | | **V1** | | | **C1** | | **S1** | | **V2** | | | **C2** | | **S2** | | **I** | | | **C3** |  |
| Programmed visit | | | | | | V1  (-30)d | | | V1 | | | V1+3 (+2)d | | V1+7  (+3)d | | V1+28  (+7)d | | | V2+3  (+2)d | | V2+7  (+3)d | | V2+28  (+7)d | | | V2+194  (+7)d |  |
| **Procedures** | | |  | | | | | | |  |  |  |  |  |  |  |  |  |  |  |  |  |  |  |  |  |  |
| Information and written informed consent | | | | | | X | | |  | | |  | |  | |  | | |  | |  | |  | | |  |  |
| Check/confirm inclusion/exclusion criteria | | | | | | X | | | X | | |  | |  | | X | | |  | |  | |  | | |  |  |
| Collect baseline demographic data | | | | | | X | | |  | | |  | |  | |  | | |  | |  | |  | | |  |  |
| Collect/review medical history | | | | | | X | | | X | | |  | | X | | X | | |  | | X | | X | | |  |  |
| Perform targeted physical examination | | | | | | X | | | X | | |  | | X | | X | | |  | | X | | X | | |  |  |
| Evalute potential for pregnancy^†^ | | | | | | X | | | X | | |  | |  | | X | | |  | |  | | X | | |  |  |
| Randomization | | | | | |  | | | X | | |  | |  | |  | | |  | |  | |  | | |  |  |
| Observe for immediate reactions for 30 minutes | | | | | |  | | | X | | |  | |  | | X | | |  | |  | |  | | |  |  |
| Evaluate solicited adverse events | | | | | |  | | | X | | | X | | X | | X | | | X | | X | | X | | |  |  |
| Survaillance of unsolicited adverse events | | | | | |  | | | X* | | | X | | X | | X | | | X | | X | | X | | | X |  |
| Update contact information | | | | | |  | | | X | | | X | | X | | X | | | X | | X | | X | | |  |  |
| **Intervention** | | | | | |  | | |  | | |  | |  | |  | | |  | |  | |  | | |  |  |
| Vacination | | | | | |  | | | X | | |  | |  | | X | | |  | |  | |  | | |  |  |
| **Participant diary** | | | | | |  | | |  | | |  | |  | |  | | |  | |  | |  | | |  |  |
| Deliver diary and thermometer | | | | | |  | | | X | | |  | |  | | X | | |  | |  | |  | | |  |  |
| Instruct participant on use of diary and thermometer | | | | | |  | | | X | | |  | |  | | X | | |  | |  | |  | | |  |  |
| Review and collect participant diary | | | | | |  | | |  | | |  | | X | |  | | |  | | X | |  | | |  |  |
| **Safety evaluation** |  | | |  | | |  | | | |  | | | |  | | |  | | | |  | | |  | | |
| Urine β-hCG ^†^ | | | | | | X | | | X | | |  | |  | | X | | |  | |  | | X | | |  |  |
| Collect blood sample for HBV, HCV and HIV testing | | | | | | X | | |  | | |  | |  | |  | | |  | |  | |  | | |  |  |
| Collect blood sample for hematology | | | | | | X | | |  | | |  | | X | |  | | |  | | X | |  | | |  |  |
| Collect blood sample for biochemical testing | | | | | | X | | |  | | |  | | X | |  | | |  | | X | |  | | |  |  |
| **Immunogenicity evaluation** | |  | | |  | | |  | | | | |  | | | |  | | |  | | | |  | | |  |
| Collect blood sample for anti-influenza serologic testing | | | | | |  | | | X | | |  | |  | | X | | |  | | X | | X | | |  |  |
| **Volume of blood collected (ml)** | |  | | |  | | |  | | | | |  | | | |  | | |  | | | |  | | |  |
| Total volume | | | | | | **17,5** | | | **30,5** | | | **-** | | **9** | | **39,5** | | | **-** | | **39,5** | | **30,5** | | | **-** |  |

^†^ For womem at risk of pregnancy

Research Protocol

# Key Roles for Individuals and Institutions Involved

| **Primary Principal Investigator and Primary Institute:** | Dr Esper Georges Kallas, Departamento de Doenças Infecciosas, Faculdade de Medicina, Universidade de São Paulo, Brasil |
| --- | --- |
| **Co-Principal Investigators and Institues** | Dr Lúcia Maria Mattei de Arruda Campos, Instituto da Criança, Hospital de Clínicas da Faculdade de Medicina da Universidade de São Paulo, HCFMUSP, Brasil.  Dr Eduardo Barbosa Coelho, Departamento de Clínica Médica, Faculdade de Medicina de Ribeirão Preto, Universidade de São Paulo, Brasil |
| **Institutional Review Boards (IRBs):** | Local Ethics Committee (LEC) of School of Medecine, Infectious Diseases Department, University of Sao Paulo, Brazil  WHO Ethics Review Committee (ERC)  National Committee for Ethics and Research (CONEP) |
|  |  |
| **Manufacturer:** | Instituto Butantan (vaccine and IB160 adjuvant)  Av. Vital Brasil, 1500 – Butantã  São Paulo - SP, 05503-900  Brazil  Contact Point: Alexander Precioso  Phone (mobile): +55 11 9964 47132  Phone (office): +55 11 3723 2121  email: [alexander.precioso@butantan.gov.br](mailto:alexander.precioso@butantan.gov.br)  Infectious Disease Research Institute (SE adjuvant)  1616 Eastlake Avenue East, Suite 400  Seattle, Washington 98102  USA  Contact Point: Anna Marie Beckmann  Phone (mobile): +1 206 437 0499  Phone (office): +1 206 858 6099  email: [annamarie.beckmann@idri.org](mailto:annamarie.beckmann@idri.org) |
|  |  |
| **Sponsor:** | Instituto Butantan  Av. Vital Brasil, 1500 – Butantã  São Paulo - SP, 05503-900  Brazil  Contact Point: Alexander Precioso  Phone (mobile): +55 11 9964 47132  Phone (office): +55 11 3723 2121  email: [alexander.precioso@butantan.gov.br](mailto:alexander.precioso@butantan.gov.br) |
|  |  |
| **Study Monitoring Organization:** |  |
|  |  |
| **Clinica**l **Sites :** | Research center 01:  Centro de Pesquisas Clínicas do Instituto Central do Hospital das Clínicas da Faculdade de Medicina da Universidade de São Paulo  Av. Dr. Enéas de Carvalho Aguiar, 155,  4º andar, Bloco 15 - Prédio dos Ambulatórios São Paulo - SP  Brasil 05403 000  Telephone: 11 2661 7845 / 2661 7846  Fax: 11 2661 7847  Research center 02:  Centro de Pesquisa Clínica do Instituto da Criança do Hospital das Clínicas da Faculdade de Medicina da Universidade de São Paulo - ICr/HCFMUSP  Av. Enéas de Carvalho Aguiar, nº 647  Cerqueira César  05415009 - Sao Paulo, SP - Brasil  Telephone: (11) 26618675  Fax: (11) 26618503  Research center 03:  Hospital das Clínicas da Faculdade de Medicina de Ribeirão Preto da Universidade de São Paulo  Bloco G Subsolo II - Campus Universitário s/n - Monte Alegre  Ribeirão Preto SP  Telephone: (16) 3602-2632 |
| **Clinical and Specialty Laboratories:** | Instituto Adolfo Lutz  Av. Dr. Arnaldo, 355  São Paulo – SP  Brasil 01246 902  LIM-60 – Laboratório de Imunologia Clínica e Alergia  Disciplina de Imunologia Clínica e Alergia  Faculdade de Medicina da Universidade de São Paulo  Av. Dr. Arnaldo, 455, sala 3205, São Paulo – SP  Brasil 01246 903  Telephone: 11 3061 8395 |
|  | Laboratório De Vírus Respiratórios e do Sarampo  Instituto Oswaldo Cruz  Fundação Oswaldo Cruz  Av. Brasil, 4365-Pavilhão Helio e Peggy Pereira/HPP sala B106  Manguinhos  21045900 - Rio de Janeiro, RJ - Brasil  Telephone: (21) 25621778  Fax: (21) 25739591 |
|  | Laboratório de Desenvolvimento Analítico  Instituto Butantan  Avenida Vital Brasil, 1500  05503-900 – São Paulo, SP – Brasil  Telephone: 11 26279453 |

# Background Information and Scientific Rationale

## 1.1 Background Information

Instituto Butantan is a public Brazilian biomedical research-manufacturer center affiliated with the São Paulo State Secretary of Health and is one of the main public producers of vaccines, antivenoms, and antitoxins in Latin America.^[[1]](#endnote-2)^

Butantan participates in the “WHO Global Action Plan for influenza vaccines” (GAP), an initiative to support development of new influenza vaccines, increase demand for seasonal vaccines, and enhance influenza vaccine production capacity. With support from international donors, including the United States (US) Department of Health and Human Services, the WHO leads a program to support influenza vaccine manufacturers in developing countries that is crucial to increasing overall manufacturing capacity as well as to enhancing regional access to vaccines.

Instituto Butantan has been recognized nationally and internationally by its influenza seasonal vaccine production^[[2]](#endnote-3)^ and has been funded by the WHO to manufacture vaccines against potential pandemic influenza viruses such as H5N1 and, more recently H7N9.

Butantan has been funded by the WHO to conduct clinical trials of its H7N9 influenza vaccine with it’s proprietary adjuvant, IB160, as one of the adjuvant components of an H7N9 influenza vaccine against potential pandemic influenza. This is the first time that the IB160 adjuvant will be evaluated in a clinical trial, however IB160 is the same formulation as MF59™, adjuvant approved for use in humans since 1997 as part of FLUAD™, a seasonal influenza vaccine for use in the elderly (more information is provided in Section 1.6.1)

In order to support the availability of adjuvants to increase immunogenicity and doses sparing for pandemic influenza vaccines, the Biomedical Advanced Research and Development Authority (BARDA) of the US Department of Health and Human Services has established an adjuvant hub at the Infectious Disease Research Institute (IDRI) in Seattle, USA. The goal of the hub is for IDRI to partner with manufacturers in the GAP program to develop adjuvanted influenza vaccine capabilities. Under the grant agreement between BARDA and IDRI, BARDA has provided funding to IDRI to produce IDRI’s proprietary stable oil-in-water emulsion (squalene) adjuvant (“SE Adjuvant”) for use in the clinical study to evaluate the reactogenicity and immunogenicity of the SE Adjuvant formulated with the Butantan H7N9 influenza antigen as a vaccine candidate.

IDRI’s SE Adjuvant has been evaluated in three clinical trials using different antigens including anH5N1 recombinant influenza (Panblok rH5) vaccine. In this Phase 2 clinical trial, healthy adult subjects were randomized (1:1:1:1) to receive two doses of rH5 (A/Indonesia/05/2005) at 7.5 µg with no adjuvant, or 3.8, 7.5 or 15 µg rH5 formulated with 2% SE. 341 subjects were enrolled in the study of which 321 received 2 doses of vaccine at days 0 and 21. Vaccination was well-tolerated in all groups.. All three adjuvanted dose groups met the current criterion for seroconversion rate for pandemic vaccines. This dose-ranging study also identified a group (15 µg per dose formulated with 2% SE) that met the criteria for both seroconversion and percentage of participants achieving an HI antibody titer ⩾40. Vaccination was well-tolerated in all groups..^[[3]](#endnote-4)^

The Division of Clinical Trials and Pharmacovigilance at Instituto Butantan has designed and conducted several clinical trials from phase I to phase IV, including influenza vaccine clinical trials.^[[4]](#endnote-5)^ *^[[5]](#endnote-6)^ ^[[6]](#endnote-7)^ ^[[7]](#endnote-8)^ ^[[8]](#endnote-9)^ ^[[9]](#endnote-10)^ ^[[10]](#endnote-11)^ ^[[11]](#endnote-12)^ ^[[12]](#endnote-13)^*

### 1.2 Overview of H7N9 Influenza disease

The first human infection with an avian influenza A (H7N9) virus was reported in China in March 2013, and since that time hundreds of cases have been documented.^[[13]](#endnote-14)^ As of 14 February 2017 a total of 1223 laboratory-confirmed human infections, including at least 380 deaths of avian influenza A(H7N9) virus have been reported to WHO since early 2013.^[[14]](#endnote-15)^

Most infections are believed to result from poultry exposure, and no evidence of sustained person-to-person spread of H7N9 has yet been found, although limited person-to-person spread appears to have occurred.^[[15]](#endnote-16)^ While human infections with the avian influenza A(H7N9) virus are unusual there is the potential for significant public health impact given the high case fatality rate observed.

### 1.3 Treatment and Prevention (vaccine, biological)

Following the emergence of avian influenza A/H7N9 influenza virus in humans in China in March 2013, the WHO Essential Regulatory Laboratories prepared candidate vaccine viruses and reagents for further development and several manufacturers have developed various inactivated influenza vaccines with and without adjuvant against A/H7N9 and tested these candidates in several trials in healthy adults. For A/H7N9 candidates used in clinical trials, HA dosing has ranged from 3.75 µg to 45 µg and a two-dose regimen has been evaluated. A single adjuvant formulation has been tested with an H7N9 vaccine and has shown significantly improved immunogenicity at lower antigen doses (dose sparing) as compared to un-adjuvanted vaccine trials. Mild pain at the injection site and headache were the most common adverse reactions identified with various candidates. Vaccines were otherwise well tolerated (see Table 2)

### 1.4 Completed and planned preclinical trials

Experimental lots of H7N9 split inactivated influenza vaccine produced by Instituto Butantan were tested in an immunogenicity and efficacy ferret challenge study in the Netherlands in 2014 with or without an oil-in-water adjuvant formulation (SWE01, with the same formulation as IB160) produced by the University of Lausanne. The H7N9 split vaccine alone was tested at a 45 and 15 μg doses and the SWE01 adjuvanted‐split vaccine a 15 and 7.5μg dose. Thirty‐seven days after vaccination, the animals were challenged intra‐tracheally with wild‐type A/Anhui/1/2013. The study demonstrated the immunogenicity of the H7N9 antigen produced by Butantan and demonstrated a clear immune potentiating effect and a potential beneficial effect on efficacy through the use of an adjuvant: high and medium dose vaccination with split vaccine alone induced very low or no HA specific antibody titers, whereas medium and low dose vaccination with split plus SWE01 induced high HA specific antibody titers. There was efficient reduction in virus replication in animals vaccinated with the adjuvanted vaccine formulations after challenge when compared to the nonadjuvanted split vaccines. Moreover, weight loss and fever were also significantly lower with inclusion of the adjuvant.

Clinical lots of H7N9 split inactivated vaccine were successfully manufactured, tested and released by Butantan in February 2017.

The vaccine antigen and adjuvant components alone and formulated as the vaccine candidates will be evaluated in a mouse immunogenicity study to be conducted at IDRI in Seattle and in a repeated dose toxicity and local tolerance study in rabbits to be conducted by the IIT Research Institute (IITRI) in Chicago.

The vaccine candidates will only be taken into clinical trials if these studies demonstrate that the vaccine candidates are immunogenic in mice and have acceptable safety profiles in the rabbit toxicology study following single and multi-dose immunizations. More details of the preclinical studies will be included in the Investigator’s Brochure and in a revision to this protocol.

## 1.5 Dose Rationale

This phase I clinical trial will be a dose finding study to evaluate safety and dose sparing effects of two different adjuvant formulations with different doses of H7N9 antigen (3.75, 7.5 and 15 µg).

The rationale for the dose range of 3.75 to 15 µg is based on published clinical data from previously conducted trials using different adjuvant formulations and H7N9 antigen, see Table 2.

## 1.6 Potential Risks and Benefits of investigational products

### 1.6.1 Potential Risks

This is a first in human administration of these combinations: the Butantan H7N9 influenza antigen adjuvanted with either the Butantan adjuvant IB160 or the IDRI SE adjuvant. However, there has been experience in administration of other H7N9 influenza vaccines(Table 2).

**Table 2: H7N9 Inactivated Split Virus Vaccines: Phase 1 and 2 Clinical Trials***

| **Producer** | **Strain** | **Adjuvant** | **Antigen Dose (µg)** | **Clinical Study Design** | **N** | **Age (Yrs)** | **Schedule** | **Immunogenicty evaluations** |
| --- | --- | --- | --- | --- | --- | --- | --- | --- |
| GSK Biologicals^[[16]](#endnote-17)^ | A/Anhui/13 | AS03_A_ or AS03_B_ | 3.75, 7.5 | Phase 1  Randomized, observer-blind | 420 | 18-64 | Gr 1-4: adjuvanted vaccine (3.75 µg, 7.5 µg)  Gr5: nonadjuvanted vaccine (15 µg) Gr6: placebo  All groups received injections on days 0 & 21 | All adjuvanted vaccines met regulatory acceptance criteria.  Seroconversion: ≥85.7% adjuvanted; 23.2% nonadjuvanted  Seroprotection: ≥91.1% adjuvanted; 28.6% nonadjuvanted  GMT: ≥92.9% adjuvanted; 17.2% nonadjuvanted |
| Sanofi Pasteur^[[17]](#endnote-18)^ | A/Shanghai/2/2013 | MF59; | 3.75, 7,5, 15, 45 | Phase 2 Randomized, double-blind | 700 | 19-64 | Gr 1-3: adjuvanted vaccine (3.75 µg, 7.5 µg, 15 µg)  Gr 4,5: One adjuvanted dose & one nonadjuvanted dose (15 µg)  Gr 6,7: nonadjuvanted vaccine (15 µg, 45 µg)  All groups received injections on days 0 & 21 | HI titer minimal w/nonadjuvanted vaccine  Seroconversion: 59% adjuvanted (3.75 µg)  GMT: 33 (3.75 µg)  Neutralizing Ab: 82% (3.75 µg)  Higher antigen doses not associated w/increased response |
| Sanofi Pasteur^[[18]](#endnote-19)^ | A/Shanghai/2/2013 | MF59;  AS03;  None | 3.75, 7,5, 15, 45 | Phase 2 Randomized, double-blind, controlled | 980 | 19-64 | Gr 1-5: AS03 adjuvanted vaccine (3.75 µg, 7.5 µg, 15 µg)  Gr 6,7: adjuvanted vaccine AS03 one dose, MF59 one dose (15 µg)  Gr 8: adjuvanted vaccine MF59 (15 µg) Gr 9, 10: nonadjuvanted vaccine (15 µg, 45 µg)  All groups received injections on days 0 & 21 | HI titer ≥40: 2% nonadjuvanted; 84% adjuvanted AS03; 57% adjuvanted MF59  GMT: 103.4 w/AS03, 29.0 w/MF59, and 41.5, 58.6 w/alternating adjuvants |
| Sanofi Pasteur^[[19]](#endnote-20)^ | A/Shanghai/2/2013 | MF59 | 3.75, 7,5, 15 | Phase 2 Randomized, partially-blind | 479 | ≥65 | Gr 1: adjuvanted vaccine (3.75 µg)  Injections on days 1, 29, 169  Gr 2: adjuvanted vaccine (3.75 µg)  Injections on days 1, 57, 169  Gr 3: adjuvanted vaccine (7.5 µg)  Injections on days 1, 29, 169  Gr 4: adjuvanted vaccine (7.5 µg)  Injections on days 1, 57, 169  Gr 5: adjuvanted vaccine (15 µg)  Injections on days 1, 29, 169  Gr 6: adjuvanted vaccine (15 µg)  Injections on days 1, 57, 169 | No data |

* All vaccines produced on egg substrate

Oil-in-water adjuvants, such as MF59 are licensed for use in several countries, this includes seasonal vaccines for use in pediatric and geriatric populations (Fluad, Novartis) and pandemic H1N1 vaccines (pandemrix, GSK). These vaccines have been shown to be safe and well-tolerated.^[[20]](#endnote-21)^ MF59 is an oil-in-water emulsion of squalene oil. Squalene, a naturally occurring substance found in humans, animals and plants, is highly purified using the vaccine manufacturing process.

Butantan’s adjuvant IB160 and IDRI’s SE are both oil-in-water adjuvants containing squalene.

We expect this study vaccine to cause side effects similar to other inactivated influenza vaccines (adjuvanted and unadjuvanted).

From worldwide use, we know that administering influenza vaccines causes the participant immediate mild pain in the arm in most recipients. Other side effects include pain and inflammation (redness/swelling) at the injection site or systemic symptoms such as headache, fever, tiredness, and body aches. It is very rare, but serious or allergic reactions may also happen. Guillain Barre syndrome is a potential serious adverse reaction of influenza vaccines in general, although rare: approximately 1 case in 1 million vaccinees, according to the CDC. Adjuvanted vaccines pose no higher risk compared to non-adjuvanted vaccines.^[[21]](#endnote-22)^

These potential risks will be addressed by several measures:

- The study is supervised by physicians and the study staff are trained and equipped to handle any vaccine reaction. To manage immediate reactions, participants remain at the study site at least 30 minutes after injection.
- The safety of the study is overseen by a Data and Safety Monitoring Board (DSMB) that meets (as specified in section 9) to review safety information..
- The study staff check in with the participants during a phone call (C1) on Day 3 (+2) following each vaccination. During this call study stuff will evaluate the occurence of solicited adverse events or other concerns. If there is any concern then participants will be invited to the clinic for further evaluation.
- Study staff will check in volunteers at a visit (S1) on Day 7 (+3) following each vaccination to check on their health status. A safety blood sample will be drawn.
- Volunteers can call the clinic at any time to report an adverse event.
- Volunteers come back to the clinic at Day 28 (+7) post each vaccination for immunogenicity blood draw and can report on any adverse events.

### 1.6.2 Potential Benefits

There will be no direct benefit to subjects from the experimental vaccine. Subjects who participate will contribute to knowledge about the safety and immunogenicity of this investigational vaccine that may lead to better prevention of H7N9 influenza in the event of a pandemic with H7N9 influenza virus. Subjects will receive a complete physical exam and routine laboratory tests to assess their health status. Society may benefit from the potential development of a new vaccine for pandemic H7N9 influenza.

# Study Hypothesis and Objectives

## 2.1 Study Hypothesis

Two doses of H7N9 vaccine candidate given with or without two novel adjuvants 28 days apart will be safe and immunogenic in this study population.

## 2.2 Study Objectives

The purpose of the study is to evaluate the safety and immunogenicity in healthy adult participants of H7N9 adjuvanted vaccine candidates as follows:

### 2.2.1 Primary Objectives: Safety, immunogencity and dose finding

- To evaluate the safety and reactogenicity of 2 intramuscular doses given 28 days apart in healthy adults of H7N9 adjuvanted and non-adjuvanted vaccine candidates compared to placebo.
- To evaluate the immunogenicity of 2 intramuscular doses given 28 days apart in healthy adults of H7N9 adjuvanted and non-adjuvanted vaccine candidates as determined by: serconversion, GMT titres and seroprotection.
- Based on safety and immunogecity findings to select 1 adjuvanted vaccine candidate from group 1 and group 2 to evalute in a future clinical trials.

# Study Outcome Measures (Endpoints)

## 3.1 Safety Endpoints

**Primary Endpoints:** The vaccine safety profiles will be assessed according to:

- Number, severity and percentage of participants with solicited and/or unsolicited local and/or systemic adverse reactions during a 7-day period post each vaccination.

**Secondary Endpoints:** The following safety parameters will be followed:

- Number and percentage of participants with unsolicited adverse reactions for 28 days post each vaccination.
- All serious adverse events (SAEs) occurring over the study period.

**Exploratory Endpoints:** The following safety parameters will be followed:

- All adverse events of special interest (AESIs) as listed in Annex A occurring over the study period.

## 3.2 Immunogenicity Endpoints

**Primary endpoints:** The three coprimary immunogenicity endpoints, which will be measured on visits V2 (4 weeks after first study injection), S2 (1 week after the second study injection) and I (4 weeks after the second study injection), are:

1. The proportion of participants that presented seroconversion (prevaccination HI antibody titer ≤1:10 and postvaccination HI antibody titer ≥1:40, or prevaccination HI antibody titer ≥1:10 and a postvaccination increase by a factor of four or more)
2. The proportion of participants with seroprotection (postvaccination HI antibody titer ≥ 1:40)
3. The geometric mean HI titer

**Secondary endpoints:** The three cosecondary immunogenicity endpoints, which will be measured on visit V2 (4 weeks after the first study injection) and I (4 weeks after the second study injection), are:

1. The proportion of participants that presented a postvaccination increase by a factor of four or more of neutralizing antibodies against Influenza H7N9 (MNT)
2. The proportion of participants with seroprotection (postvaccination MNT antibody titer ≥ 1:40)
3. The geometric mean MNT titer

**Exploratory Endpoint**: in a subset of participants samples will be analyzed also by other assays, such as single radial haemolysis (SRH) and enzyme-linked lecitine assay (ELLA), in order to obtain further information about the immune response elicited by the vaccine candidates and to determine whether recipients of the study vaccine develop antibodies that cross react with other H7N9 viruses. HAI will also be conducted using other lineages of H7N9.

# Study Design

This is a phase I, multicenter, double-blind, placebo-controlled, multi-arm parallel study to evaluate the safety and immunogenicity of monovalent adjuvanted and non-adjuvanted vaccines against H7N9 influenza virus in healthy adult volunteers.

The study aims to assess safety of different vaccine candidates and obtain preliminary data on immunogenicity to inform optimal dosing for further clinical trials.

Participants will be randomly assigned to one of 8 interventions to receive two doses, 28 days apart, of one of the six adjuvanted candidate vaccines, or a non-adjuvanted vaccine, or placebo. The summary of the design is in Table 3.

Participants will be sequentially allocated by using a block randomization An unblinded pharmacist will prepare the study product, either vaccine candidates or placebo. Participants, other clinical staff and lab personnel will remain blinded to study product allocation.

**Table 3: Study Design**

| **Intervention Groups** | **Doses** | **Number of volunteers** |
| --- | --- | --- |
| Group 1 | 3.75 µg + Adjuvant IB160 | 54 |
|  | 7.5 µg + Adjuvant IB160 | 54 |
|  | 15 µg + Adjuvant IB160 | 54 |
| Group 2 | 3.75 µg + Adjuvant SE | 54 |
|  | 7.5 µg + Adjuvant SE | 54 |
|  | 15 µg + Adjuvant SE | 54 |
| Group 3 | 15 µg | 54 |
| Group 4 | Placebo | 54 |
|  | Total | 432 |

Before the study starts, this protocol will be submitted to Ethics Committees and the Brazilian National Regulatory Agency (ANVISA) for approval. The study will be conducted in accordance with the principles of the Declaration of Helsinki and Good Clinical Practices.

All participants must sign the written informed consent before enrollment. Eligible participants will be enrolled into the trial after signing the consent form and demonstrating verbal understanding of the purpose, procedures, and possible risks during the consent discussion. This should be recorded in the source documentation.

After obtaining consent for participation in the study, participants will be screened for eligibility through medical history review, targeted physical examination, testing for serologic evidence of chronic viral hepatitis infection [hepatitis B virus (HBV), hepatitis C virus (HCV)] and human immunodeficiency virus (HIV), selected biochemical and hematological blood tests. Pre-test and post-test counseling will be offered to participants according to protocols of the Brazilian Ministry of Health. Patients who test positive for any of the serological tests will be referred to specialized prevention, treatment and care services. It should be noted that the study laboratories participate in proficiency programs for quality evaluation. Women will have a urine pregnancy test.

On the day of screening/triage (visit T), the medical history, physical exam and screening laboratory results will be reviewed by the investigator to determine if the volunteer is healthy and otherwise eligible to participate in the study.

On the day of the first study infection (visit V1), blood samples will be collected for immunogenicity testing prior to vaccination. Women will undergo a pregnancy test using urine samples, prior to administering the study injection.

After the study injection (visit V1), participants will be carefully monitored for adverse reactions for 30 minutes.

During the first week following each study injection, participants will be asked to record local and systemic signs and symptoms using preprinted Participant Diary, a thermometer, and a a local reaction measurement device. In addition to solicited signs, participants will be asked to report any other adverse events. Concomitant medications will also be recorded. Members of the investigator’s clinical team will have contact with participants via phone (C1) on Day 3 (+2) after study injection to check that participants are correctly completing the Participant Diary and to check on the participants’ well-being (occurrence of solicited and unsolicited adverse events).

Participants will return to the study site seven days after study injection, visit S1 on V1+7 (+3) days. At that time, the Principal Investigator or designee will review the Participant’s Diary, confirm the information included and transcribe all solicited and other adverse events onto source document and the case report forms. Blood will be collected for selected biochemical and hematological blood tests. Results of the laboratory tests must be reviewed by the study physician. The study physician will determine the severity grade of laboratory results using the Toxicity Table for Grading Severity of AEs (see section 9). If the result is Grade 2 or higher and clinically significant, the study physician will request repeat laboratory tests. Laboratory results that are Grade 2 or higher and considered to be clinically significant will be followed by the study physician until resolution or stabilization. “Clinically significant” means that the result warrants further medical investigation or treatment because it may have impact on a person’s health status.

Participants will then return to the study clinic 4 weeks after administration of the first study injection (Day 28 + 7). Interim medical histories and concomitant medications will again be reviewed with the participant and blood specimens will be collected for immunogenicity analyses. At visit V2, Day 28 (+7), participants will receive a second study injection (women will have a urine pregnancy test beforehand). If a women is found to be pregnant she will not receive the second study injection and counseling will be provided to them. Blood specimens will be collected for immunological testing prior to administration of study injection. Participants will be carefully monitored for adverse reactions for 30 minutes after study injection.

During the next 7 days following study injection, participants will be asked to record local and systemic signs and symptoms using a Participant Diary, a thermometer, and a local reaction measurement device. Concomitant medications will also be recorded. In addition to solicited signs, participants will be asked to report any other adverse events. Members of the investigator’s clinical team will have contact with participants via phone (C2) 3 days (+2) after study injection to check that participants are correctly completing the Participant Diary and to check on the participants’ well-being.

On the S2 visit, participants will return to the study site seven days after the second study injection, V2+7 (+3) days. At that time, the Principal Investigator or designee will review the Participant’s Diary, confirm the information included and transcribe all solicited and other adverse events onto the case report forms. Blood will be collected for selected biochemical and hematological blood tests as well as immunogenicity testing. Results of the safety laboratory tests will be reviewed by the study physician. The study physician will determine the severity grade of laboratory results using the Toxicity Table for Grading Severity of AEs (see section 9). If the result is Grade 2 or higher and clinically significant, the study physician will request repeat laboratory tests. Laboratory results that are Grade 2 or higher and considered to be clinically significant will be followed by the study physician until resolution or stabilization. “Clinically significant” means that the result warrants further medical investigation or treatment because it may have impact on a person’s health status.

On the I visit, participants will then return to the study clinic 3 weeks after administration of the second study injection, V2+28 (+7) days. Interim medical histories and concomitant medications will again be reviewed with the participant and blood specimens will be collected for immunogenicity analyses.

Participants will be contacted by phone (contact C3) on 194 (+7) daysafter administration of second dose of study product for the closure of any ongoing AEs and concomitant medications; and (b) collection of any SAEs or AESIs and new concomitant medications, if associated with the SAE/ AESI reported. This will complete participant participation in the study, however, if the participant reports any health problems, the PI may use his or her medical judgment and ask the participant to come to the clinic for an examination or any necessary testing.

Safety data review in this study is performed by a DSMB. Safety oversight is described in more detail in section 9.7.

# Study Enrollment and Withdrawal

## 5.1 Description of Participants, Source of Participants

This trial will enroll approximately 432 healthy male and female adults (non-pregnant women) aged 18 to 59 years.

Those who agree to participate in to study will be required to sign the trial informed consent form before enrollment.

The study staff will follow inclusion/exclusion criteria to determine eligibility. Screening tests will be conducted on all people interested in joining the study and their results will be reviewed with them, regardless of eligibility. Screening tests may be considered valid within the range of 30 days between screening and vaccination.

## 5.2 Participant Inclusion Criteria

The following criteria must be met before a participant may be enrolled for participation:

- Healthy male or female (non-pregnant) adults 18 through 59 years of age at the enrollment visit;
- To be available to participate in the study throughout its duration (approximately seven months);
- Healthy, as established by the medical history, physical examination, and screening laboratory evaluations;
- Capable and willing to complete Participant Diaries;
- To demonstrate intention to participate in the study, as documented by signature in the study´s informed consent form;
- For females of child-bearing potential, willing to utilize reliable birth control measures fromV1through at least 60 days following the last study vaccination.

## 5.3 Participant Exclusion Criteria

Participants meeting any of the following criteria will be excluded from participation:

- Participation in another clinical trial involving any experimental therapy within the previous three months or planned enrollment in such a trial during the period of this study.
- Evidence of active neurological, cardiac, pulmonary, hepatic or renal disease as clinical history and/or physical examination (except hypertension under control)
- Compromised infection/immune system diseases including: HIV, Hepattis B and C, diabetes mellitus, cancer (except basal cell carcinoma) and autoimmune diseases.
- Behavioral, cognitive or psychiatric disease that in the opinion of the principal investigator or his representative physician, affects the participant ability to understand and cooperate with all study protocol requirements.
- Abusive usage of alcohol or drugs in the past 12 months that has caused medical, professional or family problems, indicated by clinical history.
- Known systemic hypersensitivity to eggs or to any component of the vaccine.
- History of severe adverse reaction after previous administration of an Influenza vaccine within 6 weeks following vaccination.
- History of Guillain-Barre Syndrome or other demyelinating disease.
- Have a history of severe reactions following previous immunization with licensed or unlicensed influenza virus vaccines.
- Diagnosis of asthma with a history of hospitalization related to this condition in the last six months due to illness.
- Suspected or confirmed fever in the 3 days prior to vaccination or axillary temperature greater than 37.8 ° C on the day of vaccination.
- Use of corticosteroids (except topical or nasal) or other immunosuppressive drugs within 42 days before study initiation/baseline. It will be considered immunosuppressive dose of corticosteroids the equivalent to a dose ≥10 mg of prednisone per day for over 14 days.
- Impaired coagulation due to chronic disease or due to use anticoagulant medication (warfarin or heparin) in the 7 days preceding vaccination.
- Have received live virus vaccine within 28 days or killed virus vaccine in the last 14 days prior to vaccination, or have a scheduled immunization from the first study vaccination until 21 days after the second vaccination.
- Have received any influenza A/H7 vaccine.
- History of asplenia.
- Have received blood products in the past 6 months, including transfusions or immunoglobulin, or scheduled administration of blood products or immunoglobulin for the first 28 days after vaccination
- Any other condition that might put at risk the safety/rights of a potential participant or his/her compliance with this protocol in investigator’s opinion or his representative physician
- Laboratory values at screening equal to or greater than Grade 2 will be considered to be exclusionary. Vital signs may be performed up to three times to allow for transient conditions to resolve. Screening laboratory values that are out of range, but are considered to be due to an acute illness or process may be repeated once. Grade 1 laboratory values will be reviewed by a licensed study clinician and the clinician will determine whether the laboratory abnormality is clinically significant and should be considered exclusionary. If determined to be clinically insignificant, the study team is not required to follow the laboratory until resolution or the value is determined to be clinically stable.

## 5.4 Treatment Assignment Procedures

### 5.4.1 Randomization Procedures

This is a double-blind, placebo-controlled trial with 8 treatment groups to be randomized equally with approximately 54 subjects in each treatment group across all sites based on one randomization schedule.

Each participant will be assigned a unique screening number in this study by the central randomization electronic system after signing the informed consent. After an individual is determined to be eligible for study participation, the participant will be randomized by assigning a unique participant identification number from the randomization schedule.

The complete randomization schedule that contains the participant identification number and the corresponding randomization assignment will be produced using computer software prior to the initiation of the study. The permuted randomization block design procedure will be used to generate the randomization schedules. The randomization will be stratified to maintain the treatment group balance at each study site. The block size for each part will be chosen based on the number of treatment groups and anticipated enrollment sizes at each study site as well.

The Investigator will maintain a screening/enrollment log. The log will contain essential information including participant name, date of screening, gender, date of birth, whether or not the participant meets eligibility criteria, whether participant is enrolled and date, and if not enrolled, reason why the participant is not enrolled or not randomized.

Once a participant identification number has been assigned to a participant, it will not be used again. Additional participants may be randomized into the study at the discretion of the sponsor in the case of any participant who is randomized but does not receive any study vaccine.

### 5.4.2 Blinding and Unblinding Procedures

The randomization will be performed by an organization of an individual independent of the conduct of the study.

This is a double blind study. Participants, investigators, study personnel performing any study-related assessments following study injection, and laboratory personnel performing immunology assays will be blinded to treatment assignment.

The unblinded study product administrator is a study personnel licensed to administer medications/vaccines, but will not be involved in study-related assessments or have participant contact for data collection following study injection.

The DSMB may receive data in aggregate and by group assignment, or may be unblinded to individual study treatment assignments, as needed, to adequately assess safety issues.

Study product injected into each participant will be recorded on the Case Report Form (CRF) using the exact allocation code for each product received by each participant.

The allocation codes link treatment identification with each participant via participant identification numbers. These will be maintained in a secure location, by an individual not involved in the conduct of the study. If any participant experiences an SAE possibly related to receipt of study treatment and the investigator requests unblinding, the study Sponsor will discuss this request with the DSMB and will remain blinded throughout the process.

### 5.4.3 Reasons for Withdrawal

An enrolled/vaccinated participant may be terminated from the study for any of these reasons:

- Participant withdraws consent.
- PI, Sponsor, or the DSMB decides that termination is in the best interest of the participant.
- PI, Sponsor, or the DSMB decides that termination is necessary to protect the integrity of the study or achieve the objectives of the study.
- Interruption of study schedule makes the participant’s data unusable according to protocol requirements.
- The Sponsor terminates the study.

### 5.4.4 Handling of Withdrawals

If a participant withdraws from the study for any reason prior to the planned study duration, every attempt is made to document the participant’s health status and follow procedures outlined in section 7.4.

### 5.4.5 Strategies to Maintain and Recruit Additional Participants

Strategies to maintain participants in the trial include treating them respectfully, making sure they understand the study and potential side effects, and close communication with each participant about how the study unfolds. There is no plan to replace participants who leave the study.

# Study Products

## 6.1 Study Product Descriptions

### 6.1.1 Acquisition

Butantan will provide to the sites the investigational vaccine candidates and placebo for this trial. Butantan will manufacture the H7N9 antigen component of the vaccine candidates.

Butantan will manufacture the IB160 adjvuant. IDRI will manufacture the SE adjuvant and provide it to Butantan. All the vaccine components will be received by Butantan as the Sponsor who is responsible for ensuring receipt, storage and delivery of the vaccine components to the sites.

The unblinded study pharmacist at each site will be responsible for vaccine component receipt, storage and management. Butantan will determine with the sites, the date(s) and time(s) of delivery of vaccine to the study clinic.

### 6.1.2 Formulation, Packaging and Labeling of Vaccine Components

**H7N9 antigen vaccine component**

The H7N9 influenza antigen component produced by Instituto Butantan is an inactivated, split-virus. The antigen was developed using the Candidate Vaccine Virus A/Shanghai/2/2013(H7N9)-PR8 - (IDCDC-RG32A) developed by Center for Disease Control and Prevention (CDC), GA, USA.

The manufacturing process of the vaccine is identical to the one applied for the seasonal vaccine. Briefly, purified influenza virus suspension was grown in embryonated hen´s eggs, inactivated with formaldehyde solution, purified and concentrated by high-speed centrifugation. The vaccine antigen will be mixed with the adjuvants at the clinical sites prior to vaccination of subjects with the vaccine candidate.

**IB160 Adjuvant vaccine component**

IB160 adjuvant is manufactured at Butantan, formulated as 5% v/v squalene, 0.5% w/v Tween 80, 0.5% w/v Span 85 as an oil-in water emulsion in 10 mM citrate buffer pH 6.0-6.5. IB160 appears as a milky-white liquid and presents emulsioned particles with average diameter size of 160 nm. Each vial contains a fill volume of 1.5 ml and must be stored at 2-8°C.

**SE Adjuvant vaccine component**

SE Adjuvant is manufactured by IDRI, formulated as a 4% stable oil-in-water emulsion. SE appears as a milky-white liquid. Each 10 mL vial contains a fill volume of 1.5 mL and must be stored at 2-8°C.

**Formulation of vaccine components**

The H7N9 vaccine candidates will be evaluated at the following concentrations:

3.75 µg H7N9 antigen + Adjuvant IB160, 2.5% oil

7.5 µg H7N9 antigen + Adjuvant IB160, 2.5% oil

15 µg H7N9 antigen + Adjuvant IB160, 2.5% oil

3.75 µg H7N9 antigen + Adjuvant SE, 2% oil

7.5 µg H7N9 antigen + Adjuvant SE, 2% oil

15 µg H7N9 antigen + Adjuvant SE, 2% oil

15 µg H7N9 antigen

The H7N9 antigen will be supplied in *multidose vials* (5 doses per vial) with thimerosal added as a preservative. Adjuvants will be provided separately and mixed at the sites following the mixing strategy in Manual of Procedures.

**Placebo**

Placebo is composed by a PBS solution in multidose vials containintg thimerosal produced by Butantan.

### 6.1.3 Shipment, Storage and Stability

A continuous temperature data logger will be placed inside each carton box to monitor product temperature during the process of transportation, storage, and delivery of the product from Butantan to the study sites.

The vaccine components must not be used if the package or labeling appears to be tampered with, the label is illegible or the physical properties (color and transparency) are altered. In this case or in case of accidental disruption of the cold chain, the products may not be administered and the investigator or the responsible person should contact Butantan to receive further instructions.

Vaccine components must be stored at a temperature between +2 degrees Celsius (°C) to +8°C (in a refrigerator). Storage temperature must be monitored daily and documented on an appropriate form. Back-up power or storage must be available in case of primary power failure. Study vaccine components and placebo must never be frozen.

## 6.2 Dosage, Preparation and Administration of Study Products

### 6.2.1 Dosage and Schedule

A two dose schedule of each (0.5mL) vaccine candidate or placebo (study products) will be evaluated in this study. The study products are to be delivered intramuscularly into the deltoid in alternating arms at 28 days apart. All blood draws for laboratory assessments will be preferably from the non-vaccinated arm so that the participant does not confuse reporting of local pain and other adverse events.

### 6.2.2 Precautions and Warnings

1. The study products are only to be used for healthy adults 18 to 59 years of age participating in this study who meet the inclusion/exclusion criteria. It is forbidden to use the study products for any other purpose.
2. Strict compliance with Brazilian regulations on the use of vaccines and biologicals is required.
3. Study injection must be delivered intramuscularly into the deltoid of the participant’s non-dominant arm.
4. Any study materials that have had temperature excursions may not be used. Contact Butantan for further instructions.
5. Only health workers who have been trained for this research study may administer study injections to enrolled participants, assuring proper intramuscular injection technique and sterile injection.
6. Only one person may be injected with one needle and syringe.
7. Study participants must remain in the clinic and be monitored for 30 minutes after study injection.
8. The study sites must have adequate facilities for monitoring and treating any reactions. Prompt referral to additional needed facilities must be available.

### 6.2.3 Administration of study products

To prepare and administer study product, study sites and investigators must comply with any applicable regulations. The step-by-step instructions for the investigator (or delegated staff) are found in the Manual of Procedures.

## 6.3 Accountability Procedures for Study Products

The study products will be kept in a secure place in cold storage at the study site segregated from other products. During the study staff in charge of research product management will record information related to the delivery of study products to the trial site, conduct inventory at the trial site, check the number of doses given to the participants, check the number of unused or partially unused vials, and return these to Butantan after completing the study, if directed.

## 6.4 Assessment of Compliance with Use of the Study Products

Compliance with use of the study products will be closely monitored during the trial by the research team and trial monitors.

## 6.5 Concomitant Medications/Treatment

Concomitant medications will be documented throughout the course of the study.

Treatment of conditions that are not exclusionary should continue, if needed by the participant. Subsequent changes in concomitant treatment during the trial must also be reflected in the CRF.

Women included in the trial who are using hormonal contraception at the time of enrollment should continue using these products through 60 days after the last vaccine dose. Use of such products or any other methods of contraception must also be documented in the CRF. Condoms may be provided to all volunteers upon request.

## 6.6 Unauthorized Products

The following products are not authorized to be used during the study:

1. Any concomitant medicine or biologic specifically prescribed for the treatment of a condition which is an exclusion criterion for participation in the trial.
2. All non-study vaccines or biologics (including blood products).

Other concomitant products are allowed.

Participants will be requested not to take analgesic or antipyretic drugs *in a preventive way* (before or soon after injection)*,* as such medications might change the reactogenicity profiles of study vaccine and placebo.

# Study Schedule; Description of Visits

## 7.1 Screening/Triage (visit T)

Prior to inclusion into the vaccine study, each participant will be screened by means of multiple procedures, including laboratory testing, medical history interview and targeted physical examination. Because screening procedures are required to assess eligibility, they will be performed only after consent is obtained. The Principal Investigator or designee will record the identification of all participants who enter screening; whether they entered the trial or failed screening, and the reason for screen failure on the screening/enrollment log.

Screening visits can occur upto 30 days before V1 (first study injection).

After participants are consented, the following activities will occur:

1. The participant will be interviewed to collect baseline demographic data (date of birth or year of birth if specific date not known, gender, race/ethnicity, etc.).
2. A study clinician will interview the participant to collect a detailed medical history including concomitant medications. Study staff will review the information to confirm eligibility prior to conduct of further procedures.
3. A study clinician will perform a targeted physical examination. Results will be reviewed by study staff and with the participant to confirm eligibility prior to conduct of further procedures. If a person is considered ineligible based on medical history and physical examination, the study clinician will tell the person that he or she is not eligible, the reasons will be explained and referral for appropriate care made if necessary.
4. Blood (serum and whole) specimens will be collected for biochemical and hematological testing. Serum specimens will also be collected for testing for chronic viral hepatitis infections as well as HIV. Specimen collection information must be documented in the relevant source documentation. About 17,5 mLs of blood will be collected.
5. For women, a urine pregnancy test will be done.
6. Study staff will instruct the participant on when to return for screening results and possible enrollment into the study.

## 7.2 Study Injections and Follow-up Periods

### 7.2.1 Day of Study Injection (V1)

Visit V1 is the day of injection of study product. It can occur up to 30 days after the screening visit.

1. Study staff will confirm participant identity.
2. A study clinician will review results of all screening laboratory testing with the participant. Appropriate referrals will be conducted for any abnormalities. All results will be reviewed to confirm eligibility prior to conduct of further procedures.
3. Study staff will inquire about any new medical events since medical histories were recorded and confirm eligibility prior to conduct of further procedures.
4. A study clinician will perform a targeted physical examination if necessary. Results will be reviewed by study staff and with the participant to confirm eligibility prior to conduct of further procedures.
5. Women will have a urine pregnancy test to confirm there is no pregnancy on the day of vaccination.
6. Eligibility is confirmed after the revision of inclusion/exclusion criteria and recorded on the study CRF.
7. A study clinician will review the study procedures with each eligible participant.
8. Prior to administration of study product, a blood specimen will be collected by venipuncture from the participant’s **dominant arm** preferably for anti-influenza serologic assays. Specimen collection information must be documented on the CRF and in specimen collection logs. About 30 mLs of blood will be collected.
9. The person in charge of performing the injection will confirm that the study product about to be injected is labeled with the correct participant identification.
10. The participant will be administered one injection of the investigation product in the deltoid muscle of the non-dominant arm.
11. The participant will be observed for 30 minutes after administration. Local and systemic reactions occurring in the first 30 minutes will be recorded.
12. The participant will be given a Participant Diary, a digital thermometer and a local reaction measurement devicein which the participant will be asked to record any local and/or systemic reactions that might appear within 7 days post vaccination, and record any concomitant medications. The participant will be instructed how to use the Participant Diary, thermometer and a local reaction measurement device. All relevant explanations should be included in the Participant Diary. The Participant Diary will also have contact information for the investigators, should the participant have any questions. The participant will be informed that a member of the investigator’s team will call on V1+3 to check on the participant’s completion of the Participant Diary and participant’s well-being.
13. The participant will be instructed that if the participant experiences an AE requiring medical care, the participant should inform the study staff as soon as possible and seek medical care as appropriate. If the participant visits a health care provider, the participant should be sure to inform the health care provider of participation in this study and provide the health care provider with the investigator’s contact information.

### 7.2.2 First Week after Study Injection (contact C1)

1. The participant will complete the Participant Diary daily, reporting any local or systemic reactions, daily temperature and medications taken.
2. The participant will have been instructed that if he/she experiences an AE requiring medical care, the participant should inform the study staff as soon as possible and seek medical care as appropriate. If the participant visits a health care provider, the participant should be sure to inform the health care provider of participation in this study and provide the health care provider with the investigator’s contact information.
3. Three days after study injection (V1+3) study staff will call the participant (contact C1) to check that the participant is correctly completing the Participant Diary and to check on the participant’s well-being. If any concern arises from the safety assessment over the phone the participant will be directed to come to the study site.

### 7.2.3 Seventh Day after Study Injection (visit S1)

1. Study staff will confirm participant identity.
2. Study staff will review the Participant Diary and interim history with the participant and inquire about any new medical events since medical histories were last updated. Any AEs that have occurred will be recorded in the appropriate CRFs.
3. A study clinician will perform a targeted physical examination and record the information on the CRF. Results will be reviewed by study staff and with the participant and any AEs that have occurred will be recorded in the appropriate CRF.

Blood specimens will be collected for testing selected biochemical parameters (total bilirubin, ALT, AST, creatinine) and selected hematologic studies (WBC, Hgb, and platelets). Specimen collection information must be documented on the CRF and in specimen collection logs. About 9 mLs of blood will be collected.

1. Laboratory results will be reviewed within 36 hours of receipt by the study physician. Any out-of-normal-range result Grade 2 and above will be evaluated by the study physician to assess whether it is of clinical significance and whether it is related to vaccination. Laboratory results that are Grade 2 or above and considered to be clinically significant will be followed by the study clinician until resolution or stabilization.
2. The participant will be instructed to inform the study staff as soon as possible if he/she experiences an AE requiring medical care and to seek medical care as appropriate. If the participant visits a health care provider, the participant should be sure to inform the health care provider of participation in this study and give the health care provider the contact information for the investigator. Study staff may request to review any document related to participant´s visits or hospitalizations handed to study staff by participants (e.g.: hospital discharge summaries, etc), and considered relevant to the assessment of potential adverse events, and if permitted by the participant, extra information may be requested by study staff to other health care services/providers.

### 7.2.4 Second and Third weeks after Study Injection

There are no scheduled visits during this period. Participants may be asked to come to the study clinic if the investigator follows up on any laboratory or clinical issue. The participant will inform the study clinic of any medical events and seek medical care as needed. The participant will be instructed to give any health care provider they see the contact information for the investigator.

### 7.2.5 Immunogenicity Visit and Second Study Injection (visit V2)

The Visit V2 is scheduled 28 days after V1 for collection of immunogenicity samples for the study and to administer the second dose of vaccine or placebo.

1. Study staff will confirm participant identity.
2. Eligibility criteria will again be reviewed
3. Study staff will review interim medical histories, including any unsolicited AE and concomitant medications with the participant since these were last updated. Results will be recorded in the appropriate section of the CRFs.
4. Women will have a urine pregnancy test to confirm there is no pregnancy on the day of study injection.
5. Prior to administration of study product, a blood specimen will be collected by venipuncture from the participant for immunogenicity testing. Specimen collection information must be documented on the CRF and in specimen collection logs. About 40 mLs of blood will be collected.
6. The person performing the injection will confirm that the study product about to be injected is labeled with the correct participant identification.
7. The participant will be administered one injection of study product in the deltoid muscle of the non-dominant arm.
8. The participant will be observed for 30 minutes after administration. Local and systemic reactions occurring in the first 30 minutes will be recorded.
9. The participant will be given a Participant Diary, a digital thermometer and a local reaction measurement devicein which the participant will be asked to record any local and/or systemic reactions that might appear within 7 days after V2, and record any concomitant medications. The participant will be instructed how to use the Participant Diary, thermometer and a local reaction measurement device. All relevant explanations should be included in the Participant Diary. The Participant Diary will also have contact information for the investigators, should the participant have any questions. The participant will be informed that a member of the investigator’s team will call on visit V2+3 to check on the participant’s completion of the Participant Diary and participant’s well-being.
10. The participant will be instructed that if the participant experiences an AE requiring medical care, the participant should inform the study staff as soon as possible and seek medical care as appropriate. If the participant visits a health care provider, the participant should be sure to inform the health care provider of participation in this study and provide the health care provider with the investigator’s contact information.

### 7.2.6 First Week after second Study Injection

1. The participant will complete the Participant Diary daily, reporting any local or systemic reactions, daily temperature and medications taken.
2. The participant will have been instructed that if he/she experiences an AE requiring medical care, the participant should inform the study staff as soon as possible and seek medical care as appropriate. If the participant visits a health care provider, the participant should be sure to inform the health care provider of participation in this study and provide the health care provider with the investigator’s contact information.
3. Three days after second study injection (V2+3) study staff will call the participant (contact C2) to check that the participant is correctly completing the Participant Diary and to check on the participant’s well-being. If any concern arises from the safety assessment over the phone the participant will be directed to come to the study site.

### 7.2.7 Seventh Day after second Study Injection (visit S2)

1. Study staff will confirm participant identity.
2. Study staff will review the Participant Diary and interim history with the participant and inquire about any new medical events since medical histories were last updated. Any AEs that have occurred will be recorded in the appropriate CRF.
3. A study clinician will perform a targeted physical examination and record the information on the CRF. Results will be reviewed by study staff and with the participant and any AEs that have occurred will be recorded in the CRF.

Blood specimens will be collected for testing selected biochemical parameters (total bilirubin, ALT, creatinine) and selected hematologic studies (WBC, Hgb, and platelets) and for immunogenicity testing. Specimen collection information must be documented on the CRF and in specimen collection logs. About 40 mLs of blood will be collected.

Laboratory results will be reviewed by the study physician. Any out-of-normal-range result Grade 2 and above will be evaluated by the study physician to assess whether it is of clinical significance and whether it is related to vaccination. Laboratory results that are Grade 2 or above and considered to be clinically significant will be followed by the study clinician until resolution or stabilization.

The participant will be instructed to inform the study staff as soon as possible if he/she experiences an AE requiring medical care and to seek medical care as appropriate. If the participant visits a health care provider, the participant should be sure to inform the health care provider of participation in this study and give the health care provider the contact information for the investigator. Study staff may request to review any document related to participant´s visits or hospitalizations handed to study staff by participants (e.g.: hospital discharge summaries, etc), and considered relevant to the assessment of potential adverse events, and if permitted by the participant, extra information may be requested by study staff to other health care services/providers.

### 7.2.8 Second and Third weeks after second Study Injection

There are no scheduled visits during this period. Participants may be asked to come to the study clinic if the investigator follows up on any laboratory or clinical issue. The participant will inform the study clinic of any medical events and seek medical care as needed. The participant will be instructed to give any health care provider they see the contact information for the investigator.

### 7.2.9 Final Study Visit (visit I)

The visit I, schedule for day V2+28is the last in-clinic visit for the participant for collection of immunogenicity samples and review of unsolicited AE for the study.

1. Study staff will confirm participant identity.
2. A study clinician will perform a physical examination and record the information in the CRF. The results will be reviewed by the study team and with the participant and any AEs that have occurred will be recorded in the CRF
3. Study staff will review interim medical histories including any unsolicited AE and concomitant medications with the participant since these were last updated. Results will be recorded in the appropriate CRFs.
4. Blood specimens will be collected for immunogenicity testing. Specimen collection information must be documented on the CRF and in specimen collection logs. About 30,5 mLs of blood will be collected.

## 7.3 Final Study Call (contact C3)

## The contact C3 is schedule for day V2+194 and it is the last contact with the participant.

1. The participant will be contacted via telephone and identity confirmed.
2. Study staff will review interim medical histories and concomitant medications with the participant since these were last updated. No new concomitant medication will be recorded unless it relates to a newly identified SAE/AESI.
3. Study staff will ask about any medical event that would constitute an SAE since the last visit.
4. Study staff will ask if the participant has been hospitalized or had any new medically confirmed disease(s) since the last visit. Study staff will review any AESIs and report accordingly. If deemed clinically necessary, a visit to the study site will be scheduled.
5. No new AE information will be recorded unless it qualifies as an SAE or an AESI.
6. If an SAE/AESI is reported, the clinician should record the event on the appropriate form, notify the entities who require notification, and refer the participant for treatment of the SAE/AESI, if warranted. Study staff may request to review any document related to participant´s visits or hospitalizations handed to study staff by participants (e.g.: hospital discharge summaries, etc), and considered relevant to the assessment of the SAE and if permitted by the participant, extra information may be requested by study staff to other health care services/providers.
7. After recording the information, the participant will be discharged from the study.

## 7.4 Early Termination Visit

If a participant withdraws from the study for any reason prior to the planned study duration, every attempt is made to complete the following:

- Report of local and systemic reactogenicity and AEs are reviewed by PI (or designee).
- Specimens are obtained for chemistry, hematology and immunogenicity analysis if withdrawal occurs prior to scheduled safety laboratory testing.
- Participant Diary information is reviewed with the volunteer in detail by site staff, if in use since the last visit.
- Injection site(s) examination and a full physical examination are performed (if indicated).

Butantan must be informed of all instances of the premature termination of a participant’s participation in the trial.

If the participant develops a reaction to study vaccine which the investigator believes threatens the participant’s well-being, the withdrawn participant must be treated or transferred to a treatment facility.

## 7.5 Unscheduled Visits

Participants may present to the study center during operating hours for an unscheduled visit should they experience any AE, if a laboratory sample is to be repeated, or if the participant’s condition requires medical intervention. Data for any examinations or other procedures performed on the participant at an unscheduled visit must be recorded on the appropriate CRFs.

## 7.6 Termination of the Trial

### 7.6.1 End of Trial According to the Protocol

The end of the trial is defined as the date of the last visit/last phone contact of the last participant participating in the trial, according to the trial scheme.

### 7.6.2 Suspension and/or Premature Termination of the Trial

The trial or an arm of the trial can be suspended at any time by the Sponsor, any ethical review committee overseeing this study and as recommended by the DSMB for any safety concern. This includes, for example, and without limitation, an SAE resulting in death or an unusually high rate of SAEs.

# Study Evaluations

## 8.1 Clinical Evaluations

Safety evaluations will be made by a clinician on visits V1, V1+7, V2, V2+7 and V2+28 through targeted physical exams. Evaluation must be performed before and after administration of study product on V1 (day 0) and V2 (day 28). Reported signs and symptoms will be recorded by the participant on Participant Diary from Days 1-7 and Days 28-35 in the study.

### 8.1.1 Medical History

At enrollment, medical histories must be thoroughly reviewed with the participant. The following medical conditions, in particular, will be assessed:

- Current or recent (within two weeks of enrollment) acute illness with or without fever.
- Recent vaccination history.
- Recent receipt of immune globulin or other blood products, or injected or oral corticosteroids or other immune modulator therapy within 6 weeks before enrollment.
- Hypersensitivity of any kind, but particularly to vaccines.
- Asthma.
- Clinically relevant history of renal, gastrointestinal, hepatic, cardiovascular, hematological, dermatological, endocrine, neurological, or immunological diseases.
- Seizures, including history of febrile seizures, or any other neurologic disorder.
- Known or suspected immunologic impairment of any kind.
- Known HBV or HCV infection.
- Known HIV infection.
- Alcohol or illegal drug use.
- Medications currently taken.
- For women, pregnancy, menstrual and contraceptive history and/or history of surgical sterility.

### 8.1.2 Physical Examination

General Physical Examinations

Qualified study clinicians will conduct a physical examination of all participants at screening. This physical examination will include the following:

- Recording of general appearance
- Height and weight (only needed at one screening visit)
- Targeted physical examination
- Measurement of the following vital signs:
  - body temperature (and body location of measurement)
  - blood pressure
  - pulse and/or heart rate

*Out-of-range observations may not be considered to be exclusionary at screening nor an adverse event (AE) for the study, unless judged to be clinically significant by the PI.

## 8.2 Laboratory Evaluations

### 8.2.1 Clinical Laboratory Evaluations

All biochemical and hematological testing will be performed at a laboratory associated with the study sites in Brazil. The laboratories should follow GLP standards.

Laboratories will follow written procedures for conducting all laboratory assays.

Normal value ranges will be considered according to each clinical laboratory conducting tests. A Toxicity Table for Grading AEs, including out-of-range laboratory values, is included in section 9.3.

A laboratory result that is out-of-range but no greater than Grade 1 does not qualify as an AE and does not require determination of clinical significance or relatedness to the study products. A result will be checked as not clinically significant “NCS” on the laboratory result CRF if it is outside the lab range but lower than Grade 2.

Serology for Viral Infections (Screening only)

Blood specimens will be evaluated for the following using tests routinely used in the laboratories:

- Hepatitis B virus surface antigen (HBsAg)
- HCV antibody using anti-HCV test
- HIV serology testing

Specimen collection will occur on Day of screening. Pre and post-test counseling will be offered to participants according to protocols of the Brazilian Ministry of Health. Likewise, patients who test positive for any of the serological tests will be referred for appropriate prevention, treatment and care services. There will be coordination with laboratory services to support quality assurance and delivery of correct results. Determinant results of a participant’s testing must be obtained from the laboratory and evaluated prior to administration of any study product.

Pregnancy Test

In order to confirm pregnancy status of females, a qualitative human chorionic gonadotropin (hCG) test will be done on a urine sample collected on Day of Screening and before each study injection. No injection of study product may be given to a woman without a pregnancy test being done and to any woman with a positive pregnancy test.

Blood Chemistry and Hematology

Specimen collection will occur on visits T, S1, V2 e S2. Results of testing for the participant will be reviewed by a study physician. Considering the tests of visits S1 and S2, out-of-range results Grade 2 and above will be assessed for clinical significance and relatedness to study injection and repeated. Any results that are Grade 2 or above and clinically significant should be followed until normal or stabilized prior to termination of participant participation in the study. “Clinically significant” means the result warrants further medical investigation or treatment because it may have impact on a person’s health status. If the clinician is unsure of clinical significance, the specimen should be re-drawn and the test re-run.

Blood Chemistry

- Creatinine
- Alanine aminotransferase (ALT, also called SGPT)
- Aspartate aminotransferase (AST, also called SGOT)
- Total bilirubin

Hematology

- Number of WBC and differentials
- Hemoglobin (Hgb)
- Hematocrit (Hct)
- Platelet count

### 8.2.2 Immunogenicity Assays

Immunogenicity testing will be performed by qualified laboratories which are reference on influenza, namely Instituto Adolfo Lutz and Fundação Oswaldo Cruz using qualified assays already in established protocols. For these assays, serum specimen collection will occur on visits V1 (prior to injection of study products), V2 (prior to injection of study products), S2 (HI only) and I.

Serum Antibody to Influenza Virus Detected by Hemaggluttinin Inhibition Assay (HI)

The HI is the most frequently used serologic test for determining immunologic response to influenza vaccination. Serum specimens will be tested for the presence and titer of HI antibodies to the H7N9 influenza strain represented in the vaccine at a qualified laboratory according to established procedures. The HI assay will be conducted on serum samples from all the participants of the study in one or more Brazilian laboratories, and may also be performed in a laboratory abroad in order to confirm the results found in the laboratory (s) in Brazil, if deemed necessary by the parties involved in conducting the study.

Serum Antibody to Influenza Virus Detected by Microneutralization Assay (MNT)

The microneutralization assay is an alternative test for determining immunologic response to influenza vaccination and will be conducted in serum samples from participants as asecondary immunogenicity endpoint by one or more qualified laboratory (which may be located outside Brazil).

Regarding the sending of samples abroad, all aspects of the Brazilian laws will be complied with, according to resolution CNS no. 441 of 2011 in item 14.

### 8.2.3 Preparation, Processing and Specimens

Blood Specimens

Blood will be collected according to universal precautions for testing in multiple assays. Serum for the following: blood chemistry, HBV, HCV and HIV serology, HI antibody serology. Whole blood is collected for WBC, Hgb, Hct, and platelets.

|  | **Volume of blood** | **Day of Screening** | **V1**  **(pre-vac)** | **S1** | **V2**  **(pre-vac)** | **S2** | **I** |
| --- | --- | --- | --- | --- | --- | --- | --- |
| Whole blood for Hematology | 4 mL | ✓ |  | ✓ | ✓ | ✓ |  |
| Serum for serology for HBV, HCV and HIV infection | 8,5 mL | ✓ |  |  |  |  |  |
| Serum for biochemical parameters | 5 mL | ✓ |  | ✓ | ✓ | ✓ |  |
| Serum for anti-influenza serologic assays | 30,5 mL |  | ✓ |  | ✓ | ✓ | ✓ |

(The total volume of blood planned to be collected from each participant during the course of the study will be less than 170 milliliters.)

# Assessment of Safety and Adverse Events

## 9.1 Definition and Categorization of AEs

The primary objective of this study is to evalute the safety profile and immunogenicity of the vaccine candidates. An AE (adverse event) is defined as any untoward medical occurrence in a participant administered an study product, which may or may not bear a causal relationship with this study injection. An AE can therefore be any unfavorable and unintended sign (including an abnormal laboratory finding), symptom, or disease temporally associated with the use of a medicinal product, whether or not considered related to the medicinal product. A subset of AEs, those commonly observed after influenza vaccine administration are described in the Participant Diary and will be specifically requested for 7 days post each study injection. Those AEs are termed “solicited” and will be evaluated for potential relationship with study product. Out-of-range laboratory abnormalities observed after study injections will be graded according to the grading table (see Table 5) and their clinical significance and potential relationship to study product assessed. Only those laboratory abnormalities greater than Grade 1 will be recorded as AEs.

All AEs should be graded using the Tables for Grading AEs (Tables 4, 5 and 6). These tables contain list of specific clinical and laboratory safety measurements of interest to the study. All clinical safety evaluations must be made by a qualified physician.

Any clinical sign or symptom or laboratory finding at any time-point might be categorized as an SAE if it meets the criteria established.

All adverse events will be MedDRA coded.

## 9.2 Specification of Safety Parameters

The safety profile will be evaluated by the proportion of participants experiencing AEs, related or not related, of the following four categories:

- Adverse events occurring over the immediate 30-minute post each study injection.
- Number and percentage of participants with solicited local AE (erythema, swelling/induration, pain/tenderness, ecchymosis, pruritis) over the 7-day period post each study injection.
- Number and percentage of participants with solicited systemic AEs (fever, fatigue/malaise, myalgia, arthralgia, chills, nausea/vomiting, and headache) over the 7-day period post each study injection.
- Number and percentage of participants with unsolicited adverse events for 28 + 7 days post each study injection.
- All serious adverse events (SAEs) occurring over the entire study period.

## 9.3 Methods and Timing for Assessing and Recording Safety Parameters

### 9.3.1 Adverse Events

The occurrence of an AE might come to the attention of study personnel during study visits and interviews of a study participant presenting for medical care. Information to be collected on AEs includes event description, time of onset, clinician’s assessment of severity, relationship to study product, and time of resolution/stabilization of the event. AE assessment should be made only by those with the training and authority to make a diagnosis.

Any medical condition that was present at the time that the participant was enrolled should not be reported as an AE, but should be reported as a pre-existing condition on the Medical History Form. However, if this condition occurs with greater frequency or severity during the study, it should be recorded as an AE.

AEs, including laboratory abnormalities that are Grade 2 and above, should always be assessed for severity and relationship to study products.

This study will collect and record solicited and unsolicited local and systemic AEs (described in further detail in section below). For clarity, solicited AEs refer specifically to those asked about during the 7-day post study injection period. Unsolicited events are those not specifically solicited, but may be reported by the participant at any time or observed by study staff while the participant is at a clinic for a study visit. Unsolicited events are recorded up through 28 + 7 days post each study injection, except for SAEs, which are recorded throughout the entire study period as required (V2+194 days). If a new solicited event is reported by the participant outside of the 7-day post-vaccination window, this event will be recorded as an “unsolicited AE” because it will be outside of the 7-day window for collection of solicited reactogenicity.

### 9.3.2 Severity of Event

The severity of the solicited clinical adverse events (local and systemic), laboratory events and unsolicited AEs will be classified by a numeric scale ranging from 1 to 4, according to Table 4, 5, 6, created based on the FDA guidance.^[[22]](#endnote-23)^

**Table 4 Classification of the severity of the solicited clinical adverse events.**

| **Solicited Local Adverse Events** | | | | |
| --- | --- | --- | --- | --- |
| **Adverse Event** | **Grade 1** | **Grade 2** | **Grade 3** | **Grade 4** |
| Pain/tenderness at the injection site of the investigational product | Does not interfere  with daily activities | Repeated use of non-narcotic analgesic >24 hours  OR  Mild interference with daily activities | Any use of a narcotic analgesic  OR  Prevent daily activities | Emergency department visit*  OR  Hospitalization |
| Erythema at the injection site of the investigational product ^†^ | 25 – 50 mm | 51 – 100 mm | >100 mm | Necrosis  OR  Exfoliative dermatitis |
| Swelling/Induration at the injection site of the investigational product ^†^ | 25 – 50 mm | 51 – 100 mm  OR  Mild interference with daily activities | >100 mm | Necrosis |
| Ecchymosis at the injection site of the investigational product ^†^ | 25 – 50 mm | 51 – 100 mm | >100 mm | Necrosis |
| Pruritus at the injection site of the investigational product | Does not interfere  with daily activities | Mild interference with daily activities | Prevent daily activities | Emergency department visit*  OR  Hospitalization |
| **Solicited Systemic Adverse Events** | | | | |
| Nausea/vomiting | Does not interfere  with daily activities  OR  1 to 2 episodes in 24 hours | Mild interference with daily activities  OR  More than 2 episodes in 24 hours | Prevent daily activities, it requires IV hydration | Emergency department visit*  OR  Hospitalization  OR  Hypovolemic shock |
| Fever | 37.8 – 38.4°C | 38.5 – 38.9°C | 39.0 – 40.0°C | >40°C |
| Chills | Slight cold sensation; chills, teeth chatter | Moderate chills in the entire body, it requires the use of narcotics | Serious or prolonged, no response to narcotics | ----- |
| Headache | Does not interfere  with daily activities | Repeated use of non-narcotic analgesic >24 hours  OR  Mild interference with daily activities | Any use of a narcotic analgesic  OR  Prevent daily activities | Emergency department visit*  OR  Hospitalization |
| Fatigue/malaise | Does not interfere  with daily activities | Mild interference with daily activities | Prevent daily activities | Emergency department visit*  OR  Hospitalization |
| Myalgia | Does not interfere  with daily activities | Mild interference with daily activities | Prevent daily activities | Emergency department visit*  OR  Hospitalization |
| Arthralgia | Does not interfere  with daily activities | Mild interference with daily activities | Prevent daily activities | Emergency department visit*  OR  Hospitalization |

* It requires 12 hours or more of admission to a ward or emergency department for the management of the adverse event.

^†^ The value recorded should be measured at the largest diameter and as a continuous variable.

**Table 5 Classification of the severity of the laboratory adverse events.**

| **Adverse event** | **Grade 1** | **Grade 2** | **Grade 3** | **Grade 4** |
| --- | --- | --- | --- | --- |
| Hemoglobin decrease compared to baseline - gm/dL | 0.1 – 1.5 | 1.6 – 2.0 | 2.1 – 5.0 | >5.0 |
| Hemoglobin (women) - gm/dL | 11.0 – 12.0* | 9.5 – 10.9 | 8.0 – 9.4 | <8.0 |
| Hemoglobin (men) - gm/dL | 12.5 – 13.5* | 10.5 – 12.4 | 8.5 – 10.4 | <8.5 |
| Leukocytosis – cell/mm3 | 10,800^†^– 15,000 | 15,001 – 20,000 | 20,001 – 25,000 | >25,000 |
| Leukopenia – cell/mm3 | 2,500 – 3,500* | 1,500 – 2,499 | 1,000 – 1,499 | <1,000 |
| Lymphopenia – cell/mm3 | 750 – 1,000* | 500 – 749 | 250 – 499 | <250 |
| Neutropenia – cell/mm3 | 1,000 – 1,499* | 500 – 999 | 300-499 | <300 |
| Platelets Decreased – cell/mm3 | 125,000 – 140,000* | 100,000 – 124,000 | 25,000 – 99,000 | <25,000 |
| Creatinine – mg/dL | 1.5^†^ – 1.7 | 1.8 – 2.0 | 2.1 – 2.5 | >2.5 or requires dialysis |
| ALT/AST – increase by factor | 1.1 – 2.5 x ULN | 2.6 – 5.0 x ULN | 5.1 – 10 x ULN | >10 x ULN |
| Bilirubin – when accompanied by any increase in ALT/AST – increase by factor | 1.1 – 1.25 x ULN | 1.26 – 1.5 x ULN | 1.51 – 1.75 x ULN | >1.75 x ULN |
| Bilirubin – when ALT/AST are normal – increase by factor | 1.1 – 1.5 x ULN | 1.6 – 2.0 x ULN | 2.0 – 3.0 x ULN | >3.0 x ULN |

ILN: inferior limit of the normal range.

ULN: upper limit of the normal range.

* If the ILN is less than the upper limit of Grade 1, the ILN will be used as an upper limit to Grade 1.

^†^ If the ULN is greater than the lower limit to Grade 1, the ULN will be used as a lower limit to Grade 1.

**Table 6 Classification of the severity of the unsolicited clinical adverse events.**

| **Adverse event** | **Grade 1** | **Grade 2** | **Grade 3** | **Grade 4** |
| --- | --- | --- | --- | --- |
| Clinical adverse event (as defined by this protocol) | Does not interfere  with daily activities | Mild interference with daily activities | Prevent daily activities | Emergency department visit*  OR  Hospitalization |

* It requires 12 hours or more of admission to a ward or emergency department for the management of the adverse event.

All cases of death will have their severity classified as Grade 4.

The worst severity reported to an adverse event, until its resolution or conclusion, will be the severity used to perform the study analysis.

### 9.3.3 Causal relationship classification

All adverse events should have their causal relationship to the investigational product classified by the principal investigator or a medical officer, following the adapted classification of the "Uppsala Monitoring Centre" of the World Health Organization (WHO-UMC), described in (Table 7). The sponsor may request additional clarification from the principal investigator to justify the causal relationship attributed to the event.

A causal relationship may also be revised at the request of the DSMB following a written justification.

**Table 7 Classification of the causal relationship of the adverse events to the investigational product**

| **A reasonable causal relationship** | | | **A causal relationship NOT reasonable** | |
| --- | --- | --- | --- | --- |
| The Adverse Event is considered an Adverse Reaction | | | The Adverse Event cannot be considered an Adverse Reaction | |
| **Certain** | **Probable** | **Possible** | **Unlikely** | **Not related** |
| A clinical event, including a laboratory test abnormality (abnormal value) with a plausible temporal relationship to the administration of the intervention; | A clinical event, including a laboratory test abnormality (abnormal value) with a reasonable temporal relationship to the administration of the intervention; | A clinical event, including a laboratory test abnormality (abnormal value) with a reasonable temporal relationship to the administration of the intervention; | A clinical event, including a laboratory test abnormality (abnormal value) that due to the timing of the administration of the intervention has an unlikely relationship, but not impossible; | A clinical event, including a laboratory test abnormality (abnormal value) that due to the timing of the administration of the intervention has no relationship; |
| It cannot be explained by a concurrent disease or other intervention or medication;  The event must be definitive pharmacologically or phenomenologically (i.e., is an objective and specific disorder or a pharmacologically recognized phenomenon); | It is unlikely to be attributed to a concomitant disease or other medication or intervention; | It can also be explained by a concurrent disease or other medication or interventions; | Another disease or another medication provides a plausible explanation. | Another disease or another medication provides a plausible explanation. |
| The response to an interruption or withdrawal is plausible (pharmacologically, pathologically); | The response to the interruption or withdrawal is clinically reasonable; | There is a lack of information or lack of clarity about the withdrawal or interruption of treatment. |  |  |
| A satisfactory rechallenge procedure if necessary. | A rechallenge procedure is not necessary. |  |  |  |

### 9.3.4 Solicited Reactogenicity (Expected Reactions)

Immediate Reactions (within 30 minutes)

All participants will be observed for 30 minutes after administration of study product, with appropriate medical treatment readily available in case of an anaphylactic reaction following the administration of study product. Immediate reactions will be assessed by a study physician or appropriately trained medical staff. All reactions that occur during this time will be recorded on the CRF. Any immediate reaction which meets the criteria for an SAE must also be documented on an SAE form.

Emergency medicines will be available at the study clinic to be ready to give first aid if any adverse reactions or events should occur among any participant participating in this research.

### 9.3.5 Solicited Local and Systemic Reactions

Specific local and systemic reactions will be solicited (specifically asked of participants) while participants are in the study. These specific reactions, which are signs and symptoms, will be graded by a study clinician using the Toxicity Table for Grading Adverse Events (see Toxicity Tables 4, 5, 6). Grading will use predefined scales based on functional assessment or magnitude of reaction, where available. Where grading scales are not provided, the reaction will be graded for severity based on interference with participant functionally, as for all other AEs (see Section 9.3.2). Severity of redness, swelling and induration at the injection site should always be graded based on size.

**Local reactions:**

- Size of erythema (at site of injection) in cm
- Size of swelling/ induration (at site of injection) in cm
- Pain/Tenderness (at site of injection)
- Pruritus (at site of injection)
- Eccymosis (at site of injection) in cm

**Systemic Reactions:**

- Fever-Body temperature (and location of measurement)
- Fatigue/malaise
- Myalgia
- Arthralgia
- Chills
- Nausea/Vomiting
- Headache

### 9.3.6 Unsolicited Adverse Events

Unsolicited adverse events are any AEs that occur any time after the vaccine/placebo is given (temporally related to study product), whether or not deemed “related” to the product, and are not solicited (not specifically asked of the participant). Unsolicited AEs can be observed by study staff while the participant is at a clinic for a study visit or reported by the participant at any time. Any solicited sign or symptom starting after 7 days post- each vaccination will be recorded as an “unsolicited AE”.

### 9.3.7 Serious Adverse Events

An SAE is defined as an AE that meets one of the following conditions:

- Death.
- Life-threatening (participant at immediate risk of death.) (The term “life-threatening” in the definition of “serious” refers to an event in which the patient was at risk of death at the time of the event; it does not refer to an event that hypothetically might have caused death if it were more severe).
- Requires inpatient hospitalization or prolongation of existing hospitalization.
- Results in congenital anomaly/birth defect. (Only in the case of a woman becoming pregnant during the study period after administration of at least one injection of study product. All pregnancies must be followed to term and outcome reported to Butantan and regulatory agencies.)
- Results in a persistent or significant disability or incapacity.
- Important medical events that might not result in death, be life-threatening, or require hospitalization might be considered SAEs when, based upon appropriate medical judgment, the event might jeopardize the well-being of the participant and require medical or surgical intervention to prevent one of the outcomes listed above. (Medical and scientific judgment should be exercised in deciding whether reporting these events is appropriate.)

All SAEs occurring within 4 weeks of each study vaccination must be reviewed and evaluated by a study clinician (SAE relationship to study vaccine must be evaluated and recorded on an SAE form and reported, as specified in Section 9.4). All such SAEs should also be followed until satisfactory resolution or until the investigator deems the event to be chronic or the participant to be stable.

### 9.3.8 Procedures for Out-of-range Laboratory Test Values

Out-of-range laboratory values and AEs. To the extent possible, all reference ranges for clinical laboratory test results will be pre-specified. The site staff will follow the Toxicity Table (Table 5). Laboratory values that are out-of-range will not be considered an AE unless they qualify as a Grade 2 or above on the Toxicity Table. The reference ranges for the laboratory will be taken in to consideration when determining out of range results.

Out-of-range test values may be considered an SAE if two circumstances are met:

1. the value rises to the level of Potentially Life-threatening (Grade 4), and
2. the event is determined by the Principal Investigator to pose an immediate risk of death.

A Grade 4 laboratory result does not, in and of itself, constitute an SAE. Grade 4 places a laboratory value as a potentially life-threatening event, but a Grade 4 laboratory result should not be reported as an SAE unless the PI has determined that it does, in fact, pose an immediate risk of death.

Assessing relatedness and clinical significance of out-of-range laboratory values: All laboratory results should be reviewedAny laboratory finding Grade 2 and above is regarded as an AE. The investigator will assess relatedness and clinical significance for these findings.

Clinical judgment always should be used to determine whether a laboratory result requires additional follow up. However, the investigator should redraw any Grade 2 or above laboratory result that is deemed clinically significant at an unscheduled visit to check if the result has normalized.

Any Grade 2 or above laboratory value that is clinically significant and has not returned to normal or stabilized by day V2+194 should be marked by the investigators as “continuing” or “unresolved.” The investigator should assure that the participant is referred for appropriate medical follow-up, if indicated.

## 9.4 Reporting Procedures

All participants must be told on definitions of AE and SAE and how they must be reported to the PI or clinical staff according to sections 9.1 and 9.3 in this protocol. ICF and Participant Diary must contain specific information on whom and what time to proceed such reports or to clarify doubts on AEs and SAEs

### 9.4.1 Serious Adverse Events (SAEs)

All SAEs must be documented and reported to Butantan or its designee, even if the investigator considers that the SAE is not related to treatment. The study clinician will complete a **Serious Adverse Event (SAE) Form** within the following timelines of such events:

- All SAEs regardless of relationship to the study products, will be reported to the Sponsor within 24 hours of site awareness.

Butantan will be primarily responsible for medical monitoring of serious adverse events documented by the investigator. Details for review of serious adverse events and other unanticipated problems will be in Safety Reporting Manual drafted prior to study initiation.

### 9.4.2 Adverse Events of Special Interest (AESIs)

All AESIs must be documented in a specific AESI report form, even if the investigator considers that the AESI is not related to treatment. If the AESI is also a SAE it should follow reporting timelines described in 9.4.1. Details for review of AESI will be in Safety Reporting Manual drafted prior to study initiation.

### 9.4.3 Reporting of AEs

Collected SAEs and AEs will be reported to responsible ethical review committees. An SOP or other written procedures for reporting to the responsible committees will be developed with reporting requirements and timelines prior to study initiation. It will be the investigator’s responsibility to assure that all reportable events are reported to the Sponsor and to the ethical committee. The Sponsor will be responsible for reporting to the regulatory agency.

### 9.4.4 Other Unexpected Issues/Unanticipated Problems

During study process, if there are any problems related to the trial (unanticipated problems), the Principal Investigator(s) is responsible for reporting to Butantan to discuss reasonable ways of handling the problem. The Principal Investigator(s) is responsible for reporting any unanticipated problems that affect the health, welfare or rights of study participants or that may impact the integrity of the study data to the ethics committees involved in the review of the research. The Principal Investigator(s) should maintain written documentation of all unanticipated problems and their reporting and resolution.

### 9.4.5 Reporting of Pregnancy

Although women are tested for pregnancy before each vaccination and are instructed to use effective birth control by Day 60 after the last dose of vaccine, it is possible for a woman to report a pregnancy during that time. Any pregnancy occurring within 60 days after the last vaccination in any participant of this study should be reported in a Pregnancy Report CRF. Relevant conditions about previous pregnancies should be reported in this CRF, if applicable. The initial report of the current pregnancy should be done in the same CRF and the correspondent fields filled in throughout the prenatal follow-up. The investigator should provide the sponsor with monthly information regarding the results of the exams and clinical conditions of the participant during the prenatal period. Any case of spontaneous abortion or stillbirth should be considered as a severe adverse event (GAS) minimally by the criterion "clinically significant event", which should be reported timely following the guidelines given in item 9.4.1. It is worthy to point out that the outcome of gestation should be reported considering the type of delivery, gestational age and conditions of the newborn, including information on congenital anomalies or birth defects. Information on the health status of the newborn during the first week of life and within forty-two days after delivery should be reported to the sponsor on the same CRF, in accordance with the recommendations of the Brazilian Ministry of Health. Allocation unbliding of those who got pregnant will not occur until the conclusion of the study, unless there is a medical indication.

## 9.5 Duration of Follow up for AE Resolution

All reported AEs should be followed until resolution or stabilization. Participants who have an ongoing study product-related SAE at study completion or at discontinuation from the study will be followed by the PI or his designee until the event is resolved or determined to be irreversible, chronic, or stable by the PI.

## 9.6 Halting Rules

Study enrollment and dosing will be halted and an ad hoc DSMB review/recommendation will be performed if any of the following occur:

Study Halting Events:

1. One or more participant experiences ulceration, abscess, or necrosis at the site of injection associated with product administration.
2. One or more participant experiences laryngospasm, bronchospasm, or anaphylaxis within 24 hours following administration of study product that is associated with product administration.
3. One or more participant experiences a study injection related SAE (excluding laboratory related SAEs, which will be managed as described in section 9.3.8).

1. Two or more participants experience generalized urticaria within 72 hours that is associated with product administration.
2. During the 7 days after each study injection, any of the following occurs across all treatment groups:

**For the first 100 subjects:**

- - Five or more participants who received at least one study injection to date experience severe (Grade 3/4) study injection-related local reaction of the same category other than measured erythema or induration.
  - Three or more participants who received at least one study injection to date experience a severe (Grade 3/4) study injection-related systemic reaction of the same category (excluding laboratory AEs, which will be monitored separately).
  - Three or more participants who received at least one study injection to date experience a severe (Grade 3) study injection-related abnormality in the same laboratory parameter.
  - Two or more participants who received at least one study injection to date experience a severe (Grade 4) study injection-related abnormality in the same laboratory parameter.

**After the first 100 subjects enrolled:**

- - 8% or more participants who received at least one study injection to date experience severe (Grade 3/4) study injection-related local reaction of the same category other than measured erythema or induration.
  - 4% or more participants who received at least one study injection to date experience a severe (Grade 3/4) study injection-related systemic reaction of the same category (excluding laboratory AEs, which will be monitored separately).
  - 2% or more participants who received at least one study injection to date experience a severe (Grade 3/4) study injection-related abnormality in the same laboratory parameter.

These halting rules refer to adverse reactions suspected to be vaccine related.

Butantan retains the right to temporarily suspend or prematurely discontinue this study at any time related to safety. If the study is stopped or suspended prematurely, Butantan will inform the local principal investigator as well as regulatory authorities about the decision and the reasons for termination or suspension. If such action is taken, all efforts must be made to ensure the safety of the participants enrolled in the study. The principal investigator will assist Butantan in informing the responsible IRB/ethics committee and provide the reason for the suspension or termination. In case of premature study or study clinic closure, the monitor will conduct all activities as indicated in the Clinical Monitoring Plan.

## 9.7 Safety Oversight

### 9.7.1 Study Safety Physician

The Study Safety Physician is a physician employed by Butantan with relevant expertise whose primary responsibility is to provide independent safety monitoring in a timely fashion. He/She will review SAEs and other AEs as needed and provide an independent assessment to the DSMB.

### 9.7.2 Data Safety and Monitoring Board (DSMB)

Safety monitoring will be closely conducted by a Data Safety and Monitoring Board (DSMB). The DSMB is appointed by but external to Butantan and is comprised of an independent group of experts with experience in clinical research studies. The primary responsibility of the DSMB is to monitor participant safety. DSMB activities will be delineated in the DSMB charter. The DSMB meets regularly throughout the study and on an ad hoc basis when required.

The DSMB will have access to unblinded data during its closed session(s), if applicable. After its assessment, they will recommend continuation, modification, or termination of the clinical trial.

# Clinical Monitoring

## 10.1 Monitoring Plan

Individuals qualified by education, training and experience will carefully monitor the study sites to ensure that the ICH/GCP guidelines are being followed. The study monitors will periodically contact the site and perform on-site visits. The extent, nature, and frequency of site visits will be based on such considerations as study objectives, study design and complexity, and enrollment rate; periodicity and nature of monitoring activities will be described in the Monitoring Plan*.* The Monitoring Plan will detail reporting requirements to Butantan to keep it apprised of study progress. Representatives of Butantan or its designees may participate in monitoring visits or visit the study clinic on its own to provide proper oversight.

# Statistical Considerations

## 11.1 Sample size

The sample size for this study is approximately 432 subjects, randomized equally into 8 treatment groups of approximately 54 subjects each. This sample size is consistent with previous phase 1 studies involving influenza vaccination, and is considered to be sufficient to evaluate preliminary safety and dose-ranging immunogenicity.

Power calcultations for the detection of adverse events by study arms are described in Table 8.

- In each of the active arms (N = 54), there is a 94% power to detect an event with frequency ≥ 5%.
- For all combined dose levels of vaccine adjuvanted with IB160 (N = 162) or SE (N = 162) there is a 80% power to detect an event with frequency ≥ 1%.

**Table 8. Safety – Statistical Power for Detecting at Least 1 Safety Event Based on a Range of True Event Rates**

| **True Event Rate (%)** | **N = 54** | **N = 162** |
| --- | --- | --- |
| 0.1 | 5% | 15% |
| 0.5 | 24% | 55% |
| 1 | 42% | 80% |
| 2 | 66% | 96% |
| 3 | 81% | 99% |
| 4 | 89% | >99% |
| 5 | 94% | >99% |

Sample size and statistical power were determined using PASS® v. 15.0.2. Calculations can be checked using the following binomial distribution formula: Power (%) = (1-(1-taxa real de evento)^N). Considering true real rate of event = 0.5% and N=54, Power (5) = 100*(1-(1-0.005)^54)=23.7% turning to 24%.

## 11.2 Data Analysis

The planned statistical analyses for this study are outlined below. A detailed statistical analysis plan for preparation of the final study report will be created and made final prior to database lock and unblinding. Butantan and IDRI will participate in the review and approval of the statistical analysis plan.

Antibody titers will be transformed into log10 for the calculation of their geometric mean, and mean geometric increase. Adverse events will have their individual percentages calculated for each intervention group. Results will be summarized by point estimates, and respective 95% confidence intervals (CI). Exact (Clopper-Pearson) CIs will be calculated for all proportional endpoints. The distribution of HI antibody titers from each intervention group will be described by reverse cumulative distribution curves.

## 11.3 Definition of Analysis Sets

Definitions of populations to be analyzed are:

Enrolled Population

All screened participants who provide informed consent, regardless of the participant’s randomization and treatment status in the trial.

Full Analysis (FA) Population

All participants in the enrolled population who were randomized and received a study vaccination. This population will serve as the primary analysis population for all safety objectives and may serve also in secondary immunogenicity analysis. The analysis based on this population will serve as the supportive results for all safety objectives. Participants will be analyzed as received..

Per Protocol (PP) population

All participants in the Full Analysis population who have valid post vaccination immunogenicity measures with no major protocol deviations that are determined to potentially interfere with the immunogenicity assessment of the study vaccine. This population will serve as the primary analysis population for all immunogenicity objectives.

The criteria for exclusion of participants from the Per Protocol Population will be established before breaking the blind and will be based on the blinded review of major protocol deviations.

## 11.4 Analysis of Immunogenicity Endpoints

Immune responses to the vacccine will be evaluated by the following:

- the number of participants with a serum HI antibody titer ≥ 1:40 post-vaccination measured on days V1+28, (+7d), V2+7 (+3d) e V2+28 (+7d);;
- the number of seroconverted participants; that is with a serum HI titer on days V1+28, (+7d), V2+7 (+3d) e V2+28 (+7d);meeting the following criteria:
  - pre-vaccination titer <1:10 and a post-vaccination titer ≥ 1:40 or
  - pre-vaccination titer ≥ 1:10 and at least a four-fold increase in post-vaccination measured.
- the geometric mean titers (GMTs) of Serum Hemagglutination Inhibition (HI) antibodies pre- (V1) and post-vaccination (V1+2, (+7d), V2+7 (+3d) e V2+28 (+7d);
- the geometric mean fold rises (GMFRs) of Serum Hemagglutination Inhibition (HI) antibodies (post-vaccination / pre-vaccination).
- the number of participants with a serum MNT antibody titer ≥ 1:40 post-vaccination measured on day V1+28 and V2+28 (+7d);
- the number of seroconverted participants; that is with a serum MNT titer on day V1+28 and V2+28 (+7d); meeting the following criteria:
  - pre-vaccination titer <1:10 and a post-vaccination titer ≥ 1:40 or
  - pre-vaccination titer ≥ 1:10 and at least a four-fold increase in post-vaccination measured.
- the geometric mean titers (GMTs) of MNT antibodies pre- (V1) and post-vaccination V1+28 and V2+28 (+7d);
- the geometric mean fold rises (GMFRs) of MNT antibodies (post-vaccination / pre-vaccination).

Percentages of participants with immune response will be calculated along the corresponding two-sided exact CIs. GMT will be summarized by treatment group along with the corresponding two-sided 95% CIs, by exponentiating the corresponding log-transformed means and their 95% CIs. Titers below the lowest limit of quantitation (i.e. below the starting dilution of assay reported as “< 10”) will be set to half that limit (i.e. 10/ 2 = 5). If a titer is reported as greater or equal to the upper limit of the assay, it will be set to that limit.

## 11.5 Analysis of Safety Endpoints:

The safety profiles will be evaluated by the proportion of subjects experiencing AEs, related or not related, of the following four categories:

- Immediate reactions occurring within 30 minutes of administration of any dose, measured as observed by study staff or reported by the subject to study staff.
- Adverse events (reactogenicity) commonly associated with intramuscular vaccination (solicited local and systemic reactions) occurring through 7 days following any dose, measured as observed by study staff or reported by the subject to study staff.
- All other adverse events (including unsolicited events) following any dose, measured as observed by study staff or reported by the subject to study staff over the entire study period. This includes clinical findings and abnormal laboratory findings from blood specimens collected on Days 7 and 35.
- All serious adverse events (SAEs) occurring throughout the study period, as observed by study staff, reported by the subject to study staff, or noted by the subject diary card. This includes clinical findings and Grade 4 abnormal laboratory findings from blood specimens collected on Days 7 and 35 considered as life threatening.
- All adverse events of special interest (AESIs) occurring throughout the study period, as observed by study staff, reported by the subject to study staff, or noted by the subject diary card. This includes clinical findings and Grade 4 abnormal laboratory findings from blood specimens collected on Days 7 and 35 considered as life threatening.

Counts of all events will be reported and summarized according to event severity, as “any local AE”, or “any systemic AE”, and by relationship to administration of study product, as deemed by a blinded study clinician. Percentages of subjects experiencing each reaction or event, or at least one reaction or event will be calculated along with two-sided exact 95%CIs.

## 11.6 Interim analysis

The DSMB will conduct a formal review (first interim analysis) of the safety data of the first 100 participants (visit S1, safety data). They will either recommend continued enrollment of the remaining participants, modification, or discontinuation of enrollment. Enrollment may continue while this review is being conducted, if no halting rules have been met. The review is not required to be completed prior to receipt of the second study injection unless one of the halting rules has been met.

The DSMB will conduct a formal review (second interim analysis) of the safety data of all participants of the study after safety visit I. This analysis will also include preliminary immunogenicity data to support the decision of the Sponsor for the vaccine candidate in each adjuvant group that will move forward to a future Phase II.

# Data Handling and Record Keeping

## 12.1 Data Capture Methods

This study uses an electronic data capture system - OpenClinica® Enterprise version 3.1 or later

*eCRF*

Electronic CRFs will be developed and data will be managed to the extent possible in accordance with internationally agreed-upon standards, such as the Clinical Data Acquisition Standards Harmonization, which defines basic standards for the collection of clinical trial data, and/or The Society for Clinical Data Management’s Good Clinical Data Management Guidelines.

All the information required by the study protocol will be recorded in eCRF. All data must be derived from source documents. investigator site staff will enter and edit the data via a secure network, with secure access features (username, password and secure identification or username and password – an electronic password system). A complete electronic audit trail will be maintained. The investigator will approve the data using an *electronic signature* (Ref: 21 TheCFR Part 11), and this approval is used to confirm the accuracy of the data recorded. In case electronic CRFs (eCRFs) will be used, the investigator’s data will be accessible from the investigator’s site throughout the trial.

The electronic CRFs must be kept current to reflect patient status at each phase during the course of the trial. The patients must not be identified on the electronic CRF by name. Appropriate coded identification (i.e. Participant Number) must be used. The investigator must make a separate confidential record of these details (participant identification code list) to permit identification of all patients enrolled in a clinical trial in case follow-up is required.

All changes will be requested from the investigator through the EDC system. The investigator’s signature is requested to show he/she agrees with the change that was made. The investigator will be responsible for retaining all records pertaining to the trial as specified in the appropriate contract.

Clinical safety data will be entered onto study CRFs from laboratory report forms. Visit dates and laboratory procedure dates will be recorded on all forms. All participants will be assigned a unique screening number and participant identification number at study enrollment – one or both of these numbers will be included in the CRF and in the trial database and will serve to link study data to specific individuals. CRFs will be entered, verified for accuracy, linked by study participant number, and managed using database management software, with proper security and controls and the ability to identify who is entering or making changes to the data in the system.

*Source data and Participant Files*

Source documents provide evidence for the existence of the participant and substantiate the integrity of the data collected. Source documents are filed at the investigator’s site.

The investigator has to keep a written participant file for every participant participating in the clinical study. In this participant file, the available demographic and medical information of a participant is documented, including but not limited to: name, date of birth, sex, height, weight, participant history, concomitant diseases and concomitant drug(s) (including changes during the study), statement of entry into the study, study identification, randomization number, the date of informed consent, all study visit dates, predefined performed examinations and clinical findings, observed AEs, and reason for withdrawal from the study, if applicable.

It should be possible to verify the inclusion and exclusion criteria for the study from the available data in this file.

It must be possible to identify each participant by using this participant file. Additionally, any other documents with source, especially original printouts of data that were generated by technical equipment have to be filed. This includes laboratory reports. All these documents have to bear at least the participant identifier and the printing date printed by the recording device to indicate to which participant and to which study procedure the document belongs. The medical evaluation of such records should be documented as necessary, signed/initialed and dated by the investigator.

Data reported on the CRFs that are transcribed from source documents must be consistent with the source documents or the discrepancies must be explained.

## 12.2 Database Management and Analysis Software

Data Management will be performed by Butantan using a Data Management Plan.

All statistical analyses will be performed using a recent version of STATA.

Medical history and AEs will be coded using a recent version of the MedDRA dictionary. The frequency count and percentage of participants will be summarized according to the coded terms of system organ class and preferred term.

## 12.3 Source Documents and Source Document Access

Prior to the start of the trial, the sponsor will determine which documents or data fields completed by the investigative team will be considered source documents and documented on a Source Documentation Table. Source documents for this study may be outpatient charts, inpatient charts, laboratory analysis forms, questionnaires, and specimen collection logs.

Only authorized study staff and representatives of Butantan, study monitors authorized by Butantan, members of ethics review committees, and regulatory agencies may have direct access to source documents containing participant data. Participant identification will be revealed to authorized representatives of these organizations only when necessary.

# Quality Control and Quality Assurance

The study will be conducted in accordance with the procedures specified in the protocol and according to GCP. Study data collection forms will be designed to guide staff on study conduct; forms also will include areas for documenting that activities did, in fact, occur (even if these activities did not require recording of data) and in the appropriate sequence. All study staff must attend mandatory protocol implementation training prior to participant enrollment.

Study data are recorded on eCRFs throughout the study. After data have been entered in the study database, they are checked systematically by data management staff according to a pre-specified data validation plan. Queries are generated for site staff to clarify or correct throughout the study. An audit trail will be kept of all changes to the data. All listings of the database will be reviewed and discussed for assessment of consistency and medical plausibility during an on-going data and medical review. After resolution of all issues the database will be locked.

# Ethics/Protection of Human Participants

## 14.1 Ethical Standard

This study will be conducted in full conformity with any Brazilian regulations or guidelines for clinical trials and with the Declaration of Helsinki in order to ensure the best protection for study participants.

## 14.2 Financing and Insurance

The WHO and IDRI will fund this trial through grants provided by BARDA, funding may also be supplemented by the Butantan Foundation and/or Instituto Butantan. Any financial engagement with the clinical study sites will be regulated by a separate agreement established by Butantan.

Butantan will maintain the insurance to cover treatment for study related injuries to meet its ethical or regulatory obligations.

## 14.3 Assurance of Emergency Medical Care and Care for other Adverse Events

Study participants will be observed by qualified clinicians after each vaccination, and emergency care will be immediately available to participants who need it. If additional urgent care or resources are needed, depending on each case, the participant will be provided urgent care and transported to a hospital at higher level if needed.

## 14.4 Independent Ethics Committee and Regulatory Approval

No human participant research activities will be conducted without the review and approval of all relevant ethics committees of the entities involved in the study. The protocol and all amendments will have initial and continuing review and approval at independent local ethics committees at each site (LECs), the WHO Ethics Review Committee (ERC) and the National Committee for Research Ethics (CONEP).

This study will be reviewed and approved by ANVISA who will also review all amendments before implementation, as appropriate.

The PI or designee shall maintain copies of all application documents and forward copies of all LEC documents and approvals to Butantan prior to the start of the study. The approval letters must identify all documents approved and list the study clinic, the study investigator, protocol title, version number, and date and, Informed Consent Form (ICF) version number and date, and the date of ethics approval. The PI will sign all approved versions of the protocol.

The PI is responsible for notifying the LEC of problems related to risks for participants, according to national ethical requirements.

Butantan will report to ANVISA and the CONEP any new information related to the study vaccine which possibly affect the safety of participants or their risk/benefit ratio for participating in this trial.

The PI will be responsible for reporting to the LECs when the clinical study has been completed.

## 14.5 Informed Consent Process

The following issues must be included in discussions with potential participants prior to obtaining their informed consent to participate in the study:

- The purpose of research, the time duration that the participants need to participate, the procedures involved in the study, the scientific evidence that justifies conducting this experimental trial in humans, and the potential risks and known benefits of participating in the research.
- Randomization and that participants will have a chance of receiving either two doses of study vaccine or the placebo.
- That the study staff conducting the follow up will not know which study product (vaccine or placebo) the participant will receive and that the study staff and participant will have no way to choose which product is received by the participant.
- That the participant’s participation is voluntary, and the participant’s refusal to participate will not result in any fine or in any loss of rights or access to medical care that the participant is normally already entitled receive.
- That in the event of unforeseen circumstances or needs, the investigator may decide to withdraw the participant from continued participation in the research even without the consent of the participant.
- That the participant will be provided with the results of any new important findings related to the trial or the study vaccine which may influence the participant’s decision on whether to continue to participate in the research.
- That the participant will be told the total number of participants in the research.
- That the participant will be told who to notify as the point of contact with the investigator and LECs in case the research participant would like to know more information regarding the trial and his/her rights as a research participant.
- That the investigator is responsible for collecting from the participant signed and dated written informed consent forms regarding participation in the study before the participant may participate in the research; that the participant will be given a signed and dated copy of the form to keep; and that the investigator must keep the original signed and dated informed consent form in the investigator’s research files.

Written informed consent of the participant must be obtained before performing any trial procedures. Participants will be made aware that authorized representatives of health agencies Butantan, and authorized representatives of partnering institutions and funders (e.g., IDRI, BARDA, WHO) will have access to their confidential study information for the purposes of monitoring trial conduct or performing audits.

ICFs will embody the elements of consent as described in the Declaration of Helsinki and the ICH Harmonized Tripartite Guidelines for Good Clinical Practice. Original ICFs must be kept on file by the investigator for possible inspection. The participant must receive a copy (or second original) of the signed and dated ICF(s), and any subsequent updates or amendments to the ICF.

If approved by the local ethics committee, participants will be informed that they will be compensated for travel to and from the sites for the study visits including the travel expenses of a companioin if required. A snack will be offered to the volunteers on the days of visiting the center.

.

## 14.6 Participant Confidentiality

### 14.6.1 Confidentiality of Data

By signing the protocol, the Principal Investigator agrees that the study protocol, documentation, data, and all other information generated regarding the vaccines will be held in strict confidence. The investigator may divulge such information within regulatory restrictions and ethical considerations only to the local ethical review committee, to the Sponsor and to the study DSMB. No information concerning the study or the data may be released to any unauthorized third party without prior written approval of Butantan. Any regulatory agency deemed appropriate, may consult study documents in order to verify CRF data. Investigators will ensure that all employees involved in the study respect confidentiality.

Medical information about individual participants obtained during the course of this study is confidential and may not be disclosed to third parties, except authorized monitors, auditors or inspectors or as a required by law. Confidentiality will be ensured by the use of study participant numbers for the identification of each participant; these study participant numbers will also be used for participant data in the participant files at the site and for the CRFs.

### 14.6.2 Confidentiality of Participant Records

Participant confidentiality is strictly held in trust by the participating investigator and his or her staff. This confidentiality is extended to cover testing of biological specimens in addition to the clinical information relating to participating participants.

Study participants will not be identified by name on any data collection form or on any other documentation sent to Butantan and will not be reported by name in any report or publication resulting from data collected in this study.

Documents and data pertaining to the study will be kept in a locked room or in locked files under the responsibility of the Principal Investigator. Monitors will conduct periodic monitoring visits to ensure that the data are stored securely. Only study clinicians, study staff, monitors or the sponsor or its designee will be granted access to the study data and records.

The PI will keep individual data confidential to the extent permitted by law. Information will not be released to anyone other than the participant unless required to do so by law or directed by the participant (e.g., to release information to his or her health care provider).

## 14.7 Sharing of Study Results

### 14.7.1 Sharing of Study Results with the Participant

All results of clinical screening and safety laboratory testing will be shared with each participant or made available for review by the participant. After study completion a letter will be sent to participants thanking them for participating in the trial and informing them of their arm allocation, as well as a summary of study findings on safety and immunogenicity of vaccine..

### 14.7.2 Incidental Health Findings

The Investigator will share and discuss any incidental health findings with the participant and help the participant seek proper medical follow-up. The investigator may release participant clinical and laboratory results data to the participant’s primary care physician for incidental health findings with the agreement of the participant.

## 14.8 Biological samples storage and use of biorepository

All biological material collected during the study may, with the participant's permission, be stored in a biorepository for a maximum of 10 years for use in future research. Biological material will not be marketed or used for the development of commercial products according to the Brazilian standards for human participants research and storage of samples, including the prohibition of marketing or patent application related to these samples. All biological material will be stored and will be identified with the participant identification received by the participant after their inclusion in the study.

Samples may be exported to laboratories in the USA for validation of results and improvement of the immunogenicity tests if required by authorized representatives of partnering institutions and funders (e.g., IDRI, BARDA). Butantan will have unrestricted access to the data from the use of exported samples and the Brazilian standards for human participants research and storage of samples will be respected, including the prohibition of marketing or patent application related to these samples.

Any new research project outside of the scope of this study to be conducted with the stored material will be submitted for approval by the ethics committees. The procedures for the management of the biorepository will follow the guidelines of Brazilian Resolution No. 441 of the CNS^[[23]](#endnote-24)^.

## 14.9 Potential Risks and How They are Addressed

**Physical Risks**: The most frequent risks of this study are risks of physical injury at the injection site or blood withdrawal site. Administration of study product may cause the participant immediate mild pain in the arm. Other risks include systemic symptoms such as fever and body aches. These are all explained to participants in the consent form.

Serious or allergic reactions also may be possible. This risk is addressed by trying to screen out people who have had an allergic reaction to vaccines in the past and who may have an allergy to one of the components of the vaccine. Should an allergic reaction occur with vaccination, the study clinic will follow its SOP for handling medical emergencies and have supportive medicines in place, in addition to trained staff.

Besides administration of study product, collection of blood specimens may cause some discomfort to participants. Venipuncture is sometimes associated with fainting, discomfort or pain, bleeding, bruising, redness, swelling, local hardness, and/or infection at the puncture site. This risk is addressed by having trained staff draw the blood.

Study participants will be observed closely by qualified clinicians and care will be immediately available to participants after vaccination, including emergency care, if needed. If additional urgent care or resources are needed, the participant will be transported to a local hospital. This hospital will be identified by the investigator prior to study initiation. The study will provide this care to the participant at no cost to the participant.

In the case of expected and unexpected reactions after the use of vaccines, study participants will receive appropriate medical care and treatment immediately. Butantan will be responsible for ensuring medical cover for conditions that are directly attributable to the participants involvement in the study. This study is supported by an insurance policy covering damage to participants related to the use of the investigational product in order to meet all ethical duties.

During the study period if serious adverse event occurs that is related to the participant’s participation in the trial or to the study products, Butantan will ensure coverage for the full cost of immediate treatment according to the laws of Brazil for research participants. Study participants will be compensated for any damages resulting from the research.

**Risks to Privacy:** Anyone participating in research using their real name and medical information can face a loss of privacy. These risks are mitigated by using unique identification numbers in place of a participant’s name, restricting access to study information, and not naming or identifying a participant in any publication.

## 14.10 Benefits to Study Participants

Study participants may not benefit from being in the study, however, there is potential for some benefit:

- People may benefit from the physical exams and laboratory testing that is done in the study, as it may reveal information about their health that they did not know before.
- Participants may develop an immune response to the H7N9 candidate vaccines.

# Clinical Study Report and Publication Policy

## 15.1 Clinical Study Report

A Clinical Study Report (CSR) comprised of text and results tables reflecting all safety and immunogenicity data will be generated by Butantan. The CSR will be compliant with ICH E: 3 guidelines.

All data, documents, any recordings and information transferred to any contractor or obtained or prepared by any contractor, his/her consultants or persons associated by contractual relationships with any contractor during the trials, belong to Butantan and will be shared with partners as detailed in technical agreements.

Following completion of the clinical study report, the investigators, working with Butantan and representatives, are expected to publish the results, negative or positive, of this research in peer-reviewed scientific journal(s). Butantan may not prohibit the public dissemination of the results of this trial.

## 15.2 Publication Policy

The International Committee of Medical Journal Editors (ICMJE) member journals have adopted a trials-registration policy as a condition for publication. This policy requires that all clinical trials be registered in a public trials registry. It will be the responsibility of Butantan to register this trial in an acceptable registry. ICMJE authorship criteria will be strictly followed for publication of any manuscript(s) arising from this trial. In addition, this trial, since funded by WHO, will follow the WHO Open Access Policy, which makes research funded by WHO and published in journals available to the public without a subscription necessary.

# Annex A: List of Adverse Events of Special Interest

Gastrointestinal disorders

- Celiac disease
- Crohn’s disease
- Ulcerative colitis
- Ulcerative proctitis

Liver disorders

- Autoimmune cholangitis
- Autoimmune hepatitis
- Primary biliary cirrhosis
- Primary sclerosing cholangitis

Metabolic disease

- Addison’s disease
- Autoimmune thyroiditis (including Hashimoto thyroiditis)
- Diabetes mellitus type I
- Grave’s or Basedow’s disease

Musculoskeletal disorders

- Antisynthetase syndrome
- Dermatomyositis
- Juvenile chronic arthritis (including Still’s disease)
- Mixed connective tissue disorder
- Polymyalgia rheumatic
- Polymyositis
- Psoriatic arthropathy
- Relapsing polychondritis
- Rheumatoid arthritis
- Scleroderma, including diffuse systemic form and CREST syndrome
- Spondyloarthritis, including ankylosing spondylitis, reactive arthritis (Reiter’s Syndrome) and undifferentiated spondyloarthritis
- Systemic lupus erythematosus
- Systemic sclerosis

Neuroinflammatory disorders

- Acute disseminated encephalomyelitis, including site specific variants (e.g. non-infectious encephalitis, encephalomyelitis, myelitis, myeloradiculomyelitis)
- Cranial nerve disorders, including paralyses/paresis (e.g.Bell’s palsy)
- Guillain-Barré syndrome, including Miller Fisher syndrome and other variants
- Immune-mediated peripheral neuropathies and plexopathies, including chronic inflammatory demyelinating polyneuropathy, multifocal motor neuropathy and polyneuropathies associated with monoclonal gammopathy
- Multiple sclerosis
- Narcolepsy
- Optic neuritis
- Transverse Myelitis

Skin disorders

- Alopecia areata
- Autoimmune bullous skin diseases, including pemphigus, pemphigoid and dermatitis herpetiformis
- Cutaneous lupus erythematosus
- Erythema nodosum
- Morphoea
- Lichen planus
- Psoriasis
- Sweet’s syndrome
- Vitiligo

Vasculitides

- Large vessels vasculitis including: giant cell arteritis such as Takayasu’s arteritis and temporal arteritis.
- Medium sized and/or small vessels vasculitis including: polyarteritis nodosa, Kawasaki’s disease, microscopic polyangiitis, Wegener’s granulomatosis, Churg-Strauss syndrome (allergic granulomatous angiitis), Buerger’s disease thromboangiitis obliterans, necrotizing vasculitis and anti-neurophil cytoplasmic antibody (ANCA) positive vasculitis (type unspecified), Henoch–Schönlein purpura, Behçet's syndrome, leukocytoclastic vasculitis.

Others

- Antiphospholipid syndrome
- Autoimmune hemolytic anemia
- Autoimmune glomerulonephritis (including IgA nephropathy, glomerulonephritis rapidly progressive, membranous glomerulonephritis, membranoproliferative glomerulonephritis, and mesangial proliferative glomerulonephritis)
- Autoimmune myocarditis/cardiomyopathy
- Autoimmune thrombocytopenia
- Goodpasture syndrome
- Idiopathic pulmonary fibrosis
- Pernicious anemia
- Raynaud’s phenomenon
- Sarcoidosis
- Sjögren's syndrome
- Stevens-Johnson syndrome
- Uveitis

# References

1. Francis DP, Du YP, Precioso AR. Global vaccine supply. The increasing role of manufacturers rom middle income countries. Vaccine 2014;32:5259–65. doi:10.1016/j.vaccine.2014.07.069. [↑](#endnote-ref-2)
2. Miyaki C, Meros M, Precioso AR, Raw I. Influenza vaccine production for Brazil: A classic example of successful North-South bilateral technology transfer. Vaccine 2011;29. doi:10.1016/j.vaccine.2011.04.127. [↑](#endnote-ref-3)
3. Treanor JJ, Chu L, Essink B, Muse D, El Sahly HM, Izikson R, Goldenthal KL, Patriarca P, Dunkle LM., Stable emulsion (SE) alone is an effective adjuvant for a recombinant, baculovirus-expressed H5 influenza vaccine in healthy adults: A Phase 2 trial. Vaccine. 2017 Feb 7;35(6):923-928. doi: 10.1016/j.vaccine.2016.12.053 [↑](#endnote-ref-4)
4. Precioso AR, Miraglia JL, Campos LMA, Goulart AC, Timenetsky M do CST, Cardoso MRA, et al. A phase I randomized, double-blind, controlled trial of 2009 influenza A (H1N1) inactivated monovalent vaccines with different adjuvant systems. Vaccine 2011;29:8974–81. doi:10.1016/j.vaccine.2011.09.040. [↑](#endnote-ref-5)
5. Miraglia JL, Abdala E, Hoff PM, Luiz AM, Oliveira DS, Saad CGS, et al. Immunogenicity and reactogenicity of 2009 influenza a (H1N1) inactivated monovalent non-adjuvanted vaccine in elderly and immunocompromised patients. PLoS One 2011;6. doi:10.1371/journal.pone.0027214. [↑](#endnote-ref-6)
6. Pasoto SG, Ribeiro AC, Viana VST, Leon EP, Bueno C, Neto ML, et al. Short and long-term effects of pandemic unadjuvanted influenza A(H1N1)pdm09 vaccine on clinical manifestations and autoantibody profile in primary Sj??gren’s syndrome. Vaccine 2013;31:1793–8. doi:10.1016/j.vaccine.2013.01.057. [↑](#endnote-ref-7)
7. Aikawa NE, Campos LMA, Goldenstein-Schainberg C, Saad CGS, Ribeiro AC, Bueno C, et al. Effective seroconversion and safety following the pandemic influenza vaccination (anti-H1N1) in patients with juvenile idiopathic arthritis. Scand J Rheumatol 2013;42:34–40. doi:10.3109/03009742.2012.709272. [↑](#endnote-ref-8)
8. França ILA, Ribeiro ACM, Aikawa NE, Saad CGS, Moraes JCB, Goldstein-Schainberg C, et al. TNF blockers show distinct patterns of immune response to the pandemic influenza A H1N1 vaccine in inflammatory arthritis patients. Rheumatology (Oxford) 2012;51:2091–8. doi:10.1093/rheumatology/kes202. [↑](#endnote-ref-9)
9. Borba EF, Saad CGS, Pasoto SG, Calich ALG, Aikawa NE, Ribeiro ACM, et al. Influenza A/H1N1 vaccination of patients with SLE: Can antimalarial drugs restore diminished response under immunosuppressive therapy? Rheumatol (United Kingdom) 2012;51:1061–9. doi:10.1093/rheumatology/ker427. [↑](#endnote-ref-10)
10. Ribeiro ACM, Guedes LKN, Moraes JCB, Saad CGS, Aikawa NE, Calich AL, et al. Reduced seroprotection after pandemic H1N1 influenza adjuvant-free vaccination in patients with rheumatoid arthritis: implications for clinical practice. Ann Rheum Dis 2011;44:6–10. doi:10.1136/ard.2011.152983. [↑](#endnote-ref-11)
11. Saad CGS, Borba EF, Aikawa NE, Silva C a, Pereira RMR, Calich AL, et al. Immunogenicity and safety of the 2009 non-adjuvanted influenza A/H1N1 vaccine in a large cohort of autoimmune rheumatic diseases. Ann Rheum Dis 2011;70:1068–73. doi:10.1136/ard.2011.150250. [↑](#endnote-ref-12)
12. Azevedo LS, Gerhard J, Miraglia JL, Precioso AR, Tavares Timenetsky MDCS, Agena F, et al. Seroconversion of 2009 pandemic influenza A (H1N1) vaccination in kidney transplant patients and the influence of different risk factors. Transpl Infect Dis 2013;15:612–8. doi:10.1111/tid.12140. [↑](#endnote-ref-13)
13. Gao R, Cao B, Hu Y, Feng Z, Wang D, Hu W, et al. Human infection with a novel avian-origin influenza A (H7N9) virus. N Engl J Med 2013;368:1888–97. doi:10.1056/NEJMoa1304459. [↑](#endnote-ref-14)
14. WHO Influenza at the human-animal interface: Summary and assessment, 17 January to 14 February 2017available here: http://www.who.int/influenza/human_animal_interface/Influenza_Summary_IRA_HA_interface_02_14_2017.pdf?ua=1 (accessed on 3 March 2017) [↑](#endnote-ref-15)
15. Qi X, Qian Y-H, Bao C-J, Guo X-L, Cui L-B, Tang F-Y, et al. Probable person to person transmission of novel avian influenza A (H7N9) virus in Eastern China, 2013: epidemiological investigation. BMJ 2013;347:f4752. doi:10.1136/bmj.f4752. [↑](#endnote-ref-16)
16. Madan A, Segall N, Ferguson M, Frenette L, Kroll R, Friel D, Soni J, Li P, Innis BL, Schuind A. Immunogenicity and Safety of an AS03-Adjuvanted H7N9 Pandemic Influenza Vaccine in a Randomized Trial in Healthy Adults. J Inf Dis 2016; 214:1717-1727. Epub 2016 Sep 7 [↑](#endnote-ref-17)
17. Mulliban MJ, Bernstein DI, Winokur P, Rupp R, Anderson E, Rouphael N, Dickey M, et al. Serologic Responses to an Avian Influenza A/H7N9 Vaccine Mixed at the Point-of-use with MF59 Adjuvant: A Randomized Clinical Trial. JAMA 2014; 312:1409-1419. [↑](#endnote-ref-18)
18. Jackson L, Campbell JD, Frey S, Edwards KM, Keitel W, Kotloff KL, Berry A. et al. Effect of Varying Doses of a Monovalent H7N9 Influenza Vaccine with and without AS03 and MF59 Adjuvants on Immune Response: A Randomized Clinical Trial. JAMA 2015; 314:237-246. [↑](#endnote-ref-19)
19. H7N9 Mix and Match with MF59 in Healthy Elderly Persons. ClinicalTrials.gov Identifier: NCT02213354 [↑](#endnote-ref-20)
20. CDC information on FluAd: <https://www.cdc.gov/flu/protect/vaccine/adjuvant.htm> accessed on 16 February 2017 [↑](#endnote-ref-21)
21. Reference: L.H. Martín Arias, R. Sanz, M. Sáinz, C. Treceño, A. Carvajal, Guillain-Barré syndrome and influenza vaccines: A meta-analysis, Vaccine, Volume 33, Issue 31, 17 July 2015, Pages 3773-3778, ISSN 0264-410X, <http://dx.doi.org/10.1016/j.vaccine.2015.05.013>. [↑](#endnote-ref-22)
22. US FDA Guidance for Industry: Toxicity Grading Scale for Healthy Adult and Adolescent Volunteers Enrolled in Preventive Vaccine Clinical Trials FDA <http://www.fda.gov/BiologicsBloodVaccines/GuidanceComplianceRegulatoryInformation/Guidances/Vaccines/ucm074775.htm> [↑](#endnote-ref-23)
23. CNS/MS Resolução CNS Nº 441 [Internet]. Brasília: Conselho Nacional de Saúde; 2011 [accessed on 16 February 2017]. Available here: <http://conselho.saude.gov.br/resolucoes/2011/Reso441.pdf> [↑](#endnote-ref-24)
